# Supplementary material for: Sanitary Waters: Is It Worth Looking for Mycobacteria?
Source: Microorganisms. 2024 Sep 27;12(10):1953. doi: 10.3390/microorganisms12101953 (PMC11509539; doi:10.3390/microorganisms12101953)
Supplement: Supplementary file 1 [file microorganisms-12-01953-s001.zip › microorganisms-3145239-supplementary.pdf]

Table S1

ID	Completeness	Contamination	Coding_Density	Contig_N50	Average_Gene_Length	Genome_Size	GC_Content	Total_Coding_Sequences	Total_Contigs	Max_Contig_Length	CDS	Number of rRNA operons	tRNA genes	Genome Accession number assembly
SP1973	99,99	2,95	0,899	187118	315,5756667	6307095	0,68	6000	141	362858	5917	3	82	JBHHNS000000000
SP1974	99,99	2,95	0,899	134483	315,5437573	6306206	0,68	5999	148	362858	5916	3	82	JBHHNR000000000
SP1975	99,99	2,95	0,899	157577	316,2235392	6311731	0,68	5990	138	349127	5915	3	82	JBHHNQ000000000
SP1976	99,99	2,95	0,898	162374	316,1289623	6315489	0,68	5994	143	349127	5912	3	83	JBHHNP000000000
SP1977	99,99	2,95	0,898	180165	316,316018	6312825	0,68	5987	144	349127	5909	3	82	JBHHNO000000000
SP1978	100	3,22	0,898	187108	315,9480202	6466201	0,68	6137	145	362858	6065	3	83	JBHHNN000000000
SP1979	99,99	2,99	0,898	182405	315,741914	6311626	0,68	5998	146	362454	5923	3	82	JBHHNM000000000
SP1980	99,99	2,95	0,898	130958	314,798536	6305973	0,68	6011	153	347529	5924	3	82	JBHHNL000000000
SP1981	99,99	3,19	0,898	102025	311,9441883	6314053	0,68	6074	213	275764	5933	3	82	JBHHNK000000000
SP1982	99,99	3,18	0,898	134489	314,0039761	6318748	0,68	6036	173	362858	5945	3	82	JBHHNJ000000000
SP1983	99,99	2,95	0,898	125269	314,6290885	6316577	0,68	6023	151	389685	5934	3	82	JBHHNI000000000
SP1984	99,99	2,95	0,898	173225	316,4674349	6316014	0,68	5988	129	602067	5928	3	82	JBHHNH000000000
SP1985	99,99	3	0,898	173119	315,2749044	6317525	0,68	6013	149	522394	5921	3	82	JBHHNG000000000
SP1986	99,99	3	0,898	141325	315,5112332	6319030	0,68	6009	146	361229	5922	3	82	JBHHNF000000000
SP1987	99,99	3,01	0,898	141325	315,1408146	6316650	0,68	6015	153	362454	5929	3	82	JBHHNE000000000
SP1988	99,99	3,24	0,898	113284	314,2602058	6311470	0,68	6026	163	361953	5932	3	82	JBHHND000000000
SP1989	100	3,76	0,899	120615	314,3959854	6314726	0,68	6028	166	362856	5942	3	82	JBHHNC000000000
SP1990	99,99	2,93	0,898	173163	315,9351775	6317568	0,68	6001	137	460667	5923	3	82	JBHHNB000000000
SP1991	99,99	2,99	0,898	139403	314,3903814	6323918	0,68	6030	187	362454	5920	3	82	JBHHNA000000000
SP1998	99,99	3,23	0,898	114273	310,2750203	6359827	0,68	6145	259	362454	5988	3	82	JBHHMZ000000000
SP1999	99,99	2,95	0,898	141325	314,9343636	6321921	0,68	6018	166	362452	5925	3	82	JBHHMY000000000
SP2000	99,99	2,91	0,897	144860	312,0789732	6367888	0,68	6116	232	460578	5976	3	82	JBHHMX000000000
SP2044	99,99	3	0,897	173168	311,5225225	6350830	0,68	6105	293	362858	5931	3	82	JBHHMW000000000
SP2046	99,99	3	0,897	177477	312,2846823	6345905	0,68	6091	250	362858	5930	3	82	JBHHMV000000000
SP2043	99,99	3	0,897	173168	311,5225225	6350830	0,68	6105	293	362858	5933	3	82	JBHHMU000000000
SP2042	99,99	3,41	0,896	102947	310,9308877	6341663	0,68	6106	281	362858	5938	3	85	JBHHMT000000000
SP2047	99,99	2,95	0,898	187118	313,7348322	6327416	0,68	6049	195	469486	5931	3	82	JBHHMS000000000
SP2049	99,99	2,97	0,898	127953	312,7407834	6333647	0,68	6076	216	362858	5937	3	82	JBHHMR000000000
SP2050	99,99	2,95	0,898	182400	313,3577316	6339735	0,68	6066	223	472035	5935	3	82	JBHHMQ000000000
SP2048	99,99	2,99	0,897	120615	311,3846028	6342258	0,68	6105	262	341834	5942	3	82	JBHHMP000000000
SP2052	99,99	2,95	0,898	177572	314,4579331	6333358	0,68	6038	202	471985	5925	3	82	JBHHMO000000000
SP2051	99,99	3	0,898	157612	314,0170248	6334894	0,68	6050	204	362454	5924	3	83	JBHHMN000000000
SP2053	99,99	3	0,897	198256	313,2588991	6345854	0,68	6068	247	362858	5933	3	82	JBHHMM000000000
SP2045	99,99	2,95	0,896	141325	310,6982548	6366347	0,68	6131	325	362856	5943	3	82	JBHHML000000000
SP2041	99,99	3,71	0,897	77032	309,5539768	6329608	0,68	6123	301	245337	5942	3	82	JBHHMK000000000

Table S2

Target	Begin	End	GenBank gene	GenBank product	GenBank protein_id
G6N56_RS00100	18208	18819	0	TetR/AcrR family transcriptional regulator	WP_085257702.1
G6N56_RS00105	18951	19226	0	DUF1876 domain-containing protein	WP_085257546.1
G6N56_RS00110	19281	20084	0	HPF/RaiA family ribosome-associated protein	WP_085257545.1
G6N56_RS00115	20205	21326	0	hypothetical protein	WP_085257544.1
G6N56_RS00120	21435	23276	0	long-chain fatty acid--CoA ligase	WP_085257543.1
G6N56_RS00125	23513	24505	0	GNAT family N-acetyltransferase	WP_085257542.1
G6N56_RS00130	24595	25077	0	flavodoxin family protein	WP_085257541.1
G6N56_RS00135	25379	25648	0	hypothetical protein	WP_085257701.1
G6N56_RS00140	25833	27053	0	hypothetical protein	WP_085257540.1
G6N56_RS00145	27068	28444	0	wax ester/triacylglycerol synthase family O-acyltransferase	WP_085257539.1
G6N56_RS00150	28451	29866	0	wax ester/triacylglycerol synthase family O-acyltransferase	WP_085257538.1
G6N56_RS00155	29974	30189	0	hypothetical protein	WP_180150535.1
G6N56_RS00160	30399	31019	0	hypothetical protein	WP_085257536.1
G6N56_RS00165	31072	31500	0	Hsp20/alpha crystallin family protein	WP_085257535.1
G6N56_RS00180	32136	33320	0	site-2 protease family protein	WP_085257534.1
G6N56_RS00185	33317	34108	0	universal stress protein	WP_085257533.1
G6N56_RS00190	34167	34892	0	GAF and ANTAR domain-containing protein	WP_142280766.1
G6N56_RS00195	34912	35793	0	universal stress protein	WP_085257531.1
G6N56_RS00200	35948	36622	0	transcriptional regulator	WP_232069410.1
G6N56_RS00205	36619	37044	0	hypothetical protein	WP_085257529.1
G6N56_RS00210	37061	37408	0	cupin domain-containing protein	WP_085257528.1
G6N56_RS00220	37985	38485	0	DUF1990 family protein	WP_085257526.1
G6N56_RS00225	38509	39384	0	aldo/keto reductase	WP_085257525.1
G6N56_RS00230	39424	40038	0	PaaI family thioesterase	WP_142280764.1
G6N56_RS00235	40103	42211	thrS	threonine--tRNA ligase	WP_085257524.1
G6N56_RS00245	42838	43470	0	CDP-alcohol phosphatidyltransferase family protein	WP_085257522.1
G6N56_RS00250	43467	44417	0	phosphatidylinositol mannoside acyltransferase	WP_232069178.1
G6N56_RS00255	44417	45541	0	glycosyltransferase family 4 protein	WP_085257521.1
G6N56_RS00260	45541	46566	0	NUDIX domain-containing protein	WP_085257520.1
G6N56_RS00265	46701	47606	pdxS	pyridoxal 5'-phosphate synthase lyase subunit PdxS	WP_085257519.1

G6N56_RS00270	47621	48466	tesB		acyl-CoA thioesterase II	WP_085257697.1
G6N56_RS00275	48463	49059	pdxT		pyridoxal 5'-phosphate synthase glutaminase subunit PdxT	WP_085257518.1
G6N56_RS00280	49185	49937		0	YebC/PmpR family DNA-binding transcriptional regulator	WP_085257517.1
G6N56_RS00285	49938	51470		0	polyamine aminopropyltransferase	WP_085257516.1
G6N56_RS00290	51467	51934		0	DUF350 domain-containing protein	WP_085257515.1
G6N56_RS00295	51954	52385		0	DUF4247 domain-containing protein	WP_085257696.1
G6N56_RS00300	52382	52885		0	DUF2617 family protein	WP_085257514.1
G6N56_RS00305	52896	53504		0	DUF4178 domain-containing protein	WP_085257513.1
G6N56_RS00310	53645	54211	ruvC		crossover junction endodeoxyribonuclease RuvC	WP_085257512.1
G6N56_RS00315	54208	54807	ruvA		Holliday junction branch migration protein RuvA	WP_085257511.1
G6N56_RS00320	54804	55853	ruvB		Holliday junction branch migration DNA helicase RuvB	WP_085257510.1
G6N56_RS00325	56030	56413		0	DUF1304 domain-containing protein	WP_085257509.1
G6N56_RS00330	56497	57174		0	hypothetical protein	WP_085257508.1
G6N56_RS00335	57244	60741		0	carboxylic acid reductase	WP_085257507.1
G6N56_RS00340	60897	62237	gabT		4-aminobutyrate--2-oxoglutarate transaminase	WP_085257695.1
G6N56_RS00345	62366	62692	yajC		preprotein translocase subunit YajC	WP_085257506.1
G6N56_RS00350	62781	64589	secD		protein translocase subunit SecD	WP_085257505.1
G6N56_RS00355	64586	65863	secF		protein translocase subunit SecF	WP_085257504.1
G6N56_RS00360	65869	67524		0	ABC transporter substrate-binding protein	WP_085257503.1
G6N56_RS00375	68592	71024		0	RelA/SpoT family protein	WP_180150449.1
G6N56_RS00380	71257	73032		0	protein kinase	WP_163645073.1
G6N56_RS00385	73090	73986		0	peptidylprolyl isomerase	WP_085257720.1
G6N56_RS00390	74100	74774		0	MBL fold metallo-hydrolase	WP_085257721.1
G6N56_RS00395	74771	76042	hisS		histidine--tRNA ligase	WP_085257722.1
G6N56_RS00400	76047	76646		0	TetR/AcrR family transcriptional regulator	WP_085257723.1
G6N56_RS00405	76745	77629		0	alpha/beta fold hydrolase	WP_085257724.1
G6N56_RS00410	77808	78098		0	hypothetical protein	WP_085257725.1
G6N56_RS00415	78140	79162		0	Rv2578c family radical SAM protein	WP_085257726.1
G6N56_RS00420	79163	80731		0	metallophosphoesterase family protein	WP_085257727.1
G6N56_RS00425	80780	81232		0	hypothetical protein	WP_085257728.1
G6N56_RS00430	81253	82731		0	SulP family inorganic anion transporter	WP_085257729.1

G6N56_RS00435	82778	83533		0	ABC transporter ATP-binding protein	WP_085257730.1
G6N56_RS00440	83530	84414		0	ABC transporter permease	WP_085257731.1
G6N56_RS00445	84439	85851		0	ABC transporter substrate-binding protein	WP_085257748.1
G6N56_RS00450	86078	86422		0	DUF202 domain-containing protein	WP_232069179.1
G6N56_RS00455	86422	86739		0	DUF202 domain-containing protein	WP_085257733.1
G6N56_RS00465	86965	87174		0	hypothetical protein	WP_085257734.1
G6N56_RS00470	87203	88558		0	arylsulfatase	WP_085257735.1
G6N56_RS00475	88628	89422		0	GntR family transcriptional regulator	WP_085257736.1
G6N56_RS00480	89549	90424		0	formylglycine-generating enzyme family protein	WP_085257737.1
G6N56_RS00485	90534	91412		0	neutral zinc metallopeptidase	WP_085257738.1
G6N56_RS00490	91422	92309		0	oxidoreductase	WP_180150537.1
G6N56_RS00495	92366	94150	aspS		aspartate--tRNA ligase	WP_142280790.1
G6N56_RS00500	94157	94600		0	nitroreductase family deazaflavin-dependent oxidoreductase	WP_085257740.1
G6N56_RS00505	94606	95640		0	FUSC family protein	WP_085257741.1
G6N56_RS00510	95645	96034		0	hypothetical protein	WP_085257742.1
G6N56_RS00520	97053	98108		0	putative zinc-binding metallopeptidase	WP_085257743.1
G6N56_RS00525	98163	98828		0	hypothetical protein	WP_232069180.1
G6N56_RS00530	98856	101525		0	circularly permuted type 2 ATP-grasp protein	WP_085257745.1
G6N56_RS00535	101525	104845		0	transglutaminase family protein	WP_085257746.1
G6N56_RS00540	104917	105945		0	hypothetical protein	WP_163645074.1
G6N56_RS00545	106036	107412		0	replication-associated recombination protein A	WP_085256971.1
G6N56_RS00550	107550	108173		0	hypothetical protein	WP_085256972.1
G6N56_RS00555	108347	108736		0	secondary thiamine-phosphate synthase enzyme YjbQ	WP_180150538.1
G6N56_RS00560	108833	111532	alaS		alanine--tRNA ligase	WP_085256974.1
G6N56_RS00570	112026	113264		0	endolytic transglycosylase MltG	WP_085256976.1
G6N56_RS00580	114084	114497		0	A24 family peptidase	WP_085256978.1
G6N56_RS00585	114563	115768	aroC		chorismate synthase	WP_085256979.1
G6N56_RS00590	115768	116292		0	shikimate kinase	WP_085256980.1
G6N56_RS00595	116289	117377	aroB		3-dehydroquinate synthase	WP_085256981.1
G6N56_RS00600	117374	117802	aroQ		type II 3-dehydroquinate dehydratase	WP_085256982.1
G6N56_RS00605	117799	118518		0	B-4DMT family transporter	WP_180150451.1

G6N56_RS00610	118553	119656		0	Xaa-Pro peptidase family protein	WP_085256984.1
G6N56_RS00615	119699	120016		0	hypothetical protein	WP_085256985.1
G6N56_RS00620	120122	120685	efp		elongation factor P	WP_085256986.1
G6N56_RS00625	120688	121161	nusB		transcription antitermination factor NusB	WP_085256987.1
G6N56_RS00630	121158	121541		0	antitermination protein NusB	WP_085256988.1
G6N56_RS00635	121626	124481		0	aminotransferase class I/II-fold pyridoxal phosphate-dependen	WP_085256989.1
G6N56_RS00640	124489	124740		0	hypothetical protein	WP_085256990.1
G6N56_RS00645	124802	125335		0	hypothetical protein	WP_232069181.1
G6N56_RS00650	125821	127074		0	MFS transporter	WP_085256991.1
G6N56_RS00655	127190	127936		0	GntR family transcriptional regulator	WP_085256992.1
G6N56_RS00660	127933	128160		0	ferredoxin family protein	WP_085256993.1
G6N56_RS00665	128171	129607		0	ABC transporter substrate-binding protein	WP_085256994.1
G6N56_RS00670	129604	130461		0	ABC transporter permease	WP_085256995.1
G6N56_RS00675	130458	131195		0	ABC transporter ATP-binding protein	WP_085256996.1
G6N56_RS00680	131192	133870		0	fumarate reductase/succinate dehydrogenase flavoprotein sub	WP_180150453.1
G6N56_RS00685	133925	134518		0	TetR/AcrR family transcriptional regulator	WP_085256997.1
G6N56_RS00690	134770	135693		0	alpha/beta hydrolase	WP_085256998.1
G6N56_RS00695	135699	137129		0	amidase	WP_085256999.1
G6N56_RS00705	137784	138773		0	nitronate monooxygenase	WP_264020882.1
G6N56_RS00710	138887	139426		0	GNAT family N-acetyltransferase	WP_085257002.1
G6N56_RS00715	139438	140643		0	serine hydrolase domain-containing protein	WP_169717538.1
G6N56_RS00720	140709	140996		0	hypothetical protein	WP_232069182.1
G6N56_RS00725	141033	142070		0	hypothetical protein	WP_142280718.1
G6N56_RS00730	142110	142505		0	hypothetical protein	WP_085257005.1
G6N56_RS00735	142558	142896		0	hypothetical protein	WP_142280719.1
G6N56_RS00740	143240	143872		0	class I SAM-dependent methyltransferase	WP_142280727.1
G6N56_RS00745	143916	145196		0	HNH endonuclease signature motif containing protein	WP_085257006.1
G6N56_RS00750	145325	145891	pyrR		bifunctional pyr operon transcriptional regulator/uracil phosphо	WP_085257007.1
G6N56_RS00755	145888	146832		0	aspartate carbamoyltransferase catalytic subunit	WP_085257008.1
G6N56_RS00760	146829	148121		0	dihydroorotase	WP_085257009.1
G6N56_RS00765	148118	148627		0	transporter	WP_085257010.1

G6N56_RS00770	148624	149745	carA		glutamine-hydrolyzing carbamoyl-phosphate synthase small su	WP_085257011.1
G6N56_RS00775	149840	153190	carB		carbamoyl-phosphate synthase large subunit	WP_085257012.1
G6N56_RS00780	153187	154011	pyrF		orotidine-5'-phosphate decarboxylase	WP_085257013.1
G6N56_RS00785	154028	154450		0	TOBE domain-containing protein	WP_085257014.1
G6N56_RS00790	154860	155177	mihF		integration host factor, actinobacterial type	WP_067415791.1
G6N56_RS00795	155215	155841	gmk		guanylate kinase	WP_085257015.1
G6N56_RS00800	155937	156263	rpoZ		DNA-directed RNA polymerase subunit omega	WP_085257016.1
G6N56_RS00805	156284	157534	coaBC		bifunctional phosphopantothenoylcysteine decarboxylase/pho	WP_085257017.1
G6N56_RS00810	157651	158862	metK		methionine adenosyltransferase	WP_085257018.1
G6N56_RS00815	158859	159431		0	pyridoxamine 5'-phosphate oxidase family protein	WP_085257019.1
G6N56_RS00820	159448	160926		0	NAD(P)/FAD-dependent oxidoreductase	WP_142280728.1
G6N56_RS00825	160941	161921		0	alpha/beta hydrolase	WP_085257021.1
G6N56_RS00830	161941	162900		0	alpha/beta hydrolase	WP_085257022.1
G6N56_RS00835	162899	163681		0	lysoplasmalogenase	WP_085257023.1
G6N56_RS00840	163757	165700		0	primosomal protein N'	WP_085257094.1
G6N56_RS00845	165796	166287		0	MarR family transcriptional regulator	WP_142280720.1
G6N56_RS00850	166300	167124		0	class I SAM-dependent methyltransferase	WP_085257024.1
G6N56_RS00855	167264	168193	fmt		methionyl-tRNA formyltransferase	WP_085257096.1
G6N56_RS00860	168190	169590		0	transcription antitermination factor NusB	WP_142280721.1
G6N56_RS00865	169606	170304	rpe		ribulose-phosphate 3-epimerase	WP_142280722.1
G6N56_RS00870	170301	171323	ribD		bifunctional diaminohydroxyphosphoribosylaminopyrimidine d	WP_085257026.1
G6N56_RS00875	171344	173029		0	MFS transporter	WP_085257027.1
G6N56_RS00880	173029	173742		0	LppX_LprAFG lipoprotein	WP_085257098.1
G6N56_RS00885	173839	174441		0	riboflavin synthase	WP_085257028.1
G6N56_RS00890	174486	174851		0	hypothetical protein	WP_085257029.1
G6N56_RS00895	175089	176366		0	bifunctional 3,4-dihydroxy-2-butanone-4-phosphate synthase/(	WP_085257030.1
G6N56_RS00900	176363	176845	ribH		6,7-dimethyl-8-ribityllumazine synthase	WP_085257031.1
G6N56_RS00905	176887	177303		0	PH domain-containing protein	WP_085257099.1
G6N56_RS00910	177322	177993		0	hypothetical protein	WP_085257032.1
G6N56_RS00915	178435	180402	uvrC		excinuclease ABC subunit UvrC	WP_085257033.1
G6N56_RS00920	180399	181310	rapZ		RNase adapter RapZ	WP_085257034.1

G6N56_RS00930	182327	183304	whiA		DNA-binding protein WhiA	WP_142280729.1
G6N56_RS00935	183324	184715		0	wax ester/triacylglycerol synthase family O-acyltransferase	WP_085257037.1
G6N56_RS00940	184844	185989		0	Rieske 2Fe-2S domain-containing protein	WP_085257038.1
G6N56_RS00950	187233	188480		0	alpha/beta hydrolase	WP_085257039.1
G6N56_RS00955	188477	190099	fadD12		acyl-CoA ligase FadD12	WP_085257040.1
G6N56_RS00960	190103	190840		0	lysophospholipid acyltransferase family protein	WP_232069413.1
G6N56_RS00965	191077	191520		0	hypothetical protein	WP_085257042.1
G6N56_RS00970	191596	193377		0	DUF3556 domain-containing protein	WP_085257043.1
G6N56_RS00975	193374	194801		0	NAD(P)/FAD-dependent oxidoreductase	WP_085257044.1
G6N56_RS00980	194958	195773		0	Ig-like domain-containing protein	WP_085257045.1
G6N56_RS00985	195869	196417		0	hypothetical protein	WP_085257100.1
G6N56_RS00990	196693	197724	gap		type I glyceraldehyde-3-phosphate dehydrogenase	WP_085257101.1
G6N56_RS00995	197729	198964		0	phosphoglycerate kinase	WP_085257046.1
G6N56_RS01000	198961	199746	tpiA		triose-phosphate isomerase	WP_085257047.1
G6N56_RS01005	199777	200010	secG		preprotein translocase subunit SecG	WP_047313827.1
G6N56_RS01010	200056	202863	ppc		phosphoenolpyruvate carboxylase	WP_085257048.1
G6N56_RS01015	202880	203692		0	DUF1206 domain-containing protein	WP_085257049.1
G6N56_RS01020	203696	203908		0	hypothetical protein	WP_085257050.1
G6N56_RS01025	203945	204343		0	ATPase	WP_085257051.1
G6N56_RS01030	204343	205092	pgl		6-phosphogluconolactonase	WP_085257052.1
G6N56_RS01035	205089	206000	opcA		glucose-6-phosphate dehydrogenase assembly protein OpcA	WP_085257053.1
G6N56_RS01040	206001	207542	zwf		glucose-6-phosphate dehydrogenase	WP_085257054.1
G6N56_RS01045	207539	208660	tal		transaldolase	WP_085257055.1
G6N56_RS01050	208676	210766	tkt		transketolase	WP_085257056.1
G6N56_RS01055	211032	211970		0	heme o synthase	WP_085257057.1
G6N56_RS01060	212259	213776		0	PPE family protein	WP_085257058.1
G6N56_RS01065	213773	214747		0	quinone oxidoreductase	WP_085257102.1
G6N56_RS01075	215367	216311		0	heme A synthase	WP_085257103.1
G6N56_RS01080	216463	217242		0	ABC transporter permease	WP_085257060.1
G6N56_RS01085	217239	218186		0	ABC transporter ATP-binding protein	WP_142280724.1
G6N56_RS01090	218183	219898	mptB		polyprenol phosphomannose-dependent alpha 1,6 mannosyltr	WP_085257061.1

G6N56_RS01095	219990	220754		0	transcriptional regulator	WP_142280725.1
G6N56_RS01100	220751	222202	sufB		Fe-S cluster assembly protein SufB	WP_085257063.1
G6N56_RS01105	222199	223362	sufD		Fe-S cluster assembly protein SufD	WP_085257064.1
G6N56_RS01110	223359	224138	sufC		Fe-S cluster assembly ATPase SufC	WP_085257065.1
G6N56_RS01115	224142	225389		0	cysteine desulfurase	WP_085257066.1
G6N56_RS01120	225386	225904		0	SUF system NifU family Fe-S cluster assembly protein	WP_085257067.1
G6N56_RS01135	226951	227709		0	DUF5666 domain-containing protein	WP_180150455.1
G6N56_RS01140	227779	229608		0	acyl-CoA dehydrogenase	WP_085257071.1
G6N56_RS01145	230053	230412	trxA		thioredoxin	WP_085257072.1
G6N56_RS01155	231269	232897		0	ABC-F family ATP-binding cassette domain-containing protein	WP_085257074.1
G6N56_RS01160	232989	233180		0	helix-turn-helix domain-containing protein	WP_085257075.1
G6N56_RS01165	233210	233815		0	TetR/AcrR family transcriptional regulator	WP_085257076.1
G6N56_RS01170	233826	236651		0	aconitate hydratase	WP_142280726.1
G6N56_RS01175	237018	237566		0	hypothetical protein	WP_085257077.1
G6N56_RS01180	237806	239254	ripA		NlpC/P60 family peptidoglycan endopeptidase RipA	WP_085257078.1
G6N56_RS01185	239280	240014	ripB		NlpC/P60 family peptidoglycan endopeptidase RipB	WP_085257079.1
G6N56_RS01190	240181	241314		0	MoxR family ATPase	WP_085257080.1
G6N56_RS01195	241311	242261		0	DUF58 domain-containing protein	WP_085257081.1
G6N56_RS01200	242258	243265		0	VWA domain-containing protein	WP_085257082.1
G6N56_RS01205	243338	244180		0	type IV toxin-antitoxin system AbiEi family antitoxin	WP_085257083.1
G6N56_RS01210	244328	245107	fabG1		3-oxoacyl-ACP reductase FabG1	WP_085257084.1
G6N56_RS01215	245159	245968	inhA		NADH-dependent enoyl-ACP reductase InhA	WP_085257085.1
G6N56_RS01220	245976	246995		0	ferrochelatase	WP_085257086.1
G6N56_RS01225	246999	247910		0	hypothetical protein	WP_085257087.1
G6N56_RS01230	247970	248407		0	NfeD family protein	WP_085257088.1
G6N56_RS01235	248444	249586		0	SPFH domain-containing protein	WP_085257089.1
G6N56_RS01240	249605	249961		0	DoxX family protein	WP_085257090.1
G6N56_RS01250	251958	252335		0	GntR family transcriptional regulator	WP_264020924.1
G6N56_RS01255	252552	253091		0	nicotinamide phosphoribosyltransferase domain-containing pr	WP_142280713.1
G6N56_RS01260	253100	253870		0	orotidine 5'-phosphate decarboxylase	WP_085256950.1
G6N56_RS01265	253867	254379		0	CinA family protein	WP_085256946.1

G6N56_RS01270	255457	256944		0	nicotinate phosphoribosyltransferase	WP_085256945.1
G6N56_RS01275	257050	257820		0	orotidine 5'-phosphate decarboxylase	WP_085256944.1
G6N56_RS01280	257817	258329		0	nicotinamide-nucleotide amidohydrolase family protein	WP_085256943.1
G6N56_RS01285	258350	258952		0	HAD-IB family phosphatase	WP_232069183.1
G6N56_RS01290	259452	260816		0	MFS transporter	WP_085256941.1
G6N56_RS01295	260859	261113		0	hypothetical protein	WP_085256940.1
G6N56_RS01300	261234	261923		0	response regulator transcription factor	WP_085256939.1
G6N56_RS01305	261920	263401		0	HAMP domain-containing sensor histidine kinase	WP_085256938.1
G6N56_RS01310	263398	263652		0	hypothetical protein	WP_085256937.1
G6N56_RS01315	263680	264057		0	hypothetical protein	WP_180150457.1
G6N56_RS01320	264277	265050		0	TVP38/TMEM64 family protein	WP_085256936.1
G6N56_RS01325	265231	267054	mutA		methylmalonyl-CoA mutase small subunit	WP_085256935.1
G6N56_RS01330	267060	269312	scpA		methylmalonyl-CoA mutase	WP_085256948.1
G6N56_RS01335	269387	270376	meaB		methylmalonyl Co-A mutase-associated GTPase MeaB	WP_085256934.1
G6N56_RS01340	270440	271726		0	serine hydrolase domain-containing protein	WP_085256933.1
G6N56_RS01345	271723	272709		0	sulfotransferase family protein	WP_142280711.1
G6N56_RS01355	275566	275961		0	hypothetical protein	WP_085258168.1
G6N56_RS01360	275971	276465		0	GtrA family protein	WP_085258169.1
G6N56_RS01365	276993	277679		0	class I SAM-dependent methyltransferase	WP_085253678.1
G6N56_RS01370	277855	279114		0	nucleotide sugar dehydrogenase	WP_085253679.1
G6N56_RS01375	279111	280130		0	NAD(P)-dependent oxidoreductase	WP_085253680.1
G6N56_RS01380	280160	281638		0	glycosyltransferase	WP_085253681.1
G6N56_RS01385	281836	283461		0	hypothetical protein	WP_211287369.1
G6N56_RS01390	283731	285398		0	hypothetical protein	WP_232069185.1
G6N56_RS01395	285492	286523		0	glycosyltransferase	WP_085253683.1
G6N56_RS01400	287297	288151		0	class I SAM-dependent methyltransferase	WP_232069186.1
G6N56_RS01405	288235	288639		0	hypothetical protein	WP_085253684.1
G6N56_RS01410	289288	290079		0	methyltransferase domain-containing protein	WP_158090657.1
G6N56_RS01415	290106	291092		0	rhamnan synthesis F family protein	WP_085253686.1
G6N56_RS01425	291958	292749		0	ABC transporter permease	WP_085253687.1
G6N56_RS01430	292746	293489		0	ABC transporter permease	WP_085253688.1

G6N56_RS01435	293486	294430		0	ATP-binding cassette domain-containing protein	WP_085253689.1
G6N56_RS01440	294811	296112		0	AMP-binding protein	WP_264020568.1
G6N56_RS01445	296216	299722		0	RND family transporter	WP_085253691.1
G6N56_RS01450	299843	301117		0	glycosyltransferase	WP_085253812.1
G6N56_RS01455	301173	302615		0	condensation domain-containing protein	WP_085253692.1
G6N56_RS01460	302674	308946	pks2		type I polyketide synthase	WP_085253693.1
G6N56_RS01465	309852	310424		0	carboxymuconolactone decarboxylase family protein	WP_085253694.1
G6N56_RS01470	310539	311663		0	nitronate monooxygenase family protein	WP_085253695.1
G6N56_RS01475	311660	312304		0	TetR family transcriptional regulator	WP_085253696.1
G6N56_RS01480	313094	313324		0	Rv1535 family protein	WP_085253697.1
G6N56_RS01485	313653	316790	ileS		isoleucine--tRNA ligase	WP_085253813.1
G6N56_RS01490	316798	317625		0	class I SAM-dependent methyltransferase	WP_085253698.1
G6N56_RS01495	317801	319186		0	DNA polymerase IV	WP_163645190.1
G6N56_RS01505	320161	320748	lspA		signal peptidase II	WP_085253701.1
G6N56_RS01510	320745	321671		0	RluA family pseudouridine synthase	WP_085253702.1
G6N56_RS01515	321672	322430		0	class I SAM-dependent methyltransferase	WP_085253703.1
G6N56_RS01520	322427	323068		0	MliC family protein	WP_085253704.1
G6N56_RS01525	323313	323717		0	group 1 truncated hemoglobin	WP_085253705.1
G6N56_RS01530	323999	325051		0	SDR family NAD(P)-dependent oxidoreductase	WP_085253706.1
G6N56_RS01535	325074	325877		0	SDR family oxidoreductase	WP_085253707.1
G6N56_RS01540	326029	326460		0	SRPBCC family protein	WP_085253708.1
G6N56_RS01545	326466	329366		0	MMPL family transporter	WP_085253709.1
G6N56_RS01550	329363	329809		0	MmpS family protein	WP_264020565.1
G6N56_RS01555	330084	330680		0	TetR/AcrR family transcriptional regulator	WP_085253814.1
G6N56_RS01560	330782	334315	dnaE		DNA polymerase III subunit alpha	WP_085253711.1
G6N56_RS01565	334604	335053		0	hypothetical protein	WP_142280399.1
G6N56_RS01570	335112	335573		0	lipoprotein LpqH	WP_085253713.1
G6N56_RS01575	335783	336046		0	hypothetical protein	WP_163645075.1
G6N56_RS01585	336753	337736		0	MBL fold metallo-hydrolase	WP_085253715.1
G6N56_RS01590	337736	338179		0	PPOX class F420-dependent oxidoreductase	WP_085253716.1
G6N56_RS01595	338240	338623		0	MerR family transcriptional regulator	WP_085253717.1

G6N56_RS01600	338699	340525	fadD11		fatty acid--CoA ligase FadD11	WP_085253718.1
G6N56_RS01605	340522	342414		0	lysophospholipid acyltransferase	WP_085253719.1
G6N56_RS01610	342931	343962		0	helix-turn-helix domain-containing protein	WP_085253720.1
G6N56_RS01615	344027	344851		0	TetR family transcriptional regulator	WP_085253721.1
G6N56_RS01620	345031	345864		0	mycofactocin-coupled SDR family oxidoreductase	WP_085253722.1
G6N56_RS01625	345999	347429		0	FAD-dependent monooxygenase	WP_085253723.1
G6N56_RS01630	347426	347833		0	hypothetical protein	WP_085253724.1
G6N56_RS01635	347931	348488		0	TetR/AcrR family transcriptional regulator	WP_085253725.1
G6N56_RS01645	348999	350288	ilvA		threonine ammonia-lyase	WP_085253726.1
G6N56_RS01650	350364	351011		0	pyridoxamine 5'-phosphate oxidase family protein	WP_085253727.1
G6N56_RS01655	351052	352365		0	aminotransferase class I/II-fold pyridoxal phosphate-dependen	WP_085253728.1
G6N56_RS28375	353139	353294		0	DUF6131 family protein	WP_169717507.1
G6N56_RS01665	353389	354048		0	response regulator transcription factor	WP_085253730.1
G6N56_RS01670	354045	354959		0	class I SAM-dependent methyltransferase	WP_085253731.1
G6N56_RS01680	356780	359104	treY		malto-oligosyltrehalose synthase	WP_085253733.1
G6N56_RS01685	359108	361300	glgX		glycogen debranching protein GlgX	WP_085253734.1
G6N56_RS01690	361340	363472		0	acyltransferase family protein	WP_085253735.1
G6N56_RS01695	363567	364349	ripD		NlpC/P60 family peptidoglycan-binding protein RipD	WP_085253736.1
G6N56_RS01700	364655	365059		0	hypothetical protein	WP_232069416.1
G6N56_RS01705	365178	366482		0	adenosylmethionine--8-amino-7-oxononanoate transaminase	WP_085253738.1
G6N56_RS01710	366489	367634		0	8-amino-7-oxononanoate synthase	WP_085253739.1
G6N56_RS01715	367631	368311	bioD		dethiobiotin synthase	WP_085253740.1
G6N56_RS01720	368311	368826		0	2'-5' RNA ligase family protein	WP_085253741.1
G6N56_RS01725	368906	370261		0	hypothetical protein	WP_085253742.1
G6N56_RS01730	370258	370626		0	hypothetical protein	WP_085253743.1
G6N56_RS01735	370630	372462		0	hypothetical protein	WP_085253744.1
G6N56_RS01740	372550	372795		0	hypothetical protein	WP_085253745.1
G6N56_RS01745	372927	373961	bioB		biotin synthase BioB	WP_085253816.1
G6N56_RS01750	373966	374202		0	hypothetical protein	WP_085253746.1
G6N56_RS01755	374199	374825		0	DUF2567 domain-containing protein	WP_085253747.1
G6N56_RS01760	374831	376171		0	lipase family protein	WP_085253748.1

G6N56_RS01765	376309	377019		0	NUDIX domain-containing protein	WP_085253749.1
G6N56_RS01770	377084	378115	nadA		quinolinate synthase NadA	WP_180150540.1
G6N56_RS01775	378115	379689		0	L-aspartate oxidase	WP_142280405.1
G6N56_RS01780	379686	380540	nadC		carboxylating nicotinate-nucleotide diphosphorylase	WP_085253752.1
G6N56_RS01785	380673	381095		0	nitroreductase family deazaflavin-dependent oxidoreductase	WP_085253753.1
G6N56_RS01790	381177	382523	hisD		histidinol dehydrogenase	WP_085253754.1
G6N56_RS01795	382520	383656		0	histidinol-phosphate transaminase	WP_085253755.1
G6N56_RS01800	383653	384285	hisB		imidazoleglycerol-phosphate dehydratase HisB	WP_085253756.1
G6N56_RS01805	384291	384914	hisH		imidazole glycerol phosphate synthase subunit HisH	WP_085253757.1
G6N56_RS01810	384914	385660	priA		bifunctional 1-(5-phosphoribosyl)-5-((5- phosphoribosylamino)	WP_085253758.1
G6N56_RS01815	385669	386469		0	inositol monophosphatase	WP_085253759.1
G6N56_RS01820	386472	387257	hisF		imidazole glycerol phosphate synthase subunit HisF	WP_085253760.1
G6N56_RS01825	387254	387601	hisI		phosphoribosyl-AMP cyclohydrolase	WP_085253761.1
G6N56_RS01835	387987	389078		0	calcium:proton antiporter	WP_085253762.1
G6N56_RS01845	389592	391130		0	anthranilate synthase component I	WP_085253763.1
G6N56_RS01855	391888	392706	trpC		indole-3-glycerol phosphate synthase TrpC	WP_085253765.1
G6N56_RS01860	392766	394049	trpB		tryptophan synthase subunit beta	WP_085253766.1
G6N56_RS01865	394046	394861	trpA		tryptophan synthase subunit alpha	WP_085253767.1
G6N56_RS01870	394858	395976		0	prolipoprotein diacylglyceryl transferase	WP_085253768.1
G6N56_RS01875	396032	396427		0	TM2 domain-containing protein	WP_085253769.1
G6N56_RS01880	396435	396839		0	DUF2752 domain-containing protein	WP_085253770.1
G6N56_RS01885	396905	398323	pyk		pyruvate kinase	WP_085253771.1
G6N56_RS01890	398401	399306		0	acyl-CoA thioesterase II	WP_085253772.1
G6N56_RS01895	399533	400930		0	phosphatidylglycerol lysyltransferase domain-containing protei	WP_085253773.1
G6N56_RS01905	401938	403602	cydC		thiol reductant ABC exporter subunit CydC	WP_085253774.1
G6N56_RS01915	405265	406305	cydB		cytochrome d ubiquinol oxidase subunit II	WP_085253776.1
G6N56_RS01920	406335	407807		0	cytochrome ubiquinol oxidase subunit I	WP_085253777.1
G6N56_RS01925	407894	408487		0	HdeD family acid-resistance protein	WP_142280401.1
G6N56_RS01930	408518	409846		0	adenylate/guanylate cyclase domain-containing protein	WP_085253818.1
G6N56_RS01940	410736	411353		0	ANTAR domain-containing response regulator	WP_085253779.1
G6N56_RS01945	411442	412641		0	lipid-transfer protein	WP_085253780.1

G6N56_RS01950	412638	413081		0	Zn-ribbon domain-containing OB-fold protein	WP_085253781.1
G6N56_RS01955	413246	415909	polA		DNA polymerase I	WP_142280406.1
G6N56_RS28700	416011	416238		0	DMT family transporter	WP_232069418.1
G6N56_RS01965	416287	417735	rpsA		30S ribosomal protein S1	WP_085253783.1
G6N56_RS01970	417748	418962	coaE		dephospho-CoA kinase	WP_085253784.1
G6N56_RS28705	419105	419719		0	chitin-binding protein	WP_085253785.1
G6N56_RS01975	420018	420908	ligD		non-homologous end-joining DNA ligase	WP_085253786.1
G6N56_RS01980	420908	421906	ligD		non-homologous end-joining DNA ligase	WP_085253787.1
G6N56_RS01985	421997	423640		0	HAD hydrolase family protein	WP_232069188.1
G6N56_RS01990	423680	424114		0	CBS domain-containing protein	WP_085253789.1
G6N56_RS01995	424292	424738		0	DUF402 domain-containing protein	WP_085253790.1
G6N56_RS02000	424815	427574		0	ATP-binding cassette domain-containing protein	WP_232069189.1
G6N56_RS02005	427863	429929		0	serine/threonine-protein kinase	WP_085253792.1
G6N56_RS02010	430105	432264	uvrB		excinuclease ABC subunit UvrB	WP_085253793.1
G6N56_RS02015	432261	433700		0	MFS transporter	WP_180150460.1
G6N56_RS02025	434434	435999		0	glycosyltransferase family 39 protein	WP_085253795.1
G6N56_RS02030	436309	436752		0	universal stress protein	WP_085253796.1
G6N56_RS02035	436758	437456		0	MBL fold metallo-hydrolase	WP_085253797.1
G6N56_RS02040	437571	440486	uvrA		excinuclease ABC subunit UvrA	WP_085253798.1
G6N56_RS02045	440622	440855		0	hypothetical protein	WP_085253799.1
G6N56_RS02050	441005	442423		0	alpha/beta hydrolase-fold protein	WP_085253820.1
G6N56_RS02055	442551	445868	lysX		bifunctional lysylphosphatidylglycerol synthetase/lysine--tRNA	WP_264019393.1
G6N56_RS02060	446303	446911	infC		translation initiation factor IF-3	WP_085253800.1
G6N56_RS02065	446929	447123	rpmI		50S ribosomal protein L35	WP_085253801.1
G6N56_RS02070	447203	447598	rplT		50S ribosomal protein L20	WP_085253802.1
G6N56_RS02075	447611	448390		0	RNA methyltransferase	WP_085253803.1
G6N56_RS02080	448414	449481		0	oxygenase MpaB family protein	WP_085253804.1
G6N56_RS02085	449683	450612		0	adenylate/guanylate cyclase domain-containing protein	WP_085253805.1
G6N56_RS02090	450622	451440		0	hypothetical protein	WP_085253822.1
G6N56_RS02095	451560	452603	pheS		phenylalanine--tRNA ligase subunit alpha	WP_085253806.1
G6N56_RS02100	452603	455089	pheT		phenylalanine--tRNA ligase subunit beta	WP_085253807.1

G6N56_RS29370	455475	457142		0	PE family protein	WP_085253808.1
G6N56_RS02110	457418	458974		0	PE family protein	WP_085253809.1
G6N56_RS02115	459166	460212		0	PE family protein	WP_085253823.1
G6N56_RS29375	460396	462030		0	PE family protein	WP_085253810.1
G6N56_RS02125	462398	467215		0	PE family protein	WP_163645076.1
G6N56_RS02130	467375	468418	argC		N-acetyl-gamma-glutamyl-phosphate reductase	WP_085256893.1
G6N56_RS02135	468415	469629	argJ		bifunctional glutamate N-acetyltransferase/amino-acid acetylt	WP_085256892.1
G6N56_RS02140	469626	470513	argB		acetylglutamate kinase	WP_085256891.1
G6N56_RS02145	470552	471697		0	acetylornithine transaminase	WP_232069420.1
G6N56_RS02155	472613	473107		0	arginine repressor	WP_085256888.1
G6N56_RS02160	473123	474319		0	argininosuccinate synthase	WP_085256887.1
G6N56_RS02165	474423	475832	argH		argininosuccinate lyase	WP_085256886.1
G6N56_RS02170	475935	476996		0	type III polyketide synthase	WP_085256885.1
G6N56_RS02175	477022	483372		0	type I polyketide synthase	WP_085256884.1
G6N56_RS02195	490806	493877		0	type I polyketide synthase	WP_085256882.1
G6N56_RS02200	494062	495123		0	type III polyketide synthase	WP_085256881.1
G6N56_RS02205	495356	497125		0	ABC-F family ATP-binding cassette domain-containing protein	WP_085256880.1
G6N56_RS02210	497228	497569		0	hypothetical protein	WP_142280701.1
G6N56_RS02215	498086	501043		0	acyl-CoA synthetase	WP_232069421.1
G6N56_RS02225	501217	501840		0	TetR family transcriptional regulator	WP_085256877.1
G6N56_RS02230	501852	502694		0	ABC transporter permease	WP_197746651.1
G6N56_RS02240	503555	504829		0	ammonium transporter	WP_085256875.1
G6N56_RS02245	504871	505482		0	DNA-3-methyladenine glycosylase	WP_085256874.1
G6N56_RS02250	505574	506845	tyrS		tyrosine--tRNA ligase	WP_142280710.1
G6N56_RS02255	506932	507261		0	DUF732 domain-containing protein	WP_085256930.1
G6N56_RS02260	507328	507738		0	PaaI family thioesterase	WP_085256929.1
G6N56_RS02265	507793	508743		0	LLM class flavin-dependent oxidoreductase	WP_232069190.1
G6N56_RS02270	508828	509640		0	helix-turn-helix transcriptional regulator	WP_085256872.1
G6N56_RS02275	509766	511235		0	NAD(P)/FAD-dependent oxidoreductase	WP_232069191.1
G6N56_RS02280	511268	512587		0	NADH:flavin oxidoreductase/NADH oxidase family protein	WP_085256870.1
G6N56_RS02285	512676	513257		0	TetR/AcrR family transcriptional regulator	WP_085256869.1

G6N56_RS02290	513423	514220		0	tetratricopeptide repeat protein	WP_142280700.1
G6N56_RS02295	514223	515233		0	HAD-IIA family hydrolase	WP_085256868.1
G6N56_RS02300	515257	515433		0	hypothetical protein	WP_142280699.1
G6N56_RS02305	515442	516251		0	TlyA family RNA methyltransferase	WP_085256867.1
G6N56_RS02310	516248	517165		0	NAD kinase	WP_142280698.1
G6N56_RS02315	517179	518930	recN		DNA repair protein RecN	WP_085256866.1
G6N56_RS02320	519133	520314	steA		putative cytokinetic ring protein SteA	WP_085256865.1
G6N56_RS02325	520343	521287		0	copper transporter	WP_085256864.1
G6N56_RS02330	521451	523196		0	CTP synthase	WP_085256863.1
G6N56_RS02340	523809	524750	xerD		site-specific tyrosine recombinase XerD	WP_085256925.1
G6N56_RS02345	524856	525608		0	class I SAM-dependent methyltransferase	WP_085256861.1
G6N56_RS02350	525815	526681		0	ParA family protein	WP_085256860.1
G6N56_RS02355	526678	527505		0	segregation/condensation protein A	WP_085256859.1
G6N56_RS02360	527506	528207	scpB		SMC-Scp complex subunit ScpB	WP_085256858.1
G6N56_RS02365	528207	528950		0	pseudouridine synthase	WP_085256857.1
G6N56_RS02375	529627	531027	der		ribosome biogenesis GTPase Der	WP_085256855.1
G6N56_RS02380	531265	532062		0	SDR family oxidoreductase	WP_085256854.1
G6N56_RS02385	532074	532904		0	cyclase family protein	WP_085256853.1
G6N56_RS02390	532904	533671		0	IclR family transcriptional regulator	WP_085256852.1
G6N56_RS02400	534196	535386		0	AI-2E family transporter	WP_232069192.1
G6N56_RS02405	535457	536332		0	patatin-like phospholipase family protein	WP_232069193.1
G6N56_RS02410	536520	537224		0	alpha/beta hydrolase	WP_232069422.1
G6N56_RS02415	539248	539673		0	cellulose-binding domain-containing protein	WP_085256850.1
G6N56_RS02420	539796	540095		0	hypothetical protein	WP_085256922.1
G6N56_RS02425	540411	541667		0	DUF4010 domain-containing protein	WP_085256849.1
G6N56_RS02430	541992	542306		0	DUF732 domain-containing protein	WP_085256848.1
G6N56_RS02435	542518	542871		0	DUF732 domain-containing protein	WP_085256847.1
G6N56_RS02440	543285	543581		0	hypothetical protein	WP_085256846.1
G6N56_RS02445	543629	546388		0	LuxR family transcriptional regulator	WP_085256845.1
G6N56_RS28725	547247	547558		0	DUF3349 domain-containing protein	WP_232069423.1
G6N56_RS02455	547851	549629		0	NAD-binding protein	WP_085256843.1

G6N56_RS02460	550154	551590	glnA		type I glutamate--ammonia ligase	WP_085256842.1
G6N56_RS02465	551747	552181		0	DUF4383 domain-containing protein	WP_085256841.1
G6N56_RS02470	552257	553813		0	succinic semialdehyde dehydrogenase	WP_085256840.1
G6N56_RS02475	553912	554427		0	DUF5994 family protein	WP_232069194.1
G6N56_RS02480	554981	555214		0	hypothetical protein	WP_085256839.1
G6N56_RS02485	555283	556008		0	glycoside hydrolase	WP_085256838.1
G6N56_RS02490	556145	556618		0	VOC family protein	WP_085256837.1
G6N56_RS02495	556651	557073		0	DUF5313 domain-containing protein	WP_085256836.1
G6N56_RS02505	558024	558995		0	hypothetical protein	WP_142280695.1
G6N56_RS02510	559011	559391		0	DUF732 domain-containing protein	WP_085256833.1
G6N56_RS02515	559650	561044		0	OFA family MFS transporter	WP_085256832.1
G6N56_RS28385	561041	561178		0	hypothetical protein	WP_169717535.1
G6N56_RS02520	561269	563074		0	N-acetylglutaminylglutamine amidotransferase	WP_085256831.1
G6N56_RS02525	563071	564855	ngg		N-acetylglutaminylglutamine synthetase	WP_085256830.1
G6N56_RS02530	565043	566413		0	amidase family protein	WP_197746652.1
G6N56_RS02535	566676	568196		0	MFS transporter	WP_085256829.1
G6N56_RS02540	568333	570510		0	glutamine synthetase III	WP_085256828.1
G6N56_RS02550	570843	572648		0	serine/threonine protein kinase PknE	WP_085256826.1
G6N56_RS02555	572855	573154		0	hypothetical protein	WP_085256920.1
G6N56_RS02560	573351	574061		0	pyrimidine reductase family protein	WP_085256825.1
G6N56_RS02565	574065	574562		0	flavin reductase family protein	WP_085256824.1
G6N56_RS02570	574623	576791		0	xanthine dehydrogenase family protein molybdopterin-binding s	WP_085256823.1
G6N56_RS02575	576788	577765		0	xanthine dehydrogenase family protein subunit M	WP_085256822.1
G6N56_RS02580	577769	578371		0	(2Fe-2S)-binding protein	WP_180150462.1
G6N56_RS02585	578445	579701		0	amino acid permease	WP_085256918.1
G6N56_RS02590	579806	580555		0	ArsR family transcriptional regulator	WP_085256821.1
G6N56_RS02595	580558	582036		0	multicopper oxidase domain-containing protein	WP_232069195.1
G6N56_RS02600	582094	583083		0	LLM class F420-dependent oxidoreductase	WP_085256819.1
G6N56_RS02605	583195	583923		0	DUF4190 domain-containing protein	WP_085256818.1
G6N56_RS02610	583965	585125		0	FAD-dependent oxidoreductase	WP_085256917.1
G6N56_RS02615	585222	586079		0	hypothetical protein	WP_180150464.1

G6N56_RS02620	586099	587790		0	3-ketosteroid-delta-1-dehydrogenase	WP_264019377.1
G6N56_RS02625	587892	589031		0	Rieske 2Fe-2S domain-containing protein	WP_085256816.1
G6N56_RS02630	589031	589318		0	ferredoxin	WP_085256815.1
G6N56_RS02635	589378	593832		0	cation-translocating P-type ATPase	WP_170294985.1
G6N56_RS02640	594472	595116		0	cutinase family protein	WP_232069426.1
G6N56_RS02645	595353	597710		0	FdhF/YdeP family oxidoreductase	WP_085256813.1
G6N56_RS02650	597718	599142		0	cytochrome ubiquinol oxidase subunit I	WP_232069196.1
G6N56_RS02655	599142	600149		0	cytochrome d ubiquinol oxidase subunit II	WP_085256812.1
G6N56_RS02660	600156	600965		0	universal stress protein	WP_142280693.1
G6N56_RS02665	601030	601857	fdhD		formate dehydrogenase accessory sulfurtransferase FdhD	WP_085256810.1
G6N56_RS02670	601896	604037		0	FUSC family protein	WP_232069197.1
G6N56_RS02675	604125	605033		0	VOC family protein	WP_085256808.1
G6N56_RS02680	605045	606229		0	acyl-CoA dehydrogenase family protein	WP_085256807.1
G6N56_RS02685	606518	607381		0	alpha/beta fold hydrolase	WP_142280692.1
G6N56_RS02690	607495	608880		0	wax ester/triacylglycerol synthase family O-acyltransferase	WP_085256806.1
G6N56_RS02695	609064	609513		0	DoxX family protein	WP_085256805.1
G6N56_RS02700	609648	610466		0	transglutaminase family protein	WP_085256804.1
G6N56_RS02705	610564	610800		0	CsbD family protein	WP_085256803.1
G6N56_RS02720	611478	611858		0	hypothetical protein	WP_142280691.1
G6N56_RS02725	611953	613059		0	3-oxoacyl-[acyl-carrier-protein] synthase III C-terminal domain-	WP_085256801.1
G6N56_RS02730	613069	613572		0	isoprenylcysteine carboxyl methyltransferase family protein	WP_085256800.1
G6N56_RS02735	613577	614419		0	SDR family oxidoreductase	WP_085256799.1
G6N56_RS02740	614571	615599		0	alpha/beta hydrolase	WP_085256798.1
G6N56_RS02745	615765	618434		0	ATP-binding cassette domain-containing protein	WP_163645194.1
G6N56_RS28390	618441	618605		0	hypothetical protein	WP_169717534.1
G6N56_RS02750	618806	619504		0	tetratricopeptide repeat protein	WP_085256796.1
G6N56_RS02760	619808	620773		0	alpha/beta hydrolase	WP_232069198.1
G6N56_RS02765	620770	622371		0	NAD(P)/FAD-dependent oxidoreductase	WP_085256793.1
G6N56_RS02775	622753	623598		0	DMT family transporter	WP_085256792.1
G6N56_RS02780	623832	624929		0	ferredoxin reductase	WP_085256791.1
G6N56_RS02785	624946	626355		0	acyl-CoA desaturase	WP_085256790.1

G6N56_RS02795	626755	627069	0	hypothetical protein	WP_085256789.1
G6N56_RS02800	627260	627568	0	hypothetical protein	WP_142280690.1
G6N56_RS02805	627659	629275	0	class I adenylate-forming enzyme family protein	WP_085256788.1
G6N56_RS02815	630087	631355	0	amidohydrolase family protein	WP_085256786.1
G6N56_RS02820	631359	632177	0	class I SAM-dependent methyltransferase	WP_085256785.1
G6N56_RS02825	632186	633283	0	FHA domain-containing protein	WP_085256784.1
G6N56_RS02830	633701	633925	0	hypothetical protein	WP_085256783.1
G6N56_RS02835	633948	634976	0	nitronate monooxygenase	WP_085256782.1
G6N56_RS02840	635083	636711	0	arylsulfatase	WP_085256912.1
G6N56_RS02845	637001	638626	0	hypothetical protein	WP_142280705.1
G6N56_RS02850	638658	639893	0	FAD-dependent oxidoreductase	WP_085256780.1
G6N56_RS02855	639912	640901	0	Gfo/Idh/MocA family oxidoreductase	WP_085256779.1
G6N56_RS02865	641846	642505	0	TetR family transcriptional regulator C-terminal domain-contair	WP_085256777.1
G6N56_RS02870	642782	643987	0	cytochrome P450	WP_085256776.1
G6N56_RS02875	643984	644820	0	mycofactocin-coupled SDR family oxidoreductase	WP_085256775.1
G6N56_RS02880	644856	645827	0	enoyl-CoA hydratase/isomerase family protein	WP_085256911.1
G6N56_RS02885	645835	648201	0	CoA transferase	WP_085256774.1
G6N56_RS02890	648198	648962	0	SDR family NAD(P)-dependent oxidoreductase	WP_085256773.1
G6N56_RS02900	649956	651329	0	HNH endonuclease signature motif containing protein	WP_085256772.1
G6N56_RS02910	652745	654007	0	Xaa-Pro peptidase family protein	WP_085256910.1
G6N56_RS02920	654652	655530	0	DUF4344 domain-containing metallopeptidase	WP_085256769.1
G6N56_RS02925	655654	656124	0	hypothetical protein	WP_085256768.1
G6N56_RS02930	656282	656773	0	hypothetical protein	WP_085256767.1
G6N56_RS02935	656831	657463	0	hypothetical protein	WP_085256766.1
G6N56_RS02940	657569	658378	0	SigB/SigF/SigG family RNA polymerase sigma factor	WP_085256765.1
G6N56_RS02945	658984	659874	0	SDR family oxidoreductase	WP_085256764.1
G6N56_RS02950	659943	660728	0	heavy metal-binding domain-containing protein	WP_085256763.1
G6N56_RS02955	660728	661105	0	DUF5073 family protein	WP_085256762.1
G6N56_RS02960	661140	661769	0	cutinase family protein	WP_085256761.1
G6N56_RS02965	661832	662263	0	hypothetical protein	WP_180150543.1
G6N56_RS02970	662415	663086	0	TetR/AcrR family transcriptional regulator	WP_085256909.1

G6N56_RS02980	664198	664833	0	TetR/AcrR family transcriptional regulator	WP_085256759.1
G6N56_RS02985	664846	665397	0	class I SAM-dependent methyltransferase	WP_085256758.1
G6N56_RS02990	665550	666386	0	enoyl-CoA hydratase-related protein	WP_085256757.1
G6N56_RS02995	666472	668172	0	FAD-binding protein	WP_085256756.1
G6N56_RS03000	668169	669563	0	aromatic ring-hydroxylating dioxygenase subunit alpha	WP_085256755.1
G6N56_RS03005	669579	671105	0	acyl-CoA synthetase	WP_085256754.1
G6N56_RS03010	671116	671988	0	enoyl-CoA hydratase-related protein	WP_085256753.1
G6N56_RS03020	672234	673244	0	transketolase C-terminal domain-containing protein	WP_085256751.1
G6N56_RS03025	673247	674194	0	thiamine pyrophosphate-dependent dehydrogenase E1 compo	WP_232069427.1
G6N56_RS03030	674254	675117	0	mycofactocin-coupled SDR family oxidoreductase	WP_085256749.1
G6N56_RS03035	675384	676628	0	CaiB/BaiF CoA-transferase family protein	WP_085256748.1
G6N56_RS03040	676743	677012	0	metal-sensitive transcriptional regulator	WP_085256908.1
G6N56_RS03045	677050	677472	0	DUF302 domain-containing protein	WP_085256747.1
G6N56_RS03050	677490	677855	0	carboxymuconolactone decarboxylase family protein	WP_085256746.1
G6N56_RS03055	677980	679344	0	wax ester/triacylglycerol synthase family O-acyltransferase	WP_085256907.1
G6N56_RS03060	679648	679938	0	ANTAR domain-containing protein	WP_085256745.1
G6N56_RS03065	680004	680630	0	MarR family winged helix-turn-helix transcriptional regulator	WP_232069199.1
G6N56_RS03070	680824	682311	0	aromatic/alkene/methane monooxygenase hydroxylase/oxyger	WP_085256744.1
G6N56_RS03080	682564	682929	0	Rieske 2Fe-2S domain-containing protein	WP_085256742.1
G6N56_RS03090	683226	684233	0	aromatic/alkene monooxygenase hydroxylase subunit beta	WP_085256740.1
G6N56_RS03095	684240	684989	0	IclR family transcriptional regulator	WP_142280689.1
G6N56_RS03100	685051	686391	0	FAD-binding oxidoreductase	WP_085256904.1
G6N56_RS03105	686388	687182	0	hypothetical protein	WP_085256905.1
G6N56_RS03110	687209	687658	0	hypothetical protein	WP_085256738.1
G6N56_RS03115	687826	688167	0	DUF732 domain-containing protein	WP_085256737.1
G6N56_RS03120	688306	690090	0	DUF4407 domain-containing protein	WP_085256736.1
G6N56_RS03125	690320	690877	0	hypothetical protein	WP_085256735.1
G6N56_RS03130	690989	691417	0	SRPBCC family protein	WP_180150468.1
G6N56_RS03135	691442	691663	0	LapA family protein	WP_142280703.1
G6N56_RS03140	691835	692305	0	nuclear transport factor 2 family protein	WP_085256732.1
G6N56_RS03145	692316	693197	0	LLM class F420-dependent oxidoreductase	WP_085256731.1

G6N56_RS03150	693254	695395	malQ	4-alpha-glucanotransferase	WP_085256730.1
G6N56_RS03155	695806	697320	eccB	type VII secretion protein EccB	WP_085256729.1
G6N56_RS03160	697317	701480	eccCa	type VII secretion protein EccCa	WP_085256728.1
G6N56_RS03165	701521	702693	0	cytochrome P450	WP_085256727.1
G6N56_RS03170	702939	703133	0	ferredoxin	WP_085256726.1
G6N56_RS03175	703683	704933	0	PPE family protein	WP_085256725.1
G6N56_RS03180	705100	705399	0	PE family protein	WP_085256724.1
G6N56_RS03185	705411	706643	0	PPE family protein	WP_085256723.1
G6N56_RS03190	706815	707741	0	LysR family transcriptional regulator	WP_085256722.1
G6N56_RS03195	707827	710178	0	hypothetical protein	WP_085256721.1
G6N56_RS03200	710371	711399	0	ABC transporter substrate-binding protein	WP_232069201.1
G6N56_RS28745	711396	713048	0	ATP-binding cassette domain-containing protein	WP_232069202.1
G6N56_RS03215	713099	714190	0	hypothetical protein	WP_180150470.1
G6N56_RS03220	714265	715500	0	BTAD domain-containing putative transcriptional regulator	WP_085256719.1
G6N56_RS03225	715692	716003	0	DUF732 domain-containing protein	WP_232069203.1
G6N56_RS03230	716196	717569	0	PPE family protein	WP_085256717.1
G6N56_RS03235	717588	718904	0	PPE domain-containing protein	WP_142280688.1
G6N56_RS03240	719958	720257	0	PE family protein	WP_085256715.1
G6N56_RS03245	720609	720908	0	PE family protein	WP_085256714.1
G6N56_RS03260	721843	722745	0	ESX secretion-associated protein EspG	WP_085256712.1
G6N56_RS03270	724516	726354	0	type VII secretion-associated serine protease mycosin	WP_085256710.1
G6N56_RS03275	726374	727576	eccE	type VII secretion protein EccE	WP_085256709.1
G6N56_RS03280	727573	729405	eccA	type VII secretion AAA-ATPase EccA	WP_085256708.1
G6N56_RS03285	730215	731426	0	PPE family protein	WP_085256707.1
G6N56_RS03290	732150	733325	0	PPE family protein	WP_085256706.1
G6N56_RS29380	733548	733682	0	hypothetical protein	WP_264020926.1
G6N56_RS03300	734323	735633	0	PPE family protein	WP_085256705.1
G6N56_RS03305	735704	737107	0	PPE family protein	WP_085256704.1
G6N56_RS03310	737137	737460	0	DUF732 domain-containing protein	WP_085256703.1
G6N56_RS03315	737607	738314	0	MgtC/SapB family protein	WP_085256901.1
G6N56_RS03320	738411	739349	0	sterol desaturase family protein	WP_085256702.1

G6N56_RS03325	739450	740121		0	hypothetical protein	WP_085256900.1
G6N56_RS03330	740131	740571		0	nitroreductase family deazaflavin-dependent oxidoreductase	WP_085256701.1
G6N56_RS03335	740688	741386		0	TetR/AcrR family transcriptional regulator	WP_085256700.1
G6N56_RS03340	741487	741987		0	VOC family protein	WP_085256699.1
G6N56_RS03345	742037	743497		0	FAD-binding protein	WP_085256698.1
G6N56_RS03350	743857	745782		0	ABC transporter ATP-binding protein/permease	WP_085256697.1
G6N56_RS03355	745887	747530		0	acetolactate synthase	WP_142280686.1
G6N56_RS03360	747582	749906	secA2		accessory Sec system translocase SecA2	WP_142280685.1
G6N56_RS03370	750630	751544		0	DUF881 domain-containing protein	WP_085256695.1
G6N56_RS03375	751576	751908		0	DUF1290 domain-containing protein	WP_085256694.1
G6N56_RS03380	751937	752767		0	DUF881 domain-containing protein	WP_085256693.1
G6N56_RS03385	752794	753192	gcvH		glycine cleavage system protein GcvH	WP_085256692.1
G6N56_RS03390	753527	754012		0	glycogen accumulation regulator GarA	WP_085256691.1
G6N56_RS03395	754012	754761		0	MerR family transcriptional regulator	WP_085256690.1
G6N56_RS03400	754878	755372		0	bifunctional nuclease family protein	WP_085256899.1
G6N56_RS03405	755799	756425		0	MerR family transcriptional regulator	WP_085256689.1
G6N56_RS03410	756818	759643	gcvP		aminomethyl-transferring glycine dehydrogenase	WP_085256688.1
G6N56_RS03415	759679	761793		0	substrate-binding domain-containing protein	WP_085256687.1
G6N56_RS03420	761927	764122		0	malate synthase G	WP_085256686.1
G6N56_RS03425	764413	765471		0	hemolysin family protein	WP_085256685.1
G6N56_RS03430	765471	766826		0	hemolysin family protein	WP_085256684.1
G6N56_RS03435	767014	768450		0	GuaB1 family IMP dehydrogenase-related protein	WP_085256683.1
G6N56_RS03440	768481	769929	gndA		NADP-dependent phosphogluconate dehydrogenase	WP_085256682.1
G6N56_RS03445	770027	770977		0	M56 family metallopeptidase	WP_085256681.1
G6N56_RS03450	770992	771411		0	BlaI/MecI/CopY family transcriptional regulator	WP_085256898.1
G6N56_RS03455	771766	772188		0	PaaI family thioesterase	WP_142280683.1
G6N56_RS03465	773429	773731		0	urease subunit gamma	WP_085256680.1
G6N56_RS03470	773728	774042		0	urease subunit beta	WP_085256679.1
G6N56_RS03475	774039	775760		0	urease subunit alpha	WP_085256678.1
G6N56_RS03480	775778	776419		0	urease accessory UreF family protein	WP_085256896.1
G6N56_RS03485	776430	777119	ureG		urease accessory protein UreG	WP_085256677.1

G6N56_RS03495	777750	779138		0	NAD(P)/FAD-dependent oxidoreductase	WP_085256675.1
G6N56_RS03500	779312	780235		0	LLM class F420-dependent oxidoreductase	WP_180150547.1
G6N56_RS03505	780265	780942		0	SDR family oxidoreductase	WP_085256895.1
G6N56_RS03510	781174	781941	modA		molybdate ABC transporter substrate-binding protein	WP_085256673.1
G6N56_RS03515	781950	782744		0	ABC transporter permease	WP_085256672.1
G6N56_RS03520	782751	783845		0	sulfate/molybdate ABC transporter ATP-binding protein	WP_085256671.1
G6N56_RS03525	783942	784961		0	alanine and proline-rich secreted protein Apa	WP_163645079.1
G6N56_RS03530	785074	785376		0	GlsB/YeaQ/YmgE family stress response membrane protein	WP_085257135.1
G6N56_RS03535	785432	786460		0	zinc-binding alcohol dehydrogenase family protein	WP_085257136.1
G6N56_RS03540	786467	787225		0	CPBP family intramembrane metalloprotease	WP_085257137.1
G6N56_RS03545	787337	789751		0	phosphoketolase family protein	WP_085257138.1
G6N56_RS03550	789798	790646		0	SDR family oxidoreductase	WP_085257139.1
G6N56_RS03555	790760	791263		0	GNAT family N-acetyltransferase	WP_085257140.1
G6N56_RS03560	791914	793131		0	sodium-dependent bicarbonate transport family permease	WP_085257141.1
G6N56_RS03565	793278	794192		0	undecaprenyl-diphosphate phosphatase	WP_085257142.1
G6N56_RS03570	794334	796757		0	CoA transferase	WP_085257374.1
G6N56_RS03575	796776	798269		0	acetyl-CoA acetyltransferase	WP_085257143.1
G6N56_RS03580	798398	800479		0	NAD(P)H-binding protein	WP_085257144.1
G6N56_RS03585	800484	801209		0	DUF72 domain-containing protein	WP_085257145.1
G6N56_RS03590	801206	801841		0	endonuclease	WP_085257146.1
G6N56_RS03595	801976	802308		0	TfoX/Sxy family protein	WP_085257147.1
G6N56_RS03600	802391	804712	lon		endopeptidase La	WP_085257148.1
G6N56_RS03605	804888	806150		0	alpha-hydroxy acid oxidase	WP_085257149.1
G6N56_RS03610	806187	806624		0	DUF1810 domain-containing protein	WP_085257150.1
G6N56_RS03615	806621	807931		0	alpha-amylase family glycosyl hydrolase	WP_085257151.1
G6N56_RS03620	807942	808478		0	carboxymuconolactone decarboxylase family protein	WP_085257152.1
G6N56_RS03625	808489	809076		0	peroxiredoxin	WP_085257153.1
G6N56_RS03630	809185	810120		0	hydrogen peroxide-inducible genes activator	WP_085257154.1
G6N56_RS03635	810148	810849		0	EthD domain-containing protein	WP_085257155.1
G6N56_RS03640	810859	811302		0	TIGR03618 family F420-dependent PPOX class oxidoreductase	WP_085257156.1
G6N56_RS03645	811407	812159		0	enoyl-CoA hydratase-related protein	WP_085257157.1

G6N56_RS03650	812172	812351		0	(2Fe-2S)-binding protein	WP_085257158.1
G6N56_RS03655	812682	813161	bfr		bacterioferritin	WP_085257375.1
G6N56_RS03660	813197	815284		0	MDR family MFS transporter	WP_085257376.1
G6N56_RS03665	815327	815821		0	carboxymuconolactone decarboxylase family protein	WP_085257159.1
G6N56_RS03670	815931	816812		0	TIGR03854 family LLM class F420-dependent oxidoreductase	WP_085257160.1
G6N56_RS03675	816855	818219		0	glutamine synthetase family protein	WP_085257161.1
G6N56_RS03680	818226	819320		0	amidohydrolase family protein	WP_085257162.1
G6N56_RS03685	819352	820194		0	lysophospholipid acyltransferase family protein	WP_085257163.1
G6N56_RS03690	820194	820628		0	SRPBCC family protein	WP_085257164.1
G6N56_RS03695	820726	821184		0	nuclear transport factor 2 family protein	WP_085257165.1
G6N56_RS03700	821217	821777		0	NifU family protein	WP_085257377.1
G6N56_RS03705	821780	823717		0	ferrous iron transporter B	WP_085257166.1
G6N56_RS03710	823714	823986		0	FeoA family protein	WP_085257167.1
G6N56_RS03715	824118	825446		0	cytochrome P450	WP_085257378.1
G6N56_RS03720	825484	825906		0	lipoprotein LpqH	WP_085257168.1
G6N56_RS03725	825917	826750		0	SDR family oxidoreductase	WP_085257169.1
G6N56_RS03730	826757	827230		0	SRPBCC family protein	WP_163645080.1
G6N56_RS03735	827266	827676		0	transglycosylase family protein	WP_142280732.1
G6N56_RS03740	827807	828385		0	chorismate mutase	WP_085257380.1
G6N56_RS03745	828463	829101		0	hypothetical protein	WP_232069204.1
G6N56_RS03750	829173	830165	ag85B		diacylglycerol acyltransferase/mycolyltransferase Ag85B	WP_085257171.1
G6N56_RS03755	830707	831828		0	hypothetical protein	WP_085257382.1
G6N56_RS03760	831845	832777		0	class I SAM-dependent methyltransferase	WP_085257172.1
G6N56_RS03765	832871	833419		0	YceI family protein	WP_085257173.1
G6N56_RS03770	833433	834446		0	alcohol dehydrogenase catalytic domain-containing protein	WP_085257174.1
G6N56_RS03775	834456	835727		0	cytochrome P450	WP_085257175.1
G6N56_RS03780	836030	836434		0	hypothetical protein	WP_085257176.1
G6N56_RS03785	836446	836736		0	hypothetical protein	WP_085257177.1
G6N56_RS03790	836793	837017		0	hypothetical protein	WP_085257178.1
G6N56_RS03795	837024	838154		0	nitronate monooxygenase family protein	WP_085257179.1
G6N56_RS03800	838304	838768		0	hypothetical protein	WP_085257180.1

G6N56_RS03810	839320	841278		0	NAD(P)/FAD-dependent oxidoreductase	WP_085257181.1
G6N56_RS03820	842308	843105		0	HAD-IB family hydrolase	WP_085257183.1
G6N56_RS03825	843102	844013		0	class I SAM-dependent methyltransferase	WP_085257184.1
G6N56_RS03830	844069	844500	dtd		D-aminoacyl-tRNA deacylase	WP_085257185.1
G6N56_RS03840	844830	846164		0	competence/damage-inducible protein A	WP_232069296.1
G6N56_RS03845	846167	847414		0	sialate:H+ symport family MFS transporter	WP_085257188.1
G6N56_RS03850	847525	848589		0	amidohydrolase family protein	WP_085257189.1
G6N56_RS03860	849015	849986		0	FAD-dependent oxidoreductase	WP_085257191.1
G6N56_RS03865	850003	850464		0	hypothetical protein	WP_085257192.1
G6N56_RS03870	850497	850898		0	hypothetical protein	WP_142280733.1
G6N56_RS03880	851974	852468		0	SRPBCC family protein	WP_085257195.1
G6N56_RS03885	852468	853109		0	nitroreductase family protein	WP_085257383.1
G6N56_RS03890	853370	854329		0	nitronate monooxygenase	WP_197746653.1
G6N56_RS03895	854326	854718		0	tautomerase family protein	WP_085257196.1
G6N56_RS03900	854812	855693		0	LysR family transcriptional regulator	WP_085257197.1
G6N56_RS03905	855718	856311		0	YbhB/YbcL family Raf kinase inhibitor-like protein	WP_085257198.1
G6N56_RS03910	856362	856805		0	SRPBCC family protein	WP_085257199.1
G6N56_RS03915	856879	857886		0	medium chain dehydrogenase/reductase family protein	WP_085257200.1
G6N56_RS03920	857982	858761		0	MBL fold metallo-hydrolase	WP_085257201.1
G6N56_RS03925	858829	861117	aceA		isocitrate lyase ICL2	WP_085257202.1
G6N56_RS28760	861343	861750		0	hypothetical protein	WP_163645081.1
G6N56_RS03940	863064	864641		0	FAD-dependent monooxygenase	WP_085257204.1
G6N56_RS03945	864730	865188		0	SRPBCC family protein	WP_085257205.1
G6N56_RS03950	865294	865449		0	hypothetical protein	WP_158090741.1
G6N56_RS03955	865446	866852		0	TIGR00366 family protein	WP_085257206.1
G6N56_RS03960	867095	868924		0	fatty acyl-AMP ligase	WP_085257207.1
G6N56_RS03965	869037	869702		0	hypothetical protein	WP_085257208.1
G6N56_RS03970	869736	870497		0	DUF2071 domain-containing protein	WP_085257384.1
G6N56_RS03975	870507	871280		0	SDR family oxidoreductase	WP_085257209.1
G6N56_RS03980	871334	871810		0	nuclear transport factor 2 family protein	WP_085257210.1
G6N56_RS03985	871815	872558		0	DJ-1/PfpI family protein	WP_085257211.1

G6N56_RS03990	872575	873525		0	DJ-1/PfpI family protein	WP_085257212.1
G6N56_RS03995	873635	874129	tpx		thiol peroxidase	WP_085257213.1
G6N56_RS04000	874134	875084		0	nitronate monooxygenase	WP_085257214.1
G6N56_RS04005	875081	875440		0	NYN domain-containing protein	WP_085257385.1
G6N56_RS04010	875637	876821		0	acyl-CoA dehydrogenase family protein	WP_085257215.1
G6N56_RS04020	877570	877896		0	hypothetical protein	WP_085257217.1
G6N56_RS04025	878079	878984		0	NAD(P)-dependent oxidoreductase	WP_085257218.1
G6N56_RS04030	879039	879617		0	TetR family transcriptional regulator	WP_085257386.1
G6N56_RS04040	880506	881360		0	lysophospholipid acyltransferase family protein	WP_085257220.1
G6N56_RS04050	882196	882798		0	heme-binding protein	WP_085257221.1
G6N56_RS04055	882801	883025		0	DUF2905 domain-containing protein	WP_085257222.1
G6N56_RS04060	883344	884354		0	NADP-dependent oxidoreductase	WP_085257223.1
G6N56_RS04065	884459	885124		0	hypothetical protein	WP_085257224.1
G6N56_RS04070	885148	885993		0	SAM-dependent methyltransferase	WP_085257225.1
G6N56_RS04075	885998	886825		0	glycoside hydrolase family 16 protein	WP_085257226.1
G6N56_RS04080	887022	887636		0	hypothetical protein	WP_158090742.1
G6N56_RS04090	889349	890131		0	class I SAM-dependent methyltransferase	WP_232069206.1
G6N56_RS04100	890932	892008		0	prenyltransferase	WP_085257231.1
G6N56_RS04110	892756	894012		0	glycosyltransferase family 4 protein	WP_085257233.1
G6N56_RS04115	894323	895876		0	GMC family oxidoreductase N-terminal domain-containing prot	WP_085257234.1
G6N56_RS04120	895906	896694		0	DUF1906 domain-containing protein	WP_085257235.1
G6N56_RS04125	896938	899178	katG		catalase/peroxidase HPI	WP_085257236.1
G6N56_RS04130	899212	899652		0	Fur family transcriptional regulator	WP_163645082.1
G6N56_RS04135	899744	901030		0	Ig-like domain-containing protein	WP_085257237.1
G6N56_RS04140	901146	901952		0	hypothetical protein	WP_085257238.1
G6N56_RS04145	901999	903510		0	FAD-dependent oxidoreductase	WP_180150473.1
G6N56_RS04150	903513	904067		0	tat pathway signal sequence	WP_085257239.1
G6N56_RS04155	904121	905161		0	class I SAM-dependent methyltransferase	WP_085257240.1
G6N56_RS29390	905244	906671		0	PPE domain-containing protein	WP_085257241.1
G6N56_RS04165	906718	908136		0	PPE domain-containing protein	WP_085257242.1
G6N56_RS04170	908969	909376		0	PPOX class F420-dependent oxidoreductase	WP_085257243.1

G6N56_RS04175	909562	909828	0	DUF732 domain-containing protein	WP_232069207.1
G6N56_RS04180	909965	910801	0	MOSC domain-containing protein	WP_085257245.1
G6N56_RS04185	910814	912070	0	cytochrome P450	WP_085257246.1
G6N56_RS04190	912085	913488	0	FAD-dependent oxidoreductase	WP_085257247.1
G6N56_RS04195	913485	913700	0	ferredoxin	WP_085257248.1
G6N56_RS04200	914042	914290	0	DUF6480 family protein	WP_085257249.1
G6N56_RS04205	914479	915093	0	hypothetical protein	WP_085257250.1
G6N56_RS04210	915241	917250	0	serine/threonine-protein kinase	WP_085257251.1
G6N56_RS04215	917256	918482	0	hypothetical protein	WP_085257252.1
G6N56_RS04220	918479	919774	0	hypothetical protein	WP_232069208.1
G6N56_RS04225	919968	920963	0	sensor domain-containing protein	WP_085257253.1
G6N56_RS04230	920998	921981	0	sensor domain-containing protein	WP_085257254.1
G6N56_RS04235	922255	922494	0	hypothetical protein	WP_085257255.1
G6N56_RS04245	923239	923910	0	IF2 family translation initiation factor	WP_085257390.1
G6N56_RS04250	924045	924695	0	DUF1345 domain-containing protein	WP_085257256.1
G6N56_RS04255	924748	925410	0	cutinase family protein	WP_085257257.1
G6N56_RS04260	925570	925833	0	alpha/beta hydrolase	WP_085257258.1
G6N56_RS04265	925846	926394	0	thioredoxin family protein	WP_085257259.1
G6N56_RS04270	926694	928271	0	PecA family PE domain-processing aspartic protease	WP_085257260.1
G6N56_RS04275	928406	929326	0	DMT family transporter	WP_085257261.1
G6N56_RS04280	929374	930360	0	amidohydrolase family protein	WP_085257262.1
G6N56_RS04285	930387	930866	0	helix-turn-helix domain-containing protein	WP_085257263.1
G6N56_RS04290	930885	931157	0	hypothetical protein	WP_232069209.1
G6N56_RS04295	931710	933104	0	cytosine permease	WP_085257264.1
G6N56_RS04305	934504	935937	0	nicotinate phosphoribosyltransferase	WP_232069210.1
G6N56_RS04310	936029	936847	0	aldo/keto reductase	WP_085257267.1
G6N56_RS04315	937532	937798	0	hypothetical protein	WP_163645065.1
G6N56_RS04320	937827	939257	0	MFS transporter	WP_197746654.1
G6N56_RS04325	939304	940803	zwf	glucose-6-phosphate dehydrogenase	WP_085257269.1
G6N56_RS04330	940961	941734	0	enoyl-CoA hydratase/isomerase family protein	WP_085257270.1
G6N56_RS04335	941761	941940	0	hypothetical protein	WP_085257271.1

G6N56_RS04340	942158	946066	hrpA		ATP-dependent RNA helicase HrpA	WP_085257272.1
G6N56_RS04345	946111	946992		0	CoA ester lyase	WP_085257273.1
G6N56_RS04360	948626	949204		0	zeta toxin family protein	WP_085257274.1
G6N56_RS04365	949217	949630		0	hypothetical protein	WP_085257275.1
G6N56_RS04375	949998	951431		0	MFS transporter	WP_158090744.1
G6N56_RS04380	951654	952397		0	GntR family transcriptional regulator	WP_085257277.1
G6N56_RS04385	952411	953115		0	phosphonatase-like hydrolase	WP_085257278.1
G6N56_RS04390	953163	954155		0	zinc-binding dehydrogenase	WP_085257393.1
G6N56_RS04395	954204	954938		0	sulfite exporter TauE/SafE family protein	WP_085257279.1
G6N56_RS28795	955113	955886		0	hypothetical protein	WP_232069211.1
G6N56_RS04405	956013	957248		0	osmoprotectant NAGGN system M42 family peptidase	WP_085257281.1
G6N56_RS04410	957581	958417		0	LLM class F420-dependent oxidoreductase	WP_085257282.1
G6N56_RS04415	958614	960062		0	FAD-binding oxidoreductase	WP_085257283.1
G6N56_RS04420	960105	960920		0	DUF2470 domain-containing protein	WP_085257284.1
G6N56_RS04425	961183	961971		0	NlpC/P60 family protein	WP_142280736.1
G6N56_RS04430	962055	962711		0	TetR/AcrR family transcriptional regulator	WP_232069212.1
G6N56_RS04435	962758	963396		0	class I SAM-dependent methyltransferase	WP_085257287.1
G6N56_RS04440	963425	963976		0	FKBP-type peptidyl-prolyl cis-trans isomerase	WP_232069301.1
G6N56_RS04450	964311	964859		0	hypothetical protein	WP_085257289.1
G6N56_RS04455	964890	966080		0	serine hydrolase	WP_232069302.1
G6N56_RS04460	966095	967555		0	biotin carboxylase	WP_085257290.1
G6N56_RS04465	967736	968092		0	TetR-like C-terminal domain-containing protein	WP_085257291.1
G6N56_RS04470	968326	968928		0	TetR/AcrR family transcriptional regulator	WP_085257292.1
G6N56_RS04475	969026	969502		0	DUF1772 domain-containing protein	WP_085257293.1
G6N56_RS04480	969554	969871		0	NIPSNAP family protein	WP_085257294.1
G6N56_RS04485	969893	970270		0	DUF4267 domain-containing protein	WP_085257395.1
G6N56_RS04490	970299	970709		0	FAD-binding protein	WP_158090747.1
G6N56_RS04495	970706	970885		0	hypothetical protein	WP_142280737.1
G6N56_RS28805	971604	972134		0	sensor domain-containing protein	WP_232069303.1
G6N56_RS04505	972417	972647		0	DUF167 domain-containing protein	WP_085257297.1
G6N56_RS04510	973049	973981		0	cytochrome P450	WP_158090748.1

G6N56_RS04515	974090	974917		0	DUF1906 domain-containing protein	WP_085257299.1
G6N56_RS04520	975473	975877		0	nuclear transport factor 2 family protein	WP_085257301.1
G6N56_RS04525	975897	976589		0	WYL domain-containing protein	WP_085257302.1
G6N56_RS04535	977164	977475		0	type II toxin-antitoxin system PemK/MazF family toxin	WP_036446035.1
G6N56_RS04540	978039	978233		0	hypothetical protein	WP_142280740.1
G6N56_RS04545	978340	978945		0	class I SAM-dependent methyltransferase	WP_085257396.1
G6N56_RS04550	979061	979264		0	cold-shock protein	WP_085257303.1
G6N56_RS04555	979369	980643		0	DEAD/DEAH box helicase	WP_085257304.1
G6N56_RS04560	981244	981465		0	hypothetical protein	WP_142280741.1
G6N56_RS04565	981555	982013		0	DUF5709 domain-containing protein	WP_085257306.1
G6N56_RS04570	982249	982545		0	hypothetical protein	WP_085257307.1
G6N56_RS04575	982572	983984		0	phosphoribosyltransferase	WP_085257308.1
G6N56_RS29400	984498	985982		0	PPE family protein	WP_264019419.1
G6N56_RS29405	986047	986172		0	hypothetical protein	WP_264019418.1
G6N56_RS04585	986334	989105		0	LuxR family transcriptional regulator	WP_158090749.1
G6N56_RS04590	989261	990274		0	LLM class flavin-dependent oxidoreductase	WP_085257310.1
G6N56_RS04595	990299	990940		0	zinc-binding dehydrogenase	WP_163645083.1
G6N56_RS04605	992379	992726		0	alcohol dehydrogenase catalytic domain-containing protein	WP_163645084.1
G6N56_RS04610	993043	993609		0	hypothetical protein	WP_085257312.1
G6N56_RS04615	993696	995438	oxc		oxalyl-CoA decarboxylase	WP_085257313.1
G6N56_RS04620	995464	996726	frc		formyl-CoA transferase	WP_085257314.1
G6N56_RS04625	996760	997593		0	alpha/beta hydrolase	WP_085257315.1
G6N56_RS04630	997608	998384		0	aldehyde dehydrogenase family protein	WP_232069305.1
G6N56_RS28835	998404	998583		0	aldehyde dehydrogenase family protein	WP_232069213.1
G6N56_RS04635	998717	999424		0	TetR/AcrR family transcriptional regulator	WP_085257316.1
G6N56_RS04640	999614	1001155		0	GMC family oxidoreductase N-terminal domain-containing prot	WP_197746670.1
G6N56_RS04645	1001315	1002238		0	alpha/beta hydrolase	WP_085257318.1
G6N56_RS04650	1002295	1002903		0	TetR family transcriptional regulator	WP_085257319.1
G6N56_RS04655	1003105	1003539		0	MmpS family transport accessory protein	WP_269473813.1
G6N56_RS04660	1003536	1006442		0	MMPL family transporter	WP_085257321.1
G6N56_RS04665	1006578	1007360		0	SDR family oxidoreductase	WP_085257397.1

G6N56_RS04670	1007925	1008830	mmaA4		hydroxymycolate synthase MmaA4	WP_142280742.1
G6N56_RS04675	1008884	1009273		0	DUF1801 domain-containing protein	WP_085257322.1
G6N56_RS04680	1009385	1010185		0	thioesterase family protein	WP_085257323.1
G6N56_RS04685	1010362	1011357		0	NAD-dependent epimerase/dehydratase family protein	WP_085257324.1
G6N56_RS04695	1012368	1013696		0	acyltransferase	WP_085257326.1
G6N56_RS04700	1013971	1014285		0	hypothetical protein	WP_085257327.1
G6N56_RS04705	1014627	1015307		0	TetR/AcrR family transcriptional regulator	WP_085257328.1
G6N56_RS04710	1015861	1016655		0	ABC transporter permease	WP_085257329.1
G6N56_RS04715	1016666	1017535		0	ABC transporter permease	WP_085257400.1
G6N56_RS04720	1017542	1018861		0	MCE family protein	WP_085257330.1
G6N56_RS04725	1018858	1019898		0	virulence factor Mce family protein	WP_085257331.1
G6N56_RS04730	1019895	1021454		0	virulence factor Mce family protein	WP_085257332.1
G6N56_RS04735	1021451	1023007		0	virulence factor Mce family protein	WP_085257333.1
G6N56_RS04745	1024173	1025723		0	MlaD family protein	WP_085257334.1
G6N56_RS04750	1025875	1026477		0	type II toxin-antitoxin system prevent-host-death family antitoxi	WP_085257335.1
G6N56_RS04755	1026602	1027054		0	universal stress protein	WP_085257336.1
G6N56_RS04760	1027070	1027714		0	TetR/AcrR family transcriptional regulator	WP_085257337.1
G6N56_RS04765	1027812	1028942		0	VOC family protein	WP_085257338.1
G6N56_RS04770	1028939	1029883		0	fumarylacetoacetate hydrolase family protein	WP_085257339.1
G6N56_RS04775	1029880	1031409		0	bifunctional 3-(3-hydroxy-phenyl)propionate/3-hydroxycinnami	WP_085257340.1
G6N56_RS04780	1031492	1032382		0	universal stress protein	WP_085257341.1
G6N56_RS04785	1032635	1033546		0	formate/nitrite transporter family protein	WP_085257342.1
G6N56_RS04790	1033543	1033812		0	hypothetical protein	WP_085257343.1
G6N56_RS04795	1033809	1034438		0	RNA polymerase sigma factor	WP_085257344.1
G6N56_RS04800	1034449	1035519		0	acyl-CoA dehydrogenase family protein	WP_085257345.1
G6N56_RS04805	1035516	1036001	cynS		cyanase	WP_085257346.1
G6N56_RS04810	1036022	1036546		0	OsmC family protein	WP_085257347.1
G6N56_RS04815	1036896	1037732		0	DUF3097 domain-containing protein	WP_085257348.1
G6N56_RS04820	1037950	1038237		0	DUF3349 domain-containing protein	WP_085257349.1
G6N56_RS04825	1038234	1039235		0	patatin-like phospholipase family protein	WP_085257350.1
G6N56_RS04830	1039232	1040302	ugpC		sn-glycerol-3-phosphate ABC transporter ATP-binding protein U	WP_085257351.1

G6N56_RS04840	1041134	1042093		0	sugar ABC transporter permease	WP_085257353.1
G6N56_RS04845	1042090	1043412		0	sugar ABC transporter substrate-binding protein	WP_085257354.1
G6N56_RS04850	1043699	1044049		0	DoxX family protein	WP_085257355.1
G6N56_RS04855	1044054	1044851		0	ketosteroid isomerase family protein	WP_085257356.1
G6N56_RS04860	1044851	1045411	pncA		pyrazinamidase PncA	WP_085257357.1
G6N56_RS04865	1045511	1046578		0	DoxX family protein	WP_163645085.1
G6N56_RS04870	1046575	1047060		0	gluconokinase	WP_085257402.1
G6N56_RS04875	1047084	1049087		0	phosphotransferase	WP_085257401.1
G6N56_RS04880	1049105	1050610		0	carboxylesterase/lipase family protein	WP_085257359.1
G6N56_RS04885	1050747	1051427		0	hypothetical protein	WP_085257360.1
G6N56_RS04890	1051681	1054302		0	sugar epimerase family protein	WP_085257361.1
G6N56_RS04895	1054324	1066830		0	type I polyketide synthase	WP_085257362.1
G6N56_RS28520	1067171	1067563		0	hypothetical protein	WP_085257363.1
G6N56_RS04905	1067686	1068021	rbpA		RNA polymerase-binding protein RbpA	WP_007170328.1
G6N56_RS04920	1070926	1072458		0	amidohydrolase	WP_085257403.1
G6N56_RS04925	1072568	1073080		0	FxsA family protein	WP_085257366.1
G6N56_RS04930	1073154	1073855		0	dienelactone hydrolase family protein	WP_085257367.1
G6N56_RS04935	1073903	1074766		0	type 1 glutamine amidotransferase domain-containing protein	WP_085257368.1
G6N56_RS04940	1074770	1075156		0	PPOX class F420-dependent oxidoreductase	WP_085257369.1
G6N56_RS04945	1075423	1076655		0	DUF1214 domain-containing protein	WP_180150478.1
G6N56_RS04950	1076652	1077797		0	sulfotransferase	WP_085257370.1
G6N56_RS04955	1077803	1078447		0	TetR/AcrR family transcriptional regulator	WP_142280746.1
G6N56_RS04960	1078475	1079278		0	SDR family oxidoreductase	WP_085257372.1
G6N56_RS04965	1079300	1082872	cobN		cobaltochelatase subunit CobN	WP_085257373.1
G6N56_RS04970	1082876	1083547		0	alpha/beta fold hydrolase	WP_085257405.1
G6N56_RS04980	1085421	1086533	cobG		precorrin-3B synthase	WP_085254017.1
G6N56_RS04985	1086538	1087164		0	precorrin-8X methylmutase	WP_085254018.1
G6N56_RS04995	1088627	1091572		0	RND family transporter	WP_085254020.1
G6N56_RS05000	1091569	1092000		0	MmpS family transport accessory protein	WP_085254097.1
G6N56_RS05005	1092288	1093838		0	PPE family protein	WP_085254021.1
G6N56_RS28845	1094793	1095347	sigC		RNA polymerase sigma factor SigC	WP_232069306.1

G6N56_RS05015	1095344	1096069		0	cobalt-precorrin-6A reductase	WP_085254022.1
G6N56_RS05020	1096069	1096824	cobM		precorrin-4 C(11)-methyltransferase	WP_085254099.1
G6N56_RS05025	1096821	1097993	cbiE		precorrin-6y C5,15-methyltransferase (decarboxylating) subuni	WP_085254023.1
G6N56_RS05030	1097990	1098739		0	SDR family NAD(P)-dependent oxidoreductase	WP_085254024.1
G6N56_RS05035	1098775	1099188		0	F420-dependent biliverdin reductase	WP_085254025.1
G6N56_RS05040	1099199	1099366		0	hypothetical protein	WP_232069214.1
G6N56_RS05045	1099499	1099720		0	hypothetical protein	WP_085254027.1
G6N56_RS05050	1099826	1100038		0	hypothetical protein	WP_085254028.1
G6N56_RS05055	1100069	1102585		0	ADP-ribosyltransferase	WP_085254029.1
G6N56_RS05060	1102668	1103795		0	Xaa-Pro peptidase family protein	WP_085254030.1
G6N56_RS05065	1103898	1104854		0	5'-3' exonuclease	WP_085254031.1
G6N56_RS05070	1104859	1105602		0	DUF4333 domain-containing protein	WP_085254032.1
G6N56_RS05075	1105668	1108448		0	RNA helicase	WP_085254100.1
G6N56_RS05080	1108542	1109498	tatC		twin-arginine translocase subunit TatC	WP_142280423.1
G6N56_RS05085	1109545	1109811	tatA		Sec-independent protein translocase subunit TatA	WP_085254033.1
G6N56_RS05090	1109903	1110877		0	YafY family protein	WP_085254034.1
G6N56_RS05095	1110874	1111887		0	YafY family protein	WP_085254035.1
G6N56_RS05100	1112041	1113399	pafA		Pup--protein ligase	WP_142280424.1
G6N56_RS05105	1113587	1114426	prcA		proteasome subunit alpha	WP_085254037.1
G6N56_RS05110	1114423	1115310	prcB		proteasome subunit beta	WP_085254038.1
G6N56_RS05115	1115307	1115501		0	ubiquitin-like protein Pup	WP_085254039.1
G6N56_RS05120	1115621	1117129	dop		pup deamidase/depupylase	WP_085254040.1
G6N56_RS05125	1117229	1117984		0	sulfite exporter TauE/SafE family protein	WP_085254041.1
G6N56_RS05135	1118269	1118889		0	hypothetical protein	WP_142280431.1
G6N56_RS05140	1119048	1120877	arc		proteasome ATPase	WP_085254102.1
G6N56_RS05145	1121173	1121817		0	hypothetical protein	WP_085254043.1
G6N56_RS05150	1121826	1122125		0	DUF503 family protein	WP_085254044.1
G6N56_RS05155	1122130	1122972	trmI		tRNA (adenine(58)-N(1))-methyltransferase TrmI	WP_085254045.1
G6N56_RS05160	1123046	1123915		0	RecB family exonuclease	WP_085254046.1
G6N56_RS05165	1124119	1125489		0	FAD-containing oxidoreductase	WP_085254047.1
G6N56_RS05170	1125486	1125968		0	DUF4126 domain-containing protein	WP_085254048.1

G6N56_RS05175	1126085	1126936	hisG		ATP phosphoribosyltransferase	WP_085254049.1
G6N56_RS05180	1126939	1127220		0	phosphoribosyl-ATP diphosphatase	WP_085254103.1
G6N56_RS05190	1127830	1131606	metH		methionine synthase	WP_142280425.1
G6N56_RS05195	1131963	1132853		0	PAC2 family protein	WP_180150480.1
G6N56_RS05200	1132905	1133792		0	SDR family oxidoreductase	WP_085254052.1
G6N56_RS05205	1133829	1135064	mshC		cysteine--1-D-myo-inosityl 2-amino-2-deoxy-alpha-D-glucopyra	WP_085254053.1
G6N56_RS05210	1135091	1135870		0	inositol monophosphatase family protein	WP_085254105.1
G6N56_RS05215	1136215	1137003		0	SCO1664 family protein	WP_142280426.1
G6N56_RS05220	1137023	1137610		0	DUF3090 domain-containing protein	WP_085254055.1
G6N56_RS05225	1137687	1138421		0	histidine phosphatase family protein	WP_085254056.1
G6N56_RS05230	1138515	1138817		0	hypothetical protein	WP_085254057.1
G6N56_RS05235	1138971	1139990		0	hypothetical protein	WP_085254058.1
G6N56_RS05240	1140044	1140250		0	DUF5703 family protein	WP_085254106.1
G6N56_RS05245	1140277	1141353		0	quinone-dependent dihydroorotate dehydrogenase	WP_163645199.1
G6N56_RS05250	1141350	1142771		0	HNH endonuclease signature motif containing protein	WP_085254060.1
G6N56_RS05255	1142943	1144790		0	PE family protein	WP_085254107.1
G6N56_RS05260	1144798	1145328		0	YbhB/YbcL family Raf kinase inhibitor-like protein	WP_085254061.1
G6N56_RS05265	1145358	1146725		0	M20/M25/M40 family metallo-hydrolase	WP_085254062.1
G6N56_RS05275	1146942	1147106		0	hypothetical protein	WP_158090671.1
G6N56_RS05285	1148146	1149120		0	helix-turn-helix domain-containing protein	WP_197746655.1
G6N56_RS05290	1149121	1149975		0	alpha/beta fold hydrolase	WP_085254063.1
G6N56_RS05295	1149999	1150637		0	NAD(P)H-dependent oxidoreductase	WP_085254064.1
G6N56_RS05300	1150823	1151821		0	SDR family NAD(P)-dependent oxidoreductase	WP_232069215.1
G6N56_RS05310	1152163	1152630		0	nuclear transport factor 2 family protein	WP_085254066.1
G6N56_RS05315	1152651	1153583		0	LysR family transcriptional regulator	WP_158090673.1
G6N56_RS05320	1153753	1154577		0	alpha/beta hydrolase	WP_085254068.1
G6N56_RS05325	1154574	1155242		0	cysteine hydrolase	WP_085254069.1
G6N56_RS05330	1155266	1156105		0	MBL fold metallo-hydrolase	WP_085254070.1
G6N56_RS05335	1156413	1157048		0	pyridoxamine 5'-phosphate oxidase family protein	WP_085254071.1
G6N56_RS05340	1157111	1158868		0	MOSC domain-containing protein	WP_085254072.1
G6N56_RS05345	1159021	1160682		0	PP2C family protein-serine/threonine phosphatase	WP_085254073.1

G6N56_RS05350	1160679	1160921		0	hypothetical protein	WP_085254074.1
G6N56_RS05355	1160918	1161259		0	STAS domain-containing protein	WP_085254075.1
G6N56_RS05360	1161288	1161965		0	phosphoribosyltransferase	WP_085254076.1
G6N56_RS05365	1161975	1162346		0	phage holin family protein	WP_085254077.1
G6N56_RS05370	1162410	1163213		0	DivIVA domain-containing protein	WP_085254078.1
G6N56_RS05375	1163475	1163765		0	YggT family protein	WP_023364806.1
G6N56_RS05380	1163923	1164579		0	cell division protein SepF	WP_085254079.1
G6N56_RS05385	1164645	1165457		0	YggS family pyridoxal phosphate enzyme	WP_142280436.1
G6N56_RS05390	1165463	1166185	pgeF		peptidoglycan editing factor PgeF	WP_085254080.1
G6N56_RS05395	1166217	1167371	ftsZ		cell division protein FtsZ	WP_085254081.1
G6N56_RS05400	1167566	1168468		0	cell division protein FtsQ/DivIB	WP_232069307.1
G6N56_RS05405	1168510	1169958	murC		UDP-N-acetylmuramate--L-alanine ligase	WP_142280437.1
G6N56_RS05410	1170009	1171250	murG		undecaprenyldiphospho-muramoylpentapeptide beta-N-acetyl	WP_085254084.1
G6N56_RS05415	1171247	1173079	ftsW		putative lipid II flippase FtsW	WP_085254085.1
G6N56_RS05420	1173086	1174552	murD		UDP-N-acetylmuramoyl-L-alanine--D-glutamate ligase	WP_085254086.1
G6N56_RS05425	1174552	1175631	mraY		phospho-N-acetylmuramoyl-pentapeptide- transferase	WP_085254087.1
G6N56_RS05430	1175628	1177208	murF		UDP-N-acetylmuramoyl-tripeptide--D-alanyl-D- alanine ligase	WP_085254088.1
G6N56_RS05435	1177205	1178773		0	UDP-N-acetylmuramoyl-L-alanyl-D-glutamate--2, 6-diaminopir	WP_085254089.1
G6N56_RS05440	1178839	1179444		0	TetR/AcrR family transcriptional regulator	WP_180150483.1
G6N56_RS05445	1179514	1180623		0	FAD-dependent monooxygenase	WP_085254091.1
G6N56_RS05450	1180657	1182720		0	penicillin-binding protein 2	WP_085254092.1
G6N56_RS05455	1182717	1183967		0	hypothetical protein	WP_085254093.1
G6N56_RS05460	1183964	1185136	rsmH		16S rRNA (cytosine(1402)-N(4))-methyltransferase RsmH	WP_142280430.1
G6N56_RS05470	1185998	1186399		0	DUF3040 domain-containing protein	WP_085254096.1
G6N56_RS05475	1186729	1187349		0	N-acetyltransferase	WP_085257125.1
G6N56_RS05480	1187488	1188135		0	DUF3153 domain-containing protein	WP_085257130.1
G6N56_RS05485	1188132	1189028		0	mycobacterial-type methylenetetrahydrofolate reductase	WP_085257124.1
G6N56_RS05490	1189280	1190329		0	polyprenyl synthetase family protein	WP_085257129.1
G6N56_RS05495	1190332	1191858		0	alpha-(1->6)-mannopyranosyltransferase A	WP_085257123.1
G6N56_RS05500	1191899	1192303		0	Rv2175c family DNA-binding protein	WP_085257122.1
G6N56_RS05505	1192394	1193725		0	protein kinase	WP_180150485.1

G6N56_RS05510	1193810	1195198		0	3-deoxy-7-phosphoheptulonate synthase class II	WP_085257121.1	
G6N56_RS05515	1195257	1195742		0	polyadenylate-specific 3'-exoribonuclease AS	WP_085257120.1	
G6N56_RS05520	1195753	1196619		0	hypothetical protein	WP_180150551.1	
G6N56_RS05525	1196735	1198048		0	glycosyltransferase 87 family protein	WP_085257118.1	
G6N56_RS05530	1198045	1198773		0	lysophospholipid acyltransferase family protein	WP_085257117.1	
G6N56_RS05535	1198859	1199245		0	hypothetical protein	WP_085257116.1	
G6N56_RS05540	1199242	1200522		0	ArsA family ATPase	WP_085257115.1	
G6N56_RS05545	1200519	1200953		0	SRPBCC family protein	WP_085257114.1	
G6N56_RS05550	1201068	1201457		0	polyketide cyclase / dehydrase and lipid transport	WP_085257113.1	
G6N56_RS05555	1201607	1203412		0	long-chain fatty acid--CoA ligase	WP_085257112.1	
G6N56_RS05560	1203518	1204651	pimB		GDP-mannose-dependent alpha-(1-6)-phosphatidylinositol mo	WP_085257111.1	
G6N56_RS05565	1204661	1205506		0	DUF4157 domain-containing protein	WP_232069308.1	
G6N56_RS05570	1205541	1206674	ripC		peptidoglycan hydrolase RipC	WP_085257110.1	
G6N56_RS05575	1206843	1207088		0	hypothetical protein	WP_232069216.1	
G6N56_RS28855	1209017	1210096	trpD		anthranilate phosphoribosyltransferase	WP_158090739.1	
G6N56_RS05590	1210235	1210846		0	heme-copper oxidase subunit III	WP_085253630.1	
G6N56_RS05595	1210975	1211748		0	c-type cytochrome	WP_085253629.1	
G6N56_RS05600	1211798	1213003		0	ubiquinol-cytochrome c reductase iron-sulfur subunit	WP_085253628.1	
G6N56_RS05605	1213000	1214694		0	cytochrome bc complex cytochrome b subunit	WP_085253627.1	
G6N56_RS05610	1215050	1215691		0	DUF2561 family protein	WP_085253626.1	
G6N56_RS05615	1215691	1216647		0	MmpS family transport accessory protein	WP_085253625.1	
G6N56_RS05620	1216830	1217249		0	cytochrome c oxidase subunit 4	WP_085253624.1	
G6N56_RS05625	1217261	1218372		0	cytochrome c oxidase subunit II		0
G6N56_RS05635	1218529	1220463	asnB		asparagine synthase (glutamine-hydrolyzing)	WP_085253621.1	
G6N56_RS05640	1220531	1221505		0	carbohydrate kinase family protein	WP_085253620.1	
G6N56_RS05645	1221686	1222354		0	hypothetical protein	WP_085253619.1	
G6N56_RS05650	1222371	1222727		0	iron-sulfur cluster assembly accessory protein	WP_085253618.1	
G6N56_RS05655	1222797	1223873		0	glycerate kinase	WP_085253633.1	
G6N56_RS05660	1224055	1224735		0	DUF3043 domain-containing protein	WP_085253617.1	
G6N56_RS05665	1224762	1225298		0	bifunctional adenosylcobinamide kinase/adenosylcobinamide-	WP_085253616.1	
G6N56_RS05670	1225298	1226344	cobT		nicotinate-nucleotide--dimethylbenzimidazole phosphoribosylt	WP_085253615.1	

G6N56_RS05675	1226341	1227087		0	adenosylcobinamide-GDP ribazoletransferase	WP_085253614.1
G6N56_RS05680	1227152	1228258		0	branched-chain amino acid aminotransferase	WP_085253632.1
G6N56_RS05685	1228282	1229379	gcvT		glycine cleavage system aminomethyltransferase GcvT	WP_085253613.1
G6N56_RS05695	1230175	1231716		0	leucyl aminopeptidase	WP_085253631.1
G6N56_RS05700	1231725	1233314		0	NAD(P)/FAD-dependent oxidoreductase	WP_085253612.1
G6N56_RS05705	1233319	1233786		0	SRPBCC family protein	WP_232069217.1
G6N56_RS05710	1233930	1234274		0	aminopeptidase	WP_085253610.1
G6N56_RS05715	1234398	1236164		0	SDR family oxidoreductase	WP_085253609.1
G6N56_RS05720	1236338	1238143	sucB		2-oxoglutarate dehydrogenase, E2 component, dihydrolipoamid	WP_163645087.1
G6N56_RS05725	1238159	1239070		0	TIGR01777 family oxidoreductase	WP_085255680.1
G6N56_RS05730	1239120	1239827	lipB		lipoyl(octanoyl) transferase LipB	WP_085255671.1
G6N56_RS05735	1239824	1240756	lipA		lipoyl synthase	WP_085255670.1
G6N56_RS05740	1240786	1241538		0	DUF4191 domain-containing protein	WP_085255669.1
G6N56_RS05745	1241669	1242091		0	RDD family protein	WP_085255668.1
G6N56_RS05750	1242321	1243757	glnA		type I glutamate--ammonia ligase	WP_085255667.1
G6N56_RS05755	1243870	1245222		0	aldehyde dehydrogenase family protein	WP_085255666.1
G6N56_RS05760	1245264	1246061		0	ABC transporter permease	WP_085255665.1
G6N56_RS05770	1246936	1248135		0	ABC transporter substrate-binding protein	WP_085255679.1
G6N56_RS05775	1248178	1249215		0	ABC transporter ATP-binding protein	WP_142280594.1
G6N56_RS05780	1249291	1250037		0	GntR family transcriptional regulator	WP_085255662.1
G6N56_RS05785	1250040	1250744		0	cache domain-containing protein	WP_085255661.1
G6N56_RS05790	1250893	1251753		0	TIGR03619 family F420-dependent LLM class oxidoreductase	WP_085255678.1
G6N56_RS05795	1251750	1252394		0	PaaI family thioesterase	WP_085255660.1
G6N56_RS05800	1252391	1255375		0	bifunctional [glutamine synthetase] adenylyltransferase/[glutar	WP_085255659.1
G6N56_RS05805	1255436	1256776	glnA		type I glutamate--ammonia ligase	WP_085255658.1
G6N56_RS05810	1256868	1258421		0	alpha/beta hydrolase	WP_085255657.1
G6N56_RS05815	1258619	1260184		0	alpha/beta hydrolase	WP_142280593.1
G6N56_RS05820	1260235	1261626		0	wax ester/triacylglycerol synthase family O-acyltransferase	WP_085255655.1
G6N56_RS05825	1261781	1262626	panB		3-methyl-2-oxobutanoate hydroxymethyltransferase	WP_085255677.1
G6N56_RS05830	1262802	1263986		0	cellulase family glycosylhydrolase	WP_085255676.1
G6N56_RS05835	1264061	1265596		0	CYTH and CHAD domain-containing protein	WP_085255654.1

G6N56_RS05845	1266082	1266711		0	class I SAM-dependent methyltransferase	WP_085255653.1
G6N56_RS05850	1266759	1267334		0	XRE family transcriptional regulator	WP_085255652.1
G6N56_RS05855	1267409	1268575		0	cytochrome P450	WP_085255675.1
G6N56_RS05860	1268572	1268766		0	ferredoxin	WP_085255651.1
G6N56_RS28525	1268898	1269140		0	hypothetical protein	WP_180150487.1
G6N56_RS05870	1269152	1270252		0	bifunctional RNase H/acid phosphatase	WP_085255649.1
G6N56_RS05875	1270249	1270986		0	zinc ribbon domain-containing protein	WP_085255648.1
G6N56_RS05880	1270983	1272122		0	Nif3-like dinuclear metal center hexameric protein	WP_085255647.1
G6N56_RS05885	1272119	1273147	cobC		Rv2231c family pyridoxal phosphate-dependent protein CobC	WP_085255674.1
G6N56_RS05890	1273384	1274247		0	fumarylacetoacetate hydrolase family protein	WP_085255646.1
G6N56_RS05895	1274328	1274957		0	HAD-IA family hydrolase	WP_085255673.1
G6N56_RS05910	1276244	1277179		0	cobalamin biosynthesis protein	WP_085255643.1
G6N56_RS05915	1277378	1277773		0	VOC family protein	WP_085255642.1
G6N56_RS05920	1277789	1278907		0	epoxide hydrolase	WP_085255641.1
G6N56_RS05925	1279048	1279929		0	SDR family oxidoreductase	WP_085255640.1
G6N56_RS05930	1279961	1280569		0	hypothetical protein	WP_085255639.1
G6N56_RS05935	1280773	1281693		0	LysR family transcriptional regulator	WP_085255638.1
G6N56_RS05940	1281850	1282290		0	SDR family NAD(P)-dependent oxidoreductase	WP_085255637.1
G6N56_RS05945	1282290	1283135		0	SDR family NAD(P)-dependent oxidoreductase	WP_085255636.1
G6N56_RS05955	1283375	1283836		0	peroxiredoxin	WP_085255635.1
G6N56_RS05960	1283836	1284258		0	DUF3052 domain-containing protein	WP_085255634.1
G6N56_RS05965	1284340	1284921		0	hypothetical protein	WP_085255633.1
G6N56_RS05970	1285082	1287874	aceE		pyruvate dehydrogenase (acetyl-transferring), homodimeric typ	WP_085255632.1
G6N56_RS05975	1288144	1289472		0	PucR family transcriptional regulator	WP_085255631.1
G6N56_RS05980	1289610	1290518		0	ACP S-malonyltransferase	WP_085255630.1
G6N56_RS05985	1290598	1290945	acpM		meromycolate extension acyl carrier protein AcpM	WP_085255629.1
G6N56_RS05990	1290942	1292192	kasA		3-oxoacyl-ACP synthase KasA	WP_085255628.1
G6N56_RS06000	1293637	1295070		0	acyl-CoA carboxylase subunit beta	WP_085254605.1
G6N56_RS06005	1295152	1296714		0	FAD-dependent monooxygenase	WP_085254604.1
G6N56_RS06010	1296762	1298300		0	glycerol-3-phosphate dehydrogenase/oxidase	WP_085254603.1
G6N56_RS06020	1298869	1300479		0	FAD-binding oxidoreductase	WP_085254601.1

G6N56_RS06025	1300476	1301408		0	diacylglycerol kinase	WP_085254600.1
G6N56_RS06030	1301472	1301984		0	DUF3145 domain-containing protein	WP_085254599.1
G6N56_RS06035	1302123	1302947		0	serine hydrolase domain-containing protein	WP_085254598.1
G6N56_RS06040	1302944	1303963		0	class I SAM-dependent methyltransferase	WP_142280489.1
G6N56_RS06045	1304096	1305181		0	S-(hydroxymethyl)mycothiol dehydrogenase	WP_085254596.1
G6N56_RS06050	1305181	1305804		0	MBL fold metallo-hydrolase	WP_085254595.1
G6N56_RS06055	1306303	1307019		0	SHOCT domain-containing protein	WP_085254594.1
G6N56_RS06060	1307016	1308500	lnt		apolipoprotein N-acyltransferase	WP_085254593.1
G6N56_RS06065	1308547	1309500		0	SDR family oxidoreductase	WP_085254592.1
G6N56_RS06070	1309514	1310623		0	SAM-dependent methyltransferase	WP_085254591.1
G6N56_RS06075	1310697	1311095		0	hypothetical protein	WP_085254590.1
G6N56_RS06080	1311107	1312831		0	Hsp70 family protein	WP_085254589.1
G6N56_RS06085	1312935	1314011		0	dihydrodipicolinate reductase	WP_085254588.1
G6N56_RS06090	1314081	1315262		0	MFS transporter	WP_142280487.1
G6N56_RS06095	1315276	1315623		0	MmpS family transport accessory protein	WP_180150494.1
G6N56_RS06100	1315683	1316984		0	cytochrome P450	WP_085254585.1
G6N56_RS06105	1317002	1317541		0	ester cyclase	WP_085254584.1
G6N56_RS06110	1317551	1317991		0	hypothetical protein	WP_085254583.1
G6N56_RS06115	1317988	1318695		0	haloacid dehalogenase type II	WP_085254582.1
G6N56_RS06120	1318711	1319067		0	DUF202 domain-containing protein	WP_232069219.1
G6N56_RS06130	1319329	1320084		0	potassium channel family protein	WP_085254579.1
G6N56_RS06135	1320262	1320498		0	acyl carrier protein	WP_085254578.1
G6N56_RS06140	1320913	1321668		0	cutinase family protein	WP_085254577.1
G6N56_RS06150	1323439	1324197		0	Rv3717 family N-acetylmuramoyl-L-alanine amidase	WP_085258615.1
G6N56_RS06155	1324233	1325498		0	condensation domain-containing protein	WP_232069220.1
G6N56_RS06160	1325625	1326038		0	hypothetical protein	WP_085258614.1
G6N56_RS06165	1326061	1326726		0	enoyl-CoA hydratase-related protein	WP_085258613.1
G6N56_RS06170	1326723	1327364		0	TetR/AcrR family transcriptional regulator	WP_085258612.1
G6N56_RS06175	1327622	1328908		0	hypothetical protein	WP_085258643.1
G6N56_RS28860	1328955	1329908		0	chitinase	WP_232069310.1
G6N56_RS06185	1330969	1331421		0	VOC family protein	WP_085258610.1

G6N56_RS06190	1331510	1332763	0	cytochrome P450	WP_085258609.1
G6N56_RS06195	1332809	1334224	0	CehA/McbA family metallohydrolase	WP_142280864.1
G6N56_RS06200	1334355	1334963	0	TetR/AcrR family transcriptional regulator	WP_085258607.1
G6N56_RS06205	1335027	1335455	0	DUF1707 domain-containing protein	WP_085258606.1
G6N56_RS06210	1335469	1336260	0	SDR family oxidoreductase	WP_085258605.1
G6N56_RS06215	1336257	1336718	0	SRPBCC family protein	WP_085258604.1
G6N56_RS06220	1336751	1337605	0	phytanoyl-CoA dioxygenase family protein	WP_085258603.1
G6N56_RS06225	1337607	1337957	0	hypothetical protein	WP_085258602.1
G6N56_RS06230	1338051	1339430	0	FAD-binding oxidoreductase	WP_085258601.1
G6N56_RS06235	1339548	1340966	0	serine/threonine-protein kinase	WP_085258600.1
G6N56_RS06240	1341047	1342063	0	TIGR03617 family F420-dependent LLM class oxidoreductase	WP_085258599.1
G6N56_RS06245	1342078	1342623	0	carboxymuconolactone decarboxylase family protein	WP_085258598.1
G6N56_RS06250	1342639	1343181	0	carboxymuconolactone decarboxylase family protein	WP_085258597.1
G6N56_RS06255	1343181	1344059	0	dioxygenase	WP_085258596.1
G6N56_RS06260	1344075	1345037	0	zinc-binding alcohol dehydrogenase family protein	WP_085258595.1
G6N56_RS06265	1345156	1346040	0	LysR family transcriptional regulator	WP_085258594.1
G6N56_RS06270	1346057	1347070	0	sugar ABC transporter substrate-binding protein	WP_158090774.1
G6N56_RS06275	1347117	1348166	0	ABC transporter permease	WP_085258592.1
G6N56_RS06280	1348163	1349707	0	sugar ABC transporter ATP-binding protein	WP_085258591.1
G6N56_RS06285	1349704	1351146	0	amidohydrolase family protein	WP_232069221.1
G6N56_RS06290	1351143	1352015	0	alpha/beta hydrolase	WP_085258590.1
G6N56_RS06295	1352012	1352404	0	RidA family protein	WP_085258589.1
G6N56_RS06300	1352438	1353133	0	GntR family transcriptional regulator	WP_085258588.1
G6N56_RS06305	1353134	1354015	0	fumarylacetoacetate hydrolase family protein	WP_085258587.1
G6N56_RS06310	1354071	1355207	0	NAD(P)/FAD-dependent oxidoreductase	WP_085258586.1
G6N56_RS06315	1355485	1356738	0	ammonium transporter	WP_085258585.1
G6N56_RS06320	1356821	1358122	glnT	type III glutamate--ammonia ligase	WP_085258584.1
G6N56_RS06325	1358143	1359015	0	glutamine amidotransferase	WP_085258583.1
G6N56_RS06335	1359698	1361026	0	FMN-binding glutamate synthase family protein	WP_085258581.1
G6N56_RS06340	1361023	1362207	0	FAD-binding oxidoreductase	WP_085258580.1
G6N56_RS06345	1362219	1362866	0	XRE family transcriptional regulator	WP_085258579.1

G6N56_RS06350	1362885	1364096		0	M20 family metallopeptidase	WP_197746656.1
G6N56_RS06355	1364093	1364740		0	VOC family protein	WP_085258577.1
G6N56_RS06360	1364875	1366260		0	PPE family protein	WP_264020079.1
G6N56_RS06365	1366257	1367675		0	PPE family protein	WP_232069222.1
G6N56_RS06370	1368112	1368945		0	cyclase family protein	WP_197746657.1
G6N56_RS06375	1368959	1369420		0	flavin reductase family protein	WP_158090773.1
G6N56_RS06380	1369519	1371009		0	4-hydroxyphenylacetate 3-hydroxylase N-terminal domain-cont	WP_085258575.1
G6N56_RS06390	1371496	1372947		0	cytosine permease	WP_085258573.1
G6N56_RS06395	1373265	1374233		0	helix-turn-helix domain-containing protein	WP_142280863.1
G6N56_RS06400	1374363	1375121		0	hypothetical protein	WP_142280862.1
G6N56_RS06405	1375124	1376143		0	2-oxoglutarate and iron-dependent oxygenase domain-containi	WP_085258570.1
G6N56_RS06410	1376155	1377705		0	amino acid permease	WP_085258569.1
G6N56_RS28605	1377840	1378814		0	LacI family DNA-binding transcriptional regulator	WP_264020082.1
G6N56_RS06425	1379610	1380884		0	M24 family metallopeptidase	WP_085258567.1
G6N56_RS06430	1381279	1382064		0	creatininase family protein	WP_085258566.1
G6N56_RS06435	1382064	1383446		0	hypothetical protein	WP_085258565.1
G6N56_RS06440	1383511	1384086		0	TetR/AcrR family transcriptional regulator	WP_158090772.1
G6N56_RS29415	1384121	1384828		0	TetR/AcrR family transcriptional regulator	WP_085258563.1
G6N56_RS06450	1384855	1386618	atzF		allophanate hydrolase	WP_085258562.1
G6N56_RS06455	1386615	1390289	uca		urea carboxylase	WP_085258561.1
G6N56_RS06460	1390286	1390945		0	DUF1989 domain-containing protein	WP_085258560.1
G6N56_RS06465	1390942	1391817		0	urea carboxylase-associated family protein	WP_180150496.1
G6N56_RS06470	1392161	1393429		0	alpha/beta hydrolase	WP_085258559.1
G6N56_RS06475	1393449	1393883		0	protease inhibitor I42 family protein	WP_085258558.1
G6N56_RS06480	1393992	1395080		0	DUF5685 family protein	WP_085258557.1
G6N56_RS06485	1395223	1396815		0	Na+/H+ antiporter	WP_085258636.1
G6N56_RS06490	1396829	1397989		0	AAA family ATPase	WP_085258556.1
G6N56_RS06500	1398686	1400209		0	FAD-dependent monooxygenase	WP_085258635.1
G6N56_RS06505	1400235	1401917	alsS		acetolactate synthase AlsS	WP_085258554.1
G6N56_RS06510	1402001	1402720	budA		acetolactate decarboxylase	WP_232069224.1
G6N56_RS06515	1402728	1403180		0	DUF4190 domain-containing protein	WP_142280860.1

G6N56_RS06520	1403296	1404435	0	glycosyltransferase	WP_085258552.1
G6N56_RS06525	1404453	1404776	0	DUF732 domain-containing protein	WP_158090771.1
G6N56_RS06530	1404985	1405839	0	sulfurtransferase	WP_142280859.1
G6N56_RS06535	1405840	1406175	0	DUF732 domain-containing protein	WP_085258550.1
G6N56_RS06540	1406423	1407223	0	alpha/beta hydrolase	WP_085258549.1
G6N56_RS06545	1407220	1408479	0	cytochrome P450	WP_085258632.1
G6N56_RS06550	1408491	1409090	0	TetR/AcrR family transcriptional regulator C-terminal domain-c	WP_232069225.1
G6N56_RS06555	1409180	1409962	0	SDR family oxidoreductase	WP_085258548.1
G6N56_RS06560	1409959	1410693	0	hypothetical protein	WP_232069226.1
G6N56_RS06565	1410793	1411308	0	lipoprotein LpqH	WP_085258547.1
G6N56_RS06570	1411445	1412434	0	alpha/beta fold hydrolase	WP_085258546.1
G6N56_RS06575	1412571	1413773	0	MalY/PatB family protein	WP_085258545.1
G6N56_RS06580	1413919	1414824	0	haloalkane dehalogenase	WP_085258544.1
G6N56_RS06585	1414831	1415859	0	amidohydrolase family protein	WP_085258543.1
G6N56_RS06590	1415877	1416878	0	VOC family protein	WP_085258542.1
G6N56_RS06595	1416875	1417747	0	alpha/beta fold hydrolase	WP_085258541.1
G6N56_RS06600	1417744	1419372	0	bifunctional 3-(3-hydroxy-phenyl)propionate/3-hydroxycinnami	WP_085258540.1
G6N56_RS06605	1419419	1420045	0	TetR/AcrR family transcriptional regulator	WP_085258539.1
G6N56_RS06610	1420042	1420998	0	acetaldehyde dehydrogenase (acetylating)	WP_085258538.1
G6N56_RS06615	1420995	1422005	dmpG	4-hydroxy-2-oxovalerate aldolase	WP_085258537.1
G6N56_RS06620	1422055	1422504	0	hypothetical protein	WP_085258536.1
G6N56_RS06630	1422687	1423415	0	3'(2'),5'-bisphosphate nucleotidase CysQ	WP_085258535.1
G6N56_RS06635	1423606	1424160	0	hypothetical protein	WP_085258534.1
G6N56_RS06640	1424164	1425339	0	thiolase family protein	WP_085258533.1
G6N56_RS06645	1425543	1426601	0	cupin domain-containing protein	WP_085258629.1
G6N56_RS06650	1426651	1427043	0	hypothetical protein	WP_085258532.1
G6N56_RS06655	1427262	1427864	0	TIGR03086 family metal-binding protein	WP_085258531.1
G6N56_RS06660	1428129	1429028	0	DMT family transporter	WP_085258530.1
G6N56_RS06665	1429030	1429395	0	VOC family protein	WP_085258628.1
G6N56_RS06670	1429571	1430863	0	CBS domain-containing protein	WP_163645202.1
G6N56_RS06675	1430881	1432536	0	divalent metal cation transporter	WP_085258627.1

G6N56_RS06685	1432978	1433508		0	hypothetical protein	WP_142280858.1
G6N56_RS06690	1433706	1434899		0	MFS transporter	WP_197746658.1
G6N56_RS06695	1435128	1435877		0	GntR family transcriptional regulator	WP_085258526.1
G6N56_RS06700	1435879	1436094		0	hypothetical protein	WP_085258525.1
G6N56_RS06705	1436189	1438123	htpG		molecular chaperone HtpG	WP_085258524.1
G6N56_RS06710	1438228	1438848		0	TIGR03085 family metal-binding protein	WP_085258523.1
G6N56_RS06715	1438987	1439832		0	thioesterase family protein	WP_085258522.1
G6N56_RS06720	1439963	1440676		0	VIT family protein	WP_085258625.1
G6N56_RS28535	1441059	1441676		0	chitin-binding protein	WP_163645091.1
G6N56_RS06730	1441904	1442224		0	hypothetical protein	WP_085258521.1
G6N56_RS06735	1442231	1442863		0	class I SAM-dependent methyltransferase	WP_085258520.1
G6N56_RS06740	1442860	1443792		0	class I SAM-dependent methyltransferase	WP_085258519.1
G6N56_RS06750	1444329	1444751		0	nitroreductase family deazaflavin-dependent oxidoreductase	WP_085258517.1
G6N56_RS06755	1444953	1446188		0	mechanosensitive ion channel	WP_085258516.1
G6N56_RS06760	1446365	1447372		0	YhjD/YihY/BrkB family envelope integrity protein	WP_085258515.1
G6N56_RS06765	1447373	1447582		0	DUF3072 domain-containing protein	WP_085258624.1
G6N56_RS06770	1447648	1448730		0	PDR/VanB family oxidoreductase	WP_085258514.1
G6N56_RS06775	1448733	1449656		0	alpha/beta fold hydrolase	WP_085258513.1
G6N56_RS06780	1449677	1450585		0	metal-dependent hydrolase	WP_085258512.1
G6N56_RS06785	1450611	1453850		0	thioester reductase domain-containing protein	WP_085258511.1
G6N56_RS06790	1454317	1455303		0	diacylglycerol kinase family protein	WP_085258510.1
G6N56_RS06795	1455589	1456458		0	oxidoreductase	WP_085258509.1
G6N56_RS06800	1456637	1457554		0	DMT family transporter	WP_085258508.1
G6N56_RS29420	1457624	1458745		0	PE family protein	WP_085258507.1
G6N56_RS06810	1459128	1460318		0	PrsW family intramembrane metalloprotease	WP_085258506.1
G6N56_RS06815	1460315	1460977		0	hypothetical protein	WP_085258505.1
G6N56_RS06820	1461210	1461602		0	heme-binding protein	WP_085258504.1
G6N56_RS06825	1462157	1462501		0	cupin domain-containing protein	WP_085258503.1
G6N56_RS06830	1463623	1463922		0	PE family protein	WP_085258623.1
G6N56_RS29425	1464103	1465476		0	PPE family protein	WP_085258502.1
G6N56_RS28915	1466082	1466873		0	hypothetical protein	WP_232069312.1

G6N56_RS06845	1467317	1468201	0	PfkB family carbohydrate kinase	WP_232069227.1
G6N56_RS06850	1468235	1469239	0	LacI family DNA-binding transcriptional regulator	WP_142280856.1
G6N56_RS06855	1469286	1470086	0	glutamine amidotransferase	WP_085258498.1
G6N56_RS06860	1470823	1471863	0	NAD(P)-dependent alcohol dehydrogenase	WP_085258497.1
G6N56_RS06865	1471868	1472479	0	HAD-IB family phosphatase	WP_232069230.1
G6N56_RS06870	1472621	1474081	0	nicotinate phosphoribosyltransferase	WP_085258496.1
G6N56_RS06875	1474158	1475612	0	nicotinate phosphoribosyltransferase	WP_085258495.1
G6N56_RS06880	1476230	1477171	0	nucleoside hydrolase	WP_085258494.1
G6N56_RS06885	1477246	1478238	0	oxidoreductase	WP_158090770.1
G6N56_RS06890	1478258	1479211	0	ribose-phosphate pyrophosphokinase	WP_232069232.1
G6N56_RS06895	1479815	1480117	0	hypothetical protein	WP_142280855.1
G6N56_RS06900	1480175	1481611	0	cytosine permease	WP_085258489.1
G6N56_RS06905	1481608	1482300	0	HAD-IB family phosphatase	WP_232069234.1
G6N56_RS06910	1482631	1483704	0	phosphotriesterase	WP_142280854.1
G6N56_RS06915	1484187	1485626	0	nicotinate phosphoribosyltransferase	WP_085258486.1
G6N56_RS06920	1485708	1486805	0	LLM class flavin-dependent oxidoreductase	WP_085258485.1
G6N56_RS06925	1487040	1487372	0	NIPSNAP family protein	WP_085258620.1
G6N56_RS06930	1487486	1488229	0	ANTAR domain-containing protein	WP_085258484.1
G6N56_RS06935	1488226	1488594	0	hypothetical protein	WP_085258483.1
G6N56_RS06940	1488652	1491621	0	DUF4349 domain-containing protein	WP_085258482.1
G6N56_RS06945	1491618	1492235	0	MerR family transcriptional regulator	WP_085258481.1
G6N56_RS06955	1493098	1494735	0	PPE domain-containing protein	WP_232069235.1
G6N56_RS06960	1494790	1495200	0	hypothetical protein	WP_158090769.1
G6N56_RS06965	1495638	1498400	0	LuxR family transcriptional regulator	WP_232069236.1
G6N56_RS06970	1498578	1499447	0	3-hydroxyacyl-CoA dehydrogenase family protein	WP_264020637.1
G6N56_RS06975	1499470	1500204	0	acetoacetate decarboxylase	WP_085258476.1
G6N56_RS06980	1500291	1501217	0	LysR family transcriptional regulator	WP_085258475.1
G6N56_RS06985	1501333	1501899	0	MaoC family dehydratase	WP_085258474.1
G6N56_RS06990	1501899	1502762	0	CoA ester lyase	WP_085258473.1
G6N56_RS06995	1502759	1503949	0	acyl-CoA dehydrogenase family protein	WP_085258472.1
G6N56_RS07000	1503970	1505247	0	acetyl-CoA hydrolase/transferase C-terminal domain-containin	WP_232069238.1

G6N56_RS07005	1505388	1505741		0	MmpS family transport accessory protein	WP_232069313.1
G6N56_RS07010	1505750	1508572		0	RND family transporter	WP_085258470.1
G6N56_RS07015	1508698	1508976		0	sigma factor-like helix-turn-helix DNA-binding protein	WP_085258469.1
G6N56_RS07020	1508993	1509721		0	GntR family transcriptional regulator	WP_085258468.1
G6N56_RS07025	1509877	1510881		0	glycoside hydrolase family 6 protein	WP_085258467.1
G6N56_RS07030	1510926	1512197		0	fatty acid desaturase	WP_085258466.1
G6N56_RS07035	1512466	1513380		0	TPM domain-containing protein	WP_085258465.1
G6N56_RS07045	1513572	1514717		0	zinc-binding dehydrogenase	WP_085258464.1
G6N56_RS07050	1514772	1515164		0	YchJ family protein	WP_085258463.1
G6N56_RS07055	1515556	1515855		0	hypothetical protein	WP_085258462.1
G6N56_RS07060	1516234	1516758		0	hypothetical protein	WP_085258617.1
G6N56_RS07070	1517199	1517849		0	TetR/AcrR family transcriptional regulator	WP_085258461.1
G6N56_RS07075	1517976	1518710		0	SDR family oxidoreductase	WP_085258616.1
G6N56_RS07080	1518952	1519407		0	nuclear transport factor 2 family protein	WP_085258460.1
G6N56_RS07085	1519557	1520183		0	NAD(P)-binding domain-containing protein	WP_085258459.1
G6N56_RS07090	1520836	1521393		0	type IV toxin-antitoxin system AbiEi family antitoxin domain-cor	WP_085258458.1
G6N56_RS07095	1521822	1522874		0	DUF262 domain-containing protein	WP_085258457.1
G6N56_RS07100	1522903	1523547		0	hypothetical protein	WP_142280853.1
G6N56_RS07105	1523618	1524892		0	DNA methyltransferase	WP_085258456.1
G6N56_RS07125	1528052	1528693		0	site-specific integrase	WP_264019575.1
G6N56_RS07135	1529423	1529626		0	hypothetical protein	WP_142280752.1
G6N56_RS07140	1529646	1530152		0	nitroreductase family deazaflavin-dependent oxidoreductase	WP_232069239.1
G6N56_RS07145	1530172	1530999		0	carboxymuconolactone decarboxylase family protein	WP_085257420.1
G6N56_RS07150	1531040	1532413		0	TldD/PmbA family protein	WP_085257419.1
G6N56_RS07155	1532410	1533927		0	TldD/PmbA family protein	WP_085257418.1
G6N56_RS07160	1533974	1534852		0	sugar ABC transporter permease	WP_085257417.1
G6N56_RS07170	1535833	1537149		0	sugar ABC transporter substrate-binding protein	WP_085257422.1
G6N56_RS07175	1537251	1538129		0	universal stress protein	WP_085257415.1
G6N56_RS07180	1538126	1539559		0	amino acid permease	WP_085257414.1
G6N56_RS07185	1539588	1540814	rocD		ornithine--oxo-acid transaminase	WP_085257413.1
G6N56_RS07190	1540811	1541584		0	dimethylarginine dimethylaminohydrolase family protein	WP_232069315.1

G6N56_RS07195	1541782	1542228		0	Lrp/AsnC family transcriptional regulator	WP_085257411.1
G6N56_RS07200	1542225	1544027		0	sulfatase-like hydrolase/transferase	WP_085257410.1
G6N56_RS07210	1544513	1545373		0	energy-coupling factor transporter transmembrane protein EcfT	WP_085257408.1
G6N56_RS07215	1545370	1547412		0	ATP-binding cassette domain-containing protein	WP_232069240.1
G6N56_RS07220	1547520	1548017		0	MarR family transcriptional regulator	WP_085257406.1
G6N56_RS07225	1548137	1550650		0	ABC transporter permease	WP_163645093.1
G6N56_RS07235	1551831	1553771		0	acyl-CoA dehydrogenase	WP_085257907.1
G6N56_RS07245	1555505	1555972		0	LppP/LprE family lipoprotein	WP_085258833.1
G6N56_RS07250	1556298	1560467		0	bifunctional nitrate reductase/sulfite reductase flavoprotein su	WP_085258834.1
G6N56_RS07255	1560689	1560904		0	hypothetical protein	WP_142280885.1
G6N56_RS07260	1561131	1562525		0	MBL fold metallo-hydrolase	WP_085258807.1
G6N56_RS07265	1562586	1563167		0	rhodanese-like domain-containing protein	WP_085258808.1
G6N56_RS07270	1563174	1563674		0	CAP domain-containing protein	WP_085258809.1
G6N56_RS07275	1563674	1564306		0	hypothetical protein	WP_232069242.1
G6N56_RS07280	1564303	1564923		0	Mce protein	WP_085258810.1
G6N56_RS07285	1564966	1566435		0	MlaD family protein	WP_085258811.1
G6N56_RS07290	1566436	1567557		0	MCE family protein	WP_085258812.1
G6N56_RS07295	1567554	1568882		0	MCE family protein	WP_085258813.1
G6N56_RS07300	1568879	1570051		0	MCE family protein	WP_085258836.1
G6N56_RS07305	1570048	1571076		0	MCE family protein	WP_085258814.1
G6N56_RS07310	1571073	1572341		0	MCE family protein	WP_085258815.1
G6N56_RS07315	1572346	1573194		0	ABC transporter permease	WP_085258816.1
G6N56_RS07320	1573191	1573958		0	ABC transporter permease	WP_085258817.1
G6N56_RS29430	1574266	1574400		0	hypothetical protein	WP_264019947.1
G6N56_RS07325	1574550	1576562		0	family 2A encapsulin nanocompartment cargo protein cysteine	WP_142280886.1
G6N56_RS07335	1577843	1579117		0	thioester domain-containing protein	WP_085258819.1
G6N56_RS07340	1579127	1580059	cysK		cysteine synthase A	WP_085258820.1
G6N56_RS07345	1580056	1580751	cysE		serine O-acetyltransferase	WP_232069316.1
G6N56_RS07350	1581151	1581432		0	ANTAR domain-containing protein	WP_085258822.1
G6N56_RS07355	1581576	1581740		0	hypothetical protein	WP_158090777.1
G6N56_RS07360	1581737	1582855		0	LLM class flavin-dependent oxidoreductase	WP_085258823.1

G6N56_RS07365	1582852	1583970		0	LLM class flavin-dependent oxidoreductase	WP_085258824.1
G6N56_RS07370	1583967	1585352		0	NtaA/DmoA family FMN-dependent monooxygenase	WP_085258825.1
G6N56_RS07375	1585448	1587094		0	NAD-dependent malic enzyme	WP_085258826.1
G6N56_RS07380	1587139	1589811		0	FAD/NAD(P)-binding protein	WP_085258827.1
G6N56_RS28930	1590229	1590390		0	SDR family oxidoreductase	WP_232069243.1
G6N56_RS07400	1590874	1592829	dnaG		DNA primase	WP_085258829.1
G6N56_RS07405	1592857	1593471		0	superoxide dismutase family protein	WP_232069317.1
G6N56_RS07410	1593543	1594832		0	deoxyguanosinetriphosphate triphosphohydrolase	WP_085258831.1
G6N56_RS07415	1594922	1596898		0	TPM domain-containing protein	WP_085258832.1
G6N56_RS07430	1597732	1598058		0	hypothetical protein	WP_232069244.1
G6N56_RS07445	1598969	1600126		0	PPE family protein	WP_085254793.1
G6N56_RS07450	1600610	1601995		0	glycine--tRNA ligase	WP_085254724.1
G6N56_RS07455	1602219	1602653		0	metalloregulator ArsR/SmtB family transcription factor	WP_085254794.1
G6N56_RS07460	1602650	1603042		0	Fur family transcriptional regulator	WP_085254725.1
G6N56_RS07465	1603048	1603476		0	hypothetical protein	WP_085254726.1
G6N56_RS07470	1603476	1604366		0	decaprenyl diphosphate synthase	WP_085254727.1
G6N56_RS07480	1605218	1606717		0	amidase	WP_085254728.1
G6N56_RS29435	1606790	1606921		0	hypothetical protein	WP_264020585.1
G6N56_RS07490	1607374	1607574		0	DUF2795 domain-containing protein	WP_085254796.1
G6N56_RS07495	1607631	1608530	era		GTPase Era	WP_085254797.1
G6N56_RS07500	1608595	1609905		0	hemolysin family protein	WP_085254729.1
G6N56_RS07505	1609902	1610435	ybeY		rRNA maturation RNase YbeY	WP_085254730.1
G6N56_RS07510	1610476	1611525		0	PhoH family protein	WP_085254731.1
G6N56_RS07515	1611636	1612421		0	16S rRNA (uracil(1498)-N(3))-methyltransferase	WP_085254732.1
G6N56_RS07520	1612444	1613592	dnaJ		molecular chaperone DnaJ	WP_085254733.1
G6N56_RS07525	1613646	1614677	hrcA		heat-inducible transcriptional repressor HrcA	WP_085254734.1
G6N56_RS07530	1614871	1615185		0	type II toxin-antitoxin system VapB family antitoxin	WP_085254735.1
G6N56_RS07535	1615265	1615867		0	carboxymuconolactone decarboxylase family protein	WP_085254736.1
G6N56_RS07540	1615871	1616743		0	sigma-70 family RNA polymerase sigma factor	WP_085254737.1
G6N56_RS07545	1616779	1617297		0	hypothetical protein	WP_085254738.1
G6N56_RS07550	1617468	1617977		0	hypothetical protein	WP_085254739.1

G6N56_RS07560	1618324	1619628	mbtG		NADPH-dependent L-lysine N(6)-monooxygenase MbtG	WP_085254741.1
G6N56_RS07565	1619625	1623989		0	non-ribosomal peptide synthetase	WP_197746660.1
G6N56_RS07570	1623986	1629151		0	non-ribosomal peptide synthetase	WP_085254743.1
G6N56_RS07585	1633444	1637688		0	non-ribosomal peptide synthetase	WP_085254746.1
G6N56_RS07590	1637790	1639424		0	AMP-binding protein	WP_085254747.1
G6N56_RS07595	1639458	1641146		0	MFS transporter	WP_085254748.1
G6N56_RS07600	1641354	1642448		0	NADH:flavin oxidoreductase	WP_085254749.1
G6N56_RS07610	1643625	1644623		0	phosphotransferase family protein	WP_232069245.1
G6N56_RS07615	1644714	1645376		0	TetR/AcrR family transcriptional regulator	WP_085254750.1
G6N56_RS07620	1645506	1645775		0	cytochrome C oxidase subunit IV family protein	WP_232069246.1
G6N56_RS07625	1645775	1646278		0	cytochrome c oxidase subunit 3	WP_232069247.1
G6N56_RS07630	1646559	1647722		0	acyl-CoA dehydrogenase family protein	WP_085254752.1
G6N56_RS07635	1647730	1647891		0	hypothetical protein	WP_158090690.1
G6N56_RS07640	1647947	1648621		0	TetR/AcrR family transcriptional regulator	WP_085254753.1
G6N56_RS07645	1648681	1649832		0	acetyl-CoA C-acetyltransferase	WP_085254754.1
G6N56_RS07650	1649836	1650987		0	acyl-CoA dehydrogenase family protein	WP_085254755.1
G6N56_RS07655	1651094	1652683		0	FAD-dependent oxidoreductase	WP_085254756.1
G6N56_RS07660	1652751	1652942		0	ferredoxin	WP_085254757.1
G6N56_RS07665	1652942	1654342		0	aldehyde dehydrogenase family protein	WP_085254758.1
G6N56_RS07670	1654351	1655142		0	coniferyl-alcohol dehydrogenase	WP_085254759.1
G6N56_RS07675	1655154	1656422		0	cytochrome P450	WP_085254760.1
G6N56_RS07685	1656877	1657281		0	SCP2 sterol-binding domain-containing protein	WP_085254761.1
G6N56_RS07690	1657669	1658373		0	hypothetical protein	WP_085254762.1
G6N56_RS07695	1658878	1660197		0	NAD(P)/FAD-dependent oxidoreductase	WP_085254763.1
G6N56_RS07700	1660227	1661387		0	acyl-CoA dehydrogenase family protein	WP_085254764.1
G6N56_RS07705	1661391	1662602		0	enoyl-CoA hydratase-related protein	WP_085254765.1
G6N56_RS07720	1664286	1665536		0	condensation domain-containing protein	WP_158090691.1
G6N56_RS07725	1665703	1667469		0	class I adenylate-forming enzyme family protein	WP_085254769.1
G6N56_RS07730	1667466	1669106		0	class I adenylate-forming enzyme family protein	WP_085254770.1
G6N56_RS07735	1669181	1670041		0	SDR family oxidoreductase	WP_085254771.1
G6N56_RS07745	1671359	1672714		0	OB-fold domain-containing protein	WP_180150500.1

G6N56_RS07750	1672711	1673919		0	acetyl-CoA C-acetyltransferase	WP_085254773.1
G6N56_RS07755	1673916	1674173		0	hypothetical protein	WP_085254774.1
G6N56_RS07760	1674185	1674721		0	aromatic-ring-hydroxylating dioxygenase subunit beta	WP_085254775.1
G6N56_RS07765	1674729	1675997		0	aromatic ring-hydroxylating dioxygenase subunit alpha	WP_085254776.1
G6N56_RS07770	1675994	1676782		0	enoyl-CoA hydratase-related protein	WP_085254777.1
G6N56_RS07775	1676779	1677582		0	enoyl-CoA hydratase-related protein	WP_085254778.1
G6N56_RS07780	1677579	1678349		0	enoyl-CoA hydratase/isomerase family protein	WP_085254779.1
G6N56_RS28540	1678346	1678606		0	hypothetical protein	WP_085254780.1
G6N56_RS07795	1678722	1679519		0	SDR family oxidoreductase	WP_085254781.1
G6N56_RS07800	1680148	1681005		0	SDR family NAD(P)-dependent oxidoreductase	WP_085254801.1
G6N56_RS07805	1681030	1681950		0	reductase	WP_085254782.1
G6N56_RS07810	1681947	1683449		0	NAD(P)/FAD-dependent oxidoreductase	WP_085254783.1
G6N56_RS07815	1683466	1684395		0	MerR family transcriptional regulator	WP_085254784.1
G6N56_RS07820	1684400	1685416		0	acyl-ACP desaturase	WP_085254785.1
G6N56_RS07825	1685574	1686923		0	salicylate synthase	WP_085254786.1
G6N56_RS07830	1687426	1688661		0	sodium-dependent bicarbonate transport family permease	WP_085254787.1
G6N56_RS07835	1688884	1690110		0	sodium-dependent bicarbonate transport family permease	WP_085254788.1
G6N56_RS07840	1690128	1691300	hemW		radical SAM family heme chaperone HemW	WP_085254802.1
G6N56_RS07845	1691660	1693339		0	nitrite/sulfite reductase	WP_085254803.1
G6N56_RS07850	1693336	1694079		0	phosphoadenylyl-sulfate reductase	WP_085254789.1
G6N56_RS07865	1696064	1696876	cysW		sulfate ABC transporter permease subunit CysW	WP_085257957.1
G6N56_RS07870	1696873	1697739	cysT		sulfate ABC transporter permease subunit CysT	WP_085257958.1
G6N56_RS07875	1697736	1698782		0	sulfate ABC transporter substrate-binding protein	WP_085257959.1
G6N56_RS07880	1699086	1699280		0	hypothetical protein	WP_085257960.1
G6N56_RS07885	1699335	1700591		0	FAD-dependent oxidoreductase	WP_085257961.1
G6N56_RS07890	1700664	1702703		0	glycoside hydrolase family 15 protein	WP_085257962.1
G6N56_RS07895	1702886	1703653		0	sensor domain-containing protein	WP_158090767.1
G6N56_RS07900	1703844	1704605		0	sensor domain-containing protein	WP_085257964.1
G6N56_RS07905	1704608	1706470	lepA		translation elongation factor 4	WP_085257965.1
G6N56_RS07910	1706588	1707190		0	type II toxin-antitoxin system PemK/MazF family toxin	WP_085257966.1
G6N56_RS07915	1707272	1707700		0	CBS domain-containing protein	WP_085257967.1

G6N56_RS07920	1707818	1708648		0	ribonuclease Z	WP_085257968.1
G6N56_RS07930	1708879	1709718		0	transglutaminase family protein	WP_085257969.1
G6N56_RS07935	1709718	1710695		0	alpha-E domain-containing protein	WP_085257970.1
G6N56_RS07940	1710695	1712374		0	circularly permuted type 2 ATP-grasp protein	WP_085257971.1
G6N56_RS07945	1712540	1712800	rpsT		30S ribosomal protein S20	WP_085257972.1
G6N56_RS07950	1712816	1713775		0	DNA polymerase III subunit delta	WP_142280808.1
G6N56_RS07955	1713806	1715380		0	ComEC/Rec2 family competence protein	WP_085258014.1
G6N56_RS07960	1715431	1716312		0	ComEA family DNA-binding protein	WP_085257974.1
G6N56_RS07965	1716508	1717668		0	acyl-CoA dehydrogenase family protein	WP_085258015.1
G6N56_RS07970	1717676	1718791		0	acyl-CoA dehydrogenase family protein	WP_085257975.1
G6N56_RS07975	1718797	1719228		0	PPOX class F420-dependent oxidoreductase	WP_085257976.1
G6N56_RS07980	1719238	1730349		0	type I polyketide synthase	WP_085257977.1
G6N56_RS07985	1730772	1732193		0	condensation domain-containing protein	WP_085257978.1
G6N56_RS07990	1732275	1735274		0	RND family transporter	WP_085258016.1
G6N56_RS07995	1735326	1736462		0	acyltransferase PE	WP_085257979.1
G6N56_RS08000	1736512	1738269		0	AMP-binding protein	WP_085258017.1
G6N56_RS08005	1738357	1739415		0	dihydrodipicolinate reductase	WP_085257980.1
G6N56_RS08010	1739624	1740052		0	MmpS family transport accessory protein	WP_085258018.1
G6N56_RS08015	1740049	1743000		0	MMPL family transporter	WP_085257981.1
G6N56_RS08025	1743824	1744576		0	SGNH/GDSL hydrolase family protein	WP_085257983.1
G6N56_RS08035	1745255	1745650	rsfS		ribosome silencing factor	WP_085257985.1
G6N56_RS08040	1745647	1746303	nadD		nicotinate-nucleotide adenylyltransferase	WP_264019896.1
G6N56_RS08045	1746463	1747431		0	alpha/beta hydrolase	WP_232069248.1
G6N56_RS08050	1747439	1748884		0	VWA domain-containing protein	WP_085257987.1
G6N56_RS08055	1748895	1749782		0	MoxR family ATPase	WP_085257988.1
G6N56_RS08060	1749808	1751079		0	glutamate-5-semialdehyde dehydrogenase	WP_085257989.1
G6N56_RS08065	1751148	1752554		0	mechanosensitive ion channel family protein	WP_085257990.1
G6N56_RS08070	1752557	1754629		0	adenylate/guanylate cyclase domain-containing protein	WP_085258020.1
G6N56_RS08080	1755503	1756330		0	GntR family transcriptional regulator	WP_232069249.1
G6N56_RS08085	1756401	1757834		0	MmgE/PrpD family protein	WP_085257992.1
G6N56_RS08095	1758722	1759969		0	aconitase/3-isopropylmalate dehydratase large subunit family	WP_085257994.1

G6N56_RS08100	1759966	1760526		0	3-isopropylmalate dehydratase	WP_085257995.1
G6N56_RS08105	1760523	1761071		0	YbhB/YbcL family Raf kinase inhibitor-like protein	WP_085257996.1
G6N56_RS08110	1761407	1761805		0	VOC family protein	WP_085257997.1
G6N56_RS08115	1761838	1763901		0	NAD(+) synthase	WP_085257998.1
G6N56_RS08120	1763960	1764499		0	isochorismatase family cysteine hydrolase	WP_085258022.1
G6N56_RS08125	1764687	1765520		0	NAD-dependent deacetylase	WP_085257999.1
G6N56_RS08130	1765790	1767058		0	cytochrome P450	WP_085258000.1
G6N56_RS08135	1767162	1767812		0	TetR/AcrR family transcriptional regulator	WP_085258001.1
G6N56_RS08140	1767886	1768377		0	MarR family transcriptional regulator	WP_085258002.1
G6N56_RS08145	1768403	1768822		0	DUF5313 domain-containing protein	WP_085258003.1
G6N56_RS08150	1768819	1769931	proB		glutamate 5-kinase	WP_085258004.1
G6N56_RS08155	1769928	1771382	obgE		GTPase ObgE	WP_085258005.1
G6N56_RS08160	1771445	1771699	rpmA		50S ribosomal protein L27	WP_085258006.1
G6N56_RS08165	1771714	1772025	rplU		50S ribosomal protein L21	WP_085258007.1
G6N56_RS08170	1772261	1775140		0	Rne/Rng family ribonuclease	WP_085258008.1
G6N56_RS08175	1775446	1775856	ndk		nucleoside-diphosphate kinase	WP_085258009.1
G6N56_RS08180	1775886	1776272		0	DUF4233 domain-containing protein	WP_085258010.1
G6N56_RS08185	1776269	1777738		0	folylpolyglutamate synthase/dihydrofolate synthase family prot	WP_085258011.1
G6N56_RS08190	1777735	1780389		0	valine--tRNA ligase	WP_085258012.1
G6N56_RS08195	1780416	1781675		0	trans-acting enoyl reductase family protein	WP_085258013.1
G6N56_RS08200	1781769	1782389		0	transglycosylase family protein	WP_163645096.1
G6N56_RS08205	1783438	1784046		0	molybdenum cofactor guanylyltransferase	WP_085257134.1
G6N56_RS08210	1784061	1785167		0	2-oxoacid:ferredoxin oxidoreductase subunit beta	WP_085257133.1
G6N56_RS08215	1785164	1787140		0	2-oxoacid:acceptor oxidoreductase subunit alpha	WP_085257132.1
G6N56_RS08220	1787542	1788822	clpX		ATP-dependent Clp protease ATP-binding subunit ClpX	WP_085257131.1
G6N56_RS08230	1790479	1791123	clpP2		ATP-dependent CLP protease proteolytic subunit ClpP2	WP_085254966.1
G6N56_RS08235	1791120	1791710	clpP1		ATP-dependent CLP protease proteolytic subunit ClpP1	WP_085254965.1
G6N56_RS08240	1791838	1793271	tig		trigger factor	WP_085254964.1
G6N56_RS08255	1793650	1794858		0	serine hydrolase domain-containing protein	WP_085254963.1
G6N56_RS28955	1795329	1795544		0	hypothetical protein	WP_232069250.1
G6N56_RS08265	1795723	1796523		0	zinc finger domain-containing protein	WP_085254960.1

G6N56_RS08270	1796527	1797006		0	ribose-5-phosphate isomerase	WP_085254990.1
G6N56_RS08275	1797046	1797669		0	Rv2466c family mycothiol-dependent reductase	WP_085254959.1
G6N56_RS08280	1797779	1800373	pepN		aminopeptidase N	WP_085254958.1
G6N56_RS08285	1800464	1800931		0	DUF5130 domain-containing protein	WP_085254957.1
G6N56_RS08295	1801203	1801868		0	HNH endonuclease	WP_085254956.1
G6N56_RS08300	1802012	1802401		0	globin	WP_085254955.1
G6N56_RS08305	1802401	1803969		0	glycoside hydrolase family 13 protein	WP_085254987.1
G6N56_RS08310	1804042	1805058		0	pyridine nucleotide-disulfide oxidoreductase	WP_085254988.1
G6N56_RS29440	1805081	1805215		0	hypothetical protein	WP_264019728.1
G6N56_RS28960	1805458	1806006		0	amidohydrolase family protein	WP_264019727.1
G6N56_RS08320	1806021	1806686		0	hypothetical protein	WP_085254954.1
G6N56_RS08330	1807084	1811928		0	NAD-glutamate dehydrogenase	WP_085254952.1
G6N56_RS08335	1812014	1813690	ettA		energy-dependent translational throttle protein EttA	WP_085254951.1
G6N56_RS08340	1813771	1814253	ssb		single-stranded DNA-binding protein	WP_085254986.1
G6N56_RS08345	1814434	1816521		0	cytochrome c oxidase assembly protein	WP_085254950.1
G6N56_RS08350	1816551	1818905		0	glycerol-3-phosphate 1-O-acyltransferase	WP_085254949.1
G6N56_RS08355	1818902	1820584		0	HAD-IB family hydrolase	WP_085254948.1
G6N56_RS08360	1820581	1822050		0	wax ester/triacylglycerol synthase family O-acyltransferase	WP_085254947.1
G6N56_RS08365	1822203	1823444		0	alpha/beta hydrolase	WP_085254946.1
G6N56_RS08375	1823692	1823856		0	hypothetical protein	WP_158090707.1
G6N56_RS08380	1823874	1825127		0	IS256 family transposase	WP_085254945.1
G6N56_RS29445	1825336	1825563		0	nitronate monooxygenase	WP_142280515.1
G6N56_RS08395	1825929	1826774		0	helix-turn-helix transcriptional regulator	WP_085254944.1
G6N56_RS08400	1826807	1827538		0	alpha/beta fold hydrolase	WP_085254943.1
G6N56_RS08405	1827579	1828475		0	LLM class flavin-dependent oxidoreductase	WP_085254942.1
G6N56_RS08410	1828979	1829395		0	nitroreductase/quinone reductase family protein	WP_085254940.1
G6N56_RS08415	1829703	1830464		0	enoyl-CoA hydratase/isomerase family protein	WP_085254939.1
G6N56_RS08420	1830547	1832532		0	FAD-dependent oxidoreductase	WP_085254938.1
G6N56_RS08425	1832542	1832736		0	ferredoxin	WP_085254985.1
G6N56_RS08430	1832857	1834086		0	amidohydrolase family protein	WP_158090705.1
G6N56_RS08435	1834212	1835180		0	helix-turn-helix domain-containing protein	WP_158090704.1

G6N56_RS08440	1835185	1836279	0	CaiB/BaiF CoA-transferase family protein	WP_158090703.1
G6N56_RS08445	1836488	1838086	0	AMP-binding protein	WP_158090702.1
G6N56_RS08450	1838208	1838708	0	nuclear transport factor 2 family protein	WP_085254934.1
G6N56_RS08455	1838705	1839532	0	SDR family oxidoreductase	WP_085254933.1
G6N56_RS08460	1839556	1840779	0	cytochrome P450	WP_158090701.1
G6N56_RS08465	1841303	1843576	0	pyruvate dehydrogenase	WP_142280520.1
G6N56_RS08470	1843796	1844977	0	cytochrome P450	WP_085254931.1
G6N56_RS08475	1844993	1845193	0	ferredoxin	WP_085254930.1
G6N56_RS08480	1845285	1846052	0	SDR family oxidoreductase	WP_085254929.1
G6N56_RS08485	1846294	1846647	0	antibiotic biosynthesis monooxygenase	WP_142280513.1
G6N56_RS08490	1846644	1848335	0	FAD-dependent oxidoreductase	WP_085254927.1
G6N56_RS08495	1848474	1849610	0	hypothetical protein	WP_085254926.1
G6N56_RS08500	1849694	1850074	0	OB-fold domain-containing protein	WP_197746662.1
G6N56_RS08505	1850091	1851143	0	thiolase family protein	WP_232069251.1
G6N56_RS08510	1851504	1852553	0	helix-turn-helix domain-containing protein	WP_085254924.1
G6N56_RS08515	1852721	1853914	0	amidohydrolase family protein	WP_085254923.1
G6N56_RS08520	1854413	1856074	0	AMP-binding protein	WP_142280512.1
G6N56_RS08525	1856832	1857569	0	TetR/AcrR family transcriptional regulator	WP_085254921.1
G6N56_RS08530	1857644	1858114	0	nuclear transport factor 2 family protein	WP_142280511.1
G6N56_RS08535	1858268	1859371	0	homogentisate 1,2-dioxygenase	WP_085254919.1
G6N56_RS08540	1859629	1860792	0	alpha/beta fold hydrolase	WP_085254918.1
G6N56_RS08545	1861275	1862873	0	AMP-binding protein	WP_158090700.1
G6N56_RS08550	1862981	1864468	0	aldehyde dehydrogenase	WP_085254916.1
G6N56_RS08555	1864592	1865416	0	alpha/beta hydrolase family protein	WP_232069323.1
G6N56_RS08565	1865848	1866276	0	CAP domain-containing protein	WP_232069252.1
G6N56_RS08570	1866494	1867129	0	hypothetical protein	WP_232069253.1
G6N56_RS08580	1867755	1869221	0	MlaD family protein	WP_085254911.1
G6N56_RS08585	1869223	1870359	0	virulence factor Mce family protein	WP_085254910.1
G6N56_RS08590	1870356	1871717	0	MCE family protein	WP_085254909.1
G6N56_RS08595	1871719	1873032	0	MCE family protein	WP_085254908.1
G6N56_RS08600	1873029	1874057	0	MCE family protein	WP_085254907.1

G6N56_RS08605	1874085	1875566		0	MCE family protein	WP_085254906.1
G6N56_RS08610	1875570	1876376		0	ABC transporter permease	WP_085254905.1
G6N56_RS08615	1876421	1877188		0	ABC transporter permease	WP_180150505.1
G6N56_RS08620	1877799	1878575		0	TetR/AcrR family transcriptional regulator	WP_085254904.1
G6N56_RS08625	1878708	1879640		0	hydroxymethylglutaryl-CoA lyase	WP_085254903.1
G6N56_RS08630	1879637	1880848		0	CoA transferase	WP_085254902.1
G6N56_RS08635	1881200	1881868		0	VOC family protein	WP_197746663.1
G6N56_RS08640	1882013	1882381		0	DUF732 domain-containing protein	WP_085254901.1
G6N56_RS08645	1882454	1883209		0	SDR family NAD(P)-dependent oxidoreductase	WP_085254900.1
G6N56_RS08650	1883766	1884545		0	SDR family NAD(P)-dependent oxidoreductase	WP_264019722.1
G6N56_RS08655	1884662	1886992		0	acyl-CoA dehydrogenase	WP_232069324.1
G6N56_RS08660	1886989	1887729	fabG		3-oxoacyl-ACP reductase FabG	WP_085254897.1
G6N56_RS08665	1887882	1888052		0	hypothetical protein	WP_158090698.1
G6N56_RS08670	1888073	1888834		0	SDR family NAD(P)-dependent oxidoreductase	WP_085254896.1
G6N56_RS08675	1888834	1889769		0	SDR family NAD(P)-dependent oxidoreductase	WP_085254895.1
G6N56_RS08680	1889913	1891538		0	AMP-binding protein	WP_158090697.1
G6N56_RS08685	1892113	1892550		0	nuclear transport factor 2 family protein	WP_158090696.1
G6N56_RS08690	1892547	1893764		0	CaiB/BaiF CoA-transferase family protein	WP_085254892.1
G6N56_RS08695	1893806	1894924		0	NDMA-dependent alcohol dehydrogenase	WP_085254891.1
G6N56_RS08700	1895109	1896605		0	aldehyde dehydrogenase	WP_085254890.1
G6N56_RS08705	1897036	1898250		0	cytochrome P450	WP_158090695.1
G6N56_RS08710	1898282	1898473		0	ferredoxin	WP_085254888.1
G6N56_RS08715	1898570	1899652		0	MBL fold metallo-hydrolase	WP_085254887.1
G6N56_RS08720	1899664	1901364		0	FAD-dependent oxidoreductase	WP_085254886.1
G6N56_RS08730	1901964	1903145		0	flavin reductase	WP_085254884.1
G6N56_RS08735	1903199	1904554		0	amidohydrolase family protein	WP_085254883.1
G6N56_RS08740	1904574	1906199		0	AMP-binding protein	WP_142280508.1
G6N56_RS08745	1906228	1906662		0	VOC family protein	WP_142280507.1
G6N56_RS08750	1906659	1907912		0	MaoC family dehydratase N-terminal domain-containing protei	WP_085254880.1
G6N56_RS08755	1907983	1908753		0	SDR family oxidoreductase	WP_085254879.1
G6N56_RS08760	1909107	1909799		0	class I SAM-dependent methyltransferase	WP_085254878.1

G6N56_RS08765	1909847	1910593	0	alpha/beta hydrolase	WP_085254979.1
G6N56_RS08770	1910611	1910991	0	DUF4189 domain-containing protein	WP_085254877.1
G6N56_RS08775	1911120	1911368	0	hypothetical protein	WP_085254876.1
G6N56_RS08780	1911485	1912192	0	carboxymuconolactone decarboxylase family protein	WP_085254875.1
G6N56_RS08785	1912353	1913123	0	enoyl-CoA hydratase	WP_085254874.1
G6N56_RS08790	1913120	1914004	0	bile acid:sodium symporter	WP_085254873.1
G6N56_RS08795	1914288	1915100	0	CoA ester lyase	WP_085254872.1
G6N56_RS08800	1915097	1915594	0	MaoC family dehydratase	WP_085254871.1
G6N56_RS08805	1915647	1916813	0	acyl-CoA dehydrogenase family protein	WP_085254870.1
G6N56_RS08810	1916810	1918801	0	acetyl/propionyl/methylcrotonyl-CoA carboxylase subunit alpha	WP_085254978.1
G6N56_RS08815	1918807	1920378	0	carboxyl transferase domain-containing protein	WP_085254977.1
G6N56_RS08820	1920390	1920845	0	CoA-transferase	WP_085254869.1
G6N56_RS08825	1920842	1921303	0	CoA-transferase	WP_085254868.1
G6N56_RS08830	1921449	1922105	0	TetR/AcrR family transcriptional regulator	WP_085254867.1
G6N56_RS08835	1922173	1922970	0	MmpS family transport accessory protein	WP_085254976.1
G6N56_RS08840	1922974	1924299	0	MFS transporter	WP_085254866.1
G6N56_RS08845	1924570	1925376	cmrA	mycolate reductase	WP_085254865.1
G6N56_RS08850	1925378	1926961	0	DUF853 family protein	WP_085254975.1
G6N56_RS08855	1927072	1927719	orn	oligoribonuclease	WP_085254864.1
G6N56_RS08865	1927950	1928246	0	hypothetical protein	WP_085254863.1
G6N56_RS28970	1928247	1929170	0	hypothetical protein	WP_085254862.1
G6N56_RS08880	1929443	1930639	0	Ig-like domain-containing protein	WP_085254860.1
G6N56_RS08885	1930828	1931727	0	DMT family transporter	WP_085254859.1
G6N56_RS08890	1931786	1934140	0	MMPL family transporter	WP_085254858.1
G6N56_RS08895	1934416	1935654	0	GNAT family N-acetyltransferase	WP_085254857.1
G6N56_RS08905	1936015	1937757	0	DUF3556 domain-containing protein	WP_085254856.1
G6N56_RS08915	1938075	1938548	bcp	thioredoxin-dependent thiol peroxidase	WP_085254854.1
G6N56_RS08920	1938571	1939713	0	alpha/beta fold hydrolase	WP_085254853.1
G6N56_RS08925	1940035	1941369	0	dipeptidase	WP_085254852.1
G6N56_RS08930	1941483	1941875	0	holo-ACP synthase	WP_085254851.1
G6N56_RS08935	1941904	1951134	0	type I polyketide synthase	WP_085254850.1

G6N56_RS08940	1951798	1952520	0	DUF1906 domain-containing protein	WP_085254849.1
G6N56_RS08945	1952613	1953473	0	MerR family transcriptional regulator	WP_085254848.1
G6N56_RS08950	1953514	1954674	0	SRPBCC family protein	WP_085254847.1
G6N56_RS08955	1954675	1956018	0	lipocalin-like domain-containing protein	WP_085254846.1
G6N56_RS08960	1956110	1957585	0	aldehyde dehydrogenase	WP_085254845.1
G6N56_RS08965	1957586	1957975	0	MaoC family dehydratase	WP_085254974.1
G6N56_RS08970	1958023	1958508	0	MaoC family dehydratase N-terminal domain-containing protei	WP_142280519.1
G6N56_RS08975	1958591	1959016	0	nuclear transport factor 2 family protein	WP_085254843.1
G6N56_RS08980	1959106	1960680	0	AMP-binding protein	WP_085254842.1
G6N56_RS08985	1960700	1961827	0	Rieske 2Fe-2S domain-containing protein	WP_180150507.1
G6N56_RS08990	1961994	1962446	0	nuclear transport factor 2 family protein	WP_142280505.1
G6N56_RS08995	1962521	1962712	0	ferredoxin	WP_085254840.1
G6N56_RS09000	1962730	1965111	0	CoA transferase	WP_085254839.1
G6N56_RS09005	1965171	1965992	0	FCD domain-containing protein	WP_085254838.1
G6N56_RS09015	1966635	1967549	0	LLM class flavin-dependent oxidoreductase	WP_085254836.1
G6N56_RS09020	1967611	1968678	0	acyl-CoA dehydrogenase family protein	WP_085254835.1
G6N56_RS09025	1968675	1969772	0	acyl-CoA dehydrogenase family protein	WP_085254834.1
G6N56_RS09030	1969769	1970926	0	acyl-CoA dehydrogenase family protein	WP_180150509.1
G6N56_RS09035	1970923	1971858	0	acyl-CoA dehydrogenase family protein	WP_085254833.1
G6N56_RS09040	1971855	1973354	0	class I adenylate-forming enzyme family protein	WP_085254832.1
G6N56_RS09045	1973494	1974273	0	hypothetical protein	WP_269473816.1
G6N56_RS09050	1974540	1974947	0	hypothetical protein	WP_085254829.1
G6N56_RS09055	1975141	1976256	0	FAD-dependent oxidoreductase	WP_085254828.1
G6N56_RS09060	1976403	1977143	0	DUF4396 domain-containing protein	WP_232069255.1
G6N56_RS09065	1977199	1978248	0	hotdog fold thioesterase	WP_085254827.1
G6N56_RS09070	1978318	1979523	0	amidohydrolase family protein	WP_085254826.1
G6N56_RS09075	1979795	1981786	0	SpoIIE family protein phosphatase	WP_085254825.1
G6N56_RS09090	1984349	1985218	0	hypothetical protein	WP_085254970.1
G6N56_RS09095	1985299	1985868	0	hypothetical protein	WP_085254969.1
G6N56_RS09100	1985878	1986837	0	alpha/beta hydrolase	WP_085254823.1
G6N56_RS09105	1986854	1988017	0	amidohydrolase family protein	WP_085254822.1

G6N56_RS09110	1988017	1989063	0	amidohydrolase family protein	WP_085254821.1
G6N56_RS09115	1989060	1989419	0	Rieske (2Fe-2S) protein	WP_085254820.1
G6N56_RS09120	1989571	1990797	0	cytochrome P450	WP_085254819.1
G6N56_RS09125	1990794	1991948	0	Zn-dependent alcohol dehydrogenase	WP_085254818.1
G6N56_RS09130	1991945	1992736	0	SDR family oxidoreductase	WP_085254817.1
G6N56_RS09135	1992733	1993173	0	VOC family protein	WP_158090694.1
G6N56_RS09140	1993234	1993482	0	DUF1876 family protein	WP_085254815.1
G6N56_RS09145	1993605	1994432	0	SDR family NAD(P)-dependent oxidoreductase	WP_085254968.1
G6N56_RS09150	1994524	1995759	0	cytochrome P450	WP_085254814.1
G6N56_RS09155	1995756	1996778	0	LLM class F420-dependent oxidoreductase	WP_085254813.1
G6N56_RS09160	1996864	1998360	0	carboxyl transferase domain-containing protein	WP_085254812.1
G6N56_RS09165	1998444	1999505	0	TIGR03857 family LLM class F420-dependent oxidoreductase	WP_085254811.1
G6N56_RS09170	1999508	2000635	0	PHB depolymerase family esterase	WP_085254810.1
G6N56_RS09175	2000843	2002495	0	acyl-CoA synthetase	WP_085254809.1
G6N56_RS09180	2002584	2003780	0	cytochrome P450	WP_085254808.1
G6N56_RS09185	2003802	2005013	0	CoA transferase	WP_085254967.1
G6N56_RS09190	2005199	2005552	0	DUF485 domain-containing protein	WP_085254807.1
G6N56_RS09195	2005549	2007180	0	cation acetate symporter	WP_085254806.1
G6N56_RS09200	2007336	2007815	0	hypothetical protein	WP_085254805.1
G6N56_RS09205	2008304	2009941	0	LuxR family transcriptional regulator	WP_158090693.1
G6N56_RS09210	2010017	2010208	0	hypothetical protein	WP_158090692.1
G6N56_RS09220	2011264	2012187	0	AAA family ATPase	WP_142280641.1
G6N56_RS09225	2012467	2012751	0	hypothetical protein	WP_085256170.1
G6N56_RS09230	2013140	2013586	0	hypothetical protein	WP_142280640.1
G6N56_RS09235	2013666	2014367	0	hypothetical protein	WP_085256168.1
G6N56_RS09240	2014900	2015721	0	hypothetical protein	WP_142280639.1
G6N56_RS09245	2015769	2016341	0	hypothetical protein	WP_142280638.1
G6N56_RS09250	2016539	2019313	0	LuxR family transcriptional regulator	WP_142280637.1
G6N56_RS09255	2019396	2019677	0	hypothetical protein	WP_085256165.1
G6N56_RS09260	2020010	2020906	0	alpha/beta hydrolase	WP_264020123.1
G6N56_RS09265	2021444	2022595	0	hypothetical protein	WP_085256163.1

G6N56_RS09270	2022592	2023560		0	hypothetical protein	WP_085256162.1
G6N56_RS09275	2023563	2023823		0	acyl carrier protein	WP_232069256.1
G6N56_RS09280	2023820	2026765		0	type I polyketide synthase	WP_085256160.1
G6N56_RS09285	2026794	2028440		0	long-chain-fatty-acid--CoA ligase	WP_085256159.1
G6N56_RS09290	2028506	2029588		0	methyltransferase	WP_085256158.1
G6N56_RS09295	2029660	2030118		0	hypothetical protein	WP_085256157.1
G6N56_RS09300	2030115	2031896		0	FAD-dependent oxidoreductase	WP_085256156.1
G6N56_RS09305	2032045	2032407		0	DoxX family protein	WP_085256174.1
G6N56_RS09310	2032679	2033878		0	acyl-CoA dehydrogenase family protein	WP_085256155.1
G6N56_RS09315	2033893	2034951		0	acyl-CoA dehydrogenase family protein	WP_085256154.1
G6N56_RS09320	2034954	2035784		0	amidohydrolase family protein	WP_085256153.1
G6N56_RS09325	2035781	2037397		0	acyl-CoA synthetase	WP_085256152.1
G6N56_RS09330	2037409	2038611		0	amidohydrolase family protein	WP_085256173.1
G6N56_RS09335	2038641	2039483		0	enoyl-CoA hydratase	WP_085256172.1
G6N56_RS09340	2039640	2039912		0	GlsB/YeaQ/YmgE family stress response membrane protein	WP_085256151.1
G6N56_RS09345	2040006	2041013		0	dihydrodipicolinate synthase family protein	WP_085256150.1
G6N56_RS09350	2041059	2041856		0	SDR family oxidoreductase	WP_085256149.1
G6N56_RS09355	2042101	2042901		0	TetR/AcrR family transcriptional regulator C-terminal domain-c	WP_085256148.1
G6N56_RS09360	2042941	2043828		0	enoyl-CoA hydratase/isomerase family protein	WP_085256147.1
G6N56_RS09365	2043825	2044517		0	hypothetical protein	WP_163645099.1
G6N56_RS09370	2044648	2045469		0	enoyl-CoA hydratase-related protein	WP_085255417.1
G6N56_RS09375	2045466	2046278		0	enoyl-CoA hydratase/isomerase family protein	WP_085255418.1
G6N56_RS09390	2048525	2050105		0	class I adenylate-forming enzyme family protein	WP_085255421.1
G6N56_RS09400	2051624	2051983		0	hypothetical protein	WP_085255423.1
G6N56_RS09405	2052040	2052726		0	GntR family transcriptional regulator	WP_085255424.1
G6N56_RS09410	2052731	2053924		0	acyl-CoA dehydrogenase family protein	WP_085255425.1
G6N56_RS09415	2053921	2055150		0	CoA transferase	WP_085255426.1
G6N56_RS09420	2055147	2056364		0	acetyl-CoA C-acetyltransferase	WP_085255522.1
G6N56_RS09425	2056376	2057116	fabG		3-oxoacyl-ACP reductase FabG	WP_085255427.1
G6N56_RS09430	2057260	2058435		0	acyl-CoA dehydrogenase family protein	WP_085255428.1
G6N56_RS09435	2058512	2058940		0	pyridoxamine 5'-phosphate oxidase family protein	WP_085255429.1

G6N56_RS09440	2058954	2060027		0	phosphotransferase	WP_085255523.1
G6N56_RS09445	2060114	2061850		0	ABC transporter ATP-binding protein	WP_085255430.1
G6N56_RS09450	2061847	2064438		0	ATP-binding cassette domain-containing protein	WP_085255431.1
G6N56_RS09455	2064438	2065151		0	TetR family transcriptional regulator	WP_085255432.1
G6N56_RS09460	2065261	2065683		0	DCC1-like thiol-disulfide oxidoreductase family protein	WP_085255433.1
G6N56_RS09465	2065699	2066007		0	hypothetical protein	WP_085255434.1
G6N56_RS09475	2066550	2066930		0	hypothetical protein	WP_085255524.1
G6N56_RS09480	2066927	2067268		0	DUF3817 domain-containing protein	WP_085255435.1
G6N56_RS09485	2067374	2067976		0	non-canonical purine NTP pyrophosphatase	WP_085255436.1
G6N56_RS09490	2068237	2069016	rph		ribonuclease PH	WP_085255437.1
G6N56_RS09495	2069051	2069833		0	MBL fold metallo-hydrolase	WP_085255438.1
G6N56_RS09500	2069985	2070800	murI		glutamate racemase	WP_085255439.1
G6N56_RS09505	2070797	2071465		0	rhomboid family intramembrane serine protease	WP_085255525.1
G6N56_RS09510	2071705	2072598		0	methyltransferase	WP_085255440.1
G6N56_RS09515	2072630	2073655		0	P1 family peptidase	WP_085255441.1
G6N56_RS09520	2073652	2074245		0	DUF2017 domain-containing protein	WP_085255442.1
G6N56_RS09525	2074271	2074576	clpS		ATP-dependent Clp protease adapter ClpS	WP_085255526.1
G6N56_RS09530	2074632	2075939		0	nicotinate phosphoribosyltransferase	WP_085255443.1
G6N56_RS09535	2075959	2077968		0	ATP-dependent DNA helicase	WP_232069257.1
G6N56_RS09540	2077974	2080580		0	glycosyltransferase family 1 protein	WP_085255444.1
G6N56_RS09545	2080800	2082917		0	alpha-1,4-glucan--maltose-1-phosphate maltosyltransferase	WP_211287383.1
G6N56_RS09550	2082919	2085114	glgB		1,4-alpha-glucan branching protein GlgB	WP_085255446.1
G6N56_RS09555	2085122	2085919		0	MBL fold metallo-hydrolase	WP_085255447.1
G6N56_RS09560	2086084	2086710		0	4-carboxymuconolactone decarboxylase	WP_232069258.1
G6N56_RS09565	2087074	2087985		0	tetratricopeptide repeat protein	WP_085255449.1
G6N56_RS09570	2088154	2089335		0	acetyl-CoA C-acetyltransferase	WP_085255450.1
G6N56_RS09575	2089415	2089882	mce		methylmalonyl-CoA epimerase	WP_163645100.1
G6N56_RS28975	2089892	2090188		0	hypothetical protein	WP_085255528.1
G6N56_RS09585	2090214	2090885	nucS		endonuclease NucS	WP_085255452.1
G6N56_RS09590	2090931	2092553		0	adenylate/guanylate cyclase domain-containing protein	WP_085255453.1
G6N56_RS09600	2093062	2093436		0	hypothetical protein	WP_142280566.1

G6N56_RS09605	2093455	2094672		0	MFS transporter	WP_085255454.1
G6N56_RS09610	2094763	2095638		0	LysR family transcriptional regulator	WP_085255529.1
G6N56_RS09615	2095707	2097194		0	DNA-3-methyladenine glycosylase 2 family protein	WP_085255455.1
G6N56_RS09620	2097191	2097694		0	methylated-DNA--[protein]-cysteine S-methyltransferase	WP_085255456.1
G6N56_RS09640	2103196	2104449	murA		UDP-N-acetylglucosamine 1-carboxyvinyltransferase	WP_085255457.1
G6N56_RS09645	2104520	2105116		0	cob(I)yrinic acid a,c-diamide adenosyltransferase	WP_085255530.1
G6N56_RS09650	2105268	2105711		0	DUF2550 domain-containing protein	WP_085255458.1
G6N56_RS09655	2105718	2106083		0	F0F1 ATP synthase subunit epsilon	WP_085255459.1
G6N56_RS09660	2106108	2107574	atpD		F0F1 ATP synthase subunit beta	WP_085255460.1
G6N56_RS09665	2107596	2108510		0	F0F1 ATP synthase subunit gamma	WP_085255461.1
G6N56_RS09670	2108517	2110199	atpA		F0F1 ATP synthase subunit alpha	WP_085255462.1
G6N56_RS09675	2110278	2111618		0	F0F1 ATP synthase subunit B/delta	WP_085255463.1
G6N56_RS09680	2111626	2112156		0	F0F1 ATP synthase subunit B	WP_085255464.1
G6N56_RS09685	2112166	2112411		0	F0F1 ATP synthase subunit C	WP_071509226.1
G6N56_RS09690	2112498	2113253	atpB		F0F1 ATP synthase subunit A	WP_085255465.1
G6N56_RS09700	2113974	2115206		0	MraY family glycosyltransferase	WP_085255467.1
G6N56_RS09705	2115222	2115884		0	L-threonylcarbamoyladenylate synthase	WP_085255468.1
G6N56_RS09710	2115881	2116723	prmC		peptide chain release factor N(5)-glutamine methyltransferase	WP_085255469.1
G6N56_RS09715	2116720	2117793	prfA		peptide chain release factor 1	WP_085255470.1
G6N56_RS09720	2117890	2118117	rpmE		50S ribosomal protein L31	WP_085255531.1
G6N56_RS09725	2118307	2120142	rho		transcription termination factor Rho	WP_085255471.1
G6N56_RS09730	2120411	2121361	thrB		homoserine kinase	WP_142280568.1
G6N56_RS09735	2121370	2122452	thrC		threonine synthase	WP_085255472.1
G6N56_RS09745	2123764	2125191	lysA		diaminopimelate decarboxylase	WP_085255474.1
G6N56_RS09750	2125188	2126840	argS		arginine--tRNA ligase	WP_085255475.1
G6N56_RS09760	2127125	2127763		0	hypothetical protein	WP_085255476.1
G6N56_RS09765	2127773	2128963		0	tyrosine-type recombinase/integrase	WP_085255477.1
G6N56_RS09770	2128960	2129460		0	helix-turn-helix transcriptional regulator	WP_085255478.1
G6N56_RS09775	2129557	2129748		0	helix-turn-helix domain-containing protein	WP_085255479.1
G6N56_RS09780	2129745	2130512		0	hypothetical protein	WP_142280569.1
G6N56_RS09785	2130509	2130793		0	hypothetical protein	WP_085255480.1

G6N56_RS09790	2130790	2131467	0	hypothetical protein	WP_142280570.1
G6N56_RS09800	2132092	2132673	0	hypothetical protein	WP_085255483.1
G6N56_RS09805	2132691	2133074	0	DUF2190 family protein	WP_085255484.1
G6N56_RS09810	2133097	2133993	0	hypothetical protein	WP_085255485.1
G6N56_RS09815	2134013	2134492	0	hypothetical protein	WP_085255486.1
G6N56_RS09820	2134524	2135012	0	hypothetical protein	WP_085255487.1
G6N56_RS09825	2135153	2135407	0	hypothetical protein	WP_085255488.1
G6N56_RS09830	2135689	2136234	0	hypothetical protein	WP_085255489.1
G6N56_RS09835	2136253	2136993	0	adenylate/guanylate cyclase domain-containing protein	WP_158090715.1
G6N56_RS09845	2138051	2139316	0	low temperature requirement protein A	WP_085255492.1
G6N56_RS09850	2139570	2139935	0	hypothetical protein	WP_085255493.1
G6N56_RS09855	2140011	2140436	0	hypothetical protein	WP_085255494.1
G6N56_RS09860	2140691	2141026	0	DUF732 domain-containing protein	WP_085255495.1
G6N56_RS09865	2141187	2141723	0	NUDIX hydrolase	WP_085255496.1
G6N56_RS09870	2141784	2142155	0	nuclear transport factor 2 family protein	WP_085255497.1
G6N56_RS28980	2142347	2142799	0	transporter substrate-binding domain-containing protein	WP_232069259.1
G6N56_RS09880	2143032	2144756	0	NAD-binding protein	WP_085255498.1
G6N56_RS09885	2144759	2145568	0	hypothetical protein	WP_085255499.1
G6N56_RS09890	2145610	2146488	0	LLM class flavin-dependent oxidoreductase	WP_085255500.1
G6N56_RS09895	2146548	2146961	0	VOC family protein	WP_085255501.1
G6N56_RS09900	2147068	2147457	0	VOC family protein	WP_085255502.1
G6N56_RS09905	2147454	2147939	0	RrF2 family transcriptional regulator	WP_085255503.1
G6N56_RS09910	2147985	2149826	cysC	adenylyl-sulfate kinase	WP_085255504.1
G6N56_RS09920	2150988	2151479	0	carbonic anhydrase	WP_085255506.1
G6N56_RS09925	2151667	2152644	0	ABC transporter permease	WP_085255507.1
G6N56_RS09930	2152671	2153534	0	ABC transporter permease	WP_142280576.1
G6N56_RS09935	2153531	2155369	0	ABC transporter ATP-binding protein	WP_085255509.1
G6N56_RS09940	2155473	2157137	0	ABC transporter family substrate-binding protein	WP_085255510.1
G6N56_RS09950	2158448	2161063	0	AAA family ATPase	WP_085255512.1
G6N56_RS09955	2161060	2162205	0	exonuclease SbcCD subunit D	WP_085255513.1
G6N56_RS09960	2162323	2163771	0	PE family protein	WP_085255514.1

G6N56_RS09965	2163928	2164428	0	histidine phosphatase family protein	WP_085255515.1
G6N56_RS09970	2164432	2164992	0	DUF3558 domain-containing protein	WP_085255516.1
G6N56_RS09975	2164989	2165546	0	DUF3558 domain-containing protein	WP_085255517.1
G6N56_RS09980	2165708	2167474	0	ABC transporter ATP-binding protein	WP_085255533.1
G6N56_RS09985	2167471	2169411	0	ABC transporter ATP-binding protein	WP_085255518.1
G6N56_RS09990	2169656	2170369	0	C39 family peptidase	WP_085255519.1
G6N56_RS09995	2170606	2171751	0	BTAD domain-containing putative transcriptional regulator	WP_085255534.1
G6N56_RS10000	2171952	2173691	0	serine/threonine-protein kinase	WP_085255520.1
G6N56_RS10020	2176636	2177220	0	hypothetical protein	WP_085254110.1
G6N56_RS10025	2177884	2179020	0	adenylate/guanylate cyclase domain-containing protein	WP_163645101.1
G6N56_RS10030	2179094	2180485	0	amidase	WP_085254111.1
G6N56_RS10035	2180559	2180993	0	HIT family protein	WP_085254112.1
G6N56_RS10040	2181057	2182088	0	LLM class flavin-dependent oxidoreductase	WP_085254125.1
G6N56_RS10045	2182202	2183332	0	FAD-binding protein	WP_085254113.1
G6N56_RS10050	2183329	2184141	0	uracil-DNA glycosylase	WP_085254114.1
G6N56_RS10055	2184229	2185458	0	MFS transporter	WP_085254115.1
G6N56_RS10060	2185468	2186835	0	FAD-linked oxidase C-terminal domain-containing protein	WP_085254116.1
G6N56_RS10065	2187034	2188275	0	cytochrome P450	WP_163645102.1
G6N56_RS10070	2188272	2188853	0	TetR/AcrR family transcriptional regulator	WP_142280443.1
G6N56_RS10075	2188885	2190024	0	acyltransferase	WP_085254119.1
G6N56_RS10080	2190021	2191733	0	DEAD/DEAH box helicase	WP_085254120.1
G6N56_RS10085	2191827	2192432	0	LppP/LprE family lipoprotein	WP_085254121.1
G6N56_RS10095	2193188	2193997	0	sulfotransferase	WP_085254122.1
G6N56_RS29450	2197060	2199600	0	PE family protein	WP_163645103.1
G6N56_RS10110	2200400	2203825	0	TM0106 family RecB-like putative nuclease	WP_163645104.1
G6N56_RS10120	2205927	2206691	0	hypothetical protein	WP_085254530.1
G6N56_RS10125	2206895	2210611	0	multifunctional oxoglutarate decarboxylase/oxoglutarate dehyc	WP_085254529.1
G6N56_RS10130	2210691	2211521	0	SDR family oxidoreductase	WP_085254528.1
G6N56_RS10135	2211651	2212499	0	glycine betaine ABC transporter substrate-binding protein	WP_085254527.1
G6N56_RS10140	2212496	2213662	0	NADP-dependent malic enzyme	WP_232069264.1
G6N56_RS10145	2213703	2214692	0	malate dehydrogenase	WP_085254526.1

G6N56_RS10150	2214898	2216010	corA		magnesium/cobalt transporter CorA	WP_085254535.1
G6N56_RS10160	2216543	2217136		0	suppressor of fused domain protein	WP_085254525.1
G6N56_RS10165	2217136	2218302	ugpC		sn-glycerol-3-phosphate ABC transporter ATP-binding protein U	WP_085254524.1
G6N56_RS10170	2218306	2219130		0	carbohydrate ABC transporter permease	WP_142280481.1
G6N56_RS10175	2219135	2220064		0	sugar ABC transporter permease	WP_232069326.1
G6N56_RS10180	2220073	2221428		0	extracellular solute-binding protein	WP_232069327.1
G6N56_RS10185	2221507	2222028		0	DUF4199 domain-containing protein	WP_085254522.1
G6N56_RS10190	2222177	2222725		0	DUF4190 domain-containing protein	WP_232069265.1
G6N56_RS10195	2222781	2224088		0	CBS domain-containing protein	WP_085258170.1
G6N56_RS10200	2224085	2224621		0	DUF1003 domain-containing protein	WP_085258171.1
G6N56_RS10205	2224740	2225996		0	lytic murein transglycosylase	WP_085258172.1
G6N56_RS10210	2226316	2227473		0	P-loop NTPase	WP_142280821.1
G6N56_RS10215	2227551	2227946	tatB		Sec-independent protein translocase protein TatB	WP_085258173.1
G6N56_RS10220	2227947	2229482		0	trypsin-like peptidase domain-containing protein	WP_085258174.1
G6N56_RS10225	2229646	2230053		0	anti-sigma E factor RseA	WP_142280822.1
G6N56_RS10230	2230182	2230961	sigE		RNA polymerase sigma factor SigE	WP_085258175.1
G6N56_RS10235	2231160	2231828		0	O-methyltransferase	WP_085258176.1
G6N56_RS10245	2232636	2233559		0	ABC transporter ATP-binding protein	WP_085258178.1
G6N56_RS10250	2233565	2235190		0	ABC transporter permease	WP_269473821.1
G6N56_RS10255	2235199	2235873		0	isoprenylcysteine carboxylmethyltransferase family protein	WP_085258179.1
G6N56_RS10260	2235913	2237127	glgC		glucose-1-phosphate adenylyltransferase	WP_085258180.1
G6N56_RS10265	2237286	2238449	glgA		glycogen synthase	WP_085258181.1
G6N56_RS10270	2238469	2238642		0	DUF3117 domain-containing protein	WP_066995255.1
G6N56_RS10275	2238962	2239540	tag		DNA-3-methyladenine glycosylase I	WP_085258183.1
G6N56_RS10280	2239537	2239896		0	DivIVA domain-containing protein	WP_085258184.1
G6N56_RS10285	2239935	2240909		0	glucosyl-3-phosphoglycerate synthase	WP_085258185.1
G6N56_RS10290	2240906	2241802	folP		dihydropteroate synthase	WP_142280823.1
G6N56_RS10295	2241985	2243769	fadD6		long-chain-acyl-CoA synthetase FadD6	WP_085258187.1
G6N56_RS10300	2243850	2244413		0	TIGR00730 family Rossman fold protein	WP_085258188.1
G6N56_RS10305	2244474	2246603		0	AAA family ATPase	WP_169717553.1
G6N56_RS10310	2246648	2247391		0	hypothetical protein	WP_142280824.1

G6N56_RS10315	2247408	2248472	dapE		succinyl-diaminopimelate desuccinylase	WP_085258190.1
G6N56_RS10325	2249524	2250816		0	MFS transporter	WP_169717554.1
G6N56_RS10330	2250980	2252398		0	acyl-CoA synthetase	WP_085258193.1
G6N56_RS10335	2252408	2253313		0	alpha/beta hydrolase	WP_085258194.1
G6N56_RS10340	2253440	2254909		0	aldehyde dehydrogenase family protein	WP_085258195.1
G6N56_RS10345	2255129	2256295		0	cytochrome P450	WP_180150559.1
G6N56_RS10350	2256312	2256860		0	MaoC family dehydratase N-terminal domain-containing protei	WP_085258196.1
G6N56_RS10355	2256860	2257270		0	Zn-ribbon domain-containing OB-fold protein	WP_085258197.1
G6N56_RS10360	2257267	2257728		0	MaoC family dehydratase	WP_085258198.1
G6N56_RS10365	2257725	2258894		0	lipid-transfer protein	WP_085258199.1
G6N56_RS10370	2258917	2259510		0	TetR/AcrR family transcriptional regulator	WP_085258233.1
G6N56_RS10375	2259552	2260556		0	alpha/beta hydrolase	WP_085258234.1
G6N56_RS10380	2260896	2261849		0	proline dehydrogenase family protein	WP_085258200.1
G6N56_RS10385	2261849	2263480	pruA		L-glutamate gamma-semialdehyde dehydrogenase	WP_085258201.1
G6N56_RS10390	2263564	2265159		0	helix-turn-helix domain-containing protein	WP_142280826.1
G6N56_RS10395	2265256	2265588		0	hypothetical protein	WP_085258203.1
G6N56_RS10400	2265814	2266617		0	Stf0 family sulphotransferase	WP_085258235.1
G6N56_RS10405	2266614	2267537	cysD		sulfate adenylyltransferase subunit CysD	WP_085258204.1
G6N56_RS10410	2267537	2269462	cysC		adenylyl-sulfate kinase	WP_085258205.1
G6N56_RS29455	2269518	2269652		0	hypothetical protein	WP_264020700.1
G6N56_RS10415	2269657	2270775		0	YeiH family protein	WP_085258206.1
G6N56_RS10420	2270845	2272584	fadD21		fatty-acid--AMP ligase FAAL21/FadD21	WP_085258207.1
G6N56_RS10425	2272614	2273264		0	GAP family protein	WP_085258208.1
G6N56_RS10430	2273420	2274496		0	PE-PPE domain-containing protein	WP_085258209.1
G6N56_RS10435	2274661	2274867		0	hypothetical protein	WP_232069266.1
G6N56_RS10440	2275123	2278152		0	MMPL family transporter	WP_264020701.1
G6N56_RS10445	2278171	2279586		0	condensation domain-containing protein	WP_085258211.1
G6N56_RS10450	2279639	2285971	pks2		type I polyketide synthase	WP_085258212.1
G6N56_RS10455	2286709	2289072		0	APC family permease	WP_085258236.1
G6N56_RS10460	2289174	2290259	dapC		succinyldiaminopimelate transaminase	WP_142280827.1
G6N56_RS10465	2290307	2290633		0	ferredoxin family protein	WP_085099779.1

G6N56_RS10470	2291149	2291481		0	hemophore-related protein	WP_085258214.1
G6N56_RS10475	2291490	2291981		0	hypothetical protein	WP_085258215.1
G6N56_RS10480	2292096	2294666		0	bifunctional FO biosynthesis protein CofGH	WP_085258216.1
G6N56_RS10485	2294926	2295825		0	PE family protein	WP_085258217.1
G6N56_RS10490	2295940	2296398		0	hypothetical protein	WP_085258218.1
G6N56_RS10495	2296500	2297402	mshB		N-acetyl-1-D-myo-inositol-2-amino-2-deoxy-alpha- D-glucopyra	WP_085258219.1
G6N56_RS10500	2297495	2298106		0	TetR/AcrR family transcriptional regulator	WP_085258220.1
G6N56_RS29460	2298119	2300470		0	PE family protein	WP_163645106.1
G6N56_RS10510	2300745	2302640		0	ABC transporter family substrate-binding protein	WP_163645205.1
G6N56_RS10515	2302686	2304575	typA		translational GTPase TypA	WP_085258223.1
G6N56_RS10525	2304876	2305268		0	(deoxy)nucleoside triphosphate pyrophosphohydrolase	WP_085258224.1
G6N56_RS10530	2305295	2305579		0	4a-hydroxytetrahydrobiopterin dehydratase	WP_085258225.1
G6N56_RS10535	2305715	2306920		0	mannosyltransferase	WP_085258226.1
G6N56_RS10540	2307112	2307789		0	hypothetical protein	WP_163645107.1
G6N56_RS10545	2307836	2308852		0	hypothetical protein	WP_085253956.1
G6N56_RS10550	2308908	2309054		0	DUF1059 domain-containing protein	WP_085253955.1
G6N56_RS10555	2309051	2309716		0	HhH-GPD-type base excision DNA repair protein	WP_085253969.1
G6N56_RS10560	2310100	2310537		0	nitroreductase family deazaflavin-dependent oxidoreductase	WP_085253954.1
G6N56_RS10565	2310753	2310938		0	DUF5302 domain-containing protein	WP_085253953.1
G6N56_RS10570	2310955	2311398		0	PPOX class F420-dependent oxidoreductase	WP_085253952.1
G6N56_RS10575	2311455	2311976		0	DUF1697 domain-containing protein	WP_085253951.1
G6N56_RS10580	2311973	2312791		0	class I SAM-dependent methyltransferase	WP_085253950.1
G6N56_RS10585	2312788	2313156		0	GntR family transcriptional regulator	WP_085253949.1
G6N56_RS10590	2313194	2313907		0	NAD-dependent deacylase	WP_085253948.1
G6N56_RS10595	2314005	2314646		0	class I SAM-dependent methyltransferase	WP_085253947.1
G6N56_RS10600	2314643	2315326		0	TetR/AcrR family transcriptional regulator	WP_085253968.1
G6N56_RS10605	2315343	2317676		0	MMPL family transporter	WP_085253967.1
G6N56_RS29465	2317981	2318112		0	hypothetical protein	WP_142280419.1
G6N56_RS10610	2318247	2318999		0	3-hydroxyacyl-CoA dehydrogenase	WP_085253946.1
G6N56_RS10615	2319039	2320121		0	CaiB/BaiF CoA-transferase family protein	WP_085253966.1
G6N56_RS10620	2320213	2321025		0	enoyl-CoA hydratase	WP_085253945.1

G6N56_RS10625	2321111	2322448		0	SDR family oxidoreductase	WP_085253944.1
G6N56_RS10630	2322455	2323177		0	type II CAAX endopeptidase family protein	WP_085253965.1
G6N56_RS10635	2323174	2324319		0	NAD(P)H-binding protein	WP_085253943.1
G6N56_RS10640	2324832	2325332		0	isoprenylcysteine carboxyl methyltransferase family protein	WP_169717509.1
G6N56_RS10645	2325329	2326345		0	NAD(P)/FAD-dependent oxidoreductase	WP_085253941.1
G6N56_RS10650	2326423	2327226		0	crotonase/enoyl-CoA hydratase family protein	WP_085253940.1
G6N56_RS10655	2327223	2328371		0	thiolase family protein	WP_085253939.1
G6N56_RS10660	2328636	2330120		0	aldehyde dehydrogenase family protein	WP_085253938.1
G6N56_RS10665	2330212	2331426		0	cytochrome P450	WP_085253937.1
G6N56_RS10675	2332396	2333520		0	spirocyclase AveC family protein	WP_085253935.1
G6N56_RS10680	2333524	2334642		0	ABC transporter substrate-binding protein	WP_085253934.1
G6N56_RS10685	2334725	2335531		0	CbbQ/NirQ/NorQ/GpvN family protein	WP_142280418.1
G6N56_RS10690	2335528	2337249		0	VWA domain-containing protein	WP_180150515.1
G6N56_RS10695	2337367	2338338		0	SDR family NAD(P)-dependent oxidoreductase	WP_085253962.1
G6N56_RS10700	2338437	2339597		0	acyl-CoA dehydrogenase family protein	WP_085253933.1
G6N56_RS10705	2339594	2340703		0	acyl-CoA dehydrogenase family protein	WP_085253932.1
G6N56_RS10710	2340853	2342139		0	TetR/AcrR family transcriptional regulator	WP_085253931.1
G6N56_RS10715	2342217	2343059		0	SDR family oxidoreductase	WP_085253961.1
G6N56_RS10720	2343171	2344370		0	cytochrome P450	WP_085253930.1
G6N56_RS10725	2344418	2346046		0	GMC family oxidoreductase N-terminal domain-containing prot	WP_085253929.1
G6N56_RS10730	2346264	2347265		0	AraC family transcriptional regulator	WP_158090664.1
G6N56_RS29015	2347270	2348562		0	haloalkane dehalogenase	WP_158090663.1
G6N56_RS29020	2348604	2348915		0	helix-turn-helix domain-containing protein	WP_232069267.1
G6N56_RS29025	2349010	2349237		0	hypothetical protein	WP_232069268.1
G6N56_RS10745	2349577	2350626		0	aldo/keto reductase	WP_085253926.1
G6N56_RS10750	2351256	2351816		0	TetR/AcrR family transcriptional regulator	WP_085253960.1
G6N56_RS10755	2351846	2352487		0	SDR family oxidoreductase	WP_085253925.1
G6N56_RS10760	2353036	2355306	metE		5-methyltetrahydropteroyltriglutamate-- homocysteine S-methy	WP_085253924.1
G6N56_RS10765	2355375	2357099		0	DUF3556 domain-containing protein	WP_085253923.1
G6N56_RS10770	2357218	2357433		0	hypothetical protein	WP_085253922.1
G6N56_RS10780	2360838	2361965		0	bifunctional 2-methylcitrate synthase/citrate synthase	WP_085253920.1

G6N56_RS10785	2361962	2362879	prpB		methylisocitrate lyase	WP_085253919.1
G6N56_RS10790	2362876	2364384		0	MmgE/PrpD family protein	WP_085253918.1
G6N56_RS10795	2364495	2365940		0	short-chain fatty acyl-CoA regulator family protein	WP_142280417.1
G6N56_RS10800	2366009	2367373		0	FAD-dependent oxidoreductase	WP_085253959.1
G6N56_RS10805	2367387	2367896		0	DoxX family protein	WP_085253916.1
G6N56_RS10810	2368031	2369653		0	pyruvate, phosphate dikinase	WP_085253915.1
G6N56_RS10815	2369650	2370264		0	MarR family transcriptional regulator	WP_085253914.1
G6N56_RS10820	2370266	2371492		0	DUF1298 domain-containing protein	WP_085253958.1
G6N56_RS10825	2371522	2372430		0	alpha/beta fold hydrolase	WP_085253913.1
G6N56_RS10830	2372617	2373099		0	VOC family protein	WP_085253912.1
G6N56_RS10835	2373263	2374126		0	alpha/beta hydrolase	WP_085253911.1
G6N56_RS10840	2374142	2375164	gnd		decarboxylating 6-phosphogluconate dehydrogenase	WP_085253910.1
G6N56_RS10845	2375189	2376577		0	glucose-6-phosphate dehydrogenase	WP_085253909.1
G6N56_RS10850	2376700	2377533		0	adenylate/guanylate cyclase domain-containing protein	WP_085253908.1
G6N56_RS10860	2377842	2378522		0	guanylate cyclase	WP_085253906.1
G6N56_RS10865	2378519	2378842		0	putative quinol monooxygenase	WP_085253957.1
G6N56_RS10870	2379196	2379714		0	C40 family peptidase	WP_163645108.1
G6N56_RS10875	2379904	2380398		0	nitroreductase/quinone reductase family protein	WP_085256467.1
G6N56_RS10885	2380757	2381137		0	hypothetical protein	WP_180150517.1
G6N56_RS10890	2381261	2382262		0	hypothetical protein	WP_085256465.1
G6N56_RS10895	2382259	2382570		0	hypothetical protein	WP_085256464.1
G6N56_RS10900	2382642	2383142		0	nitroreductase/quinone reductase family protein	WP_085256463.1
G6N56_RS10905	2383192	2384826		0	recombinase family protein	WP_232069270.1
G6N56_RS10910	2384823	2385236		0	hypothetical protein	WP_142280660.1
G6N56_RS10915	2385333	2385551		0	helix-turn-helix domain-containing protein	WP_085256460.1
G6N56_RS10920	2385800	2386141		0	hypothetical protein	WP_232069271.1
G6N56_RS10925	2386138	2387565		0	AAA family ATPase	WP_085256459.1
G6N56_RS10930	2387747	2388142		0	hypothetical protein	WP_232069272.1
G6N56_RS10935	2388310	2388474		0	hypothetical protein	WP_158090727.1
G6N56_RS10940	2388471	2388962		0	hypothetical protein	WP_085256457.1
G6N56_RS10945	2389037	2389504		0	hypothetical protein	WP_085256456.1

G6N56_RS10950	2390041	2390442		0	hypothetical protein	WP_085256455.1
G6N56_RS10955	2390950	2391144		0	hypothetical protein	WP_142280659.1
G6N56_RS10960	2391523	2391840		0	hypothetical protein	WP_085256454.1
G6N56_RS10965	2391873	2392766		0	tyrosine-type recombinase/integrase	WP_232069273.1
G6N56_RS10970	2392763	2393035		0	hypothetical protein	WP_142280658.1
G6N56_RS10975	2393149	2394237	ychF		redox-regulated ATPase YchF	WP_085256452.1
G6N56_RS10980	2394447	2395613		0	hypothetical protein	WP_180150519.1
G6N56_RS10985	2395610	2396617		0	4-hydroxy-3-methylbut-2-enyl diphosphate reductase	WP_085256451.1
G6N56_RS10990	2396707	2397282		0	lipid droplet-associated protein	WP_085256450.1
G6N56_RS10995	2397279	2398529	xseA		exodeoxyribonuclease VII large subunit	WP_085256449.1
G6N56_RS11005	2398828	2399916		0	NAD-dependent epimerase/dehydratase family protein	WP_085256447.1
G6N56_RS11010	2399917	2401452		0	carboxylesterase/lipase family protein	WP_085256446.1
G6N56_RS11015	2401462	2402259		0	dienelactone hydrolase family protein	WP_085256445.1
G6N56_RS11020	2402282	2402971		0	DUF4245 domain-containing protein	WP_180150522.1
G6N56_RS11025	2403027	2404139	glpX		class II fructose-bisphosphatase	WP_085256443.1
G6N56_RS11030	2404167	2405591		0	class II fumarate hydratase	WP_085256442.1
G6N56_RS11040	2406681	2408141		0	adenylate/guanylate cyclase domain-containing protein	WP_085256440.1
G6N56_RS11045	2408216	2409091		0	polysaccharide deacetylase family protein	WP_085256439.1
G6N56_RS11050	2409159	2410460		0	PhoH family protein	WP_085256438.1
G6N56_RS11055	2410700	2411527		0	acyl-ACP desaturase	WP_085256470.1
G6N56_RS11060	2411636	2412916		0	serine hydroxymethyltransferase	WP_085256469.1
G6N56_RS11065	2413044	2413982	coaA		type I pantothenate kinase	WP_085256437.1
G6N56_RS11070	2414029	2414439		0	hypothetical protein	WP_085256436.1
G6N56_RS11075	2414732	2416102		0	hypothetical protein	WP_085256435.1
G6N56_RS11080	2416142	2416930		0	isoprenyl transferase	WP_085256434.1
G6N56_RS11085	2417090	2417860		0	hemolysin III family protein	WP_085256433.1
G6N56_RS11090	2417857	2418228		0	nuclear transport factor 2 family protein	WP_085256432.1
G6N56_RS11095	2418240	2420240		0	thioredoxin domain-containing protein	WP_085256431.1
G6N56_RS11105	2420514	2421386	mca		mycothiol conjugate amidase Mca	WP_085256429.1
G6N56_RS11110	2421500	2421934		0	DUF4307 domain-containing protein	WP_085256428.1
G6N56_RS11115	2422149	2422643	greA		transcription elongation factor GreA	WP_007168983.1

G6N56_RS11120	2422722	2423888	0	cystathionine gamma-synthase	WP_085256427.1
G6N56_RS11125	2423927	2424661	0	RDD family protein	WP_163645110.1
G6N56_RS11130	2424774	2426180	0	cystathionine beta-synthase	WP_085257815.1
G6N56_RS11135	2426230	2427297	0	alpha/beta hydrolase	WP_085257816.1
G6N56_RS11140	2427550	2428494	0	SGNH/GDSL hydrolase family protein	WP_142280797.1
G6N56_RS11145	2428590	2429807	0	acetyl-CoA C-acetyltransferase	WP_085257818.1
G6N56_RS11150	2430010	2430855	0	Bax inhibitor-1/YccA family protein	WP_085257819.1
G6N56_RS11155	2431083	2432120	0	enoyl-CoA hydratase/isomerase family protein	WP_085257820.1
G6N56_RS11160	2432133	2432924	0	enoyl-CoA hydratase	WP_085257821.1
G6N56_RS11165	2432935	2434680	0	alpha/beta-hydrolase family protein	WP_085257822.1
G6N56_RS11170	2434681	2435124	0	rhodanese-like domain-containing protein	WP_085257823.1
G6N56_RS11175	2435124	2435705	0	cysteine dioxygenase family protein	WP_085257824.1
G6N56_RS11180	2435876	2436271	0	lipoprotein LpqV	WP_158090764.1
G6N56_RS11185	2436369	2437457	0	patatin-like phospholipase family protein	WP_142280796.1
G6N56_RS11190	2437461	2438330	0	patatin-like phospholipase family protein	WP_085257827.1
G6N56_RS11195	2438359	2439228	0	class II glutamine amidotransferase	WP_085257828.1
G6N56_RS11205	2440661	2442286	0	long-chain fatty acid--CoA ligase	WP_085256099.1
G6N56_RS11210	2442518	2443573	0	YncE family protein	WP_085256100.1
G6N56_RS11215	2444348	2445421	0	class 1 fructose-bisphosphatase	WP_085256101.1
G6N56_RS11220	2445454	2446341	0	phosphoribulokinase	WP_085256102.1
G6N56_RS11225	2446338	2447837	0	NADH-quinone oxidoreductase subunit M	WP_142280631.1
G6N56_RS11230	2447837	2449723	nuoL	NADH-quinone oxidoreductase subunit L	WP_085256103.1
G6N56_RS11235	2449727	2449873	0	hypothetical protein	WP_158090724.1
G6N56_RS11240	2449949	2450794	0	hypothetical protein	WP_085256104.1
G6N56_RS11245	2450791	2451969	0	NHL repeat-containing protein	WP_085256105.1
G6N56_RS11250	2451966	2452841	0	NifU family protein	WP_085256106.1
G6N56_RS11255	2452838	2454088	0	hypothetical protein	WP_085256107.1
G6N56_RS11260	2454094	2454360	0	hypothetical protein	WP_085256117.1
G6N56_RS11265	2454366	2455193	0	hydrogenase maturation protease	WP_085256108.1
G6N56_RS11270	2455217	2456824	0	nickel-dependent hydrogenase large subunit	WP_085256109.1
G6N56_RS11275	2456900	2457871	0	hydrogenase	WP_085256110.1

G6N56_RS11280	2457908	2458465		0	DUF1641 domain-containing protein	WP_085256111.1
G6N56_RS11285	2458721	2459782		0	AraC family transcriptional regulator	WP_085256112.1
G6N56_RS11290	2459790	2460692		0	LysR family transcriptional regulator	WP_085256113.1
G6N56_RS11295	2460689	2461642	cbbX		CbbX protein	WP_085256114.1
G6N56_RS11300	2461639	2462064		0	ribulose bisphosphate carboxylase small subunit	WP_085256118.1
G6N56_RS11305	2462127	2463557		0	form I ribulose bisphosphate carboxylase large subunit	WP_085256115.1
G6N56_RS11315	2464720	2465946		0	GAF domain-containing sensor histidine kinase	WP_232069275.1
G6N56_RS11320	2465943	2466623		0	response regulator transcription factor	WP_085257836.1
G6N56_RS11325	2466769	2467452	rpe		ribulose-phosphate 3-epimerase	WP_085257837.1
G6N56_RS11330	2467449	2469536	tkt		transketolase	WP_085257838.1
G6N56_RS11340	2470635	2471738	hypD		hydrogenase formation protein HypD	WP_085254563.1
G6N56_RS11350	2471983	2474301	hypF		carbamoyltransferase HypF	WP_232069276.1
G6N56_RS11355	2474479	2474820		0	hydrogenase maturation nickel metallochaperone HypA	WP_085254573.1
G6N56_RS11360	2474852	2475658	hypB		hydrogenase nickel incorporation protein HypB	WP_085254565.1
G6N56_RS11365	2475689	2476453		0	DUF427 domain-containing protein	WP_085254566.1
G6N56_RS11370	2476464	2476736		0	DUF6295 family protein	WP_085254567.1
G6N56_RS11375	2476746	2477501		0	dienelactone hydrolase family protein	WP_197746666.1
G6N56_RS11380	2477584	2478393		0	hypothetical protein	WP_085254568.1
G6N56_RS11385	2478635	2479639		0	chemotaxis protein CheB	WP_085254569.1
G6N56_RS11390	2479642	2479785		0	hypothetical protein	WP_158090689.1
G6N56_RS11395	2479787	2481013		0	class I SAM-dependent methyltransferase	WP_085254575.1
G6N56_RS11405	2481561	2482397		0	PE domain-containing protein	WP_085254570.1
G6N56_RS11410	2482456	2483661		0	PPE family protein	WP_085254571.1
G6N56_RS11425	2484609	2485952		0	PPE family protein	WP_085256662.1
G6N56_RS11430	2486665	2487441		0	response regulator transcription factor	WP_085256661.1
G6N56_RS11435	2487438	2488967		0	HAMP domain-containing sensor histidine kinase	WP_085256660.1
G6N56_RS29470	2489776	2493312		0	carboxylic acid reductase	WP_085256659.1
G6N56_RS11455	2493719	2494132		0	hypothetical protein	WP_142280676.1
G6N56_RS29040	2494731	2495069		0	hypothetical protein	WP_232069277.1
G6N56_RS29045	2495203	2495385		0	hypothetical protein	WP_211287400.1
G6N56_RS11470	2495382	2495471		0	potassium-transporting ATPase subunit F	WP_142280675.1

G6N56_RS11475	2495471	2497144	kdpA		potassium-transporting ATPase subunit KdpA	WP_085256656.1
G6N56_RS11480	2497141	2499294	kdpB		potassium-transporting ATPase subunit KdpB	WP_163645112.1
G6N56_RS11485	2499296	2500219		0	potassium-transporting ATPase subunit C	WP_085256654.1
G6N56_RS11490	2500231	2502792		0	sensor histidine kinase KdpD	WP_085256653.1
G6N56_RS11495	2502789	2503469		0	response regulator	WP_085256652.1
G6N56_RS11500	2503568	2504518		0	Ppx/GppA phosphatase family protein	WP_085256651.1
G6N56_RS11510	2504993	2505679		0	septum formation initiator family protein	WP_085256649.1
G6N56_RS11515	2505692	2506981	eno		phosphopyruvate hydratase	WP_085256648.1
G6N56_RS11520	2507062	2507790		0	lytic transglycosylase domain-containing protein	WP_142280681.1
G6N56_RS11525	2508066	2509067		0	nucleoside triphosphate pyrophosphohydrolase	WP_085256646.1
G6N56_RS11530	2509117	2512773	mfd		transcription-repair coupling factor	WP_085256645.1
G6N56_RS11535	2512882	2514222	lysA		diaminopimelate decarboxylase	WP_085256644.1
G6N56_RS11540	2514229	2514822		0	TetR/AcrR family transcriptional regulator	WP_142280680.1
G6N56_RS11550	2515139	2516599	glmU		bifunctional UDP-N-acetylglucosamine diphosphorylase/gluco	WP_085256642.1
G6N56_RS11555	2516679	2517659		0	ribose-phosphate diphosphokinase	WP_085256641.1
G6N56_RS11560	2517660	2518007	arsC		arsenate reductase (glutaredoxin)	WP_085256640.1
G6N56_RS11565	2518004	2518651		0	LpqN/LpqT family lipoprotein	WP_085256639.1
G6N56_RS11575	2519283	2519921		0	50S ribosomal protein L25/general stress protein Ctc	WP_085256638.1
G6N56_RS11580	2519934	2520509	pth		aminoacyl-tRNA hydrolase	WP_085256637.1
G6N56_RS11585	2520588	2522222		0	fatty acyl-AMP ligase	WP_085256636.1
G6N56_RS11590	2522522	2523472		0	4-(cytidine 5'-diphospho)-2-C-methyl-D-erythritol kinase	WP_085256635.1
G6N56_RS11600	2524460	2525587		0	resuscitation-promoting factor	WP_085256634.1
G6N56_RS11605	2525734	2526558		0	TatD family hydrolase	WP_085256633.1
G6N56_RS11610	2526615	2528174	metG		methionine--tRNA ligase	WP_085256669.1
G6N56_RS11615	2528298	2528660		0	PAS domain-containing protein	WP_085256632.1
G6N56_RS11620	2528846	2529550		0	TetR/AcrR family transcriptional regulator	WP_085256631.1
G6N56_RS11625	2529680	2530357		0	TetR/AcrR family transcriptional regulator	WP_085256630.1
G6N56_RS11630	2530907	2532568		0	hypothetical protein	WP_085256668.1
G6N56_RS11635	2532580	2534445		0	EAL domain-containing protein	WP_085256667.1
G6N56_RS11640	2534736	2535920		0	FAD-dependent oxidoreductase	WP_085256629.1
G6N56_RS11645	2535917	2536816		0	RNA polymerase sigma-70 factor	WP_085256628.1

G6N56_RS11650	2536866	2538128	0	aminodeoxychorismate synthase component I	WP_085256627.1
G6N56_RS11655	2538314	2539366	0	hypothetical protein	WP_085256626.1
G6N56_RS11660	2539375	2540211	rsmI	16S rRNA (cytidine(1402)-2'-O)-methyltransferase	WP_085256625.1
G6N56_RS11665	2540236	2541816	0	dolichyl-phosphate-mannose--protein mannosyltransferase	WP_085256624.1
G6N56_RS11670	2541936	2543150	arcA	arginine deiminase	WP_085256623.1
G6N56_RS11675	2543219	2543815	0	alpha-ketoglutarate-dependent dioxygenase AlkB	WP_180150561.1
G6N56_RS28410	2543828	2544484	0	DUF5642 family protein	WP_085256621.1
G6N56_RS28415	2544512	2545201	0	DUF5642 family protein	WP_085256666.1
G6N56_RS11685	2545252	2546202	0	GNAT family N-acetyltransferase	WP_142280677.1
G6N56_RS11690	2546742	2547482	0	methyltransferase domain-containing protein	WP_085256619.1
G6N56_RS11695	2547488	2547739	0	hypothetical protein	WP_085256618.1
G6N56_RS11700	2548017	2549141	0	M20/M25/M40 family metallo-hydrolase	WP_085256617.1
G6N56_RS11705	2549372	2549911	0	YceI family protein	WP_085256665.1
G6N56_RS11710	2550457	2550612	0	hypothetical protein	WP_158090735.1
G6N56_RS11715	2550628	2550831	0	hypothetical protein	WP_085256616.1
G6N56_RS11720	2550889	2551635	0	SDR family oxidoreductase	WP_085256615.1
G6N56_RS11725	2551747	2552295	0	TetR/AcrR family transcriptional regulator	WP_085256614.1
G6N56_RS11730	2552357	2552797	0	helix-turn-helix domain-containing protein	WP_158090734.1
G6N56_RS11735	2552838	2553899	0	hypothetical protein	WP_142280673.1
G6N56_RS11745	2554286	2554522	0	hypothetical protein	WP_085256611.1
G6N56_RS11750	2554550	2554987	0	hypothetical protein	WP_085256610.1
G6N56_RS11755	2555167	2556924	0	ATP-binding protein	WP_085256609.1
G6N56_RS11760	2556988	2557365	0	hypothetical protein	WP_085256608.1
G6N56_RS11765	2557362	2557838	0	hypothetical protein	WP_142280672.1
G6N56_RS11770	2558064	2558528	0	YbjN domain-containing protein	WP_085256606.1
G6N56_RS11775	2558693	2560507	0	AIPR family protein	WP_085256605.1
G6N56_RS11785	2561941	2562333	dndE	DNA sulfur modification protein DndE	WP_085256603.1
G6N56_RS11790	2562336	2564312	dndD	DNA sulfur modification protein DndD	WP_085256602.1
G6N56_RS11795	2564309	2565850	dndC	DNA phosphorothioation system sulfurtransferase DndC	WP_085256601.1
G6N56_RS11800	2565847	2566995	dndB	DNA sulfur modification protein DndB	WP_085256600.1
G6N56_RS11805	2567095	2568294	dndA	cysteine desulfurase DndA	WP_085256599.1

G6N56_RS11810	2568308	2568481	0	hypothetical protein	WP_158090732.1
G6N56_RS11815	2568547	2569458	0	hypothetical protein	WP_085256598.1
G6N56_RS11820	2569461	2569796	0	hypothetical protein	WP_085256597.1
G6N56_RS11825	2570006	2570920	0	hypothetical protein	WP_232069331.1
G6N56_RS11830	2571014	2571385	0	hypothetical protein	WP_085256596.1
G6N56_RS11835	2572596	2573669	0	hypothetical protein	WP_142280670.1
G6N56_RS11840	2573698	2574816	0	DUF4062 domain-containing protein	WP_158090731.1
G6N56_RS11845	2574813	2575376	0	hypothetical protein	WP_085256591.1
G6N56_RS11850	2575874	2576146	0	hypothetical protein	WP_085256590.1
G6N56_RS11855	2576314	2577504	0	hypothetical protein	WP_085256589.1
G6N56_RS11865	2579247	2580041	0	hypothetical protein	WP_085256588.1
G6N56_RS11870	2580043	2581122	0	hypothetical protein	WP_085256587.1
G6N56_RS11875	2581119	2581919	0	VWA domain-containing protein	WP_085256586.1
G6N56_RS11880	2581916	2582464	0	hypothetical protein	WP_085256585.1
G6N56_RS11885	2582461	2582664	0	hypothetical protein	WP_085256584.1
G6N56_RS11895	2583057	2585255	0	type IV secretion system DNA-binding domain-containing prote	WP_085256582.1
G6N56_RS11900	2585252	2586247	0	replication-relaxation family protein	WP_085256581.1
G6N56_RS11905	2586244	2587278	0	site-specific DNA-methyltransferase	WP_085256580.1
G6N56_RS11910	2587365	2587697	0	hypothetical protein	WP_085256579.1
G6N56_RS11915	2587866	2588444	0	antirestriction protein ArdA	WP_232069278.1
G6N56_RS11920	2588509	2589051	0	helix-turn-helix transcriptional regulator	WP_232069279.1
G6N56_RS29055	2589767	2590153	0	hypothetical protein	WP_232069280.1
G6N56_RS11935	2590214	2590456	0	hypothetical protein	WP_163645113.1
G6N56_RS11940	2590514	2590663	0	hypothetical protein	WP_163645114.1
G6N56_RS11950	2590913	2591932	0	hypothetical protein	WP_085256080.1
G6N56_RS11955	2592125	2592778	0	GNAT family protein	WP_085256079.1
G6N56_RS11960	2592785	2594077	0	molybdopterin molybdotransferase MoeA	WP_085256078.1
G6N56_RS11965	2594124	2595035	0	UTP--glucose-1-phosphate uridylyltransferase	WP_085256077.1
G6N56_RS11970	2595112	2595747	0	5-formyltetrahydrofolate cyclo-ligase	WP_085256098.1
G6N56_RS11975	2595792	2596139	0	zinc ribbon domain-containing protein	WP_085256076.1
G6N56_RS11980	2596205	2596873	0	SAF domain-containing protein	WP_085256075.1

G6N56_RS11985	2596940	2597401	mscL		large-conductance mechanosensitive channel protein MscL	WP_085256074.1
G6N56_RS11990	2597418	2597975		0	MogA/MoaB family molybdenum cofactor biosynthesis protein	WP_085256073.1
G6N56_RS11995	2597976	2599439		0	trypsin-like peptidase domain-containing protein	WP_085256072.1
G6N56_RS12000	2599520	2601076		0	HAMP domain-containing sensor histidine kinase	WP_085256071.1
G6N56_RS12005	2601076	2601762	mprA		two-component system response regulator MprA	WP_085256097.1
G6N56_RS12010	2601853	2602026	rpmF		50S ribosomal protein L32	WP_085256070.1
G6N56_RS12015	2602322	2604034		0	acyclic terpene utilization AtuA family protein	WP_085256096.1
G6N56_RS12020	2604031	2605179		0	acyl-CoA dehydrogenase family protein	WP_085256069.1
G6N56_RS12025	2605176	2606771		0	acyl-CoA carboxylase subunit beta	WP_085256068.1
G6N56_RS12030	2606777	2608744		0	biotin carboxylase N-terminal domain-containing protein	WP_085256067.1
G6N56_RS12035	2608755	2609921		0	acyl-CoA dehydrogenase family protein	WP_085256066.1
G6N56_RS12040	2609931	2610824		0	enoyl-CoA hydratase family protein	WP_085256065.1
G6N56_RS12045	2610902	2611504		0	DUF1707 domain-containing protein	WP_085256095.1
G6N56_RS12050	2611506	2612354		0	aldo/keto reductase	WP_085256064.1
G6N56_RS12055	2612412	2613131		0	aquaporin	WP_085256063.1
G6N56_RS12060	2613218	2615212		0	VWA domain-containing protein	WP_085256062.1
G6N56_RS12070	2616662	2618245	purH		bifunctional phosphoribosylaminoimidazolecarboxamide formy	WP_085256094.1
G6N56_RS12075	2618260	2618889	purN		phosphoribosylglycinamide formyltransferase	WP_085256060.1
G6N56_RS12080	2618951	2620306		0	DUF6350 family protein	WP_085256059.1
G6N56_RS12085	2620355	2621284		0	DUF5336 domain-containing protein	WP_085256058.1
G6N56_RS12090	2621485	2622333		0	LLM class F420-dependent oxidoreductase	WP_085256057.1
G6N56_RS12095	2622409	2623929		0	acetyl-CoA acetyltransferase	WP_085256056.1
G6N56_RS12100	2623981	2624883	sucD		succinate--CoA ligase subunit alpha	WP_085256055.1
G6N56_RS12105	2624896	2626059	sucC		ADP-forming succinate--CoA ligase subunit beta	WP_085256054.1
G6N56_RS12110	2626383	2627438		0	M23 family metallopeptidase	WP_232069332.1
G6N56_RS12115	2627440	2628003		0	hypothetical protein	WP_142280625.1
G6N56_RS12120	2628078	2630402	pcrA		DNA helicase PcrA	WP_085256052.1
G6N56_RS12125	2630647	2630964		0	chorismate mutase	WP_085256051.1
G6N56_RS12130	2631320	2632984	pgi		glucose-6-phosphate isomerase	WP_085256050.1
G6N56_RS12135	2633013	2633762		0	SDR family oxidoreductase	WP_085256049.1
G6N56_RS29060	2633850	2634317		0	zinc finger domain-containing protein	WP_232069333.1

G6N56_RS12145	2634718	2635461	cobF		precorrin-6A synthase (deacetylating)	WP_085256047.1
G6N56_RS12150	2635580	2636485		0	diiron oxygenase	WP_085256046.1
G6N56_RS12155	2636728	2637708		0	DUF4873 domain-containing protein	WP_232069334.1
G6N56_RS12160	2638212	2638973		0	STAS domain-containing protein	WP_085256045.1
G6N56_RS12165	2639095	2639961		0	LLM class flavin-dependent oxidoreductase	WP_085256044.1
G6N56_RS12170	2640245	2641108		0	polyphosphate kinase 2 family protein	WP_232069281.1
G6N56_RS12175	2642113	2643300		0	PPE family protein	WP_085256043.1
G6N56_RS12180	2643884	2644636		0	YoaK family protein	WP_158090722.1
G6N56_RS12185	2644920	2645837		0	SAM-dependent methyltransferase	WP_085256041.1
G6N56_RS12190	2645914	2647851		0	fumarylacetoacetate hydrolase family protein	WP_085256040.1
G6N56_RS12195	2647848	2650103		0	ATP-dependent DNA ligase	WP_085256039.1
G6N56_RS12200	2650230	2651747		0	mannitol dehydrogenase family protein	WP_085256038.1
G6N56_RS12205	2651744	2652610		0	HAD family hydrolase	WP_085256037.1
G6N56_RS12210	2652668	2654077		0	mannitol dehydrogenase family protein	WP_085256036.1
G6N56_RS12215	2654074	2655000		0	carbohydrate kinase	WP_085256035.1
G6N56_RS12220	2655165	2656103		0	Ku protein	WP_085256034.1
G6N56_RS12225	2656113	2657015	pstA		phosphate ABC transporter permease PstA	WP_085256033.1
G6N56_RS12230	2657062	2657997	pstC		phosphate ABC transporter permease subunit PstC	WP_232069335.1
G6N56_RS12235	2658067	2659182	pstS		phosphate ABC transporter substrate-binding protein PstS	WP_142280624.1
G6N56_RS12240	2659470	2660261		0	SDR family oxidoreductase	WP_085256030.1
G6N56_RS12245	2660272	2661348		0	diacylglycerol kinase	WP_085256029.1
G6N56_RS12250	2661644	2662870		0	Nramp family divalent metal transporter	WP_085256028.1
G6N56_RS12255	2662871	2663926		0	poly-gamma-glutamate hydrolase family protein	WP_085256027.1
G6N56_RS12260	2663949	2664773		0	LLM class F420-dependent oxidoreductase	WP_085256026.1
G6N56_RS29070	2666246	2666485		0	hypothetical protein	WP_232069432.1
G6N56_RS12275	2666509	2666883		0	nucleotidyl transferase AbiEii/AbiGii toxin family protein	WP_264019561.1
G6N56_RS12285	2667302	2668063		0	glucose 1-dehydrogenase	WP_085256024.1
G6N56_RS12290	2668172	2669167		0	NADPH:quinone oxidoreductase family protein	WP_085256023.1
G6N56_RS12295	2669164	2670885		0	class I adenylate-forming enzyme family protein	WP_085256022.1
G6N56_RS12300	2670943	2671791		0	cyclase family protein	WP_142280623.1
G6N56_RS12305	2671893	2672699		0	aldolase/citrate lyase family protein	WP_085256020.1

G6N56_RS12315	2673487	2674155	0	TetR/AcrR family transcriptional regulator	WP_142280622.1
G6N56_RS12320	2674593	2675882	0	acyl-CoA dehydrogenase family protein	WP_085256090.1
G6N56_RS12325	2676168	2677163	0	phosphotransferase family protein	WP_085256017.1
G6N56_RS12330	2677160	2677870	0	GntR family transcriptional regulator	WP_085256016.1
G6N56_RS12335	2678017	2678550	0	cupin domain-containing protein	WP_085256089.1
G6N56_RS12340	2679199	2679693	0	SDR family oxidoreductase	WP_232069282.1
G6N56_RS12345	2679898	2680440	0	TetR/AcrR family transcriptional regulator C-terminal domain-c	WP_232069283.1
G6N56_RS12350	2680632	2681045	0	thioesterase family protein	WP_085256013.1
G6N56_RS12355	2681423	2682157	0	SDR family oxidoreductase	WP_085256012.1
G6N56_RS12360	2682161	2682640	0	nitroreductase family deazaflavin-dependent oxidoreductase	WP_085256011.1
G6N56_RS12365	2682640	2683317	0	TetR/AcrR family transcriptional regulator	WP_085256010.1
G6N56_RS12375	2684223	2685398	0	glycosyltransferase	WP_085256009.1
G6N56_RS12380	2685424	2686992	0	GMC family oxidoreductase N-terminal domain-containing prot	WP_180150526.1
G6N56_RS12385	2687027	2688052	0	helix-turn-helix domain-containing protein	WP_085256008.1
G6N56_RS28550	2688504	2690522	0	adenylate/guanylate cyclase domain-containing protein	WP_232069284.1
G6N56_RS12395	2691017	2691274	0	hypothetical protein	WP_085256006.1
G6N56_RS12400	2691291	2692790	0	DUF4185 domain-containing protein	WP_085256005.1
G6N56_RS12405	2693238	2693465	0	lipase chaperone	WP_085256003.1
G6N56_RS12410	2693544	2694950	0	MFS transporter	WP_232069285.1
G6N56_RS12415	2695180	2696406	0	acetyl-CoA acetyltransferase	WP_085256002.1
G6N56_RS12420	2696409	2697923	0	carotenoid oxygenase family protein	WP_085256001.1
G6N56_RS12425	2697920	2698405	0	VOC family protein	WP_085256086.1
G6N56_RS12430	2698409	2698834	0	hypothetical protein	WP_085256000.1
G6N56_RS12435	2699018	2699491	0	SRPBCC family protein	WP_085255999.1
G6N56_RS12440	2699496	2699684	0	antitoxin	WP_085255998.1
G6N56_RS12445	2699733	2702114	0	HAD-IC family P-type ATPase	WP_085255997.1
G6N56_RS12450	2702111	2703694	0	serine hydrolase	WP_085255996.1
G6N56_RS12455	2703725	2704843	0	MBL fold metallo-hydrolase	WP_085255995.1
G6N56_RS12460	2704849	2705580	0	enoyl-CoA hydratase	WP_085255994.1
G6N56_RS12465	2705608	2707080	0	carboxyl transferase domain-containing protein	WP_085255993.1
G6N56_RS12470	2707182	2707355	0	hypothetical protein	WP_232069286.1

G6N56_RS12475	2707397	2707702		0	FAD-dependent monooxygenase	WP_232069336.1	
G6N56_RS12480	2707727	2708428	prrA		two-component system response regulator PrrA	WP_163645116.1	
G6N56_RS12485	2708439	2709779		0	HAMP domain-containing sensor histidine kinase	WP_085255991.1	
G6N56_RS12490	2709776	2709994		0	hypothetical protein		0
G6N56_RS12495	2712075	2712347		0	hypothetical protein	WP_142280621.1	
G6N56_RS28420	2712349	2712501		0	hypothetical protein	WP_169717525.1	
G6N56_RS12500	2712513	2713520		0	OmpA family protein	WP_085255990.1	
G6N56_RS12505	2713629	2713892		0	DUF2630 family protein	WP_085255989.1	
G6N56_RS12510	2713889	2715457		0	NAD(P)/FAD-dependent oxidoreductase	WP_142280628.1	
G6N56_RS12515	2715554	2716849		0	citrate synthase	WP_085255988.1	
G6N56_RS12520	2717306	2718184		0	amidohydrolase family protein	WP_085255987.1	
G6N56_RS12525	2718251	2718922	pdxH		pyridoxamine 5'-phosphate oxidase	WP_085255986.1	
G6N56_RS12530	2718994	2720115		0	citrate synthase 2	WP_085255985.1	
G6N56_RS12535	2720237	2720686		0	VOC family protein	WP_085255984.1	
G6N56_RS12540	2720694	2722391		0	4Fe-4S binding protein	WP_085255983.1	
G6N56_RS12545	2722410	2723432		0	diiron oxygenase	WP_085255982.1	
G6N56_RS12550	2723678	2724796	serC		phosphoserine transaminase	WP_085256084.1	
G6N56_RS12555	2724917	2725675	sepH		septation protein SepH	WP_085255981.1	
G6N56_RS12565	2725921	2726745		0	RNA methyltransferase	WP_085255979.1	
G6N56_RS12570	2726742	2727173		0	MarR family transcriptional regulator	WP_085255978.1	
G6N56_RS12575	2727283	2727558		0	DUF2530 domain-containing protein	WP_085256083.1	
G6N56_RS12580	2727588	2728043		0	SRPBCC family protein	WP_085255977.1	
G6N56_RS12585	2728040	2728990		0	DUF3027 domain-containing protein	WP_142280620.1	
G6N56_RS12590	2729069	2730787		0	MFS transporter	WP_142280619.1	
G6N56_RS12595	2730784	2731284		0	DUF2771 domain-containing protein	WP_085255974.1	
G6N56_RS12600	2731461	2732327		0	DUF559 domain-containing protein	WP_085255973.1	
G6N56_RS12605	2732400	2733404		0	glutathione S-transferase C-terminal domain-containing protei	WP_085256082.1	
G6N56_RS12610	2733558	2733968		0	cold-shock protein	WP_085255972.1	
G6N56_RS12615	2734133	2734537		0	YccF domain-containing protein	WP_085255971.1	
G6N56_RS12620	2734534	2735622	moaA		GTP 3',8-cyclase MoaA	WP_085255970.1	
G6N56_RS12625	2735627	2735899		0	MoaD/ThiS family protein	WP_085255969.1	

G6N56_RS12630	2736337	2737113		0	transglycosylase family protein	WP_163645117.1
G6N56_RS12635	2737214	2737639		0	molybdenum cofactor biosynthesis protein MoaE	WP_085255886.1
G6N56_RS12640	2737636	2738118		0	MogA/MoaB family molybdenum cofactor biosynthesis protein	WP_085255885.1
G6N56_RS12645	2738118	2738606	moaC		cyclic pyranopterin monophosphate synthase MoaC	WP_085255884.1
G6N56_RS12650	2738614	2738814		0	hypothetical protein	WP_142280608.1
G6N56_RS12655	2738949	2741210		0	helicase-associated domain-containing protein	WP_085255882.1
G6N56_RS12660	2741272	2742921		0	DEAD/DEAH box helicase	WP_085255881.1
G6N56_RS12665	2743061	2743354		0	antibiotic biosynthesis monooxygenase	WP_085255880.1
G6N56_RS12670	2743351	2744340		0	LLM class F420-dependent oxidoreductase	WP_085255879.1
G6N56_RS12675	2744476	2745699		0	FAD-dependent monooxygenase	WP_085255878.1
G6N56_RS12680	2745704	2746564		0	LLM class F420-dependent oxidoreductase	WP_085255914.1
G6N56_RS12685	2746733	2748877		0	3-hydroxyacyl-CoA dehydrogenase NAD-binding domain-contai	WP_085255877.1
G6N56_RS12690	2748882	2750093		0	acetyl-CoA C-acetyltransferase	WP_085255876.1
G6N56_RS12695	2750278	2751447		0	pyridoxal phosphate-dependent aminotransferase	WP_085255875.1
G6N56_RS12700	2751452	2751904		0	SRPBCC family protein	WP_085255874.1
G6N56_RS12705	2751966	2752421		0	SRPBCC family protein	WP_085255873.1
G6N56_RS12710	2752479	2753558		0	CaiB/BaiF CoA-transferase family protein	WP_085255872.1
G6N56_RS12715	2753564	2754007		0	SRPBCC family protein	WP_085255913.1
G6N56_RS12720	2754066	2755772		0	alpha-keto acid decarboxylase family protein	WP_085255912.1
G6N56_RS12725	2755794	2755973		0	(2Fe-2S)-binding protein	WP_085255871.1
G6N56_RS12730	2755987	2756610		0	fatty-acid--CoA ligase	WP_085255911.1
G6N56_RS12735	2756621	2757151		0	twin-arginine translocation pathway signal	WP_232069337.1
G6N56_RS12740	2757277	2758158		0	hypothetical protein	WP_085255910.1
G6N56_RS12745	2758351	2758932		0	hypothetical protein	WP_142280606.1
G6N56_RS12750	2758975	2760411		0	FadD3 family acyl-CoA ligase	WP_085255868.1
G6N56_RS12755	2760408	2761598		0	amidohydrolase family protein	WP_085255867.1
G6N56_RS12760	2761683	2762105		0	lipoprotein LpqH	WP_085255866.1
G6N56_RS12765	2762216	2762962		0	enoyl-CoA hydratase/isomerase family protein	WP_085255865.1
G6N56_RS12775	2763300	2764022		0	TetR/AcrR family transcriptional regulator	WP_085255863.1
G6N56_RS12780	2764238	2765509		0	cytochrome P450	WP_085255862.1
G6N56_RS12785	2765519	2765800		0	cytochrome C oxidase subunit IV family protein	WP_085255861.1

G6N56_RS12790	2765804	2766376	0	cytochrome c oxidase subunit 3	WP_085255860.1
G6N56_RS12795	2766373	2767116	0	hypothetical protein	WP_085255859.1
G6N56_RS12800	2767113	2767880	0	hypothetical protein	WP_085255858.1
G6N56_RS12805	2768051	2768566	0	TetR/AcrR family transcriptional regulator	WP_085255909.1
G6N56_RS12810	2768730	2769017	0	hypothetical protein	WP_085255857.1
G6N56_RS12815	2769036	2769443	0	hypothetical protein	WP_232069338.1
G6N56_RS29080	2769878	2770345	0	hypothetical protein	WP_232069287.1
G6N56_RS12825	2770468	2770929	0	twin-arginine translocation pathway signal	WP_232069288.1
G6N56_RS12830	2771043	2771558	0	hypothetical protein	WP_085255854.1
G6N56_RS12835	2771584	2773248	0	MlaD family protein	WP_085255853.1
G6N56_RS12840	2773249	2774691	0	MCE family protein	WP_085255852.1
G6N56_RS12845	2774688	2776115	0	MCE family protein	WP_142280605.1
G6N56_RS12850	2776112	2777194	0	MCE family protein	WP_085255851.1
G6N56_RS12860	2778224	2779501	0	MCE family protein	WP_085255849.1
G6N56_RS12865	2779521	2780366	0	ABC transporter permease	WP_085255848.1
G6N56_RS12870	2780386	2781135	0	ABC transporter permease	WP_232069339.1
G6N56_RS12875	2781534	2782739	0	CoA transferase	WP_085255846.1
G6N56_RS12880	2782736	2784172	0	aldehyde dehydrogenase family protein	WP_232069340.1
G6N56_RS12885	2784518	2785219	0	SDR family NAD(P)-dependent oxidoreductase	WP_085255844.1
G6N56_RS12890	2785383	2785580	0	ferredoxin	WP_085255843.1
G6N56_RS12895	2785595	2786959	0	cytochrome P450	WP_085255842.1
G6N56_RS12900	2787046	2788221	0	acyl-CoA dehydrogenase family protein	WP_085255841.1
G6N56_RS12910	2789304	2789954	0	nitroreductase family protein	WP_085255840.1
G6N56_RS12915	2790136	2790531	0	carboxymuconolactone decarboxylase family protein	WP_085255839.1
G6N56_RS12920	2790919	2792010	0	amidohydrolase family protein	WP_085255838.1
G6N56_RS12925	2792041	2793186	0	amidohydrolase family protein	WP_085255837.1
G6N56_RS12930	2793188	2793601	0	Rieske 2Fe-2S domain-containing protein	WP_142280604.1
G6N56_RS12935	2793618	2794484	0	alpha/beta hydrolase	WP_085255836.1
G6N56_RS12940	2794490	2794963	0	hypothetical protein	WP_085255835.1
G6N56_RS12945	2794997	2796295	0	NADH-ubiquinone oxidoreductase-F iron-sulfur binding region c	WP_085255834.1
G6N56_RS12955	2796582	2798243	0	OB-fold domain-containing protein	WP_085255833.1

G6N56_RS12960	2798240	2799190	0	alpha/beta hydrolase	WP_085255832.1
G6N56_RS12965	2799187	2800002	0	NAD(P)-dependent oxidoreductase	WP_085255831.1
G6N56_RS12970	2799999	2800796	0	NAD(P)-dependent oxidoreductase	WP_085255830.1
G6N56_RS12975	2800906	2802108	0	cytochrome P450	WP_085255829.1
G6N56_RS12980	2802105	2802296	0	ferredoxin	WP_085255828.1
G6N56_RS12985	2802301	2802615	0	DUF1330 domain-containing protein	WP_085255827.1
G6N56_RS12990	2802643	2802834	0	ferredoxin	WP_085255826.1
G6N56_RS12995	2802831	2804105	0	cytochrome P450	WP_085255825.1
G6N56_RS13000	2804236	2804751	0	hypothetical protein	WP_085255824.1
G6N56_RS13005	2804748	2805356	0	TetR/AcrR family transcriptional regulator	WP_085255823.1
G6N56_RS13010	2805455	2806603	0	thiolase family protein	WP_085255822.1
G6N56_RS13015	2806751	2807917	0	acyl-CoA dehydrogenase family protein	WP_085255821.1
G6N56_RS13020	2808030	2809328	0	amidohydrolase family protein	WP_085255820.1
G6N56_RS13025	2809775	2810314	0	carboxymuconolactone decarboxylase family protein	WP_085255819.1
G6N56_RS13030	2810319	2811605	0	aromatic ring-hydroxylating dioxygenase subunit alpha	WP_085255818.1
G6N56_RS13040	2811916	2813136	0	amidohydrolase family protein	WP_085255816.1
G6N56_RS13050	2813542	2814639	0	acyl-CoA dehydrogenase family protein	WP_085255814.1
G6N56_RS13055	2814641	2815444	0	acyl-CoA dehydrogenase family protein	WP_085255813.1
G6N56_RS13060	2815555	2815818	0	DUF4190 domain-containing protein	WP_085255812.1
G6N56_RS13065	2815815	2816990	0	acyl-CoA dehydrogenase family protein	WP_085255811.1
G6N56_RS13070	2817231	2817881	0	TetR/AcrR family transcriptional regulator	WP_085255810.1
G6N56_RS13075	2818096	2818935	0	mycofactocin-coupled SDR family oxidoreductase	WP_085255809.1
G6N56_RS13080	2818946	2819776	0	SDR family NAD(P)-dependent oxidoreductase	WP_085255808.1
G6N56_RS13085	2819867	2820403	0	cupin domain-containing protein	WP_085255807.1
G6N56_RS13090	2820418	2821224	0	SDR family oxidoreductase	WP_085255806.1
G6N56_RS13100	2822374	2823552	0	Xaa-Pro peptidase family protein	WP_085255903.1
G6N56_RS13105	2823621	2824682	0	amidohydrolase family protein	WP_180150565.1
G6N56_RS13110	2824835	2826082	0	cytochrome P450	WP_085255804.1
G6N56_RS13115	2826084	2826278	0	ferredoxin	WP_085255803.1
G6N56_RS13120	2826289	2826966	0	TetR family transcriptional regulator	WP_085255802.1
G6N56_RS13125	2827157	2827798	0	PAS domain-containing protein	WP_085255801.1

G6N56_RS13130	2827813	2828628		0	SDR family oxidoreductase	WP_085255800.1
G6N56_RS13135	2828820	2829587		0	SDR family oxidoreductase	WP_085255901.1
G6N56_RS13140	2829619	2830431		0	alpha/beta fold hydrolase	WP_085255900.1
G6N56_RS13145	2830461	2831450		0	cyclase family protein	WP_085255899.1
G6N56_RS13155	2832300	2833607		0	LLM class flavin-dependent oxidoreductase	WP_085255799.1
G6N56_RS13160	2833611	2835350		0	FAD-binding protein	WP_085255798.1
G6N56_RS13170	2836212	2837906		0	bifunctional 3-(3-hydroxy-phenyl)propionate/3-hydroxycinnami	WP_085255796.1
G6N56_RS13175	2837906	2838769		0	alpha/beta hydrolase	WP_085255795.1
G6N56_RS13180	2838880	2839560		0	helix-turn-helix domain-containing protein	WP_085255794.1
G6N56_RS13185	2839586	2841202		0	FAD-dependent oxidoreductase	WP_085255793.1
G6N56_RS13190	2841312	2842529		0	histidine kinase	WP_085255897.1
G6N56_RS13200	2843279	2844193		0	thiamine pyrophosphate-dependent enzyme	WP_180150528.1
G6N56_RS13205	2844346	2845224	meaB		methylmalonyl Co-A mutase-associated GTPase MeaB	WP_085255791.1
G6N56_RS13210	2845350	2845910		0	GNAT family N-acetyltransferase	WP_085255895.1
G6N56_RS13215	2846003	2846869		0	proline iminopeptidase-family hydrolase	WP_085255790.1
G6N56_RS13220	2846881	2847648		0	enoyl-CoA hydratase/isomerase family protein	WP_085255789.1
G6N56_RS13225	2847650	2848738		0	acyl-CoA dehydrogenase family protein	WP_085255788.1
G6N56_RS13235	2849608	2851011		0	AMP-binding protein	WP_085255786.1
G6N56_RS13240	2851093	2851416		0	2Fe-2S iron-sulfur cluster-binding protein	WP_085255785.1
G6N56_RS13245	2851433	2851753		0	hypothetical protein	WP_085255784.1
G6N56_RS13250	2851848	2852744		0	TIGR03620 family F420-dependent LLM class oxidoreductase	WP_232069290.1
G6N56_RS13255	2852741	2853634		0	SDR family oxidoreductase	WP_085255782.1
G6N56_RS13260	2853794	2854690		0	LysR family transcriptional regulator	WP_085255781.1
G6N56_RS13265	2854791	2855810		0	methyltransferase	WP_085255780.1
G6N56_RS13270	2855912	2856952		0	CoA transferase	WP_085255779.1
G6N56_RS13280	2857884	2858309		0	cobalamin B12-binding domain-containing protein	WP_085255777.1
G6N56_RS13285	2858310	2859887		0	methylmalonyl-CoA mutase family protein	WP_085255776.1
G6N56_RS13290	2859972	2861225		0	MFS transporter	WP_085255775.1
G6N56_RS13295	2861367	2862173		0	methyltransferase domain-containing protein	WP_085255774.1
G6N56_RS13305	2862990	2863622		0	TetR family transcriptional regulator	WP_142280600.1
G6N56_RS13310	2863684	2865405		0	CocE/NonD family hydrolase	WP_085255772.1

G6N56_RS13315	2866128	2866763		0	maleylpyruvate isomerase family mycothiol-dependent enzyme	WP_085255770.1
G6N56_RS13320	2867072	2867509		0	MarR family transcriptional regulator	WP_085255769.1
G6N56_RS13325	2867628	2869115		0	MFS transporter	WP_197746667.1
G6N56_RS13330	2869168	2869377		0	hypothetical protein	WP_085255767.1
G6N56_RS13335	2869668	2870546		0	LLM class F420-dependent oxidoreductase	WP_085255766.1
G6N56_RS13365	2872749	2873546		0	TIGR04255 family protein	WP_232069291.1
G6N56_RS13370	2873557	2874465		0	class I SAM-dependent methyltransferase	WP_085255764.1
G6N56_RS13375	2874582	2875082		0	nucleoside deaminase	WP_085255763.1
G6N56_RS13380	2875131	2875511		0	metalloregulator ArsR/SmtB family transcription factor	WP_085255762.1
G6N56_RS13385	2875548	2875751		0	zinc transporter Slc39a7	WP_085255761.1
G6N56_RS13390	2875776	2876801		0	oxygenase MpaB family protein	WP_085255760.1
G6N56_RS13395	2876866	2877480		0	TetR/AcrR family transcriptional regulator	WP_142280598.1
G6N56_RS13400	2877675	2878691		0	acyl-ACP desaturase	WP_085255759.1
G6N56_RS13405	2878698	2879831	dusB		tRNA dihydrouridine synthase DusB	WP_085255758.1
G6N56_RS13410	2880034	2882157		0	LCP family protein	WP_085255757.1
G6N56_RS13415	2882214	2882882	phoU		phosphate signaling complex protein PhoU	WP_085255756.1
G6N56_RS13420	2882887	2883663	pstB		phosphate ABC transporter ATP-binding protein PstB	WP_085255755.1
G6N56_RS13425	2883781	2884911	pstS		phosphate ABC transporter substrate-binding protein PstS	WP_232069292.1
G6N56_RS13430	2885020	2885940	mshD		mycothiol synthase	WP_085255753.1
G6N56_RS13435	2885937	2886713		0	response regulator transcription factor	WP_085255752.1
G6N56_RS13440	2886891	2887703	lmeA		mannan chain length control protein LmeA	WP_085255892.1
G6N56_RS13445	2887700	2888116		0	thioredoxin family protein	WP_085255751.1
G6N56_RS13450	2888338	2888811		0	DUF4395 domain-containing protein	WP_085255750.1
G6N56_RS13455	2888858	2889691		0	sulfurtransferase	WP_085255749.1
G6N56_RS13460	2889694	2889996		0	DUF1416 domain-containing protein	WP_085255748.1
G6N56_RS13465	2890142	2890810		0	FABP family protein	WP_085255747.1
G6N56_RS13470	2890894	2893266		0	hypothetical protein	WP_085255746.1
G6N56_RS13475	2893377	2894906		0	sensor domain-containing protein	WP_085255745.1
G6N56_RS13480	2895156	2896025		0	aminodeoxychorismate lyase	WP_142280611.1
G6N56_RS13485	2896092	2896670		0	hypothetical protein	WP_085255743.1
G6N56_RS13490	2896850	2897230		0	metal-sensitive transcriptional regulator	WP_085255742.1

G6N56_RS13495	2897240	2898334		0	folate-binding protein YgfZ	WP_085255741.1
G6N56_RS13500	2898480	2898665		0	DUF3073 domain-containing protein	WP_085255740.1
G6N56_RS13505	2898758	2899852	purM		phosphoribosylformylglycinamidine cyclo-ligase	WP_085255891.1
G6N56_RS13510	2899925	2901460	purF		amidophosphoribosyltransferase	WP_085255739.1
G6N56_RS13515	2901598	2901987		0	sterol carrier family protein	WP_085255738.1
G6N56_RS13520	2902007	2903212		0	MCE family protein	WP_085255737.1
G6N56_RS13525	2903225	2903845		0	CPBP family intramembrane metalloprotease	WP_142280610.1
G6N56_RS13530	2903842	2906139	purL		phosphoribosylformylglycinamidine synthase subunit PurL	WP_163645118.1
G6N56_RS13535	2906256	2907512		0	M18 family aminopeptidase	WP_085255889.1
G6N56_RS13540	2907635	2908651		0	Dyp-type peroxidase	WP_085255735.1
G6N56_RS13545	2908648	2909448		0	family 1 encapsulin nanocompartment shell protein	WP_085255734.1
G6N56_RS13550	2909647	2909817		0	hypothetical protein	WP_158090717.1
G6N56_RS13555	2909935	2910813		0	alpha/beta hydrolase	WP_085255732.1
G6N56_RS13560	2910885	2911604		0	helix-turn-helix domain-containing protein	WP_085255731.1
G6N56_RS13570	2912405	2913454		0	zinc-binding alcohol dehydrogenase family protein	WP_085255729.1
G6N56_RS13575	2913557	2914264		0	haloacid dehalogenase type II	WP_085255728.1
G6N56_RS13585	2915567	2916028		0	cupin domain-containing protein	WP_085255727.1
G6N56_RS13590	2916025	2916423		0	VOC family protein	WP_085255726.1
G6N56_RS13600	2917252	2917716		0	hypothetical protein	WP_085255725.1
G6N56_RS13605	2917730	2918404	purQ		phosphoribosylformylglycinamidine synthase subunit PurQ	WP_085255724.1
G6N56_RS13610	2918401	2918640	purS		phosphoribosylformylglycinamidine synthase subunit PurS	WP_085255723.1
G6N56_RS13620	2919411	2920049		0	MBL fold metallo-hydrolase	WP_085255721.1
G6N56_RS13625	2920079	2921755		0	FAD-binding dehydrogenase	WP_085255720.1
G6N56_RS13630	2921803	2922507		0	DUF2334 domain-containing protein	WP_085255719.1
G6N56_RS13635	2922693	2924306		0	DHA2 family efflux MFS transporter permease subunit	WP_142280597.1
G6N56_RS13640	2924307	2926454		0	S9 family peptidase	WP_085255717.1
G6N56_RS13645	2926451	2927356		0	phosphoribosylaminoimidazolesuccinocarboxamide synthase	WP_085255716.1
G6N56_RS13650	2927409	2928038		0	hypothetical protein	WP_085255715.1
G6N56_RS13655	2928065	2928829		0	class I SAM-dependent methyltransferase	WP_085255714.1
G6N56_RS13660	2928889	2930148		0	cytochrome P450	WP_085255713.1
G6N56_RS13665	2930145	2931563	purB		adenylosuccinate lyase	WP_085255712.1

G6N56_RS13675	2932530	2933165		0	TetR family transcriptional regulator	WP_180150567.1
G6N56_RS13680	2933221	2934135		0	alpha/beta hydrolase-fold protein	WP_085255709.1
G6N56_RS13685	2934166	2935749		0	gamma-glutamyltransferase family protein	WP_085255708.1
G6N56_RS13690	2935746	2937014	purD		phosphoribosylamine--glycine ligase	WP_085255707.1
G6N56_RS13695	2937064	2937468		0	MarR family transcriptional regulator	WP_085255706.1
G6N56_RS13700	2937550	2938635		0	aldo/keto reductase	WP_085255705.1
G6N56_RS13710	2939194	2939622		0	carboxymuconolactone decarboxylase family protein	WP_085255704.1
G6N56_RS13715	2939622	2940476		0	NAD(P)-dependent oxidoreductase	WP_085255703.1
G6N56_RS13720	2940473	2941222		0	SDR family oxidoreductase	WP_085255702.1
G6N56_RS13725	2941237	2942715		0	aldehyde dehydrogenase	WP_085255701.1
G6N56_RS13730	2942954	2943583		0	TetR/AcrR family transcriptional regulator	WP_232069342.1
G6N56_RS13735	2943580	2944806		0	cytochrome P450	WP_085255699.1
G6N56_RS13740	2944807	2945631		0	SDR family oxidoreductase	WP_085255698.1
G6N56_RS13745	2945628	2946983		0	cytochrome P450	WP_085255697.1
G6N56_RS13750	2946993	2947217		0	ferredoxin	WP_085255696.1
G6N56_RS13755	2947219	2947758		0	hypothetical protein	WP_085255695.1
G6N56_RS13760	2947928	2949055		0	NDMA-dependent alcohol dehydrogenase	WP_085255694.1
G6N56_RS13765	2949106	2949441		0	hypothetical protein	WP_264020485.1
G6N56_RS13770	2949456	2949881		0	ketosteroid isomerase family protein	WP_085255693.1
G6N56_RS13780	2950372	2951814		0	HAMP domain-containing sensor histidine kinase	WP_085255691.1
G6N56_RS13785	2951868	2952587	phoP		two-component system response regulator PhoP	WP_085255690.1
G6N56_RS13790	2952748	2953473		0	hypothetical protein	WP_085255888.1
G6N56_RS13805	2955850	2956863		0	helix-turn-helix domain-containing protein	WP_085255689.1
G6N56_RS13815	2957890	2959407		0	GMC family oxidoreductase N-terminal domain-containing prot	WP_197746672.1
G6N56_RS13830	2960950	2961135		0	transposase	WP_232069343.1
G6N56_RS13835	2961249	2961560		0	transposase	WP_085255685.1
G6N56_RS13840	2961691	2962845		0	acyl-CoA dehydrogenase family protein	WP_085255684.1
G6N56_RS13850	2963606	2965099		0	UbiD family decarboxylase	WP_085255682.1
G6N56_RS13855	2965099	2965701		0	UbiX family flavin prenyltransferase	WP_085255681.1
G6N56_RS13860	2966240	2966554		0	tautomerase family protein	WP_163645119.1
G6N56_RS13870	2967855	2968046		0	hypothetical protein	WP_085253900.1

G6N56_RS13875	2968196	2968729		0	TetR/AcrR family transcriptional regulator	WP_158090662.1
G6N56_RS13880	2968966	2969937		0	SDR family NAD(P)-dependent oxidoreductase	WP_197746668.1
G6N56_RS13885	2970040	2971200		0	IS3 family transposase	WP_158090661.1
G6N56_RS13890	2971497	2972729		0	LuxR C-terminal-related transcriptional regulator	WP_085253897.1
G6N56_RS13895	2972961	2974211		0	hypothetical protein	WP_085253896.1
G6N56_RS13900	2974588	2975859		0	acyl-CoA dehydrogenase family protein	WP_085253895.1
G6N56_RS13905	2975861	2976601		0	SDR family oxidoreductase	WP_085253894.1
G6N56_RS13915	2977757	2979286		0	long-chain fatty acid--CoA ligase	WP_085253893.1
G6N56_RS29090	2979294	2979782		0	SRPBCC family protein	WP_232069344.1
G6N56_RS13925	2980516	2981037		0	flavin reductase family protein	WP_158090660.1
G6N56_RS13940	2983407	2984483		0	LLM class flavin-dependent oxidoreductase	WP_085253891.1
G6N56_RS13945	2984483	2984989		0	SRPBCC family protein	WP_085253904.1
G6N56_RS13950	2985082	2985591		0	helix-turn-helix domain-containing protein	WP_085253890.1
G6N56_RS13955	2985916	2986710		0	DUF732 domain-containing protein	WP_085253888.1
G6N56_RS13960	2986873	2987145		0	hypothetical protein	WP_085253887.1
G6N56_RS13970	2988075	2988854		0	enoyl-CoA hydratase	WP_085253886.1
G6N56_RS28560	2989001	2989936		0	hypothetical protein	WP_180150327.1
G6N56_RS13980	2989978	2990442		0	lipoprotein LpqH	WP_085253885.1
G6N56_RS13985	2990604	2991248		0	S1 family peptidase	WP_085253902.1
G6N56_RS13990	2991552	2992973		0	DHA2 family efflux MFS transporter permease subunit	WP_085253884.1
G6N56_RS13995	2993018	2993419		0	nitroreductase family deazaflavin-dependent oxidoreductase	WP_085253883.1
G6N56_RS14000	2993564	2994439		0	alpha/beta hydrolase	WP_180150533.1
G6N56_RS29105	2994893	2995063		0	hypothetical protein	WP_211287370.1
G6N56_RS14010	2995228	2996376		0	TDT family transporter	WP_085253882.1
G6N56_RS14015	2996461	2996703		0	hypothetical protein	WP_085253881.1
G6N56_RS14020	2996978	2998522		0	LCP family protein	WP_085253880.1
G6N56_RS14025	2998525	2999316		0	SDR family NAD(P)-dependent oxidoreductase	WP_085253879.1
G6N56_RS14030	2999382	3000275		0	NAD(P)-dependent oxidoreductase	WP_085253878.1
G6N56_RS14040	3001243	3002175		0	enoyl-CoA hydratase	WP_085253876.1
G6N56_RS14045	3002266	3003057	fetB		iron export ABC transporter permease subunit FetB	WP_085253875.1
G6N56_RS14050	3003054	3003737		0	phosphate ABC transporter ATP-binding protein	WP_085253874.1

G6N56_RS14055	3003844	3004008		0	hypothetical protein	WP_142280413.1
G6N56_RS14060	3004009	3005502		0	trehalose-6-phosphate synthase	WP_085253873.1
G6N56_RS14065	3005569	3006144		0	hypothetical protein	WP_085253872.1
G6N56_RS14070	3006152	3006643		0	mammalian cell entry protein	WP_085253871.1
G6N56_RS14075	3006643	3007344		0	mammalian cell entry protein	WP_085253870.1
G6N56_RS14080	3007344	3009065		0	virulence factor Mce family protein	WP_085253869.1
G6N56_RS14085	3009062	3010243		0	virulence factor Mce family protein	WP_085253868.1
G6N56_RS14090	3010240	3011667		0	virulence factor Mce family protein	WP_085253867.1
G6N56_RS14095	3011664	3012752		0	virulence factor Mce family protein	WP_232069345.1
G6N56_RS14105	3013770	3014972		0	MCE family protein	WP_085253864.1
G6N56_RS14110	3014992	3015834		0	ABC transporter permease	WP_085253863.1
G6N56_RS14115	3015853	3016617		0	ABC transporter permease	WP_085253862.1
G6N56_RS14120	3016852	3017766		0	3-oxoacyl-ACP reductase	WP_085253861.1
G6N56_RS14125	3017801	3017992		0	ferredoxin	WP_085253860.1
G6N56_RS14130	3018183	3019364		0	acyl-CoA dehydrogenase	WP_085253859.1
G6N56_RS14135	3019390	3020487		0	acyl-CoA dehydrogenase family protein	WP_085253858.1
G6N56_RS14140	3020596	3022116	fadD17		long-chain-fatty-acid--CoA ligase FadD17	WP_085253857.1
G6N56_RS29475	3024321	3027983		0	PE family protein	WP_163645121.1
G6N56_RS14160	3028690	3030240		0	acetolactate synthase large subunit	WP_085255378.1
G6N56_RS14170	3031908	3032537		0	TetR/AcrR family transcriptional regulator	WP_197746628.1
G6N56_RS14175	3032571	3033395		0	amidohydrolase family protein	WP_085255375.1
G6N56_RS14180	3033425	3034192		0	fructosamine kinase family protein	WP_085255374.1
G6N56_RS14185	3034189	3035841		0	acyl-CoA synthetase	WP_085255373.1
G6N56_RS14190	3035906	3036727		0	crotonase/enoyl-CoA hydratase family protein	WP_085255372.1
G6N56_RS14195	3037072	3038292		0	cytochrome P450	WP_085255371.1
G6N56_RS14200	3038330	3039052		0	acetoacetate decarboxylase family protein	WP_085255415.1
G6N56_RS14205	3039113	3040144		0	LLM class F420-dependent oxidoreductase	WP_085255370.1
G6N56_RS14210	3040216	3041211		0	OB-fold nucleic acid binding domain-containing protein	WP_085255369.1
G6N56_RS14215	3041226	3042290		0	thiolase domain-containing protein	WP_085255368.1
G6N56_RS14220	3042290	3043489		0	thiolase domain-containing protein	WP_085255367.1
G6N56_RS14225	3043486	3043893		0	nuclear transport factor 2 family protein	WP_085255366.1

G6N56_RS14230	3043934	3044458		0	gamma carbonic anhydrase family protein	WP_085255365.1
G6N56_RS14235	3044570	3045727		0	Rieske 2Fe-2S domain-containing protein	WP_085255364.1
G6N56_RS14240	3045733	3046188		0	hypothetical protein	WP_085255363.1
G6N56_RS14245	3046190	3047338		0	sulfotransferase	WP_085255362.1
G6N56_RS14250	3047338	3048120		0	SDR family oxidoreductase	WP_085255361.1
G6N56_RS14255	3048123	3049250		0	hypothetical protein	WP_085255360.1
G6N56_RS14260	3049339	3049644		0	hypothetical protein	WP_085255359.1
G6N56_RS29120	3049695	3050867		0	PPE family protein	WP_085255358.1
G6N56_RS14270	3050991	3052046	dmpG		4-hydroxy-2-oxovalerate aldolase	WP_085255357.1
G6N56_RS14275	3052043	3052963		0	acetaldehyde dehydrogenase (acetylating)	WP_085255356.1
G6N56_RS14280	3053312	3054097		0	2-keto-4-pentenoate hydratase	WP_085255355.1
G6N56_RS14285	3054170	3055852	kstD		3-oxosteroid 1-dehydrogenase	WP_085255414.1
G6N56_RS14290	3055854	3056714		0	MaoC family dehydratase	WP_085255354.1
G6N56_RS14295	3056776	3057621		0	DUF559 domain-containing protein	WP_085255353.1
G6N56_RS14300	3057731	3058900		0	lipid-transfer protein	WP_085255352.1
G6N56_RS14305	3058900	3059301		0	MaoC family dehydratase	WP_085255351.1
G6N56_RS14310	3059298	3060257		0	bifunctional MaoC family dehydratase N-terminal/OB-fold nucl	WP_085255350.1
G6N56_RS14315	3060254	3061417		0	acyl-CoA dehydrogenase family protein	WP_085255349.1
G6N56_RS14325	3062441	3063682		0	cytochrome P450	WP_085255347.1
G6N56_RS14330	3063804	3064979		0	steroid 3-ketoacyl-CoA thiolase	WP_085255346.1
G6N56_RS14335	3065002	3065388		0	hypothetical protein	WP_085255345.1
G6N56_RS14340	3065397	3065843		0	nitroreductase family deazaflavin-dependent oxidoreductase	WP_085255344.1
G6N56_RS14345	3066080	3067270		0	cytochrome P450	WP_085255343.1
G6N56_RS14350	3067369	3068274		0	SDR family oxidoreductase	WP_085255342.1
G6N56_RS14355	3068289	3069047		0	SDR family oxidoreductase	WP_085255413.1
G6N56_RS14360	3069148	3069906		0	enoyl-CoA hydratase family protein	WP_085255341.1
G6N56_RS14365	3069903	3070787		0	CoA transferase subunit A	WP_085255340.1
G6N56_RS14370	3070784	3071527		0	CoA-transferase	WP_085255339.1
G6N56_RS14375	3071527	3072594		0	nitronate monooxygenase	WP_085255338.1
G6N56_RS14380	3072752	3073033		0	hypothetical protein	WP_085255337.1
G6N56_RS14385	3073043	3074203		0	acetyl-CoA C-acetyltransferase	WP_085255411.1

G6N56_RS14390	3074215	3074817	kstR2		TetR family transcriptional regulator KstR2	WP_142280551.1
G6N56_RS14395	3074992	3075156		0	hypothetical protein	WP_158090714.1
G6N56_RS14400	3075179	3075967		0	SDR family oxidoreductase	WP_085255336.1
G6N56_RS14405	3076088	3077236		0	acyl-CoA dehydrogenase family protein	WP_085255335.1
G6N56_RS14410	3077308	3078861	fadD3		3-((3aS,4S,7aS)-7a-methyl-1, 5-dioxo-octahydro-1H-inden-4-yl	WP_085255334.1
G6N56_RS14415	3078861	3079994		0	acyl-CoA dehydrogenase family protein	WP_085255333.1
G6N56_RS14420	3079991	3080941		0	acyl-CoA dehydrogenase family protein	WP_142280550.1
G6N56_RS14425	3080938	3081897		0	acyl-CoA dehydrogenase family protein	WP_085255332.1
G6N56_RS14430	3081894	3083063		0	pyridoxal phosphate-dependent aminotransferase	WP_085255331.1
G6N56_RS14440	3085037	3085867		0	arylamine N-acetyltransferase	WP_085255329.1
G6N56_RS14445	3085964	3086575		0	disulfide bond formation protein DsbA	WP_085255328.1
G6N56_RS14450	3086925	3087362		0	hypothetical protein	WP_085255327.1
G6N56_RS14455	3087499	3088062		0	flavin reductase family protein	WP_085255326.1
G6N56_RS14460	3088076	3088978		0	VOC family protein	WP_085255325.1
G6N56_RS14465	3088978	3089850		0	alpha/beta fold hydrolase	WP_085255324.1
G6N56_RS14470	3089847	3091031		0	flavin-dependent monooxygenase	WP_085255323.1
G6N56_RS14475	3091185	3092267		0	ferredoxin--NADP reductase	WP_085255322.1
G6N56_RS14480	3092320	3092853		0	hypothetical protein	WP_085255321.1
G6N56_RS14485	3093027	3093839		0	slipin family protein	WP_085255320.1
G6N56_RS14490	3093963	3096086		0	acyl-CoA dehydrogenase	WP_085255319.1
G6N56_RS14495	3096337	3096954	kstR		cholesterol catabolism transcriptional regulator KstR	WP_163645211.1
G6N56_RS14500	3097278	3098357		0	LacI family DNA-binding transcriptional regulator	WP_085255317.1
G6N56_RS14505	3098497	3099405		0	zinc ABC transporter substrate-binding protein	WP_085255409.1
G6N56_RS14510	3099413	3100237		0	metal ABC transporter ATP-binding protein	WP_085255316.1
G6N56_RS14515	3100234	3101094		0	metal ABC transporter permease	WP_085255315.1
G6N56_RS14520	3101118	3101876		0	5-oxoprolinase subunit PxpA	WP_085255314.1
G6N56_RS14525	3101876	3102796		0	MBL fold metallo-hydrolase	WP_085255313.1
G6N56_RS14530	3102809	3104050		0	ArsB/NhaD family transporter	WP_085255312.1
G6N56_RS14535	3104074	3105807		0	AMP-binding protein	WP_085255311.1
G6N56_RS14540	3105804	3106793		0	glycerophosphodiester phosphodiesterase	WP_142280561.1
G6N56_RS14545	3106828	3107757	rlmB		23S rRNA (guanosine(2251)-2'-O)-methyltransferase RlmB	WP_085255309.1

G6N56_RS14550	3107758	3109164	cysS		cysteine--tRNA ligase	WP_085255308.1
G6N56_RS14555	3109214	3109690	ispF		2-C-methyl-D-erythritol 2,4-cyclodiphosphate synthase	WP_085255307.1
G6N56_RS14560	3109687	3110373	ispD		2-C-methyl-D-erythritol 4-phosphate cytidylyltransferase	WP_085255306.1
G6N56_RS14565	3110390	3110878	carD		RNA polymerase-binding transcription factor CarD	WP_007166539.1
G6N56_RS14570	3111204	3111725		0	hypothetical protein	WP_085255305.1
G6N56_RS14575	3111774	3113201	radA		DNA repair protein RadA	WP_085255304.1
G6N56_RS14580	3113198	3114271	disA		DNA integrity scanning diadenylate cyclase DisA	WP_085255303.1
G6N56_RS14585	3114278	3115084		0	hypothetical protein	WP_142280548.1
G6N56_RS14590	3115190	3115807		0	carbonic anhydrase	WP_085255301.1
G6N56_RS14595	3115806	3116717		0	A/G-specific adenine glycosylase	WP_085255300.1
G6N56_RS14600	3116730	3118208		0	NADH-quinone oxidoreductase subunit C	WP_085255299.1
G6N56_RS14605	3118205	3119671		0	proton-conducting transporter membrane subunit	WP_085255298.1
G6N56_RS14610	3119671	3120333		0	hypothetical protein	WP_085255297.1
G6N56_RS14615	3120330	3121280		0	NADH-quinone oxidoreductase subunit H	WP_085255408.1
G6N56_RS14620	3121286	3123217		0	proton-conducting transporter membrane subunit	WP_142280560.1
G6N56_RS14625	3123259	3123738	nuoB		NADH-quinone oxidoreductase subunit NuoB	WP_085255295.1
G6N56_RS14630	3123744	3124079		0	lsr2/espR transcriptional regulator	WP_085255294.1
G6N56_RS14635	3124271	3125041		0	alpha/beta hydrolase	WP_085255293.1
G6N56_RS14645	3125400	3126764		0	serine hydrolase	WP_085255291.1
G6N56_RS14650	3127054	3127398		0	hypothetical protein	WP_142280547.1
G6N56_RS14655	3127395	3128639		0	HNH endonuclease signature motif containing protein	WP_085255288.1
G6N56_RS14660	3128755	3129297		0	histidine phosphatase family protein	WP_085255287.1
G6N56_RS14665	3129294	3130070		0	CbtA family protein	WP_085255286.1
G6N56_RS14670	3130080	3130292		0	CbtB-domain containing protein	WP_085255285.1
G6N56_RS14675	3130496	3133027	clpC1		ATP-dependent protease ATP-binding subunit ClpC	WP_085255284.1
G6N56_RS14680	3133342	3133680	lsr2		iron-regulated nucleoid-associated protein Lsr2	WP_066817684.1
G6N56_RS14685	3133807	3135303	lysS		lysine--tRNA ligase	WP_085255283.1
G6N56_RS14690	3135348	3136172		0	type III pantothenate kinase	WP_085255282.1
G6N56_RS14695	3136175	3136603		0	aspartate 1-decarboxylase	WP_085255281.1
G6N56_RS14700	3136603	3137544	panC		pantoate--beta-alanine ligase	WP_085255280.1
G6N56_RS14705	3137541	3138452		0	DUF2520 domain-containing protein	WP_085255279.1

G6N56_RS14710	3138549	3139937		0	hypothetical protein	WP_085255278.1
G6N56_RS14715	3140018	3140494		0	DUF3180 domain-containing protein	WP_085255277.1
G6N56_RS14720	3140494	3141042	folK		2-amino-4-hydroxy-6- hydroxymethyldihydropteridine diphosph	WP_085255276.1
G6N56_RS14730	3141433	3142266	folP		dihydropteroate synthase	WP_142280546.1
G6N56_RS14735	3142263	3142868	folE		GTP cyclohydrolase I FolE	WP_085255275.1
G6N56_RS14740	3142885	3145275	ftsH		ATP-dependent zinc metalloprotease FtsH	WP_085255274.1
G6N56_RS14745	3145425	3145841		0	VOC family protein	WP_085255273.1
G6N56_RS14750	3145956	3146924		0	alpha/beta hydrolase	WP_142280559.1
G6N56_RS14755	3146921	3148105		0	LLM class flavin-dependent oxidoreductase	WP_085255272.1
G6N56_RS14760	3148105	3149121		0	zinc-binding dehydrogenase	WP_085255271.1
G6N56_RS14765	3149134	3150240		0	MSMEG_0565 family glycosyltransferase	WP_085255404.1
G6N56_RS14770	3150243	3151088		0	carbon-nitrogen hydrolase family protein	WP_085255270.1
G6N56_RS14775	3151099	3152523		0	AIR synthase related protein	WP_085255269.1
G6N56_RS14780	3152523	3153578		0	MSMEG_0568 family radical SAM protein	WP_163645122.1
G6N56_RS14785	3153591	3154862		0	MSMEG_0569 family flavin-dependent oxidoreductase	WP_085255268.1
G6N56_RS14795	3155148	3156047		0	carbon-nitrogen hydrolase family protein	WP_085255266.1
G6N56_RS14800	3156088	3156609		0	MSMEG_0572 family nitrogen starvation response protein	WP_085255403.1
G6N56_RS14805	3157037	3158239		0	PPE family protein	WP_085255265.1
G6N56_RS14810	3158248	3158547		0	PE family protein	WP_085255264.1
G6N56_RS14815	3158839	3159582		0	SIMPL domain-containing protein	WP_085255263.1
G6N56_RS14820	3159587	3160240	hpt		hypoxanthine phosphoribosyltransferase	WP_142280544.1
G6N56_RS14830	3161189	3162238		0	zinc-dependent metalloprotease	WP_085255260.1
G6N56_RS14835	3162235	3163620	dacB		D-alanyl-D-alanine carboxypeptidase/D-alanyl-D-alanine-endo	WP_142280543.1
G6N56_RS14840	3163735	3164223		0	inorganic diphosphatase	WP_085255258.1
G6N56_RS14845	3164220	3165302		0	DUF475 domain-containing protein	WP_085255257.1
G6N56_RS14850	3165406	3166659		0	hypothetical protein	WP_085255256.1
G6N56_RS14855	3166662	3167375		0	glycosyltransferase family 2 protein	WP_142280557.1
G6N56_RS14860	3167372	3167713		0	DUF2304 domain-containing protein	WP_085255255.1
G6N56_RS14870	3168620	3170428		0	hypothetical protein	WP_163645124.1
G6N56_RS14880	3170804	3172024		0	DNA polymerase III subunit delta'	WP_085255254.1
G6N56_RS14885	3172180	3173808		0	adenylate/guanylate cyclase domain-containing protein	WP_085255253.1

G6N56_RS14890	3173812	3176622	topA		type I DNA topoisomerase	WP_085255252.1
G6N56_RS14895	3176817	3177407		0	hypothetical protein	WP_085255251.1
G6N56_RS14900	3177540	3177743	cspA		cold shock protein CspA	WP_007166602.1
G6N56_RS14905	3177992	3180319		0	DEAD/DEAH box helicase	WP_085255399.1
G6N56_RS14910	3180820	3181857		0	DUF5628 domain-containing protein	WP_085255250.1
G6N56_RS14915	3181854	3183404		0	radical SAM protein	WP_232069060.1
G6N56_RS14920	3183401	3183823		0	hypothetical protein	WP_142280542.1
G6N56_RS14925	3183820	3184617		0	prolipoprotein diacylglyceryl transferase	WP_085255248.1
G6N56_RS14930	3184627	3185037		0	flp pilus-assembly TadE/G-like family protein	WP_264020546.1
G6N56_RS14940	3185275	3185475		0	DUF4244 domain-containing protein	WP_085255246.1
G6N56_RS14945	3185494	3186090		0	type II secretion system F family protein	WP_180150335.1
G6N56_RS14950	3186087	3186890		0	type II secretion system F family protein	WP_085255245.1
G6N56_RS14955	3186887	3188062		0	TadA family conjugal transfer-associated ATPase	WP_085255244.1
G6N56_RS14960	3188059	3189123		0	CpaE-like family protein	WP_085255395.1
G6N56_RS14965	3189607	3190464		0	HAD-IB family hydrolase	WP_085255243.1
G6N56_RS14970	3190897	3191655		0	oxidoreductase	WP_085255242.1
G6N56_RS14975	3191667	3193301		0	ABC transporter ATP-binding protein	WP_085255394.1
G6N56_RS14985	3194166	3195092		0	ABC transporter permease	WP_085255240.1
G6N56_RS14990	3195094	3196707		0	ABC transporter substrate-binding protein	WP_085255239.1
G6N56_RS14995	3197228	3197668		0	DUF4232 domain-containing protein	WP_085255393.1
G6N56_RS15000	3197884	3199842	acs		acetate--CoA ligase	WP_085255238.1
G6N56_RS15005	3199844	3200542		0	serine protease	WP_142280541.1
G6N56_RS15010	3200731	3201273		0	phage holin family protein	WP_085255237.1
G6N56_RS15015	3201274	3202239		0	alpha/beta hydrolase	WP_085255236.1
G6N56_RS15020	3202245	3203438	marP		acid resistance serine protease MarP	WP_142280540.1
G6N56_RS15025	3203450	3204199		0	CoA pyrophosphatase	WP_142280555.1
G6N56_RS15030	3204264	3204905		0	TlpA disulfide reductase family protein	WP_085255233.1
G6N56_RS15035	3204915	3205652	nth		endonuclease III	WP_163645212.1
G6N56_RS15040	3205757	3206152		0	hypothetical protein	WP_142280539.1
G6N56_RS15045	3206250	3206924	crp		cAMP-activated global transcriptional regulator CRP	WP_044508824.1
G6N56_RS15050	3207032	3207823		0	MBL fold metallo-hydrolase	WP_085255231.1

G6N56_RS15055	3207829	3208284		0	RidA family protein	WP_085255230.1
G6N56_RS15060	3208285	3208446		0	DUF4177 domain-containing protein	WP_142280538.1
G6N56_RS15065	3208522	3209550		0	ArsA family ATPase	WP_085255229.1
G6N56_RS15070	3209547	3210677		0	ArsA family ATPase	WP_085255228.1
G6N56_RS15075	3210927	3211277		0	WhiB family transcriptional regulator	WP_085255390.1
G6N56_RS15080	3211638	3214064	ponA2		transglycosylase/D,D-transpeptidase PonA2	WP_085255227.1
G6N56_RS15085	3214129	3215088		0	metallophosphoesterase	WP_085255226.1
G6N56_RS15090	3215085	3216161		0	PLP-dependent cysteine synthase family protein	WP_180150338.1
G6N56_RS15100	3216375	3216719		0	metalloregulator ArsR/SmtB family transcription factor	WP_085255225.1
G6N56_RS15105	3216725	3218173	arsB		ACR3 family arsenite efflux transporter	WP_085255388.1
G6N56_RS15110	3218194	3218610		0	ArsI/CadI family heavy metal resistance metalloenzyme	WP_085255224.1
G6N56_RS15115	3218607	3219212		0	restriction endonuclease	WP_085255387.1
G6N56_RS15120	3219266	3220672		0	cytochrome P450	WP_142280537.1
G6N56_RS15125	3220712	3221008		0	DUF4193 domain-containing protein	WP_085255223.1
G6N56_RS15130	3221148	3221402		0	hypothetical protein	WP_142280536.1
G6N56_RS15135	3221460	3221768		0	hypothetical protein	WP_085255221.1
G6N56_RS15140	3221803	3222489		0	HAD family hydrolase	WP_085255220.1
G6N56_RS15145	3222501	3223001		0	DUF6328 family protein	WP_085255219.1
G6N56_RS15150	3223038	3223388		0	STAS domain-containing protein	WP_232069061.1
G6N56_RS15155	3223543	3224442		0	STAS domain-containing protein	WP_085255217.1
G6N56_RS15165	3224856	3225284		0	ATP-binding protein	WP_085255215.1
G6N56_RS15170	3225316	3225915		0	SpoIIE family protein phosphatase	WP_085255214.1
G6N56_RS15175	3225912	3228134		0	SpoIIE family protein phosphatase	WP_085255213.1
G6N56_RS15180	3228177	3229214		0	iron-containing redox enzyme family protein	WP_085255212.1
G6N56_RS15190	3229392	3230093		0	class I SAM-dependent methyltransferase	WP_264020410.1
G6N56_RS15195	3230199	3230753		0	type 1 glutamine amidotransferase domain-containing protein	WP_085255211.1
G6N56_RS15200	3230769	3231938		0	zinc-dependent alcohol dehydrogenase	WP_085255210.1
G6N56_RS15205	3231935	3232879		0	SRPBCC family protein	WP_085255209.1
G6N56_RS15210	3233056	3234042		0	LLM class F420-dependent oxidoreductase	WP_085255208.1
G6N56_RS15215	3234149	3235234	nrfD		polysulfide reductase NrfD	WP_085255207.1
G6N56_RS15220	3235242	3236213		0	4Fe-4S dicluster domain-containing protein	WP_085255383.1

G6N56_RS15230	3239799	3241139		0	PPE family protein	WP_085255204.1
G6N56_RS15235	3241136	3242539		0	PPE family protein	WP_232069063.1
G6N56_RS15250	3243389	3243688		0	PE family protein	WP_085255202.1
G6N56_RS15255	3244200	3245666		0	APC family permease	WP_085255201.1
G6N56_RS15260	3245899	3246360		0	Lrp/AsnC family transcriptional regulator	WP_085255381.1
G6N56_RS15265	3246582	3247409		0	polysaccharide deacetylase	WP_232069064.1
G6N56_RS15270	3247406	3248524		0	C45 family autoproteolytic acyltransferase/hydolase	WP_232069065.1
G6N56_RS15275	3248930	3249694		0	SDR family NAD(P)-dependent oxidoreductase	WP_085255199.1
G6N56_RS15280	3249691	3250743		0	aromatic ring-hydroxylating dioxygenase subunit alpha	WP_232069067.1
G6N56_RS15290	3251793	3253295		0	gamma-aminobutyraldehyde dehydrogenase	WP_085255196.1
G6N56_RS15295	3253683	3255068		0	aspartate aminotransferase family protein	WP_085255195.1
G6N56_RS15300	3255127	3256755		0	PucR family transcriptional regulator	WP_085255194.1
G6N56_RS15305	3256905	3258398		0	CoA-acylating methylmalonate-semialdehyde dehydrogenase	WP_085255193.1
G6N56_RS15310	3258410	3259810		0	aspartate aminotransferase family protein	WP_085255380.1
G6N56_RS15315	3259876	3260991	ald		alanine dehydrogenase	WP_085255192.1
G6N56_RS15320	3261111	3261602		0	Lrp/AsnC family transcriptional regulator	WP_085255191.1
G6N56_RS15330	3262953	3263945	selD		selenide, water dikinase SelD	WP_085258401.1
G6N56_RS15345	3265363	3267057		0	selenocysteine-specific translation elongation factor	WP_085258239.1
G6N56_RS15350	3267062	3269221		0	FUSC family protein	WP_085258240.1
G6N56_RS15355	3269271	3269735		0	GatB/YqeY domain-containing protein	WP_085258241.1
G6N56_RS15360	3269807	3270952		0	hypothetical protein	WP_085258402.1
G6N56_RS15365	3270966	3271619		0	DUF4129 domain-containing protein	WP_085258242.1
G6N56_RS15370	3271616	3272755		0	DUF4350 domain-containing protein	WP_232069068.1
G6N56_RS15375	3272752	3273738		0	MoxR family ATPase	WP_085258244.1
G6N56_RS15380	3273812	3275134		0	DUF58 domain-containing protein	WP_085258245.1
G6N56_RS15385	3275139	3276140		0	stage II sporulation protein M	WP_085258246.1
G6N56_RS15400	3277249	3277641		0	hypothetical protein	WP_085258249.1
G6N56_RS15405	3277846	3278583		0	PadR family transcriptional regulator	WP_085258250.1
G6N56_RS15410	3278690	3280237	glpK		glycerol kinase GlpK	WP_085258251.1
G6N56_RS15415	3280300	3281784		0	glutamate--cysteine ligase	WP_085258252.1
G6N56_RS15420	3281807	3282496		0	class I SAM-dependent methyltransferase	WP_085258253.1

G6N56_RS15425	3282609	3283754	egtE	ergothioneine biosynthesis PLP-dependent enzyme EgtE	WP_085258254.1
G6N56_RS15430	3283751	3284716	egtD	L-histidine N(alpha)-methyltransferase	WP_085258255.1
G6N56_RS15435	3284732	3285415	egtC	ergothioneine biosynthesis protein EgtC	WP_085258256.1
G6N56_RS15440	3285415	3286728	egtB	ergothioneine biosynthesis protein EgtB	WP_085258257.1
G6N56_RS15450	3288154	3288801	0	sensor domain-containing protein	WP_142280844.1
G6N56_RS15455	3288896	3290338	0	catalase	WP_085258259.1
G6N56_RS15460	3290489	3290908	0	hypothetical protein	WP_085258260.1
G6N56_RS15465	3291080	3291448	0	hypothetical protein	WP_232069349.1
G6N56_RS15470	3291733	3292866	0	DUF4185 domain-containing protein	WP_085258262.1
G6N56_RS15475	3292874	3293920	0	aspartate-semialdehyde dehydrogenase	WP_085258263.1
G6N56_RS15480	3293921	3295186	0	aspartate kinase	WP_085258264.1
G6N56_RS15485	3295492	3297294	leuA	2-isopropylmalate synthase	WP_163645126.1
G6N56_RS15490	3297298	3298290	0	DEDDh family exonuclease	WP_085258265.1
G6N56_RS15495	3298363	3299592	0	Mur ligase family protein	WP_085258266.1
G6N56_RS15505	3300452	3301060	0	C39 family peptidase	WP_085258268.1
G6N56_RS15510	3301065	3301676	recR	recombination mediator RecR	WP_085258269.1
G6N56_RS15515	3301690	3302046	0	YbaB/EbfC family nucleoid-associated protein	WP_085258270.1
G6N56_RS15520	3302145	3302906	0	Rv3717 family N-acetylmuramoyl-L-alanine amidase	WP_085258271.1
G6N56_RS15525	3303023	3303460	0	SRPBCC family protein	WP_085258272.1
G6N56_RS15530	3303584	3304918	0	FAD-binding oxidoreductase	WP_180150340.1
G6N56_RS15535	3304915	3306222	0	class I SAM-dependent methyltransferase	WP_197746630.1
G6N56_RS15540	3306599	3308032	0	DNA polymerase III subunits gamma/tau	WP_232069070.1
G6N56_RS15545	3308161	3309453	0	aminotransferase class I/II-fold pyridoxal phosphate-dependen	WP_142280832.1
G6N56_RS15560	3309887	3310690	0	hypothetical protein	WP_085258275.1
G6N56_RS15565	3310707	3311081	0	hypothetical protein	WP_085258276.1
G6N56_RS15570	3311222	3311710	0	lipoprotein LpqH	WP_085258277.1
G6N56_RS15575	3311806	3313518	0	hypothetical protein	WP_085258278.1
G6N56_RS15580	3313515	3314867	0	CHAT domain-containing protein	WP_085258279.1
G6N56_RS15585	3314871	3315185	0	hypothetical protein	WP_142280833.1
G6N56_RS15590	3315269	3316600	0	hypothetical protein	WP_085258281.1
G6N56_RS15595	3316597	3317736	0	hypothetical protein	WP_085258282.1

G6N56_RS15600	3317803	3318861		0	dihydrodipicolinate reductase	WP_085258283.1
G6N56_RS15605	3318858	3319016		0	hypothetical protein	WP_163645068.1
G6N56_RS15610	3319084	3319257		0	CsbD family protein	WP_066868284.1
G6N56_RS15615	3319326	3319973		0	cutinase family protein	WP_142280846.1
G6N56_RS15620	3320005	3320988		0	SDR family NAD(P)-dependent oxidoreductase	WP_085258407.1
G6N56_RS15625	3320997	3321383		0	PPOX class F420-dependent oxidoreductase	WP_085258285.1
G6N56_RS15630	3321490	3322053		0	TetR/AcrR family transcriptional regulator	WP_085258286.1
G6N56_RS15640	3323348	3323929		0	TetR/AcrR family transcriptional regulator	WP_085258408.1
G6N56_RS15645	3324023	3324535		0	PaaI family thioesterase	WP_085258287.1
G6N56_RS15650	3324569	3325342		0	SDR family NAD(P)-dependent oxidoreductase	WP_085258288.1
G6N56_RS15655	3325348	3325965		0	isochorismatase family cysteine hydrolase	WP_085258289.1
G6N56_RS15660	3326067	3327419		0	hypothetical protein	WP_085258290.1
G6N56_RS15670	3328218	3329243	ligD		non-homologous end-joining DNA ligase	WP_085258292.1
G6N56_RS15675	3329281	3330360		0	ATP-dependent DNA ligase	WP_085258293.1
G6N56_RS15680	3330447	3330815		0	hypothetical protein	WP_085258294.1
G6N56_RS15685	3330898	3332130		0	cytochrome P450	WP_085258295.1
G6N56_RS15690	3332161	3333066		0	haloalkane dehalogenase	WP_085258296.1
G6N56_RS15695	3333070	3333555		0	NUDIX domain-containing protein	WP_085258297.1
G6N56_RS15700	3333559	3334914		0	wax ester/triacylglycerol synthase family O-acyltransferase	WP_085258298.1
G6N56_RS15705	3334955	3336031		0	SDR family oxidoreductase	WP_085258299.1
G6N56_RS15710	3336050	3337852		0	long-chain fatty acid--CoA ligase	WP_085258300.1
G6N56_RS15715	3337981	3338379		0	DUF1330 domain-containing protein	WP_232069071.1
G6N56_RS15720	3338376	3338789		0	hypothetical protein	WP_085258301.1
G6N56_RS15725	3338956	3340047		0	dihydrodipicolinate reductase	WP_085258302.1
G6N56_RS15730	3340044	3340817		0	SDR family NAD(P)-dependent oxidoreductase	WP_085258303.1
G6N56_RS15735	3340820	3341422		0	TetR/AcrR family transcriptional regulator	WP_085258410.1
G6N56_RS29480	3341514	3341819		0	hypothetical protein	WP_180150324.1
G6N56_RS15745	3341992	3342963		0	LLM class flavin-dependent oxidoreductase	WP_085258305.1
G6N56_RS15750	3342965	3343444		0	VOC family protein	WP_085258411.1
G6N56_RS15755	3343470	3344219		0	SDR family oxidoreductase	WP_085258306.1
G6N56_RS15760	3344216	3345472		0	MBL fold metallo-hydrolase	WP_232069072.1

G6N56_RS15765	3345492	3346757	0	sulfotransferase	WP_085258307.1
G6N56_RS15770	3346853	3347239	0	DUF2237 domain-containing protein	WP_085258308.1
G6N56_RS15775	3347425	3347808	0	hypothetical protein	WP_085258309.1
G6N56_RS15780	3348047	3349204	0	cellulase family glycosylhydrolase	WP_085258310.1
G6N56_RS15785	3349345	3349587	0	hypothetical protein	WP_142280837.1
G6N56_RS15795	3349763	3350242	0	nucleoside deaminase	WP_085258413.1
G6N56_RS15805	3350353	3350874	0	tRNA adenosine deaminase-associated protein	WP_085258311.1
G6N56_RS15810	3351034	3351972	0	prephenate dehydrogenase	WP_264020804.1
G6N56_RS15825	3353259	3353945	0	ABC transporter permease	WP_085258315.1
G6N56_RS15830	3353948	3355057	0	ABC transporter ATP-binding protein	WP_085258414.1
G6N56_RS15835	3355057	3356004	0	ABC transporter substrate-binding protein	WP_085258316.1
G6N56_RS15840	3356096	3356464	0	lipopolysaccharide assembly protein LapA domain-containing p	WP_085258317.1
G6N56_RS15845	3356479	3357531	0	phosphotransferase family protein	WP_085258318.1
G6N56_RS15850	3357567	3358175	0	histidine phosphatase family protein	WP_085258319.1
G6N56_RS15855	3358261	3359292	0	phosphotransferase family protein	WP_085258415.1
G6N56_RS15860	3359294	3360052	0	glucose 1-dehydrogenase	WP_085258320.1
G6N56_RS15865	3360070	3361353	0	acyl-CoA dehydrogenase family protein	WP_085258321.1
G6N56_RS15875	3361957	3362928	0	NADPH:quinone oxidoreductase family protein	WP_085258323.1
G6N56_RS15880	3363015	3364895	0	alkyl sulfatase dimerization domain-containing protein	WP_163645128.1
G6N56_RS15885	3365057	3365542	0	lipoprotein LpqH	WP_085258325.1
G6N56_RS15890	3365539	3367065	0	HAMP domain-containing sensor histidine kinase	WP_085258326.1
G6N56_RS28425	3367899	3368054	0	hypothetical protein	WP_169717555.1
G6N56_RS15900	3368080	3368322	0	hypothetical protein	WP_085258328.1
G6N56_RS15905	3368870	3369280	0	MarR family transcriptional regulator	WP_142280840.1
G6N56_RS15910	3369357	3370304	0	zinc-binding dehydrogenase	WP_085258330.1
G6N56_RS15915	3370301	3371245	0	class I SAM-dependent methyltransferase	WP_085258331.1
G6N56_RS15920	3371514	3372512	0	phosphotransferase family protein	WP_085258416.1
G6N56_RS15925	3372566	3373063	0	hypothetical protein	WP_085258332.1
G6N56_RS15930	3373093	3373713	0	hypothetical protein	WP_085258333.1
G6N56_RS28430	3374282	3374425	0	hypothetical protein	WP_169717556.1
G6N56_RS15940	3374574	3374912	0	DUF732 domain-containing protein	WP_085258334.1

G6N56_RS28435	3374987	3375148		0	hypothetical protein	WP_169717557.1
G6N56_RS15945	3375308	3375769		0	nitroreductase family deazaflavin-dependent oxidoreductase	WP_085258335.1
G6N56_RS15960	3376547	3377617		0	pyridoxal phosphate-dependent aminotransferase	WP_085258336.1
G6N56_RS15965	3377614	3378075		0	DUF4334 domain-containing protein	WP_085258337.1
G6N56_RS15970	3378162	3378746		0	TIGR03086 family metal-binding protein	WP_180150344.1
G6N56_RS15975	3378770	3379594		0	crotonase/enoyl-CoA hydratase family protein	WP_085258339.1
G6N56_RS15980	3379624	3380859	lipE		lipase LipE	WP_085258340.1
G6N56_RS15985	3380888	3381079		0	hypothetical protein	WP_085258341.1
G6N56_RS15990	3381170	3382288		0	hypothetical protein	WP_085258342.1
G6N56_RS16000	3382416	3383384		0	NAD(P)H-quinone oxidoreductase	WP_085258343.1
G6N56_RS16005	3383489	3384685		0	cysteine desulfurase-like protein	WP_085258344.1
G6N56_RS16010	3384805	3386775		0	hypothetical protein	WP_085258345.1
G6N56_RS16025	3388173	3389099		0	glycosyltransferase family 2 protein	WP_085258347.1
G6N56_RS16030	3389096	3389926		0	ABC transporter permease	WP_085258348.1
G6N56_RS16035	3389970	3390341		0	hypothetical protein	WP_142280843.1
G6N56_RS16040	3390452	3390676		0	hypothetical protein	WP_085258350.1
G6N56_RS16045	3390732	3391871		0	flotillin family protein	WP_085258351.1
G6N56_RS16050	3391874	3392794		0	ribonuclease Z	WP_085258352.1
G6N56_RS16055	3392867	3393343		0	GreA/GreB family elongation factor	WP_085258353.1
G6N56_RS16060	3393385	3393825		0	GtrA family protein	WP_085258354.1
G6N56_RS16065	3393858	3395240		0	FAD-binding oxidoreductase	WP_085258355.1
G6N56_RS16070	3395252	3396016		0	decaprenylphospho-beta-D-erythro-pentofuranosid- 2-ulose 2-	WP_085258356.1
G6N56_RS16075	3396019	3397893		0	galactan 5-O-arabinofuranosyltransferase	WP_085258357.1
G6N56_RS16080	3397900	3401178		0	arabinosyltransferase domain-containing protein	WP_085258358.1
G6N56_RS16085	3401271	3404594		0	arabinosyltransferase domain-containing protein	WP_085258359.1
G6N56_RS16090	3404591	3407851		0	arabinosyltransferase domain-containing protein	WP_085258360.1
G6N56_RS16095	3408024	3408542		0	hypothetical protein	WP_085258361.1
G6N56_RS16100	3408767	3409198		0	hypothetical protein	WP_169717558.1
G6N56_RS29485	3409209	3409688		0	FHA domain-containing protein	WP_085258363.1
G6N56_RS16110	3409708	3410475		0	Crp/Fnr family transcriptional regulator	WP_085258364.1
G6N56_RS16115	3410502	3412274		0	acyl-CoA dehydrogenase family protein	WP_085258365.1

G6N56_RS16120	3412393	3413259		0	cyclopropane mycolic acid synthase family methyltransferase	WP_085258366.1
G6N56_RS16125	3413268	3414215		0	SDR family NAD(P)-dependent oxidoreductase	WP_085258367.1
G6N56_RS16130	3414329	3415198		0	helix-turn-helix transcriptional regulator	WP_085258368.1
G6N56_RS16135	3415252	3416826		0	acyl-CoA carboxylase subunit beta	WP_142280850.1
G6N56_RS16145	3422253	3424148	fadD32		long-chain-fatty-acid--AMP ligase FadD32	WP_085258371.1
G6N56_RS16150	3424417	3425427		0	cutinase family protein	WP_085258372.1
G6N56_RS16155	3425683	3426591		0	alpha/beta hydrolase family protein	WP_085258373.1
G6N56_RS16160	3426748	3427785	ag85A		diacylglycerol acyltransferase/mycolyltransferase Ag85A	WP_085258374.1
G6N56_RS16165	3428168	3430138		0	terminal beta-(1->2)-arabinofuranosyltransferase	WP_085258375.1
G6N56_RS16180	3431575	3433470		0	glycosyltransferase	WP_085258377.1
G6N56_RS16185	3433467	3434663	glf		UDP-galactopyranose mutase	WP_085258378.1
G6N56_RS16190	3434931	3436025		0	PirG	WP_180150348.1
G6N56_RS16195	3436187	3437833		0	LGFP repeat-containing protein	WP_180150569.1
G6N56_RS29140	3437879	3439804		0	alanine racemase	WP_085258381.1
G6N56_RS16210	3439898	3441445		0	PE family protein	WP_085258420.1
G6N56_RS16215	3441551	3442021		0	DUF350 domain-containing protein	WP_085258382.1
G6N56_RS16220	3442068	3443234		0	glutathionylspermidine synthase family protein	WP_085258383.1
G6N56_RS16225	3443231	3443905		0	hypothetical protein	WP_232069073.1
G6N56_RS16230	3444017	3444832		0	Cof-type HAD-IIB family hydrolase	WP_085258384.1
G6N56_RS16235	3444864	3445649		0	lysophospholipid acyltransferase family protein	WP_085258385.1
G6N56_RS16240	3445667	3446422		0	lysophospholipid acyltransferase family protein	WP_085258386.1
G6N56_RS16245	3446429	3447187		0	1-acyl-sn-glycerol-3-phosphate acyltransferase	WP_085258387.1
G6N56_RS16250	3447327	3448877		0	MBL fold metallo-hydrolase	WP_085258388.1
G6N56_RS16255	3448874	3449218		0	hypothetical protein	WP_085258389.1
G6N56_RS16260	3449243	3450799		0	NAD(P)/FAD-dependent oxidoreductase	WP_085258390.1
G6N56_RS16265	3450861	3451475		0	TetR/AcrR family transcriptional regulator	WP_085258391.1
G6N56_RS16270	3451547	3452032		0	DUF2834 domain-containing protein	WP_085258392.1
G6N56_RS16275	3452029	3452616		0	class I SAM-dependent methyltransferase	WP_085258422.1
G6N56_RS16280	3452672	3453418		0	helix-turn-helix transcriptional regulator	WP_085258393.1
G6N56_RS16285	3453469	3454728	serS		serine--tRNA ligase	WP_085258394.1
G6N56_RS16290	3454841	3456199		0	septum formation family protein	WP_085258395.1

G6N56_RS16295	3456203	3456553		0	metallopeptidase family protein	WP_085258396.1
G6N56_RS16300	3456601	3456795		0	hypothetical protein	WP_085258397.1
G6N56_RS16305	3456803	3457498		0	histidine phosphatase family protein	WP_085258398.1
G6N56_RS16310	3457495	3458439	pheA		prephenate dehydratase	WP_085258399.1
G6N56_RS16315	3458531	3459307		0	DUF2470 domain-containing protein	WP_085258400.1
G6N56_RS16320	3459329	3460531		0	LCP family protein	WP_163645129.1
G6N56_RS16325	3460538	3461362		0	glycerophosphodiester phosphodiesterase	WP_085258438.1
G6N56_RS16330	3461368	3462444		0	DUF4328 domain-containing protein	WP_085258439.1
G6N56_RS28440	3462944	3463099		0	hypothetical protein	WP_169717559.1
G6N56_RS16335	3463277	3463900		0	superoxide dismutase	WP_085258440.1
G6N56_RS16340	3464064	3464597		0	peptidase	WP_085258441.1
G6N56_RS16350	3465890	3466288		0	transcriptional regulator	WP_085258453.1
G6N56_RS16355	3466423	3467079		0	DUF6474 family protein	WP_085258442.1
G6N56_RS16360	3467127	3467741		0	YcnI family protein	WP_085258454.1
G6N56_RS16365	3467738	3468238		0	copper resistance protein CopC	WP_085258443.1
G6N56_RS29490	3468329	3468790		0	nucleoid-structuring protein H-NS	WP_085258444.1
G6N56_RS16375	3468792	3469271	rraA		ribonuclease E activity regulator RraA	WP_085258445.1
G6N56_RS16380	3469268	3470737		0	NAD(P)/FAD-dependent oxidoreductase	WP_085258446.1
G6N56_RS16385	3470814	3471455		0	TetR/AcrR family transcriptional regulator	WP_085258447.1
G6N56_RS16390	3471480	3472679	dinB		DNA polymerase IV	WP_085258448.1
G6N56_RS16400	3473250	3474257		0	PHP domain-containing protein	WP_085258449.1
G6N56_RS16405	3474281	3474517		0	hypothetical protein	WP_085258455.1
G6N56_RS16410	3474570	3475427		0	LLM class F420-dependent oxidoreductase	WP_085258450.1
G6N56_RS16415	3475494	3476885		0	PPE family protein	WP_232069074.1
G6N56_RS16420	3477127	3477423		0	PE family protein	WP_085257424.1
G6N56_RS16425	3477427	3478086		0	CoA-transferase	WP_085257425.1
G6N56_RS16430	3478095	3478829		0	hypothetical protein	WP_232069075.1
G6N56_RS16435	3478923	3481235		0	hydantoinase B/oxoprolinase family protein	WP_085257427.1
G6N56_RS16440	3481274	3483403		0	hydantoinase/oxoprolinase family protein	WP_085257428.1
G6N56_RS16445	3483431	3483859		0	acetone carboxylase subunit gamma	WP_085257429.1
G6N56_RS16450	3483900	3485813		0	hydantoinase/oxoprolinase family protein	WP_085257430.1

G6N56_RS16455	3486019	3487869		0	helix-turn-helix domain-containing protein	WP_085257431.1
G6N56_RS16465	3489745	3494322	gltB		glutamate synthase large subunit	WP_085257433.1
G6N56_RS16475	3494963	3496183		0	MinD/ParA family protein	WP_180150353.1
G6N56_RS16480	3496342	3496623		0	WhiB family transcriptional regulator	WP_085254127.1
G6N56_RS16485	3496771	3497061		0	WhiB family transcriptional regulator	WP_085254128.1
G6N56_RS16490	3497562	3498296		0	transcriptional regulator	WP_085254129.1
G6N56_RS16495	3498651	3500018		0	EspA/EspE family type VII secretion system effector	WP_085254130.1
G6N56_RS16500	3500093	3500410		0	ESX-1 secretion-associated protein	WP_085254131.1
G6N56_RS16505	3500413	3501264		0	ESX secretion-associated protein EspG	WP_085254132.1
G6N56_RS16510	3501299	3501913		0	hypothetical protein	WP_085254184.1
G6N56_RS16515	3501965	3503686	eccA		type VII secretion AAA-ATPase EccA	WP_085254133.1
G6N56_RS16520	3503690	3505138	eccB		type VII secretion protein EccB	WP_085254134.1
G6N56_RS16525	3505138	3507384	eccCa		type VII secretion protein EccCa	WP_085254135.1
G6N56_RS16530	3507381	3509135	eccCb		type VII secretion protein EccCb	WP_085254136.1
G6N56_RS16535	3509405	3510628		0	PPE family protein	WP_085254137.1
G6N56_RS16540	3510717	3511019		0	WXG100 family type VII secretion target	WP_085254138.1
G6N56_RS16545	3511060	3511347		0	WXG100 family type VII secretion target	WP_085254139.1
G6N56_RS16550	3511448	3512965		0	MinD/ParA family protein	WP_085254140.1
G6N56_RS16555	3512962	3514509	eccD		type VII secretion integral membrane protein EccD	WP_085254141.1
G6N56_RS16560	3514598	3515323		0	hypothetical protein	WP_085254142.1
G6N56_RS29495	3515365	3517602		0	hypothetical protein	WP_085254143.1
G6N56_RS16570	3517740	3519263		0	hypothetical protein	WP_085254144.1
G6N56_RS16575	3519558	3520268		0	FkbM family methyltransferase	WP_085254145.1
G6N56_RS16580	3520335	3521111		0	glycosyltransferase	WP_085254146.1
G6N56_RS16585	3521121	3522161		0	DUF4254 domain-containing protein	WP_158090675.1
G6N56_RS16590	3522158	3523219		0	glycosyltransferase family 9 protein	WP_085254148.1
G6N56_RS16600	3524201	3524998		0	sulfotransferase	WP_211287375.1
G6N56_RS16605	3525052	3525402		0	YbaB/EbfC family nucleoid-associated protein	WP_085254151.1
G6N56_RS16615	3526930	3528276	mycP		type VII secretion-associated serine protease mycosin	WP_085254185.1
G6N56_RS16625	3529727	3531589		0	alpha/beta hydrolase	WP_163645132.1
G6N56_RS16630	3531598	3532104		0	pullulanase	WP_085254153.1

G6N56_RS16635	3532124	3533575		0	CCA tRNA nucleotidyltransferase	WP_085254154.1
G6N56_RS16640	3533716	3534447		0	NUDIX hydrolase	WP_085254155.1
G6N56_RS16645	3534444	3536858		0	hypothetical protein	WP_085254156.1
G6N56_RS16650	3536855	3540529	murJ		murein biosynthesis integral membrane protein MurJ	WP_085254157.1
G6N56_RS16655	3540594	3541166	sigM		RNA polymerase sigma factor SigM	WP_085254158.1
G6N56_RS16660	3541212	3541976		0	hypothetical protein	WP_085254159.1
G6N56_RS16665	3542058	3543068	trxB		thioredoxin-disulfide reductase	WP_085254160.1
G6N56_RS16670	3543065	3543418	trxA		thioredoxin	WP_085254161.1
G6N56_RS16675	3543552	3544772		0	N-acetylmuramoyl-L-alanine amidase	WP_085254162.1
G6N56_RS16685	3545787	3546770		0	ParB/RepB/Spo0J family partition protein	WP_085254164.1
G6N56_RS16695	3547752	3548474	rsmG		16S rRNA (guanine(527)-N(7))-methyltransferase RsmG	WP_085254166.1
G6N56_RS16700	3548525	3549103		0	R3H domain-containing nucleic acid-binding protein	WP_085254167.1
G6N56_RS16705	3549178	3550287	yidC		membrane protein insertase YidC	WP_085254168.1
G6N56_RS16715	3550603	3550971	rnpA		ribonuclease P protein component	WP_085254170.1
G6N56_RS16720	3550992	3551135	rpmH		50S ribosomal protein L34	WP_085254171.1
G6N56_RS16725	3551756	3553282	dnaA		chromosomal replication initiator protein DnaA	WP_085254187.1
G6N56_RS16730	3553769	3554968	dnaN		DNA polymerase III subunit beta	WP_085254172.1
G6N56_RS16735	3554970	3556127	recF		DNA replication/repair protein RecF	WP_085254173.1
G6N56_RS16740	3556142	3556669		0	DUF721 family protein	WP_085254188.1
G6N56_RS16745	3556917	3558929	gyrB		DNA topoisomerase (ATP-hydrolyzing) subunit B	WP_085254189.1
G6N56_RS16750	3558969	3561488	gyrA		DNA gyrase subunit A	WP_085254174.1
G6N56_RS16755	3561619	3562518		0	DUF3566 domain-containing protein	WP_085254175.1
G6N56_RS16780	3563221	3563655	cwsA		cell wall synthesis protein CwsA	WP_085254176.1
G6N56_RS16785	3563792	3564340		0	peptidylprolyl isomerase	WP_085254177.1
G6N56_RS16790	3564348	3564773		0	PH domain-containing protein	WP_085254178.1
G6N56_RS16795	3564929	3565210	crgA		cell division protein CrgA	WP_085254179.1
G6N56_RS16800	3565351	3566103		0	DUF881 domain-containing protein	WP_085254180.1
G6N56_RS16810	3566807	3568684	pknB		Stk1 family PASTA domain-containing Ser/Thr kinase	WP_085254190.1
G6N56_RS16815	3568681	3570036		0	serine/threonine-protein kinase	WP_232069077.1
G6N56_RS16820	3570033	3571511	pbpA		D,D-transpeptidase PbpA	WP_085254182.1
G6N56_RS16825	3571508	3572917		0	FtsW/RodA/SpoVE family cell cycle protein	WP_085254183.1

G6N56_RS16830	3572914	3574440		0	protein phosphatase 2C domain-containing protein	WP_163645133.1
G6N56_RS16835	3574437	3574904		0	FHA domain-containing protein	WP_085257434.1
G6N56_RS16840	3575028	3576662		0	DUF3662 and FHA domain-containing protein	WP_163645134.1
G6N56_RS16850	3577318	3578172		0	hypothetical protein	WP_142280755.1
G6N56_RS16855	3578644	3580248		0	amino acid permease	WP_197746631.1
G6N56_RS16860	3580310	3581287		0	alpha/beta fold hydrolase	WP_158090752.1
G6N56_RS16865	3581380	3582114		0	TetR/AcrR family transcriptional regulator	WP_158090753.1
G6N56_RS16870	3582143	3583471	glnT		type III glutamate--ammonia ligase	WP_142280760.1
G6N56_RS16880	3584136	3585119		0	pirin family protein	WP_085257440.1
G6N56_RS16885	3585171	3586553		0	acyl-CoA dehydrogenase family protein	WP_085257441.1
G6N56_RS16890	3586619	3587035		0	WhiB family transcriptional regulator	WP_085257442.1
G6N56_RS16895	3587179	3587967		0	helix-turn-helix transcriptional regulator	WP_085257443.1
G6N56_RS16900	3588024	3588773		0	C40 family peptidase	WP_085257477.1
G6N56_RS16905	3588770	3589132		0	DUF4226 domain-containing protein	WP_085257444.1
G6N56_RS16910	3589249	3590625		0	DUF4226 domain-containing protein	WP_085257445.1
G6N56_RS16915	3590669	3590986		0	ESX-1 secretion-associated protein	WP_085257446.1
G6N56_RS16920	3590983	3591288		0	DUF2694 family protein	WP_085257447.1
G6N56_RS28450	3591399	3591542		0	hypothetical protein	WP_169717544.1
G6N56_RS16925	3591624	3593000		0	DUF5631 domain-containing protein	WP_085257448.1
G6N56_RS16930	3593066	3593383		0	DUF2710 family protein	WP_085257449.1
G6N56_RS16935	3593576	3594349		0	TIGR03084 family metal-binding protein	WP_085257450.1
G6N56_RS16940	3594386	3595669		0	MFS transporter	WP_085257451.1
G6N56_RS16945	3595773	3596426		0	YqgE/AlgH family protein	WP_142280756.1
G6N56_RS16950	3596430	3596762		0	hypothetical protein	WP_085257452.1
G6N56_RS16955	3596831	3597919		0	LpqN/LpqT family lipoprotein	WP_085257453.1
G6N56_RS16960	3598106	3601003	leuS		leucine--tRNA ligase	WP_085257454.1
G6N56_RS16965	3601025	3601696		0	SDR family oxidoreductase	WP_085257455.1
G6N56_RS16970	3601734	3602240		0	MarR family transcriptional regulator	WP_085257456.1
G6N56_RS16975	3602303	3603052		0	amino acid ABC transporter ATP-binding protein	WP_085257457.1
G6N56_RS16980	3603049	3604845		0	ABC transporter substrate-binding protein/permease	WP_085257458.1
G6N56_RS16985	3604894	3605634		0	GntR family transcriptional regulator	WP_085257459.1

G6N56_RS16990	3605755	3606549		0	LLM class F420-dependent oxidoreductase	WP_085257460.1
G6N56_RS16995	3606565	3607479		0	alpha/beta hydrolase	WP_085257461.1
G6N56_RS17000	3607605	3608711		0	inositol-3-phosphate synthase	WP_085257462.1
G6N56_RS17005	3608787	3609323		0	PadR family transcriptional regulator	WP_085257463.1
G6N56_RS17010	3609459	3610337		0	DUF1707 domain-containing protein	WP_085257464.1
G6N56_RS17015	3610446	3610868		0	DUF5318 domain-containing protein	WP_085257480.1
G6N56_RS17020	3610966	3613413		0	transglycosylase domain-containing protein	WP_232069078.1
G6N56_RS17025	3613437	3615104		0	glycosyltransferase family 87 protein	WP_085257479.1
G6N56_RS17030	3615308	3616270		0	phosphate/phosphite/phosphonate ABC transporter substrate-	WP_142280757.1
G6N56_RS17035	3616267	3617055	phnC		phosphonate ABC transporter ATP-binding protein	WP_085257482.1
G6N56_RS17040	3617052	3618683	phnE		phosphonate ABC transporter, permease protein PhnE	WP_085257466.1
G6N56_RS17045	3618783	3619550		0	GntR family transcriptional regulator	WP_085257483.1
G6N56_RS17055	3619971	3620552	phnH		phosphonate C-P lyase system protein PhnH	WP_085257468.1
G6N56_RS17060	3620552	3621661		0	carbon-phosphorus lyase complex subunit PhnI	WP_085257469.1
G6N56_RS17065	3621658	3623487		0	alpha-D-ribose 1-methylphosphonate 5-phosphate C-P-lyase P	WP_142280758.1
G6N56_RS17070	3623493	3624227		0	ATP-binding cassette domain-containing protein	WP_085257485.1
G6N56_RS17075	3624230	3625423		0	alpha-D-ribose 1-methylphosphonate 5-triphosphate diphosph	WP_142280759.1
G6N56_RS17080	3625420	3626241		0	inositol monophosphatase family protein	WP_085257471.1
G6N56_RS17085	3626278	3626808		0	HD domain-containing protein	WP_085257472.1
G6N56_RS17090	3626850	3627488		0	DJ-1/PfpI family protein	WP_085257473.1
G6N56_RS17095	3627601	3627891	rpsF		30S ribosomal protein S6	WP_068070741.1
G6N56_RS17100	3627963	3628487		0	single-stranded DNA-binding protein	WP_163645136.1
G6N56_RS17105	3628532	3628789	rpsR		30S ribosomal protein S18	WP_085256564.1
G6N56_RS17110	3628814	3629272	rplI		50S ribosomal protein L9	WP_085256563.1
G6N56_RS17115	3629806	3631182	dnaB		replicative DNA helicase	WP_085256562.1
G6N56_RS17120	3631270	3631971		0	alpha/beta hydrolase	WP_158090729.1
G6N56_RS17125	3633105	3634367		0	condensation domain-containing protein	WP_163645137.1
G6N56_RS17130	3634948	3635397		0	helix-turn-helix domain-containing protein	WP_085256559.1
G6N56_RS17135	3635686	3636159		0	DoxX family protein	WP_085256558.1
G6N56_RS17145	3637195	3638223		0	HAMP domain-containing sensor histidine kinase	WP_085256556.1
G6N56_RS17155	3639209	3639658		0	hypothetical protein	WP_142280668.1

G6N56_RS17160	3639715	3640170		0	DM13 domain-containing protein	WP_085256553.1
G6N56_RS17165	3640567	3641010	msrB		peptide-methionine (R)-S-oxide reductase MsrB	WP_232069353.1
G6N56_RS17170	3641462	3642013	msrA		peptide-methionine (S)-S-oxide reductase MsrA	WP_264020405.1
G6N56_RS17175	3642149	3643897		0	cytochrome c biogenesis protein CcdA	WP_085256551.1
G6N56_RS17180	3644030	3644782		0	SDR family oxidoreductase	WP_085256550.1
G6N56_RS17185	3644895	3645497		0	TetR/AcrR family transcriptional regulator	WP_085256549.1
G6N56_RS17190	3645788	3646405		0	TetR/AcrR family transcriptional regulator	WP_085256548.1
G6N56_RS17195	3646498	3647010		0	hypothetical protein	WP_085256547.1
G6N56_RS17200	3647119	3647730		0	hypothetical protein	WP_085256546.1
G6N56_RS17205	3648289	3648807		0	hypothetical protein	WP_085256545.1
G6N56_RS17210	3648921	3649856		0	NAD-dependent epimerase/dehydratase family protein	WP_085256544.1
G6N56_RS17215	3649950	3650591		0	TetR/AcrR family transcriptional regulator	WP_085256543.1
G6N56_RS17220	3650724	3651542		0	IclR family transcriptional regulator	WP_085256542.1
G6N56_RS17225	3651597	3652730		0	LLM class flavin-dependent oxidoreductase	WP_085256541.1
G6N56_RS17230	3652748	3653509		0	SDR family oxidoreductase	WP_085256540.1
G6N56_RS17235	3653561	3654877		0	bifunctional 3,4-dihydroxy-2-butanone-4-phosphate synthase/(	WP_085256539.1
G6N56_RS17245	3655827	3656516		0	class I SAM-dependent methyltransferase	WP_085256537.1
G6N56_RS17250	3656536	3657534		0	DMT family transporter	WP_180150355.1
G6N56_RS17255	3657690	3658541		0	LysR family transcriptional regulator	WP_085256535.1
G6N56_RS17260	3658544	3658921		0	hypothetical protein	WP_085256575.1
G6N56_RS17265	3659008	3659727		0	CoA pyrophosphatase	WP_085256534.1
G6N56_RS29500	3659735	3659866		0	hypothetical protein	WP_264020402.1
G6N56_RS17270	3660103	3660531		0	hypothetical protein	WP_085256533.1
G6N56_RS28455	3660890	3661057		0	hypothetical protein	WP_169717530.1
G6N56_RS17275	3661534	3662382		0	class I SAM-dependent methyltransferase	WP_232069354.1
G6N56_RS17280	3662502	3662840		0	DUF732 domain-containing protein	WP_085256531.1
G6N56_RS17285	3662863	3663690		0	SDR family NAD(P)-dependent oxidoreductase	WP_085256530.1
G6N56_RS17290	3663707	3664567		0	LLM class F420-dependent oxidoreductase	WP_085256529.1
G6N56_RS17295	3664587	3665021		0	MarR family transcriptional regulator	WP_085256528.1
G6N56_RS17300	3665106	3665528		0	nitroreductase family deazaflavin-dependent oxidoreductase	WP_085256527.1
G6N56_RS17310	3665915	3666856		0	2-hydroxyacid dehydrogenase	WP_085256526.1

G6N56_RS17315	3666876	3667868		0	Lrp/AsnC family transcriptional regulator	WP_232069080.1
G6N56_RS17320	3667989	3668732		0	SDR family NAD(P)-dependent oxidoreductase	WP_085256525.1
G6N56_RS17325	3668755	3669612		0	LLM class flavin-dependent oxidoreductase	WP_085256524.1
G6N56_RS17330	3669750	3670607		0	LLM class F420-dependent oxidoreductase	WP_085256523.1
G6N56_RS17335	3670801	3671619		0	formate/nitrite transporter family protein	WP_085256573.1
G6N56_RS17340	3671730	3672182		0	ATP-binding protein	WP_085256522.1
G6N56_RS17345	3672198	3673232		0	4Fe-4S binding protein	WP_085256521.1
G6N56_RS17350	3673286	3674641		0	wax ester/triacylglycerol synthase family O-acyltransferase	WP_085256520.1
G6N56_RS17355	3674756	3676756	malQ		4-alpha-glucanotransferase	WP_085256519.1
G6N56_RS17360	3677013	3677897		0	cyclopropane mycolic acid synthase family methyltransferase	WP_085256518.1
G6N56_RS17365	3678038	3678541		0	hypothetical protein	WP_180150357.1
G6N56_RS17370	3678696	3679049		0	VOC family protein	WP_085256516.1
G6N56_RS17375	3679222	3679488		0	YdeI/OmpD-associated family protein	WP_085256515.1
G6N56_RS17380	3679642	3680517		0	DMT family transporter	WP_085256514.1
G6N56_RS17385	3680857	3681684		0	alpha/beta hydrolase	WP_085256572.1
G6N56_RS17390	3681696	3682583		0	hypothetical protein	WP_085256513.1
G6N56_RS17395	3682645	3683220		0	hemerythrin domain-containing protein	WP_085256512.1
G6N56_RS17400	3683352	3684422		0	LLM class flavin-dependent oxidoreductase	WP_085256511.1
G6N56_RS17405	3684651	3685592		0	catalase family peroxidase	WP_085256510.1
G6N56_RS17410	3685827	3686876		0	2-dehydropantoate 2-reductase N-terminal domain-containing	WP_232069081.1
G6N56_RS17415	3686891	3687613		0	CPBP family intramembrane metalloprotease	WP_232069082.1
G6N56_RS17420	3687734	3688624		0	SAM-dependent methyltransferase	WP_085256507.1
G6N56_RS17425	3688799	3691726		0	ornithine decarboxylase	WP_085256506.1
G6N56_RS17430	3691723	3692646		0	SNG1 family protein	WP_232069083.1
G6N56_RS17435	3692883	3694193		0	MFS transporter	WP_232069084.1
G6N56_RS17440	3694435	3694710		0	hypothetical protein	WP_085256569.1
G6N56_RS17445	3694815	3695600		0	ABC transporter ATP-binding protein	WP_142280667.1
G6N56_RS17450	3695597	3696568		0	aliphatic sulfonate ABC transporter substrate-binding protein	WP_085256568.1
G6N56_RS17455	3696670	3697338		0	ABC transporter permease	WP_085256504.1
G6N56_RS17460	3697350	3699077	ctaD		cytochrome c oxidase subunit I	WP_085256503.1
G6N56_RS17465	3699254	3700477		0	DUF4185 domain-containing protein	WP_163645138.1

G6N56_RS17475	3700972	3701928		0	FAD-binding oxidoreductase	WP_085256501.1
G6N56_RS17480	3701950	3703167		0	GNAT family N-acetyltransferase	WP_085256500.1
G6N56_RS17485	3703214	3704425		0	acyl-CoA dehydrogenase family protein	WP_085256499.1
G6N56_RS17490	3704439	3705050		0	TetR/AcrR family transcriptional regulator	WP_085256498.1
G6N56_RS17495	3705328	3706656		0	HAMP domain-containing sensor histidine kinase	WP_085256567.1
G6N56_RS17500	3706903	3708462		0	SulP family inorganic anion transporter	WP_085256497.1
G6N56_RS17505	3708469	3708849		0	hypothetical protein	WP_232069085.1
G6N56_RS17510	3708860	3710227		0	aromatic ring-hydroxylating dioxygenase subunit alpha	WP_085256496.1
G6N56_RS17515	3710243	3712003		0	amidohydrolase family protein	WP_085256495.1
G6N56_RS17520	3712206	3712655		0	ATP-binding protein	WP_085256494.1
G6N56_RS17525	3712719	3713096		0	STAS domain-containing protein	WP_085256493.1
G6N56_RS17530	3713616	3714647		0	catalase family peroxidase	WP_085256565.1
G6N56_RS17535	3714644	3715219		0	cytochrome b/b6 domain-containing protein	WP_085256492.1
G6N56_RS17540	3715265	3716158		0	fructose bisphosphate aldolase	WP_085256491.1
G6N56_RS17545	3716174	3717253		0	DUF190 domain-containing protein	WP_085256490.1
G6N56_RS17550	3717311	3717910		0	TetR/AcrR family transcriptional regulator	WP_085256489.1
G6N56_RS17555	3718089	3720314		0	acyl-CoA dehydrogenase	WP_085256488.1
G6N56_RS17560	3720330	3721502		0	sulfotransferase	WP_085256487.1
G6N56_RS17565	3721499	3722602		0	DUF1214 domain-containing protein	WP_085256486.1
G6N56_RS17570	3722620	3723237		0	TetR family transcriptional regulator	WP_232069086.1
G6N56_RS17575	3723259	3724713		0	aldehyde dehydrogenase family protein	WP_085256484.1
G6N56_RS17585	3726141	3727478		0	amidohydrolase family protein	WP_085256482.1
G6N56_RS17590	3727482	3729806		0	CoA transferase	WP_085256481.1
G6N56_RS17600	3730127	3731293		0	thiolase family protein	WP_085256479.1
G6N56_RS17605	3731290	3731706		0	Zn-ribbon domain-containing OB-fold protein	WP_085256478.1
G6N56_RS17615	3732295	3733932	fadD1		fatty-acid--CoA ligase FadD1	WP_085256476.1
G6N56_RS17620	3734001	3734762		0	SDR family oxidoreductase	WP_085256475.1
G6N56_RS17630	3736190	3736975		0	helix-turn-helix domain-containing protein	WP_232069087.1
G6N56_RS17635	3737069	3738703		0	cation acetate symporter	WP_085257752.1
G6N56_RS17640	3738703	3739050		0	DUF485 domain-containing protein	WP_142280791.1
G6N56_RS17645	3739413	3740516		0	carbon-nitrogen hydrolase family protein	WP_232069088.1

G6N56_RS17650	3740513	3741223	0	FCD domain-containing protein	WP_085257754.1
G6N56_RS17655	3741407	3742606	0	CoA transferase	WP_085257755.1
G6N56_RS17660	3742603	3744135	0	carboxyl transferase domain-containing protein	WP_085257756.1
G6N56_RS17665	3744235	3744504	0	hypothetical protein	WP_085257757.1
G6N56_RS17670	3744582	3746330	0	helix-turn-helix domain-containing protein	WP_085257758.1
G6N56_RS17675	3746457	3747950	0	CoA-acylating methylmalonate-semialdehyde dehydrogenase	WP_085257759.1
G6N56_RS17680	3747959	3748294	0	nitroreductase family deazaflavin-dependent oxidoreductase	WP_232069355.1
G6N56_RS17690	3749138	3752197	0	RND family transporter	WP_085257762.1
G6N56_RS17695	3752294	3752992	0	TetR/AcrR family transcriptional regulator	WP_085257763.1
G6N56_RS17700	3753026	3753622	0	hypothetical protein	WP_085257764.1
G6N56_RS17705	3753801	3754757	0	polysaccharide deacetylase family protein	WP_085257765.1
G6N56_RS17710	3754897	3756288	0	alpha/beta hydrolase-fold protein	WP_085257766.1
G6N56_RS17715	3756401	3758632	0	heavy metal translocating P-type ATPase	WP_085257767.1
G6N56_RS17720	3758629	3758841	0	heavy metal-associated domain-containing protein	WP_085257768.1
G6N56_RS17725	3758925	3759746	0	mycofactocin-coupled SDR family oxidoreductase	WP_085257769.1
G6N56_RS17730	3759840	3760313	0	SRPBCC family protein	WP_085257770.1
G6N56_RS17740	3760609	3762417	0	NAD(P)/FAD-dependent oxidoreductase	WP_085257771.1
G6N56_RS17745	3762414	3764078	0	NAD(P)/FAD-dependent oxidoreductase	WP_085257772.1
G6N56_RS17750	3764212	3765609	0	DUF2252 domain-containing protein	WP_085257773.1
G6N56_RS17755	3765776	3766843	0	sulfate ABC transporter substrate-binding protein	WP_232069089.1
G6N56_RS17760	3766862	3768637	0	SulP family inorganic anion transporter	WP_085257775.1
G6N56_RS17765	3768646	3769473	0	SDR family oxidoreductase	WP_085257776.1
G6N56_RS17770	3769533	3770828	0	sodium:proton exchanger	WP_085257777.1
G6N56_RS29505	3771378	3772325	0	LysR family transcriptional regulator	WP_085257778.1
G6N56_RS17780	3772417	3773826	0	MmgE/PrpD family protein	WP_085257779.1
G6N56_RS17785	3773876	3774646	0	DUF2127 domain-containing protein	WP_142280793.1
G6N56_RS17790	3774674	3775516	0	SDR family oxidoreductase	WP_085257781.1
G6N56_RS17795	3775659	3775823	0	hypothetical protein	WP_232069090.1
G6N56_RS17800	3775836	3776447	0	class I SAM-dependent methyltransferase	WP_085257783.1
G6N56_RS17805	3776458	3777060	0	CGNR zinc finger domain-containing protein	WP_169717546.1
G6N56_RS17810	3777146	3777838	0	SDR family oxidoreductase	WP_085257797.1

G6N56_RS17815	3777894	3778349	0	SRPBCC family protein	WP_085257784.1
G6N56_RS17820	3778406	3779143	0	methyltransferase domain-containing protein	WP_085257798.1
G6N56_RS17825	3779263	3780639	0	adenylate/guanylate cyclase domain-containing protein	WP_085257785.1
G6N56_RS17830	3780700	3781275	0	TetR/AcrR family transcriptional regulator	WP_085257786.1
G6N56_RS17835	3781375	3782313	0	oxygenase MpaB family protein	WP_085257787.1
G6N56_RS17840	3782571	3783665	0	AMP-binding protein	WP_085257788.1
G6N56_RS17845	3783701	3785065	0	aldehyde dehydrogenase family protein	WP_085257799.1
G6N56_RS17855	3785606	3786034	0	cupin domain-containing protein	WP_158090762.1
G6N56_RS17860	3786148	3786846	0	cupin domain-containing protein	WP_232069091.1
G6N56_RS17865	3786843	3787577	0	cutinase family protein	WP_085257792.1
G6N56_RS17870	3787773	3788663	0	SDR family NAD(P)-dependent oxidoreductase	WP_085257793.1
G6N56_RS17875	3788660	3790258	0	AMP-binding protein	WP_085257794.1
G6N56_RS17890	3796345	3797292	0	sigma-70 family RNA polymerase sigma factor	WP_085254296.1
G6N56_RS17895	3797449	3798219	0	SDR family oxidoreductase	WP_085254297.1
G6N56_RS17900	3798252	3798989	0	DUF899 domain-containing protein	WP_085254424.1
G6N56_RS17905	3799084	3799317	0	hypothetical protein	WP_085254298.1
G6N56_RS17910	3799338	3799724	0	nuclear transport factor 2 family protein	WP_232069092.1
G6N56_RS17915	3799769	3800635	0	rhomboid family intramembrane serine protease	WP_085254300.1
G6N56_RS17920	3800632	3801093	0	SRPBCC family protein	WP_085254301.1
G6N56_RS17925	3801104	3801877	0	SDR family oxidoreductase	WP_085254302.1
G6N56_RS17930	3801879	3803468	0	AMP-binding protein	WP_085254303.1
G6N56_RS17935	3803478	3804239	0	GntR family transcriptional regulator	WP_085254425.1
G6N56_RS17940	3804245	3805807	0	SMP-30/gluconolactonase/LRE family protein	WP_085254426.1
G6N56_RS17950	3806870	3808237	0	aromatic ring-hydroxylating dioxygenase subunit alpha	WP_232069093.1
G6N56_RS17955	3808234	3809406	0	LLM class flavin-dependent oxidoreductase	WP_085254305.1
G6N56_RS17960	3809403	3811256	0	NAD(P)/FAD-dependent oxidoreductase	WP_085254306.1
G6N56_RS17970	3812179	3813381	0	CoA transferase	WP_085254307.1
G6N56_RS17975	3813608	3815611	0	acyltransferase family protein	WP_085254308.1
G6N56_RS17980	3815608	3816267	0	class I SAM-dependent methyltransferase	WP_085254309.1
G6N56_RS17985	3816738	3816926	0	hypothetical protein	WP_142280456.1
G6N56_RS17990	3816970	3818691	0	divalent metal cation transporter	WP_085254311.1

G6N56_RS18000	3819746	3820345		0	hypothetical protein	WP_085254313.1
G6N56_RS18005	3820357	3823851		0	carboxylic acid reductase	WP_142280461.1
G6N56_RS18010	3824029	3824811		0	L,D-transpeptidase	WP_085254315.1
G6N56_RS18015	3824978	3825625		0	isochorismatase family cysteine hydrolase	WP_085254316.1
G6N56_RS18020	3825702	3826634		0	LysR family transcriptional regulator	WP_085254429.1
G6N56_RS18025	3826646	3828355	oxc		oxalyl-CoA decarboxylase	WP_085254317.1
G6N56_RS18030	3828559	3830145		0	FadD7 family fatty acid--CoA ligase	WP_085254318.1
G6N56_RS18035	3830151	3832310		0	elongation factor G-like protein EF-G2	WP_085254319.1
G6N56_RS18040	3832451	3832858		0	TIGR03668 family PPOX class F420-dependent oxidoreductase	WP_085254320.1
G6N56_RS18045	3833004	3833471		0	nuclear transport factor 2 family protein	WP_085254321.1
G6N56_RS18050	3833618	3834691		0	S1C family serine protease	WP_085254430.1
G6N56_RS18055	3834813	3836588	treS		maltose alpha-D-glucosyltransferase	WP_085254431.1
G6N56_RS18060	3836585	3837937		0	maltokinase	WP_085254322.1
G6N56_RS18065	3838012	3838707		0	YoaK family protein	WP_085254323.1
G6N56_RS18070	3838768	3839769	ag85C		diacylglycerol acyltransferase/mycolyltransferase Ag85C	WP_085254432.1
G6N56_RS18075	3839933	3840679		0	ABC transporter permease	WP_232069357.1
G6N56_RS18080	3840750	3841577		0	ATP-binding cassette domain-containing protein	WP_085254324.1
G6N56_RS18085	3841574	3842026		0	MarR family transcriptional regulator	WP_180150359.1
G6N56_RS18090	3842093	3842590		0	metallophosphoesterase	WP_085254434.1
G6N56_RS28460	3842630	3842803		0	hypothetical protein	WP_169717514.1
G6N56_RS18095	3842925	3843380		0	MaoC family dehydratase	WP_085254326.1
G6N56_RS18100	3843459	3844754		0	acyl-CoA dehydrogenase family protein	WP_085254435.1
G6N56_RS18105	3844849	3845550		0	GAF and ANTAR domain-containing protein	WP_085254327.1
G6N56_RS18110	3845684	3846580		0	manganese catalase family protein	WP_085254328.1
G6N56_RS18115	3846630	3847208		0	hemerythrin domain-containing protein	WP_085254329.1
G6N56_RS18120	3847321	3848412		0	F420-dependent hydroxymycolic acid dehydrogenase	WP_085254330.1
G6N56_RS18125	3848465	3849070		0	GNAT family N-acetyltransferase	WP_085254331.1
G6N56_RS18130	3849117	3849482		0	hypothetical protein	WP_085254332.1
G6N56_RS18135	3849558	3850232		0	TetR family transcriptional regulator	WP_085254333.1
G6N56_RS18140	3850303	3851373		0	ferredoxin reductase	WP_085254334.1
G6N56_RS18145	3851405	3852508		0	acyl-CoA desaturase	WP_085254335.1

G6N56_RS18150	3852544	3853059		0	SRPBCC family protein	WP_085254336.1
G6N56_RS18155	3853098	3854210		0	DUF1214 domain-containing protein	WP_085254337.1
G6N56_RS18160	3854207	3854782		0	TetR/AcrR family transcriptional regulator	WP_085254437.1
G6N56_RS18165	3854882	3856048		0	sulfotransferase	WP_232069358.1
G6N56_RS18170	3856059	3856949		0	alpha/beta hydrolase	WP_085254338.1
G6N56_RS18175	3857079	3858122		0	zinc-binding dehydrogenase	WP_085254339.1
G6N56_RS18180	3858125	3858337		0	TOBE domain-containing protein	WP_085254438.1
G6N56_RS18185	3858375	3858971		0	TetR/AcrR family transcriptional regulator	WP_085254439.1
G6N56_RS18190	3859111	3860433		0	cytochrome P450	WP_085254440.1
G6N56_RS18195	3860452	3860961	msrA		peptide-methionine (S)-S-oxide reductase MsrA	WP_085254340.1
G6N56_RS18200	3861053	3861559		0	nuclear transport factor 2 family protein	WP_085254341.1
G6N56_RS18205	3861556	3862608		0	NAD-dependent epimerase/dehydratase family protein	WP_085254342.1
G6N56_RS18215	3863052	3863453		0	nuclear transport factor 2 family protein	WP_085254344.1
G6N56_RS18220	3863512	3864426		0	DNA-3-methyladenine glycosylase	WP_085254345.1
G6N56_RS18225	3864505	3865056		0	DNA starvation/stationary phase protection protein	WP_085254346.1
G6N56_RS18230	3865079	3866545		0	chloride channel protein	WP_085254347.1
G6N56_RS18235	3866657	3867640		0	pirin family protein	WP_085254441.1
G6N56_RS18240	3867643	3868278		0	TetR/AcrR family transcriptional regulator	WP_085254348.1
G6N56_RS18245	3868348	3869289		0	class I SAM-dependent methyltransferase	WP_085254349.1
G6N56_RS18250	3869322	3870254		0	class I SAM-dependent methyltransferase	WP_142280464.1
G6N56_RS18255	3870255	3871178		0	class I SAM-dependent methyltransferase	WP_085254351.1
G6N56_RS18260	3871265	3872698		0	aldehyde dehydrogenase family protein	WP_085254352.1
G6N56_RS18265	3872749	3873609		0	SDR family oxidoreductase	WP_085254353.1
G6N56_RS18270	3873615	3874583		0	NADPH:quinone oxidoreductase family protein	WP_085254354.1
G6N56_RS18275	3874593	3876383		0	PE-PPE domain-containing protein	WP_085254355.1
G6N56_RS18280	3876451	3878175		0	PE-PPE domain-containing protein	WP_085254356.1
G6N56_RS18285	3878324	3880063		0	PE-PPE domain-containing protein	WP_085254442.1
G6N56_RS18290	3880191	3881009		0	tyrosine-protein phosphatase	WP_085254357.1
G6N56_RS18295	3881011	3882222		0	acyl-CoA dehydrogenase family protein	WP_163645220.1
G6N56_RS18300	3882612	3883703		0	Re/Si-specific NAD(P)(+) transhydrogenase subunit alpha	WP_085254358.1
G6N56_RS18305	3883706	3884041		0	NAD(P) transhydrogenase subunit alpha	WP_085254359.1

G6N56_RS18310	3884038	3885462		0	NAD(P)(+) transhydrogenase (Re/Si-specific) subunit beta	WP_085254360.1
G6N56_RS18315	3885598	3885723		0	hypothetical protein	WP_090350544.1
G6N56_RS18325	3886533	3887981		0	PE-PPE domain-containing protein	WP_085254362.1
G6N56_RS18330	3888092	3888988		0	alpha/beta fold hydrolase	WP_085254363.1
G6N56_RS18335	3889095	3890195		0	diacylglycerol kinase	WP_085254364.1
G6N56_RS18340	3890211	3890951		0	glucose 1-dehydrogenase	WP_085254365.1
G6N56_RS18345	3890998	3892347		0	FAD-binding oxidoreductase	WP_085254366.1
G6N56_RS18350	3892344	3892895		0	TetR/AcrR family transcriptional regulator	WP_085254367.1
G6N56_RS18355	3892927	3893757		0	thioesterase family protein	WP_085254368.1
G6N56_RS18360	3893774	3894589		0	SMP-30/gluconolactonase/LRE family protein	WP_085254369.1
G6N56_RS18365	3894723	3895604		0	thioesterase family protein	WP_085254444.1
G6N56_RS18370	3895610	3896530		0	LLM class F420-dependent oxidoreductase	WP_085254370.1
G6N56_RS18375	3896531	3897688		0	acyl-CoA dehydrogenase family protein	WP_085254371.1
G6N56_RS18380	3897776	3898504		0	ZIP family metal transporter	WP_085254372.1
G6N56_RS18385	3898684	3899349		0	nitroreductase family protein	WP_085254373.1
G6N56_RS18390	3899630	3901483		0	FAD-linked oxidase C-terminal domain-containing protein	WP_085254374.1
G6N56_RS18395	3901485	3903086	mdlC		benzoylformate decarboxylase	WP_085254375.1
G6N56_RS18400	3903127	3903285		0	hypothetical protein	WP_232069094.1
G6N56_RS18405	3903550	3904722		0	helix-turn-helix domain-containing protein	WP_085254376.1
G6N56_RS18415	3905423	3906739		0	amidohydrolase family protein	WP_085254378.1
G6N56_RS18420	3906802	3907701		0	hypothetical protein	WP_085254379.1
G6N56_RS18425	3907874	3909070		0	PucR family transcriptional regulator	WP_085254380.1
G6N56_RS29510	3909263	3909388		0	hypothetical protein	WP_264020428.1
G6N56_RS18430	3909396	3910883		0	LuxR C-terminal-related transcriptional regulator	WP_158090679.1
G6N56_RS18435	3910895	3911578		0	DUF427 domain-containing protein	WP_085254382.1
G6N56_RS18445	3912068	3913249		0	CoA transferase	WP_085254383.1
G6N56_RS18450	3913406	3913993		0	hypothetical protein	WP_085254384.1
G6N56_RS18455	3914073	3915266		0	enolase C-terminal domain-like protein	WP_085254385.1
G6N56_RS18460	3915297	3916604		0	hydroxyacid-oxoacid transhydrogenase	WP_085254386.1
G6N56_RS18465	3916609	3917079		0	thioesterase family protein	WP_085254387.1
G6N56_RS18470	3917135	3917575		0	SRPBCC family protein	WP_085254388.1

G6N56_RS18475	3917583	3918275		0	GntR family transcriptional regulator	WP_085254389.1
G6N56_RS18480	3918414	3920063	fadD5		fatty-acid--CoA ligase FadD5	WP_085254390.1
G6N56_RS18485	3920267	3921046		0	ABC transporter permease	WP_180150577.1
G6N56_RS18490	3921048	3921917		0	ABC transporter permease	WP_085254392.1
G6N56_RS18495	3921922	3923244		0	MCE family protein	WP_085254393.1
G6N56_RS18500	3923241	3924281		0	virulence factor Mce family protein	WP_085254394.1
G6N56_RS18505	3924278	3925870		0	virulence factor Mce family protein	WP_085254395.1
G6N56_RS18510	3925867	3927438		0	virulence factor Mce family protein	WP_085254396.1
G6N56_RS18520	3928709	3930283		0	MlaD family protein	WP_085254398.1
G6N56_RS18530	3930990	3931955		0	RDD family protein	WP_085254400.1
G6N56_RS18540	3932473	3933123		0	Mce protein	WP_085254445.1
G6N56_RS18550	3933793	3934911		0	phosphodiester glycosidase family protein	WP_142280458.1
G6N56_RS18555	3934991	3936373		0	YhgE/Pip domain-containing protein	WP_085254404.1
G6N56_RS18560	3936515	3937240		0	pirin-like bicupin family protein	WP_085254405.1
G6N56_RS18565	3937265	3938131		0	MarR family transcriptional regulator	WP_085254406.1
G6N56_RS18570	3938194	3939327		0	dihydrodipicolinate reductase	WP_085254407.1
G6N56_RS18575	3939769	3941145		0	alpha/beta hydrolase domain-containing protein	WP_085254408.1
G6N56_RS18580	3941142	3942185		0	sigma-70 family RNA polymerase sigma factor	WP_142280459.1
G6N56_RS18585	3942204	3943052		0	alpha/beta hydrolase	WP_085254410.1
G6N56_RS18590	3943194	3943943		0	DUF2786 domain-containing protein	WP_085254411.1
G6N56_RS18595	3943945	3944448		0	TIGR04338 family metallohydrolase	WP_085254446.1
G6N56_RS18600	3944783	3946240		0	PPE family protein	WP_085254412.1
G6N56_RS18605	3946237	3947409		0	FAD-dependent monooxygenase	WP_085254413.1
G6N56_RS18615	3948098	3950599		0	ABC transporter permease	WP_158090680.1
G6N56_RS18620	3950602	3951693		0	UbiA family prenyltransferase	WP_085254416.1
G6N56_RS18630	3952762	3955923		0	thiamine pyrophosphate-binding protein	WP_158090681.1
G6N56_RS18635	3955991	3957553		0	cytochrome P450	WP_232069095.1
G6N56_RS18640	3957586	3958152		0	TetR/AcrR family transcriptional regulator	WP_085254419.1
G6N56_RS29185	3958936	3959082		0	metallothionein	WP_085254449.1
G6N56_RS18655	3959243	3959929		0	O-methyltransferase	WP_085254420.1
G6N56_RS18660	3959940	3961655	ilvD		dihydroxy-acid dehydratase	WP_085254421.1

G6N56_RS18665	3961726	3962016		0	metal-sensitive transcriptional regulator	WP_085254422.1
G6N56_RS18670	3962138	3963370		0	MFS transporter	WP_085254423.1
G6N56_RS18675	3963477	3964469		0	L,D-transpeptidase	WP_163645139.1
G6N56_RS18685	3965138	3967369		0	molybdopterin-dependent oxidoreductase	WP_085258026.1
G6N56_RS18690	3967371	3969356		0	M13 family metallopeptidase	WP_085258027.1
G6N56_RS18695	3969399	3970037		0	hypothetical protein	WP_085258028.1
G6N56_RS18700	3970046	3970735		0	hypothetical protein	WP_085258062.1
G6N56_RS18705	3970741	3971250		0	transcriptional regulator	WP_085258029.1
G6N56_RS18710	3971247	3974285		0	MMPL family transporter	WP_085258030.1
G6N56_RS18725	3976497	3977654		0	AI-2E family transporter	WP_085258033.1
G6N56_RS18730	3977853	3980744		0	MMPL family transporter	WP_085258034.1
G6N56_RS18735	3980794	3981516		0	NYN domain-containing protein	WP_085258035.1
G6N56_RS18740	3981530	3982351	trmB		tRNA (guanosine(46)-N7)-methyltransferase TrmB	WP_142280812.1
G6N56_RS18745	3982524	3983615		0	hypothetical protein	WP_085258037.1
G6N56_RS18750	3983612	3985165		0	hypothetical protein	WP_085258063.1
G6N56_RS18755	3985340	3987169		0	phosphoenolpyruvate carboxykinase (GTP)	WP_085258038.1
G6N56_RS18760	3987231	3988760	fadD4		fatty-acid--CoA ligase FadD4	WP_085258039.1
G6N56_RS18765	3988820	3989701		0	enoyl-CoA hydratase/isomerase family protein	WP_232069359.1
G6N56_RS18775	3990169	3991323		0	acyl-CoA dehydrogenase family protein	WP_085258042.1
G6N56_RS18780	3991362	3992375		0	MaoC family dehydratase	WP_085258043.1
G6N56_RS18790	3993602	3994906		0	amidohydrolase family protein	WP_085258045.1
G6N56_RS18795	3994907	3995812		0	alpha/beta hydrolase	WP_085258046.1
G6N56_RS18800	3996026	3996793		0	alpha/beta hydrolase	WP_085258064.1
G6N56_RS18805	3996948	3999065		0	molybdopterin oxidoreductase family protein	WP_169717550.1
G6N56_RS18810	3999414	4001939	nirB		nitrite reductase large subunit NirB	WP_264020236.1
G6N56_RS18815	4001936	4002262		0	nitrite reductase (NAD(P)H) small subunit	WP_085258047.1
G6N56_RS18820	4002420	4003079		0	methyltransferase domain-containing protein	WP_085258048.1
G6N56_RS18825	4003079	4004392		0	molybdopterin-dependent oxidoreductase	WP_085258049.1
G6N56_RS18830	4004394	4004960		0	hypothetical protein	WP_085258050.1
G6N56_RS18835	4004980	4006161		0	alpha/beta hydrolase	WP_085258051.1
G6N56_RS18840	4006206	4007615		0	wax ester/triacylglycerol synthase family O-acyltransferase	WP_085258052.1

G6N56_RS18845	4007706	4008503	0	crotonase/enoyl-CoA hydratase family protein	WP_085258053.1
G6N56_RS18850	4008571	4010085	0	fatty acid--CoA ligase family protein	WP_085258054.1
G6N56_RS18855	4010082	4010378	0	hypothetical protein	WP_085258055.1
G6N56_RS18860	4010391	4010957	0	TetR/AcrR family transcriptional regulator	WP_085258056.1
G6N56_RS29515	4011047	4011178	0	hypothetical protein	WP_264020237.1
G6N56_RS18865	4011527	4012999	0	aldehyde dehydrogenase	WP_085258057.1
G6N56_RS18870	4013092	4013877	0	class I SAM-dependent methyltransferase	WP_085258066.1
G6N56_RS18875	4013922	4015076	0	glycosyltransferase family 4 protein	WP_085258058.1
G6N56_RS18885	4016780	4018060	0	DUF3068 domain-containing protein	WP_142280811.1
G6N56_RS18890	4018283	4019431	0	acyltransferase	WP_085258067.1
G6N56_RS29520	4019444	4020436	0	hypothetical protein	WP_163645141.1
G6N56_RS18900	4020437	4021498	0	hypothetical protein	WP_085257801.1
G6N56_RS18905	4021495	4022583	0	hypothetical protein	WP_085257802.1
G6N56_RS18915	4023037	4023909	0	DUF58 domain-containing protein	WP_085257804.1
G6N56_RS18920	4023916	4024920	0	MoxR family ATPase	WP_085257805.1
G6N56_RS18925	4024947	4026140	0	DUF4878 domain-containing protein	WP_085257806.1
G6N56_RS18930	4026310	4027287	0	phosphotriesterase-related protein	WP_085257807.1
G6N56_RS18935	4027405	4029102	0	acyl-CoA dehydrogenase family protein	WP_085257808.1
G6N56_RS18940	4029191	4029874	0	TetR/AcrR family transcriptional regulator	WP_085257809.1
G6N56_RS18945	4029871	4030812	0	R2-like ligand-binding oxidase	WP_085257810.1
G6N56_RS18950	4030834	4031988	0	alpha/beta fold hydrolase	WP_085257811.1
G6N56_RS18955	4032075	4032716	0	TetR/AcrR family transcriptional regulator	WP_085257812.1
G6N56_RS18960	4032729	4034147	0	NADP-dependent succinic semialdehyde dehydrogenase	WP_085257813.1
G6N56_RS18965	4034338	4035984	0	acetolactate synthase large subunit	WP_085257814.1
G6N56_RS18975	4037371	4037931	0	hypothetical protein	WP_085254679.1
G6N56_RS18980	4037928	4039367	0	lipase maturation factor family protein	WP_085254678.1
G6N56_RS18985	4039437	4043621	0	alpha-(1->3)-arabinofuranosyltransferase	WP_142280494.1
G6N56_RS18990	4043734	4043907	0	DUF2613 domain-containing protein	WP_003874046.1
G6N56_RS18995	4044016	4044267	0	DUF2613 domain-containing protein	WP_085254676.1
G6N56_RS19000	4044399	4045550	0	glycoside hydrolase family 3 N-terminal domain-containing pro	WP_085254675.1
G6N56_RS19005	4045648	4046298	0	TetR/AcrR family transcriptional regulator	WP_085254674.1

G6N56_RS19010	4046333	4047568		0	chloride channel protein	WP_085254673.1
G6N56_RS19015	4047575	4048420		0	MaoC/PaaZ C-terminal domain-containing protein	WP_085254672.1
G6N56_RS19020	4048437	4049813		0	3-oxoacyl-ACP reductase	WP_085254671.1
G6N56_RS19025	4049976	4051295		0	acetyl-CoA C-acetyltransferase	WP_085254670.1
G6N56_RS19030	4051647	4053482		0	acyl-CoA dehydrogenase	WP_085254669.1
G6N56_RS19035	4053673	4054704		0	2-oxoglutarate and iron-dependent oxygenase domain-containi	WP_085254668.1
G6N56_RS19040	4054711	4055199		0	flavin reductase family protein	WP_085254667.1
G6N56_RS28470	4055216	4055371		0	hypothetical protein	WP_169717518.1
G6N56_RS19045	4055441	4056187		0	succinate dehydrogenase/fumarate reductase iron-sulfur subu	WP_085254666.1
G6N56_RS19050	4056187	4058103		0	fumarate reductase/succinate dehydrogenase flavoprotein sub	WP_085254665.1
G6N56_RS19055	4058149	4058976		0	hypothetical protein	WP_085254664.1
G6N56_RS19060	4059065	4059361		0	hypothetical protein	WP_085254663.1
G6N56_RS19070	4059892	4060353		0	Hsp20/alpha crystallin family protein	WP_085254662.1
G6N56_RS19075	4060569	4063151	nirB		nitrite reductase large subunit NirB	WP_085254661.1
G6N56_RS19080	4063148	4063504	nirD		nitrite reductase small subunit NirD	WP_085254660.1
G6N56_RS19085	4063501	4063950		0	hypothetical protein	WP_085254659.1
G6N56_RS19095	4064773	4065930		0	uroporphyrinogen-III synthase	WP_085254657.1
G6N56_RS19100	4066008	4067405		0	nitrate/nitrite transporter	WP_142280493.1
G6N56_RS19105	4067821	4068366		0	GNAT family N-acetyltransferase	WP_169717517.1
G6N56_RS19110	4068377	4069294		0	5-oxoprolinase/urea amidolyase family protein	WP_142280496.1
G6N56_RS19115	4069324	4069986		0	allophanate hydrolase subunit 1	WP_085254654.1
G6N56_RS19120	4070088	4071092		0	ABC transporter substrate-binding protein	WP_085254653.1
G6N56_RS19125	4071149	4071733		0	TetR family transcriptional regulator	WP_085254686.1
G6N56_RS19130	4071899	4073290		0	nitrate/nitrite transporter	WP_085254652.1
G6N56_RS19135	4073401	4074639		0	DNA polymerase domain-containing protein	WP_085254651.1
G6N56_RS19140	4074699	4076378	fadD2		long-chain-fatty-acid--CoA ligase FadD2	WP_085254650.1
G6N56_RS19145	4076392	4076754		0	ATPase	WP_085254685.1
G6N56_RS28570	4077255	4077596		0	hypothetical protein	WP_180150365.1
G6N56_RS19155	4077671	4079881		0	acyl-CoA dehydrogenase	WP_085254649.1
G6N56_RS19160	4079932	4080915		0	2-hydroxyacid dehydrogenase	WP_085254648.1
G6N56_RS19165	4081018	4082151		0	alpha/beta hydrolase	WP_085254647.1

G6N56_RS19175	4082880	4083464		0	VOC family protein	WP_085254646.1
G6N56_RS19180	4083461	4084009		0	hypothetical protein	WP_085254645.1
G6N56_RS19190	4084526	4085227		0	TetR/AcrR family transcriptional regulator	WP_085254644.1
G6N56_RS19195	4085314	4086210		0	oxygenase MpaB family protein	WP_232069361.1
G6N56_RS19200	4086220	4086777		0	cation transporter	WP_232069362.1
G6N56_RS19205	4086866	4087177		0	metalloregulator ArsR/SmtB family transcription factor	WP_085254641.1
G6N56_RS19210	4087291	4089045		0	PPE family protein	WP_085254640.1
G6N56_RS19215	4089179	4090162		0	permease	WP_085254639.1
G6N56_RS19220	4090162	4090887		0	TIGR03943 family protein	WP_085254638.1
G6N56_RS19225	4090896	4091285		0	hypothetical protein	WP_085254637.1
G6N56_RS19230	4091326	4091583	rpsR		30S ribosomal protein S18	WP_085254636.1
G6N56_RS19235	4091583	4091888	rpsN		30S ribosomal protein S14	WP_085254635.1
G6N56_RS19240	4091891	4092055	rpmG		50S ribosomal protein L33	WP_085254634.1
G6N56_RS19245	4092055	4092291	rpmB		50S ribosomal protein L28	WP_085254633.1
G6N56_RS19250	4092399	4093598		0	GTP-binding protein	WP_085254632.1
G6N56_RS19255	4093598	4093852		0	type B 50S ribosomal protein L31	WP_085254631.1
G6N56_RS19260	4094108	4094527		0	transcriptional repressor	WP_085254682.1
G6N56_RS19265	4094563	4095426		0	metal ABC transporter permease	WP_180150581.1
G6N56_RS19275	4096267	4097214		0	zinc ABC transporter substrate-binding protein	WP_232069096.1
G6N56_RS19280	4097364	4098272		0	class I SAM-dependent methyltransferase	WP_085254628.1
G6N56_RS19285	4098407	4099912		0	PPE domain-containing protein	WP_085254627.1
G6N56_RS19290	4100101	4101954	eccA		type VII secretion AAA-ATPase EccA	WP_085254626.1
G6N56_RS19295	4101951	4103576	eccB		type VII secretion protein EccB	WP_085254625.1
G6N56_RS19300	4103573	4107550	eccCa		type VII secretion protein EccCa	WP_085254624.1
G6N56_RS19305	4107547	4107855		0	PE family protein	WP_085254623.1
G6N56_RS19310	4107858	4109378		0	PPE family protein	WP_085254622.1
G6N56_RS19315	4109436	4109729		0	WXG100 family type VII secretion target	WP_085254621.1
G6N56_RS19320	4109757	4110047		0	WXG100 family type VII secretion target	WP_085254620.1
G6N56_RS19325	4110069	4110953		0	ESX secretion-associated protein EspG	WP_085254619.1
G6N56_RS19330	4111036	4112445	eccD		type VII secretion integral membrane protein EccD	WP_142280495.1
G6N56_RS19335	4112442	4113842	mycP		type VII secretion-associated serine protease mycosin	WP_085254617.1

G6N56_RS19340	4113839	4114819	eccE		type VII secretion protein EccE	WP_085254616.1
G6N56_RS19345	4114816	4116024		0	alpha/beta fold hydrolase	WP_085254680.1
G6N56_RS19350	4116161	4116946		0	trans-aconitate 2-methyltransferase	WP_085254615.1
G6N56_RS19355	4116960	4118345		0	sulfatase	WP_232069363.1
G6N56_RS19360	4118360	4119007	bluB		5,6-dimethylbenzimidazole synthase	WP_085254613.1
G6N56_RS19370	4119343	4119825		0	hypothetical protein	WP_085254611.1
G6N56_RS19375	4119884	4120549		0	phosphatase PAP2 family protein	WP_085254610.1
G6N56_RS19380	4120576	4121232		0	L,D-transpeptidase	WP_085254609.1
G6N56_RS19390	4121731	4122366		0	TetR/AcrR family transcriptional regulator	WP_085254607.1
G6N56_RS19395	4122405	4123631		0	hypothetical protein	WP_085254606.1
G6N56_RS19400	4123797	4125707		0	Hsp70 family protein	WP_163645143.1
G6N56_RS19405	4125800	4127143		0	hypothetical protein	WP_163645144.1
G6N56_RS19410	4127209	4127610		0	hypothetical protein	WP_085256142.1
G6N56_RS19415	4127611	4128207		0	DUF3060 domain-containing protein	WP_085256141.1
G6N56_RS19420	4128289	4129314		0	NADP-dependent oxidoreductase	WP_180150367.1
G6N56_RS19425	4129429	4130025		0	hypothetical protein	WP_085256139.1
G6N56_RS19430	4130266	4131102		0	glycoside hydrolase family 16 protein	WP_085256138.1
G6N56_RS19435	4131106	4132626		0	glycosyltransferase family 39 protein	WP_232069097.1
G6N56_RS19440	4132662	4132967		0	hypothetical protein	WP_085256137.1
G6N56_RS19445	4132993	4133661		0	GAP family protein	WP_163645145.1
G6N56_RS19455	4134364	4135092		0	hypothetical protein	WP_085256135.1
G6N56_RS19460	4135118	4136608		0	bifunctional phosphatase PAP2/diacylglycerol kinase family pro	WP_085256134.1
G6N56_RS19465	4136618	4136959		0	DUF732 domain-containing protein	WP_085256133.1
G6N56_RS19470	4137156	4139006		0	alpha/beta hydrolase	WP_085256132.1
G6N56_RS19475	4139040	4139561		0	hypothetical protein	WP_180150369.1
G6N56_RS19480	4139639	4140118		0	M15 family metallopeptidase	WP_232069365.1
G6N56_RS19485	4140219	4140905		0	TetR family transcriptional regulator	WP_142280635.1
G6N56_RS19490	4141003	4141551		0	MmpS family transport accessory protein	WP_085256129.1
G6N56_RS19495	4141548	4144478		0	MMPL family transporter	WP_085256128.1
G6N56_RS19500	4144814	4145635		0	hypothetical protein	WP_232069366.1
G6N56_RS19505	4145780	4146022		0	hypothetical protein	WP_142280634.1

G6N56_RS19515	4146775	4147071		0	hypothetical protein	WP_232069098.1
G6N56_RS19520	4147073	4148461		0	tyrosine-type recombinase/integrase	WP_085256124.1
G6N56_RS19525	4148458	4149612		0	nucleotide-binding protein	WP_085256123.1
G6N56_RS19535	4150048	4150701		0	hypothetical protein	WP_142280633.1
G6N56_RS19540	4150738	4151310	dcd		dCTP deaminase	WP_085256122.1
G6N56_RS19545	4151441	4153336		0	hypothetical protein	WP_197746633.1
G6N56_RS19550	4153533	4154999		0	hypothetical protein	WP_163645146.1
G6N56_RS19555	4154996	4156120		0	hypothetical protein	WP_163645221.1
G6N56_RS19560	4156266	4157759		0	hypothetical protein	WP_232069099.1
G6N56_RS19565	4157841	4159166		0	UDP-glucose/GDP-mannose dehydrogenase family protein	WP_085258024.1
G6N56_RS19600	4167540	4168553		0	AraC family transcriptional regulator	WP_042792003.1
G6N56_RS19605	4168680	4169000		0	2Fe-2S iron-sulfur cluster-binding protein	WP_042792002.1
G6N56_RS19610	4169072	4170412		0	cytochrome P450	WP_051472937.1
G6N56_RS19615	4170409	4171611		0	FAD-dependent oxidoreductase	WP_042792000.1
G6N56_RS19625	4172517	4173017		0	PaaI family thioesterase	WP_042791998.1
G6N56_RS29525	4173990	4174439		0	ISAs1 family transposase	WP_142278381.1
G6N56_RS19680	4180614	4181321		0	TetR/AcrR family transcriptional regulator	WP_051472936.1
G6N56_RS19685	4181473	4181982		0	hotdog domain-containing protein	WP_042792068.1
G6N56_RS19695	4182320	4183282		0	IS481 family transposase	WP_042792067.1
G6N56_RS19720	4186062	4186952		0	alpha/beta hydrolase	WP_042791994.1
G6N56_RS19725	4186974	4187642		0	TetR/AcrR family transcriptional regulator	WP_042791993.1
G6N56_RS19730	4187742	4188626		0	metal-dependent hydrolase	WP_042792065.1
G6N56_RS19735	4188780	4189448		0	transglutaminase family protein	WP_042791992.1
G6N56_RS19740	4189445	4190542		0	BtrH N-terminal domain-containing protein	WP_082278412.1
G6N56_RS19745	4190565	4191671		0	NDMA-dependent alcohol dehydrogenase	WP_042791991.1
G6N56_RS19750	4191719	4193335		0	long-chain fatty acid--CoA ligase	WP_042909773.1
G6N56_RS19755	4193390	4193800		0	SCP2 sterol-binding domain-containing protein	WP_042791990.1
G6N56_RS19760	4194038	4194976		0	R2-like ligand-binding oxidase	WP_042791989.1
G6N56_RS19765	4195054	4195767		0	TetR/AcrR family transcriptional regulator	WP_051472933.1
G6N56_RS19770	4196085	4197227		0	acyl-CoA dehydrogenase family protein	WP_180150374.1
G6N56_RS19775	4197438	4198895		0	cytochrome P450	WP_042792062.1

G6N56_RS19780	4199031	4199621		0	TetR family transcriptional regulator	WP_042792061.1
G6N56_RS19785	4199917	4202934		0	MMPL family transporter	WP_232069100.1
G6N56_RS19805	4206093	4206416		0	transposase	WP_197746634.1
G6N56_RS19810	4206417	4207406		0	tyrosine-type recombinase/integrase	WP_042909774.1
G6N56_RS19815	4207403	4208182		0	tyrosine-type recombinase/integrase	WP_197746635.1
G6N56_RS19820	4208290	4209363		0	IS110 family transposase	WP_033711099.1
G6N56_RS19865	4218454	4219908		0	M28 family peptidase	WP_085257898.1
G6N56_RS19870	4219905	4221404		0	M28 family metallopeptidase	WP_085257897.1
G6N56_RS19875	4221453	4221878		0	DUF2752 domain-containing protein	WP_085257906.1
G6N56_RS19880	4221892	4222224		0	CD225/dispanin family protein	WP_085257896.1
G6N56_RS19885	4222513	4223028		0	serine/threonine protein kinase	WP_085257895.1
G6N56_RS19890	4223228	4223752		0	serine/threonine protein kinase	WP_085257894.1
G6N56_RS19895	4223756	4224514		0	SGNH/GDSL hydrolase family protein	WP_085257893.1
G6N56_RS19900	4224519	4225277		0	thiazole synthase	WP_085257892.1
G6N56_RS19910	4225464	4226474	thiO		glycine oxidase ThiO	WP_085257890.1
G6N56_RS19915	4226637	4227308	thiE		thiamine phosphate synthase	WP_085257889.1
G6N56_RS19925	4228068	4229387	glnX		protein kinase G-activating protein GlnX	WP_085257887.1
G6N56_RS19930	4229387	4230346		0	glutamate ABC transporter substrate-binding protein	WP_085257886.1
G6N56_RS19935	4230346	4232616		0	serine/threonine-protein kinase PknG	WP_085257885.1
G6N56_RS19940	4232804	4233664		0	hypothetical protein	WP_085257884.1
G6N56_RS19945	4233749	4235077		0	DUF1800 domain-containing protein	WP_085257883.1
G6N56_RS19950	4235090	4236277		0	DUF1501 domain-containing protein	WP_085257905.1
G6N56_RS19960	4237426	4239525	pta		phosphate acetyltransferase	WP_085257881.1
G6N56_RS19965	4239537	4240547	fgd		glucose-6-phosphate dehydrogenase (coenzyme-F420)	WP_085257880.1
G6N56_RS19970	4240703	4241455		0	MBL fold metallo-hydrolase	WP_085257879.1
G6N56_RS19985	4242402	4244660		0	MMPL family transporter	WP_085257876.1
G6N56_RS19990	4244733	4245638		0	class I SAM-dependent methyltransferase	WP_085257875.1
G6N56_RS19995	4245645	4246016		0	DUF3054 domain-containing protein	WP_085257874.1
G6N56_RS20000	4246084	4246830		0	SDR family oxidoreductase	WP_085257873.1
G6N56_RS20005	4246847	4248031		0	acyl-CoA dehydrogenase	WP_085257872.1
G6N56_RS20010	4248047	4249195		0	acyl-CoA dehydrogenase family protein	WP_085257871.1

G6N56_RS20015	4249195	4249869		0	TetR/AcrR family transcriptional regulator	WP_085257870.1
G6N56_RS20020	4249866	4250471		0	hypothetical protein	WP_085257869.1
G6N56_RS20025	4250573	4251952		0	NAD(P)/FAD-dependent oxidoreductase	WP_085257868.1
G6N56_RS20030	4251961	4253178		0	O-succinylhomoserine sulfhydrylase	WP_085257867.1
G6N56_RS20035	4253175	4253597		0	rhodanese-like domain-containing protein	WP_085257866.1
G6N56_RS20040	4253594	4254784	purT		formate-dependent phosphoribosylglycinamide formyltransfera	WP_232069367.1
G6N56_RS20045	4254804	4255442		0	PaaI family thioesterase	WP_085257865.1
G6N56_RS20050	4255439	4256737		0	adenylosuccinate synthase	WP_085257864.1
G6N56_RS20055	4256907	4257557		0	peptidase M50	WP_085257863.1
G6N56_RS20060	4257567	4258340		0	site-2 protease family protein	WP_085257862.1
G6N56_RS20065	4258382	4259278		0	cation diffusion facilitator family transporter	WP_085257861.1
G6N56_RS20070	4259275	4259688		0	DUF3151 domain-containing protein	WP_085257903.1
G6N56_RS20075	4259767	4260627		0	hypothetical protein	WP_085257860.1
G6N56_RS20080	4261015	4262049	fbaA		class II fructose-bisphosphate aldolase	WP_085257859.1
G6N56_RS20090	4262763	4264712		0	acetoacetate--CoA ligase	WP_163645149.1
G6N56_RS20095	4264709	4265839		0	glycoside hydrolase family 76 protein	WP_085257856.1
G6N56_RS20105	4266410	4267876		0	molybdopterin-dependent oxidoreductase	WP_197746636.1
G6N56_RS20110	4268519	4269721		0	VWA domain-containing protein	WP_085257853.1
G6N56_RS20115	4269728	4270381		0	SRPBCC family protein	WP_085257852.1
G6N56_RS20120	4270395	4271276		0	MoxR family ATPase	WP_085257902.1
G6N56_RS20125	4271273	4272151		0	XdhC family protein	WP_085257851.1
G6N56_RS20130	4272203	4274614		0	aerobic carbon-monoxide dehydrogenase large subunit	WP_085257850.1
G6N56_RS20135	4274611	4275087		0	(2Fe-2S)-binding protein	WP_085257849.1
G6N56_RS20140	4275090	4275974		0	xanthine dehydrogenase family protein subunit M	WP_085257848.1
G6N56_RS20145	4276039	4277199		0	XdhC family protein	WP_085257847.1
G6N56_RS20150	4277223	4278188		0	LysR family transcriptional regulator	WP_085257846.1
G6N56_RS20155	4278477	4279121		0	RNA methyltransferase	WP_085257901.1
G6N56_RS20160	4279118	4280026		0	hypothetical protein	WP_085257845.1
G6N56_RS20170	4280557	4281330		0	SDR family oxidoreductase	WP_085257844.1
G6N56_RS20175	4281382	4282251	ttfA		trehalose monomycolate transport factor TtfA	WP_085257843.1
G6N56_RS20180	4282353	4284899	clpB		ATP-dependent chaperone ClpB	WP_085257842.1

G6N56_RS20185	4285048	4286292		0	hypothetical protein	WP_085257841.1
G6N56_RS20190	4286289	4286789		0	hypothetical protein	WP_085257840.1
G6N56_RS20200	4288093	4294791		0	PE family protein	WP_163645224.1
G6N56_RS20205	4294991	4295218		0	PE family protein	WP_085254556.1
G6N56_RS20210	4295240	4296394		0	FAD-binding oxidoreductase	WP_085254538.1
G6N56_RS20215	4296398	4297288		0	aldose 1-epimerase	WP_085254539.1
G6N56_RS20220	4297293	4297688		0	helix-turn-helix domain-containing protein	WP_085254540.1
G6N56_RS20225	4297688	4298866	dnaJ		molecular chaperone DnaJ	WP_085254541.1
G6N56_RS20230	4298920	4299561	grpE		nucleotide exchange factor GrpE	WP_085254542.1
G6N56_RS20235	4299558	4301417	dnaK		molecular chaperone DnaK	WP_085254543.1
G6N56_RS20240	4301598	4302512		0	LysR family transcriptional regulator	WP_085254557.1
G6N56_RS20245	4302682	4304130		0	aldehyde dehydrogenase family protein	WP_232069102.1
G6N56_RS20250	4304141	4305091		0	amidohydrolase family protein	WP_085254545.1
G6N56_RS20255	4305171	4306622		0	amino acid permease	WP_085254546.1
G6N56_RS20260	4306619	4307212		0	nucleotidyltransferase family protein	WP_085254547.1
G6N56_RS20265	4307262	4309592		0	xanthine dehydrogenase family protein molybdopterin-binding s	WP_085254548.1
G6N56_RS20270	4309589	4310479		0	xanthine dehydrogenase family protein subunit M	WP_085254549.1
G6N56_RS20280	4311095	4311661		0	hypothetical protein	WP_085254558.1
G6N56_RS28480	4311832	4312359		0	hypothetical protein	WP_169717516.1
G6N56_RS20290	4312430	4314106		0	Hsp70 family protein	WP_085254551.1
G6N56_RS20295	4314222	4317110		0	(Fe-S)-binding protein	WP_085254552.1
G6N56_RS20300	4317141	4318442		0	pyridoxal phosphate-dependent aminotransferase	WP_085254553.1
G6N56_RS20305	4318463	4319737		0	YibE/F family protein	WP_085254560.1
G6N56_RS20315	4320588	4320941		0	nuclear transport factor 2 family protein	WP_085254561.1
G6N56_RS20320	4320972	4321727		0	maleylpyruvate isomerase family mycothiol-dependent enzyme	WP_085254555.1
G6N56_RS20325	4321831	4323045		0	DUF222 domain-containing protein	WP_163645151.1
G6N56_RS20390	4335013	4335690		0	hypothetical protein	WP_085240914.1
G6N56_RS20400	4337506	4338102		0	TetR/AcrR family transcriptional regulator	WP_096288364.1
G6N56_RS20405	4338584	4338760		0	hypothetical protein	WP_158089970.1
G6N56_RS20410	4338909	4339208		0	hypothetical protein	WP_042792006.1
G6N56_RS20415	4339256	4339897		0	TetR/AcrR family transcriptional regulator	WP_038534967.1

G6N56_RS20420	4340592	4341845		0	MCE family protein	WP_269473822.1
G6N56_RS20425	4341901	4342053		0	hypothetical protein	WP_158089969.1
G6N56_RS20430	4342226	4342888		0	TetR/AcrR family transcriptional regulator	WP_042792008.1
G6N56_RS20470	4350883	4352340		0	peptidase	WP_085258783.1
G6N56_RS20480	4352969	4353796	thiD		bifunctional hydroxymethylpyrimidine kinase/phosphomethylpy	WP_085258782.1
G6N56_RS20485	4353793	4355424	thiC		phosphomethylpyrimidine synthase ThiC	WP_085258803.1
G6N56_RS20490	4355595	4356281		0	PAS and ANTAR domain-containing protein	WP_232069104.1
G6N56_RS20495	4356412	4356768		0	STAS domain-containing protein	WP_085258780.1
G6N56_RS20500	4356765	4360628		0	SpoIIE family protein phosphatase	WP_085258779.1
G6N56_RS20505	4360625	4360900		0	hypothetical protein	WP_142280884.1
G6N56_RS29535	4360964	4361257		0	Rv1535 domain-containing protein	WP_264020610.1
G6N56_RS20515	4365433	4365879		0	hypothetical protein	WP_085258776.1
G6N56_RS20520	4366001	4366828		0	exodeoxyribonuclease III	WP_085258775.1
G6N56_RS20525	4366831	4367670		0	GNAT family N-acetyltransferase	WP_085258774.1
G6N56_RS20530	4367670	4368263		0	peptide deformylase	WP_085258773.1
G6N56_RS20535	4368412	4368720		0	DUF3263 domain-containing protein	WP_085258772.1
G6N56_RS20540	4368740	4369213		0	LytR C-terminal domain-containing protein	WP_085258771.1
G6N56_RS20550	4369891	4371021		0	glutamate--cysteine ligase	WP_142280877.1
G6N56_RS20555	4371021	4371659		0	LON peptidase substrate-binding domain-containing protein	WP_085258768.1
G6N56_RS20560	4371656	4372486		0	enoyl-CoA hydratase-related protein	WP_085258767.1
G6N56_RS20570	4373058	4373987		0	SDR family NAD(P)-dependent oxidoreductase	WP_085258765.1
G6N56_RS20575	4373984	4375441		0	acyl-CoA reductase	WP_085258764.1
G6N56_RS20580	4375438	4376868		0	hypothetical protein	WP_085258763.1
G6N56_RS20585	4377003	4379186		0	AAA family ATPase	WP_085258762.1
G6N56_RS20590	4379183	4380046	pssA		CDP-diacylglycerol--serine O-phosphatidyltransferase	WP_085258761.1
G6N56_RS20595	4380043	4380765		0	phosphatidylserine decarboxylase	WP_085258760.1
G6N56_RS20605	4381985	4383190		0	molybdopterin molybdotransferase MoeA	WP_085258758.1
G6N56_RS20610	4383209	4384147		0	SDR family NAD(P)-dependent oxidoreductase	WP_085258757.1
G6N56_RS20620	4384674	4386110		0	NAD(P)/FAD-dependent oxidoreductase	WP_085258755.1
G6N56_RS20625	4386320	4386826		0	cupin domain-containing protein	WP_085258754.1
G6N56_RS20630	4386857	4387558		0	M24 family metallopeptidase	WP_085258753.1

G6N56_RS20635	4387569	4388483		0	SDR family NAD(P)-dependent oxidoreductase	WP_085258752.1
G6N56_RS20640	4388581	4389147		0	TetR/AcrR family transcriptional regulator	WP_085258751.1
G6N56_RS20645	4389351	4390976	groL		chaperonin GroEL	WP_077099793.1
G6N56_RS29230	4391471	4391686		0	DUF559 domain-containing protein	WP_232069105.1
G6N56_RS20655	4391732	4392136		0	hypothetical protein	WP_085258750.1
G6N56_RS20660	4392155	4393573		0	PPE family protein	WP_085258749.1
G6N56_RS20665	4393612	4395354		0	amidohydrolase family protein	WP_085258748.1
G6N56_RS20670	4395420	4395932		0	mycothiol transferase	WP_085258747.1
G6N56_RS20675	4395942	4396805		0	hypothetical protein	WP_085258746.1
G6N56_RS20680	4396802	4398475		0	cytochrome c biogenesis protein DipZ	WP_085258745.1
G6N56_RS20685	4398486	4401356		0	RND family transporter	WP_180150376.1
G6N56_RS20690	4401353	4401799		0	MmpS family protein	WP_085258744.1
G6N56_RS20695	4402280	4402741		0	DUF5078 domain-containing protein	WP_085258743.1
G6N56_RS20700	4402807	4403715		0	crotonase/enoyl-CoA hydratase family protein	WP_085258742.1
G6N56_RS20705	4403727	4404038		0	hypothetical protein	WP_085258741.1
G6N56_RS20710	4404114	4406141		0	prolyl oligopeptidase family serine peptidase	WP_085258740.1
G6N56_RS20715	4406208	4407731		0	aldehyde dehydrogenase family protein	WP_085258739.1
G6N56_RS20720	4407815	4408282		0	DUF779 domain-containing protein	WP_232069369.1
G6N56_RS20730	4408571	4409131		0	hypothetical protein	WP_085258736.1
G6N56_RS20735	4409170	4410573	lpdA		dihydrolipoyl dehydrogenase	WP_085258735.1
G6N56_RS20740	4410576	4410851		0	hypothetical protein	WP_085258734.1
G6N56_RS20745	4410848	4411426		0	carboxymuconolactone decarboxylase family protein	WP_085258733.1
G6N56_RS20750	4411423	4412844	ramB		acetate metabolism transcriptional regulator RamB	WP_085258732.1
G6N56_RS20755	4413005	4413799		0	acyl-[acyl-carrier-protein] thioesterase	WP_085258731.1
G6N56_RS20760	4414069	4415355	aceA		isocitrate lyase	WP_085258730.1
G6N56_RS20765	4415574	4416437		0	3-hydroxybutyryl-CoA dehydrogenase	WP_085258729.1
G6N56_RS20770	4416541	4417419		0	cyclopropane mycolic acid synthase family methyltransferase	WP_085258728.1
G6N56_RS20775	4417438	4418301	pcaA		cyclopropane mycolic acid synthase PcaA	WP_085258727.1
G6N56_RS20780	4418484	4419203		0	TetR/AcrR family transcriptional regulator	WP_085258726.1
G6N56_RS20785	4419342	4420667		0	DUF445 domain-containing protein	WP_085258725.1
G6N56_RS20790	4420752	4421180		0	helix-turn-helix transcriptional regulator	WP_085258801.1

G6N56_RS20795	4421307	4421900	hbhA	heparin-binding hemagglutinin HbhA	WP_085258724.1
G6N56_RS20800	4421992	4422279	0	DUF2516 family protein	WP_085258723.1
G6N56_RS20805	4422284	4422727	0	DUF2599 domain-containing protein	WP_085258722.1
G6N56_RS20810	4422727	4423416	deoC	deoxyribose-phosphate aldolase	WP_085258721.1
G6N56_RS20815	4423438	4424466	0	DUF2993 domain-containing protein	WP_085258720.1
G6N56_RS20820	4424463	4425293	0	carbon-nitrogen hydrolase family protein	WP_085258719.1
G6N56_RS20825	4425329	4425853	0	DUF2505 domain-containing protein	WP_085258718.1
G6N56_RS20830	4425880	4426986	0	UDP-N-acetylmuramate dehydrogenase	WP_085258717.1
G6N56_RS20835	4427094	4428416	0	Ig-like domain-containing protein	WP_085258716.1
G6N56_RS20845	4429327	4430634	0	ROK family protein	WP_085258714.1
G6N56_RS20850	4430693	4432033	mshA	D-inositol-3-phosphate glycosyltransferase	WP_085258713.1
G6N56_RS20855	4432030	4432602	0	YbjN domain-containing protein	WP_142280874.1
G6N56_RS20860	4432613	4433362	0	phosphoglyceromutase	WP_085258712.1
G6N56_RS20865	4433514	4434734	0	ATP-binding protein	WP_142280873.1
G6N56_RS20870	4434784	4435467	regX	two-component sensory transduction protein RegX	WP_085258800.1
G6N56_RS20875	4435560	4435778	0	ferric uptake regulation protein	WP_085258710.1
G6N56_RS20880	4436389	4436811	0	MmpS family transport accessory protein	WP_180150379.1
G6N56_RS20885	4436808	4439744	0	RND family transporter	WP_085258708.1
G6N56_RS20890	4440014	4441873	0	GMC family oxidoreductase	WP_085258707.1
G6N56_RS20895	4441870	4442199	0	hypothetical protein	WP_085258706.1
G6N56_RS20900	4442196	4443146	0	hypothetical protein	WP_085258705.1
G6N56_RS20905	4443190	4443912	0	GntR family transcriptional regulator	WP_085258704.1
G6N56_RS20910	4443925	4444710	0	hypothetical protein	WP_085258703.1
G6N56_RS20915	4444817	4445830	0	Ppx/GppA family phosphatase	WP_085258702.1
G6N56_RS20920	4445827	4446813	0	hypothetical protein	WP_085258701.1
G6N56_RS20925	4446831	4447670	0	sugar phosphate isomerase/epimerase	WP_085258700.1
G6N56_RS20930	4447686	4448525	0	thioesterase family protein	WP_085258699.1
G6N56_RS20935	4448553	4449428	proC	pyrroline-5-carboxylate reductase	WP_180150381.1
G6N56_RS20940	4449569	4449826	0	cell division/environmental response transcriptional regulator	WP_085258697.1
G6N56_RS20945	4449985	4450086	0	AURKAIP1/COX24 domain-containing protein	WP_003402602.1
G6N56_RS20950	4450156	4451286	0	SDR family oxidoreductase	WP_085258696.1

G6N56_RS20960	4452525	4453433	cmaA2		cyclopropane mycolic acid synthase CmaA2	WP_085258695.1
G6N56_RS20965	4453444	4453956		0	MaoC family dehydratase N-terminal domain-containing protei	WP_085258694.1
G6N56_RS20970	4454000	4454905		0	HAD-IB family hydrolase	WP_085258693.1
G6N56_RS20975	4455000	4455590		0	hypothetical protein	WP_085258692.1
G6N56_RS20980	4455679	4455819		0	hypothetical protein	WP_158090775.1
G6N56_RS20990	4456158	4456469		0	WXG100 family type VII secretion target	WP_085258690.1
G6N56_RS20995	4456512	4456790		0	glutaredoxin family protein	WP_085258689.1
G6N56_RS21000	4456888	4458276		0	glutamyl-tRNA reductase	WP_085258688.1
G6N56_RS21005	4458290	4459240	hemC		hydroxymethylbilane synthase	WP_085258798.1
G6N56_RS21010	4459260	4460945		0	uroporphyrinogen-III synthase	WP_085258687.1
G6N56_RS21015	4461055	4462044	hemB		porphobilinogen synthase	WP_085258686.1
G6N56_RS21020	4462059	4462619		0	DUF3093 domain-containing protein	WP_180150383.1
G6N56_RS21030	4462860	4463270		0	STAS domain-containing protein	WP_142280881.1
G6N56_RS21035	4463554	4464792		0	acyltransferase	WP_085258684.1
G6N56_RS21040	4464883	4465578		0	SGNH/GDSL hydrolase family protein	WP_085258683.1
G6N56_RS21045	4465808	4467970		0	acyltransferase family protein	WP_085258682.1
G6N56_RS21050	4467978	4468835		0	alpha/beta hydrolase-fold protein	WP_180150585.1
G6N56_RS21055	4469031	4470176		0	LLM class flavin-dependent oxidoreductase	WP_085258681.1
G6N56_RS21060	4470194	4471528		0	MFS transporter	WP_085258680.1
G6N56_RS21065	4471596	4473014		0	amino acid permease	WP_232069106.1
G6N56_RS21070	4473024	4473794		0	hypothetical protein	WP_085258679.1
G6N56_RS21075	4473898	4474290		0	nitroreductase family deazaflavin-dependent oxidoreductase	WP_085258678.1
G6N56_RS21080	4474516	4475844	hemL		glutamate-1-semialdehyde 2,1-aminomutase	WP_085258677.1
G6N56_RS21085	4475844	4476452		0	histidine phosphatase family protein	WP_085258676.1
G6N56_RS21090	4476455	4477054		0	TlpA disulfide reductase family protein	WP_180150386.1
G6N56_RS21095	4477051	4477830		0	cytochrome c biogenesis CcdA family protein	WP_085258675.1
G6N56_RS21100	4477887	4479470		0	cytochrome c biogenesis protein ResB	WP_085258793.1
G6N56_RS21105	4479467	4480444	ccsB		c-type cytochrome biogenesis protein CcsB	WP_085258674.1
G6N56_RS21110	4480537	4481763		0	MinD/ParA family protein	WP_232069371.1
G6N56_RS21115	4481776	4481934		0	hypothetical protein	WP_142280870.1
G6N56_RS21120	4481983	4482366		0	DUF4229 domain-containing protein	WP_085258673.1

G6N56_RS21125	4482487	4483359		0	1,4-dihydroxy-2-naphthoate polyprenyltransferase	WP_085258790.1
G6N56_RS21130	4483433	4484218		0	S-methyl-5'-thioadenosine phosphorylase	WP_085258789.1
G6N56_RS21135	4484215	4485243		0	NAD-dependent epimerase/dehydratase family protein	WP_085258672.1
G6N56_RS21140	4485262	4486896		0	hypothetical protein	WP_232069373.1
G6N56_RS21145	4487218	4489065		0	hypothetical protein	WP_085258670.1
G6N56_RS21150	4489084	4489743		0	glycosyltransferase family 2 protein	WP_085258669.1
G6N56_RS21155	4489740	4490411		0	DUF2064 domain-containing protein	WP_085258668.1
G6N56_RS21160	4490510	4491850		0	hypothetical protein	WP_085258788.1
G6N56_RS21165	4491886	4492962	menE		o-succinylbenzoate--CoA ligase	WP_232069374.1
G6N56_RS21170	4493031	4493333		0	DUF3349 domain-containing protein	WP_085258667.1
G6N56_RS21175	4493387	4493686		0	hypothetical protein	WP_085258666.1
G6N56_RS21180	4493683	4494933		0	inorganic phosphate transporter	WP_085258665.1
G6N56_RS21185	4495020	4495427		0	VOC family protein	WP_085258664.1
G6N56_RS21190	4495449	4496345		0	SDR family oxidoreductase	WP_085258663.1
G6N56_RS21195	4496388	4497290		0	1,4-dihydroxy-2-naphthoyl-CoA synthase	WP_085258662.1
G6N56_RS21200	4497301	4497756		0	nitroreductase family deazaflavin-dependent oxidoreductase	WP_085258661.1
G6N56_RS21205	4497768	4498688		0	aldo/keto reductase	WP_085258660.1
G6N56_RS21210	4498773	4499315		0	TetR/AcrR family transcriptional regulator	WP_085258659.1
G6N56_RS21215	4499396	4500997	fadD8		fatty-acid--CoA ligase FadD8	WP_085258658.1
G6N56_RS21220	4501075	4502679		0	amidohydrolase	WP_085258657.1
G6N56_RS21230	4503644	4504432		0	alpha/beta hydrolase	WP_085258655.1
G6N56_RS21235	4504502	4506139	menD		2-succinyl-5-enolpyruvyl-6-hydroxy-3- cyclohexene-1-carboxyli	WP_085258654.1
G6N56_RS21240	4506153	4506680		0	DUF3592 domain-containing protein	WP_085258653.1
G6N56_RS21245	4506759	4507916		0	glycosyltransferase family 1 protein	WP_085258652.1
G6N56_RS21250	4508032	4508856		0	SDR family oxidoreductase	WP_085258786.1
G6N56_RS21255	4508912	4509601		0	demethylmenaquinone methyltransferase	WP_085258651.1
G6N56_RS21260	4509632	4509982		0	DUF732 domain-containing protein	WP_085258650.1
G6N56_RS21265	4510018	4510257		0	hypothetical protein	WP_142280869.1
G6N56_RS21270	4510334	4511548	menJ		menaquinone reductase	WP_085258785.1
G6N56_RS21275	4511597	4512613		0	polyprenyl synthetase family protein	WP_085258649.1
G6N56_RS21280	4512705	4513565	htpX		zinc metalloprotease HtpX	WP_085258648.1

G6N56_RS21285	4513633	4513833		0	toxin-antitoxin system HicB family antitoxin	WP_232069107.1
G6N56_RS21290	4513842	4515365		0	SulP family inorganic anion transporter	WP_085258647.1
G6N56_RS21295	4515589	4516614		0	NAD(P)H-dependent glycerol-3-phosphate dehydrogenase	WP_085258646.1
G6N56_RS21300	4516774	4518192		0	trypsin-like peptidase domain-containing protein	WP_232069108.1
G6N56_RS21305	4518196	4519677		0	NAD(P)/FAD-dependent oxidoreductase	WP_163645154.1
G6N56_RS21310	4519788	4520279		0	YajQ family cyclic di-GMP-binding protein	WP_085254450.1
G6N56_RS21320	4520524	4521279		0	DUF2182 domain-containing protein	WP_085254451.1
G6N56_RS21325	4521399	4521755	csoR		copper-sensing transcriptional repressor CsoR	WP_085254452.1
G6N56_RS21330	4521794	4522084		0	DUF1490 family protein	WP_085254509.1
G6N56_RS21335	4522147	4524534		0	copper-translocating P-type ATPase	WP_142280476.1
G6N56_RS21340	4524735	4525097		0	hypothetical protein	WP_085254454.1
G6N56_RS21345	4525119	4526054		0	M56 family metallopeptidase	WP_085254455.1
G6N56_RS21350	4526051	4526434		0	BlaI/MecI/CopY family transcriptional regulator	WP_085254456.1
G6N56_RS21355	4526720	4527958		0	divalent metal cation transporter	WP_232069375.1
G6N56_RS21360	4527955	4528980		0	cation diffusion facilitator family transporter	WP_085254458.1
G6N56_RS21365	4529252	4530283		0	fused (3R)-hydroxyacyl-ACP dehydratase subunits HadA/HadB	WP_085254459.1
G6N56_RS21370	4530290	4531645		0	hypothetical protein	WP_085254460.1
G6N56_RS21375	4531868	4532809		0	DUF1254 domain-containing protein	WP_085254461.1
G6N56_RS21380	4533027	4533917		0	NYN domain-containing protein	WP_085254510.1
G6N56_RS21385	4534007	4534879		0	alpha/beta fold hydrolase	WP_085254462.1
G6N56_RS21390	4535144	4536778		0	fatty acyl-AMP ligase	WP_085254463.1
G6N56_RS21395	4537046	4537417		0	DUF732 domain-containing protein	WP_085254464.1
G6N56_RS21400	4537461	4538450		0	aldo/keto reductase	WP_085254465.1
G6N56_RS21405	4538447	4539217		0	cupin domain-containing protein	WP_085254466.1
G6N56_RS21410	4539961	4540812		0	alpha/beta hydrolase family protein	WP_232069376.1
G6N56_RS21415	4541804	4542571		0	ABC transporter permease	WP_142280471.1
G6N56_RS21420	4542607	4543425		0	ABC transporter permease	WP_232069377.1
G6N56_RS21425	4543431	4544885		0	MCE family protein	WP_085254512.1
G6N56_RS21430	4544926	4545954		0	MlaD family protein	WP_085254469.1
G6N56_RS21435	4545951	4547324		0	MCE family protein	WP_085254470.1
G6N56_RS21440	4547485	4548831		0	MCE family protein	WP_264020139.1

G6N56_RS21445	4548846	4549964	0	virulence factor Mce family protein	WP_085254471.1
G6N56_RS21450	4549967	4551424	0	MlaD family protein	WP_085254472.1
G6N56_RS21455	4551531	4552214	0	cyclase family protein	WP_232069110.1
G6N56_RS21460	4552608	4553756	0	acyl-CoA dehydrogenase family protein	WP_085254473.1
G6N56_RS21465	4553807	4554835	0	acyl-CoA dehydrogenase family protein	WP_264020141.1
G6N56_RS21475	4555604	4556554	0	PDR/VanB family oxidoreductase	WP_085254476.1
G6N56_RS21480	4556564	4556749	0	ferredoxin	WP_085254515.1
G6N56_RS21485	4556767	4558005	0	cytochrome P450	WP_158090683.1
G6N56_RS21490	4558174	4558929	0	SDR family NAD(P)-dependent oxidoreductase	WP_232069378.1
G6N56_RS21495	4559098	4560963	0	NAD(P)/FAD-dependent oxidoreductase	WP_232069111.1
G6N56_RS21500	4561305	4562351	0	hypothetical protein	WP_085254479.1
G6N56_RS21505	4562348	4563511	0	thiolase family protein	WP_085254480.1
G6N56_RS21510	4563532	4563969	0	zinc ribbon domain-containing protein	WP_142280472.1
G6N56_RS21515	4564033	4564722	0	TetR/AcrR family transcriptional regulator	WP_085254482.1
G6N56_RS21520	4565066	4566307	0	2-oxo acid dehydrogenase subunit E2	WP_232069112.1
G6N56_RS21525	4566318	4566482	0	hypothetical protein	WP_158090684.1
G6N56_RS21530	4566959	4567558	0	VOC family protein	WP_085254484.1
G6N56_RS21535	4567560	4568429	0	fumarylacetoacetate hydrolase family protein	WP_085254485.1
G6N56_RS21540	4568431	4570218	0	FAD-dependent monooxygenase	WP_085254486.1
G6N56_RS21545	4570254	4571591	0	hypothetical protein	WP_142280473.1
G6N56_RS21550	4571592	4572806	0	thiolase family protein	WP_232069114.1
G6N56_RS21555	4572822	4574426	0	AMP-binding protein	WP_085254488.1
G6N56_RS21560	4574586	4574990	0	nuclear transport factor 2 family protein	WP_085254489.1
G6N56_RS21565	4575011	4575358	0	DoxX family protein	WP_085254490.1
G6N56_RS21570	4575378	4577726	0	pyruvate dehydrogenase	WP_085254491.1
G6N56_RS21575	4577734	4578501	0	hypothetical protein	WP_232069115.1
G6N56_RS21580	4578498	4579124	0	hypothetical protein	WP_232069116.1
G6N56_RS21595	4580357	4581364	0	amidohydrolase family protein	WP_158090685.1
G6N56_RS21600	4581895	4583433	0	AMP-binding protein	WP_085254494.1
G6N56_RS21605	4583509	4584171	0	DUF2889 domain-containing protein	WP_085254495.1
G6N56_RS21610	4584252	4585040	0	SDR family NAD(P)-dependent oxidoreductase	WP_085254496.1

G6N56_RS21615	4585281	4585943		0	TetR/AcrR family transcriptional regulator	WP_085254497.1
G6N56_RS21620	4585940	4586278		0	non-heme iron oxygenase ferredoxin subunit	WP_232069117.1
G6N56_RS21625	4586370	4587194		0	SDR family oxidoreductase	WP_232069118.1
G6N56_RS21635	4587657	4589036		0	Rieske 2Fe-2S domain-containing protein	WP_232069119.1
G6N56_RS21640	4589033	4589977		0	VOC family protein	WP_085254501.1
G6N56_RS21645	4590352	4591119		0	IclR family transcriptional regulator	WP_158090686.1
G6N56_RS21650	4591214	4592401		0	FAD-dependent oxidoreductase	WP_085254521.1
G6N56_RS21660	4592726	4593697		0	alpha/beta hydrolase	WP_085254504.1
G6N56_RS21665	4593976	4594752		0	DUF4386 family protein	WP_085254505.1
G6N56_RS21675	4595393	4595632		0	cytochrome C oxidase subunit IV family protein	WP_158090687.1
G6N56_RS21680	4595700	4597316		0	NAD(P)/FAD-dependent oxidoreductase	WP_232069379.1
G6N56_RS21690	4598925	4600109		0	cytochrome P450	WP_163645156.1
G6N56_RS21695	4600408	4600602		0	hypothetical protein	WP_142280817.1
G6N56_RS21700	4600925	4601719		0	fumarylacetoacetate hydrolase family protein	WP_085258157.1
G6N56_RS21705	4601731	4602669		0	acetaldehyde dehydrogenase (acetylating)	WP_085258156.1
G6N56_RS21710	4602669	4603703	dmpG		4-hydroxy-2-oxovalerate aldolase	WP_085258155.1
G6N56_RS21715	4603703	4605334		0	aldehyde dehydrogenase family protein	WP_085258154.1
G6N56_RS21720	4605434	4606462		0	LLM class flavin-dependent oxidoreductase	WP_085258153.1
G6N56_RS21725	4606821	4607258		0	MmpS family transport accessory protein	WP_197746677.1
G6N56_RS21730	4607255	4610122		0	MMPL family transporter	WP_085258151.1
G6N56_RS21735	4610185	4610811		0	TetR/AcrR family transcriptional regulator	WP_085258150.1
G6N56_RS21740	4610947	4613154		0	FUSC family protein	WP_085258167.1
G6N56_RS21745	4613219	4614346	galT		galactose-1-phosphate uridylyltransferase	WP_085258149.1
G6N56_RS21750	4614343	4615434		0	galactokinase	WP_085258148.1
G6N56_RS21755	4615483	4615821		0	hypothetical protein	WP_085258147.1
G6N56_RS21760	4615818	4616489		0	PAS and ANTAR domain-containing protein	WP_085258146.1
G6N56_RS21765	4616502	4616984		0	hypothetical protein	WP_085258166.1
G6N56_RS21775	4617402	4620050		0	GH92 family glycosyl hydrolase	WP_085258145.1
G6N56_RS21780	4620059	4622434		0	lysylphosphatidylglycerol synthase transmembrane domain-co	WP_085258144.1
G6N56_RS21785	4622582	4623532		0	class I SAM-dependent methyltransferase	WP_085258165.1
G6N56_RS21790	4623540	4624487		0	class I SAM-dependent methyltransferase	WP_085258143.1

G6N56_RS21795	4624491	4625252		0	TVP38/TMEM64 family protein	WP_085258142.1
G6N56_RS21800	4625275	4626072		0	SDR family NAD(P)-dependent oxidoreductase	WP_085258141.1
G6N56_RS21805	4626084	4627844	recD		exodeoxyribonuclease V subunit alpha	WP_085258140.1
G6N56_RS21810	4627841	4631134	recB		exodeoxyribonuclease V subunit beta	WP_085258139.1
G6N56_RS21815	4631134	4634439	recC		exodeoxyribonuclease V subunit gamma	WP_085258138.1
G6N56_RS21820	4634535	4635050		0	nitroreductase family protein	WP_085258137.1
G6N56_RS21825	4635119	4635961		0	lysophospholipid acyltransferase family protein	WP_085258136.1
G6N56_RS21830	4635961	4636416		0	SRPBCC family protein	WP_085258135.1
G6N56_RS21835	4636511	4637206		0	crotonase/enoyl-CoA hydratase family protein	WP_085258134.1
G6N56_RS21840	4637223	4638056		0	hypothetical protein	WP_085258133.1
G6N56_RS21845	4638153	4638866		0	MBL fold metallo-hydrolase	WP_085258132.1
G6N56_RS21850	4638963	4639172		0	type II toxin-antitoxin system VapB family antitoxin	WP_085258131.1
G6N56_RS21865	4639530	4639697	rpmG		50S ribosomal protein L33	WP_003898538.1
G6N56_RS21870	4639744	4640223	hadA		(3R)-hydroxyacyl-ACP dehydratase subunit HadA	WP_085258130.1
G6N56_RS21880	4640642	4641142	hadC		(3R)-hydroxyacyl-ACP dehydratase subunit HadC	WP_085258128.1
G6N56_RS21890	4641479	4641955	secE		preprotein translocase subunit SecE	WP_085258127.1
G6N56_RS21895	4641987	4642712	nusG		transcription termination/antitermination protein NusG	WP_085258126.1
G6N56_RS21900	4642772	4643200	rplK		50S ribosomal protein L11	WP_085258125.1
G6N56_RS21905	4643269	4643976	rplA		50S ribosomal protein L1	WP_085258124.1
G6N56_RS21910	4644065	4645282		0	sigma-70 family RNA polymerase sigma factor	WP_085258123.1
G6N56_RS21915	4645291	4645989		0	YciI family protein	WP_085258122.1
G6N56_RS21920	4646097	4646993	mmaA4		hydroxymycolate synthase MmaA4	WP_085258164.1
G6N56_RS21925	4647079	4647960	mmaA3		methoxy mycolic acid synthase MmaA3	WP_085258121.1
G6N56_RS21930	4648103	4648966	mmaA2		cyclopropane mycolic acid synthase MmaA2	WP_085258120.1
G6N56_RS21935	4648978	4649838	mmaA1		mycolic acid methyltransferase MmaA1	WP_085258119.1
G6N56_RS21940	4649927	4650844		0	alpha/beta hydrolase	WP_085258118.1
G6N56_RS21945	4650844	4652190		0	AarF/ABC1/UbiB kinase family protein	WP_180150390.1
G6N56_RS21950	4652269	4652841		0	DinB family protein	WP_085258117.1
G6N56_RS21955	4652912	4657096		0	NEW3 domain-containing protein	WP_085258116.1
G6N56_RS21960	4657093	4657710		0	malonyl CoA-ACP transacylase	WP_085258115.1
G6N56_RS21965	4657716	4658645		0	ROK family protein	WP_085258114.1

G6N56_RS21970	4658928	4659503	rplJ		50S ribosomal protein L10	WP_085258112.1
G6N56_RS21975	4659534	4659926	rplL		50S ribosomal protein L7/L12	WP_085258111.1
G6N56_RS21980	4659978	4660676		0	TetR/AcrR family transcriptional regulator	WP_085258162.1
G6N56_RS21985	4660748	4662244		0	carotenoid oxygenase family protein	WP_085258110.1
G6N56_RS21990	4662414	4663424		0	ABC transporter ATP-binding protein	WP_085258109.1
G6N56_RS21995	4664000	4667434		0	DNA-directed RNA polymerase subunit beta	WP_085258108.1
G6N56_RS22000	4667495	4671445		0	DNA-directed RNA polymerase subunit beta'	WP_085258107.1
G6N56_RS29235	4671627	4672046		0	hypothetical protein	WP_085258106.1
G6N56_RS29240	4672194	4672442		0	hypothetical protein	WP_085258105.1
G6N56_RS22010	4672513	4673268		0	deoxyribonuclease IV	WP_085258161.1
G6N56_RS22015	4673275	4674108		0	PHB depolymerase family esterase	WP_085258104.1
G6N56_RS22020	4674156	4675784		0	acyl-CoA dehydrogenase family protein	WP_085258103.1
G6N56_RS22025	4675791	4676729		0	crotonase/enoyl-CoA hydratase family protein	WP_085258102.1
G6N56_RS22030	4676732	4677463		0	PaaX family transcriptional regulator C-terminal domain-contai	WP_085258101.1
G6N56_RS22035	4677460	4678227		0	crotonase/enoyl-CoA hydratase family protein	WP_085258100.1
G6N56_RS22040	4678232	4678711		0	DUF3060 domain-containing protein	WP_085258099.1
G6N56_RS22045	4678745	4679113		0	DUF3060 domain-containing protein	WP_142280814.1
G6N56_RS22050	4679326	4679973		0	TetR/AcrR family transcriptional regulator	WP_085258097.1
G6N56_RS22055	4680201	4680575	rpsL		30S ribosomal protein S12	WP_007767794.1
G6N56_RS22060	4680575	4681045	rpsG		30S ribosomal protein S7	WP_085258096.1
G6N56_RS22065	4681117	4683222	fusA		elongation factor G	WP_085258095.1
G6N56_RS22070	4683334	4684524	tuf		elongation factor Tu	WP_085258094.1
G6N56_RS29545	4684692	4685393		0	PE family protein	WP_085258093.1
G6N56_RS22080	4685465	4686280		0	SHOCT domain-containing protein	WP_085258092.1
G6N56_RS22085	4686370	4687197		0	mycofactocin-coupled SDR family oxidoreductase	WP_085258091.1
G6N56_RS22090	4687207	4688424		0	FAD/NAD(P)-binding oxidoreductase	WP_085258090.1
G6N56_RS22095	4688421	4689428		0	D-2-hydroxyacid dehydrogenase family protein	WP_232069380.1
G6N56_RS22100	4689425	4690471		0	DUF2332 family protein	WP_085258089.1
G6N56_RS22105	4690468	4691064	mftR		mycofactocin system transcriptional regulator	WP_163645157.1
G6N56_RS22110	4691160	4691249	mftA		mycofactocin precursor MftA	WP_085258088.1
G6N56_RS22120	4691526	4692692	mftC		mycofactocin radical SAM maturase	WP_085258086.1

G6N56_RS22125	4692696	4693883	mftD		mycofactocin biosynthesis FMN-dependent deaminase MftD	WP_085258085.1
G6N56_RS22130	4694010	4694741	mftE		mycofactocin biosynthesis peptidyl-dipeptidase MftE	WP_085258084.1
G6N56_RS22135	4694738	4696150	mftF		mycofactocin biosynthesis glycosyltransferase MftF	WP_085258083.1
G6N56_RS22140	4696159	4697598	mftG		mycofactocin system GMC family oxidoreductase MftG	WP_085258082.1
G6N56_RS29550	4697615	4698442		0	PE family protein	WP_085258081.1
G6N56_RS22150	4698964	4700550		0	MFS transporter	WP_085258080.1
G6N56_RS22155	4700626	4701315		0	TetR/AcrR family transcriptional regulator	WP_085258079.1
G6N56_RS22160	4701767	4702072	rpsJ		30S ribosomal protein S10	WP_003873519.1
G6N56_RS22165	4702088	4702741	rplC		50S ribosomal protein L3	WP_085258078.1
G6N56_RS22170	4702741	4703406	rplD		50S ribosomal protein L4	WP_085258077.1
G6N56_RS22175	4703406	4703708	rplW		50S ribosomal protein L23	WP_085258076.1
G6N56_RS22180	4703738	4704580	rplB		50S ribosomal protein L2	WP_085258075.1
G6N56_RS22185	4704599	4704880	rpsS		30S ribosomal protein S19	WP_085258074.1
G6N56_RS22190	4704877	4705365	rplV		50S ribosomal protein L22	WP_085258073.1
G6N56_RS22195	4705365	4706189	rpsC		30S ribosomal protein S3	WP_085258072.1
G6N56_RS22200	4706193	4706609	rplP		50S ribosomal protein L16	WP_085258071.1
G6N56_RS22205	4706609	4706842	rpmC		50S ribosomal protein L29	WP_085258070.1
G6N56_RS22210	4706842	4707156	rpsQ		30S ribosomal protein S17	WP_085258069.1
G6N56_RS22215	4707280	4709631		0	arylsulfatase	WP_085258068.1
G6N56_RS22220	4709739	4710701		0	DUF4436 domain-containing protein	WP_163645158.1
G6N56_RS22225	4710712	4711833	pstS		phosphate ABC transporter substrate-binding protein PstS	WP_085257829.1
G6N56_RS22230	4712129	4713127		0	DUF4436 domain-containing protein	WP_085257830.1
G6N56_RS22235	4713362	4714216		0	DUF4436 domain-containing protein	WP_142280799.1
G6N56_RS22245	4715461	4716900		0	recombinase family protein	WP_180150392.1
G6N56_RS22250	4717108	4718637		0	plasmid replication initiator protein	WP_232069382.1
G6N56_RS22280	4723058	4723810		0	3-hydroxyacyl-CoA dehydrogenase	WP_042792154.1
G6N56_RS22285	4723876	4724958		0	CaiB/BaiF CoA-transferase family protein	WP_042792155.1
G6N56_RS22290	4725237	4725926		0	transglutaminase family protein	WP_042792156.1
G6N56_RS22295	4725997	4726260		0	hypothetical protein	WP_134770525.1
G6N56_RS22305	4726976	4728307		0	cytochrome P450	WP_042792157.1
G6N56_RS22310	4728373	4729485		0	NDMA-dependent alcohol dehydrogenase	WP_042792190.1

G6N56_RS22315	4729529	4730983	0	aldehyde dehydrogenase	WP_042792191.1	
G6N56_RS22320	4731495	4732508	0	phosphotransferase family protein	WP_158090667.1	
G6N56_RS22330	4733289	4734488	0	acyl-CoA dehydrogenase family protein	WP_085253971.1	
G6N56_RS22335	4734498	4735253	0	3-oxoacyl-ACP reductase family protein	WP_042792158.1	
G6N56_RS22340	4735261	4736454	0	thiolase family protein	WP_134770526.1	
G6N56_RS22345	4736507	4737448	0	PDR/VanB family oxidoreductase	WP_232069120.1	
G6N56_RS22350	4737610	4737954	0	hypothetical protein	WP_232069121.1	
G6N56_RS22355	4737951	4738247	0	hypothetical protein	WP_232069122.1	
G6N56_RS22360	4738244	4738966	0	phenylacetate-CoA oxygenase subunit PaaI	WP_042792197.1	
G6N56_RS22365	4739171	4739836	0	TetR/AcrR family transcriptional regulator	WP_042792160.1	
G6N56_RS22370	4740207	4740524	0	MaoC family dehydratase N-terminal domain-containing protei	WP_197746638.1	
G6N56_RS22375	4740554	4740868	0	MaoC family dehydratase		0
G6N56_RS22380	4740865	4741656	0	enoyl-CoA hydratase/isomerase family protein	WP_042792162.1	
G6N56_RS22385	4741653	4743017	0	AMP-binding protein	WP_042791964.1	
G6N56_RS22390	4743014	4743220	0	hypothetical protein	WP_042791965.1	
G6N56_RS22395	4743458	4744171	0	ABC transporter permease	WP_051472929.1	
G6N56_RS22405	4745030	4746436	0	MCE family protein	WP_042792163.1	
G6N56_RS22410	4746471	4747499	0	virulence factor Mce family protein	WP_042792164.1	
G6N56_RS22415	4747496	4748827	0	virulence factor Mce family protein	WP_085254014.1	
G6N56_RS22420	4748845	4750191	0	virulence factor Mce family protein	WP_042792321.1	
G6N56_RS22425	4750188	4751345	0	virulence factor Mce family protein	WP_042792201.1	
G6N56_RS22430	4751346	4752797	0	MlaD family protein	WP_042792202.1	
G6N56_RS22435	4752868	4753497	0	hypothetical protein	WP_042792203.1	
G6N56_RS22440	4753494	4754147	0	hypothetical protein	WP_042792204.1	
G6N56_RS22445	4754158	4754556	0	DUF732 domain-containing protein	WP_042792205.1	
G6N56_RS22450	4754729	4755142	0	hypothetical protein	WP_134770529.1	
G6N56_RS22455	4755625	4756320	0	CAP domain-containing protein	WP_042792207.1	
G6N56_RS22460	4756505	4757797	0	IS256 family transposase	WP_051472998.1	
G6N56_RS28580	4758896	4759948	0	hypothetical protein	WP_085253974.1	
G6N56_RS22480	4759948	4760256	0	hypothetical protein	WP_085253975.1	
G6N56_RS22485	4760520	4761062	0	DinB family protein	WP_085253976.1	

G6N56_RS22490	4761215	4761583	rplN		50S ribosomal protein L14	WP_003403649.1
G6N56_RS22495	4761584	4761901	rplX		50S ribosomal protein L24	WP_085253977.1
G6N56_RS22500	4761901	4762464	rplE		50S ribosomal protein L5	WP_085253978.1
G6N56_RS22505	4762468	4762653		0	type Z 30S ribosomal protein S14	WP_007167851.1
G6N56_RS22510	4762721	4763119	rpsH		30S ribosomal protein S8	WP_085253979.1
G6N56_RS22515	4763143	4763682	rplF		50S ribosomal protein L6	WP_085253980.1
G6N56_RS22520	4763685	4764092	rplR		50S ribosomal protein L18	WP_085253981.1
G6N56_RS22525	4764130	4764795	rpsE		30S ribosomal protein S5	WP_085253982.1
G6N56_RS22530	4764792	4764998	rpmD		50S ribosomal protein L30	WP_085253983.1
G6N56_RS22535	4764995	4765435	rplO		50S ribosomal protein L15	WP_085253984.1
G6N56_RS22540	4765436	4766365		0	LLM class flavin-dependent oxidoreductase	WP_085253985.1
G6N56_RS22545	4766400	4768172	sppA		signal peptide peptidase SppA	WP_163645159.1
G6N56_RS22555	4768169	4769080		0	class I SAM-dependent methyltransferase	WP_085253987.1
G6N56_RS22565	4769989	4770939		0	class I SAM-dependent methyltransferase	WP_085253989.1
G6N56_RS22570	4771007	4771942		0	MBL fold metallo-hydrolase	WP_085253990.1
G6N56_RS22575	4771942	4772604		0	L-fuculose-phosphate aldolase	WP_085253991.1
G6N56_RS22580	4772601	4773587		0	NAD(P)-dependent oxidoreductase	WP_085253992.1
G6N56_RS22585	4773600	4774982		0	FGGY-family carbohydrate kinase	WP_085253993.1
G6N56_RS22590	4774992	4775657		0	GNAT family N-acetyltransferase	WP_085253994.1
G6N56_RS22595	4775777	4776682		0	class I SAM-dependent methyltransferase	WP_163645161.1
G6N56_RS22600	4776903	4778228	secY		preprotein translocase subunit SecY	WP_085253996.1
G6N56_RS22605	4778225	4778770		0	adenylate kinase	WP_085253997.1
G6N56_RS22610	4778773	4779573	map		type I methionyl aminopeptidase	WP_085253998.1
G6N56_RS22615	4779627	4780172		0	sigma-70 family RNA polymerase sigma factor	WP_085253999.1
G6N56_RS22620	4780205	4780942		0	zf-HC2 domain-containing protein	WP_085254000.1
G6N56_RS22630	4781512	4782018		0	MarR family transcriptional regulator	WP_085254001.1
G6N56_RS22635	4782022	4782891		0	alpha/beta hydrolase	WP_158090668.1
G6N56_RS22640	4783004	4783609		0	TetR/AcrR family transcriptional regulator	WP_158090669.1
G6N56_RS22645	4783606	4784499	mmsB		3-hydroxyisobutyrate dehydrogenase	WP_085254003.1
G6N56_RS22650	4784510	4785679		0	isobutyryl-CoA dehydrogenase	WP_085254004.1
G6N56_RS22655	4785695	4787215		0	CoA-acylating methylmalonate-semialdehyde dehydrogenase	WP_085254005.1

G6N56_RS22665	4789332	4789904	0	TetR/AcrR family transcriptional regulator	WP_085254007.1
G6N56_RS22670	4789915	4791333	0	NAD(P)-binding protein	WP_085254008.1
G6N56_RS22675	4791350	4791889	0	sigma-70 family RNA polymerase sigma factor	WP_085254009.1
G6N56_RS22680	4791886	4792587	0	Rieske 2Fe-2S domain-containing protein	WP_085254010.1
G6N56_RS22685	4792617	4793000	0	cupredoxin family copper-binding protein	WP_085254011.1
G6N56_RS22690	4792997	4793923	0	metallophosphoesterase	WP_085254012.1
G6N56_RS22700	4794299	4796233	0	PE domain-containing protein	WP_163645162.1
G6N56_RS22705	4796350	4797762	0	PecA family PE domain-processing aspartic protease	WP_232069124.1
G6N56_RS22710	4797915	4798523	rfbC	dTDP-4-dehydrorhamnose 3,5-epimerase	WP_085255536.1
G6N56_RS22715	4798525	4799520	rfbB	dTDP-glucose 4,6-dehydratase	WP_085255537.1
G6N56_RS22720	4799608	4800453	0	LLM class F420-dependent oxidoreductase	WP_085255538.1
G6N56_RS22725	4800482	4801957	0	ATP-binding protein	WP_232069125.1
G6N56_RS22730	4801954	4803639	0	FAD-dependent oxidoreductase	WP_085255608.1
G6N56_RS22735	4803910	4804131	infA	translation initiation factor IF-1	WP_003418601.1
G6N56_RS22740	4804183	4804296	rpmJ	50S ribosomal protein L36	WP_003879483.1
G6N56_RS22745	4804448	4804822	rpsM	30S ribosomal protein S13	WP_008260257.1
G6N56_RS22750	4804829	4805248	rpsK	30S ribosomal protein S11	WP_085255539.1
G6N56_RS22755	4805262	4805867	rpsD	30S ribosomal protein S4	WP_085255540.1
G6N56_RS22760	4805942	4806985	0	DNA-directed RNA polymerase subunit alpha	WP_085255541.1
G6N56_RS22770	4807641	4808528	truA	tRNA pseudouridine(38-40) synthase TruA	WP_085255543.1
G6N56_RS22775	4808525	4809145	0	cutinase family protein	WP_085255544.1
G6N56_RS22780	4809203	4810102	0	cutinase family protein	WP_085255610.1
G6N56_RS22785	4810226	4811593	eccB	type VII secretion protein EccB	WP_085255545.1
G6N56_RS22795	4812844	4814241	eccD	type VII secretion integral membrane protein EccD	WP_142280589.1
G6N56_RS22800	4814333	4817875	eccCb	type VII secretion protein EccCb	WP_085255547.1
G6N56_RS22805	4817872	4819101	0	type VII secretion-associated protein	WP_085255612.1
G6N56_RS22810	4819144	4819461	0	WXG100 family type VII secretion target	WP_085255548.1
G6N56_RS22815	4819474	4819770	0	WXG100 family type VII secretion target	WP_085255549.1
G6N56_RS22820	4820006	4820449	rplM	50S ribosomal protein L13	WP_085255550.1
G6N56_RS22825	4820446	4820919	rpsI	30S ribosomal protein S9	WP_085255551.1
G6N56_RS22830	4821059	4822396	glmM	phosphoglucosamine mutase	WP_085255552.1

G6N56_RS22835	4822451	4822792		0	type VII secretion target	WP_085255553.1
G6N56_RS22840	4822795	4824090		0	hypothetical protein	WP_085255554.1
G6N56_RS22845	4824094	4825137		0	LLM class F420-dependent oxidoreductase	WP_085255555.1
G6N56_RS22850	4825190	4826029		0	hypothetical protein	WP_085255556.1
G6N56_RS22855	4826039	4826254		0	Rv1535 family protein	WP_085255557.1
G6N56_RS29260	4826777	4826986		0	DUF2510 domain-containing protein	WP_232069383.1
G6N56_RS29265	4827011	4827247		0	hypothetical protein	WP_232069126.1
G6N56_RS22865	4827261	4829126	glmS		glutamine--fructose-6-phosphate transaminase (isomerizing)	WP_085255558.1
G6N56_RS22870	4829155	4829970		0	DUF4436 domain-containing protein	WP_232069127.1
G6N56_RS22875	4830115	4830993		0	DUF4436 domain-containing protein	WP_085255559.1
G6N56_RS22880	4831048	4831761		0	hypothetical protein	WP_142280579.1
G6N56_RS22885	4831764	4833644		0	alpha/beta fold hydrolase	WP_142280580.1
G6N56_RS22890	4833641	4834561		0	class I SAM-dependent methyltransferase	WP_085255614.1
G6N56_RS22895	4834680	4836101		0	NAD(P)H-hydrate dehydratase	WP_085255615.1
G6N56_RS22900	4836117	4837508		0	glutamate decarboxylase	WP_180150590.1
G6N56_RS22905	4837572	4838747	alr		alanine racemase	WP_085255562.1
G6N56_RS22915	4839839	4840312	tsaE		tRNA (adenosine(37)-N6)-threonylcarbamoyltransferase comp	WP_142280582.1
G6N56_RS22920	4840309	4840929	tsaB		tRNA (adenosine(37)-N6)-threonylcarbamoyltransferase comp	WP_085255616.1
G6N56_RS22925	4840926	4841390	rimI		ribosomal protein S18-alanine N-acetyltransferase	WP_085255564.1
G6N56_RS22930	4841387	4842418	tsaD		tRNA (adenosine(37)-N6)-threonylcarbamoyltransferase comp	WP_085255565.1
G6N56_RS22935	4842656	4842958	groES		co-chaperone GroES	WP_007167930.1
G6N56_RS22940	4843044	4844666	groL		chaperonin GroEL	WP_085255566.1
G6N56_RS22945	4844904	4845668		0	TetR/AcrR family transcriptional regulator C-terminal domain-c	WP_232069128.1
G6N56_RS22950	4845687	4847198		0	aldehyde dehydrogenase family protein	WP_085255568.1
G6N56_RS22955	4847205	4848107		0	amidohydrolase family protein	WP_085255569.1
G6N56_RS22960	4848181	4848825		0	Crp/Fnr family transcriptional regulator	WP_085255618.1
G6N56_RS22965	4849052	4849342		0	hypothetical protein	WP_085255570.1
G6N56_RS22970	4849536	4850423		0	alpha/beta fold hydrolase	WP_085255571.1
G6N56_RS22975	4850553	4851794		0	fatty acid desaturase	WP_085255572.1
G6N56_RS22980	4852630	4853658		0	GAF domain-containing protein	WP_232069129.1
G6N56_RS22985	4853678	4854694	gap		type I glyceraldehyde-3-phosphate dehydrogenase	WP_085255619.1

G6N56_RS22990	4854808	4855260		0 hypothetical protein	WP_232069130.1
G6N56_RS22995	4855675	4857543		0 pyruvate kinase	WP_232069131.1
G6N56_RS23000	4857621	4860455		0 cation-transporting P-type ATPase	WP_085255575.1
G6N56_RS23005	4860482	4861168		0 TetR/AcrR family transcriptional regulator C-terminal domain-c	WP_232069384.1
G6N56_RS29270	4861417	4861869		0 hypothetical protein	WP_085255577.1
G6N56_RS23015	4861952	4862125		0 CsbD family protein	WP_085255578.1
G6N56_RS23020	4862176	4862355		0 DUF6131 family protein	WP_085255579.1
G6N56_RS23025	4862638	4863057		0 hypothetical protein	WP_180150395.1
G6N56_RS29275	4863422	4863913		0 hypothetical protein	WP_232069132.1
G6N56_RS23035	4864034	4865512		0 glycoside hydrolase family 130 protein	WP_163645163.1
G6N56_RS23040	4865509	4866531		0 glycosyltransferase	WP_085255581.1
G6N56_RS23045	4866528	4867673		0 glycosyltransferase	WP_142280592.1
G6N56_RS23050	4868186	4868938		0 GAF and ANTAR domain-containing protein	WP_232069133.1
G6N56_RS23055	4869296	4869796		0 STAS domain-containing protein	WP_232069134.1
G6N56_RS23060	4870118	4870519		0 hydrogenase	WP_085255584.1
G6N56_RS23065	4870574	4870882	whiB3	redox-responsive transcriptional regulator WhiB3	WP_085255585.1
G6N56_RS23070	4871246	4872052		0 hypothetical protein	WP_085255586.1
G6N56_RS23080	4872703	4873506		0 anti-sigma-D factor RsdA	WP_085255588.1
G6N56_RS23085	4873514	4873924		0 DUF5319 domain-containing protein	WP_085255589.1
G6N56_RS23090	4874099	4875700	guaB	IMP dehydrogenase	WP_085255622.1
G6N56_RS23095	4875708	4876835		0 GuaB3 family IMP dehydrogenase-related protein	WP_085255590.1
G6N56_RS23100	4876900	4878636		0 GMC family oxidoreductase	WP_085255591.1
G6N56_RS23105	4878641	4879537		0 TauD/TfdA family dioxygenase	WP_085255592.1
G6N56_RS23110	4879598	4880167		0 TetR/AcrR family transcriptional regulator	WP_085255593.1
G6N56_RS23115	4880171	4882534		0 glycoside hydrolase family 65 protein	WP_085255594.1
G6N56_RS23120	4882531	4883307		0 beta-phosphoglucomutase family hydrolase	WP_232069385.1
G6N56_RS23125	4883530	4885107	guaA	glutamine-hydrolyzing GMP synthase	WP_142280584.1
G6N56_RS23130	4885117	4885761		0 hypothetical protein	WP_211287386.1
G6N56_RS23135	4885861	4886538		0 hypothetical protein	WP_085255598.1
G6N56_RS23140	4886544	4888121		0 DNA polymerase Y family protein	WP_180150397.1
G6N56_RS23150	4888905	4889858		0 nucleoside hydrolase	WP_085255599.1

G6N56_RS23155	4889855	4890532		0	2OG-Fe dioxygenase family protein	WP_085255626.1
G6N56_RS23160	4890651	4892657		0	SDR family oxidoreductase	WP_085255600.1
G6N56_RS23165	4892709	4893416		0	histidine phosphatase family protein	WP_085255601.1
G6N56_RS23170	4893499	4894371		0	MaoC family dehydratase	WP_085255602.1
G6N56_RS23175	4894375	4895610	otsB		trehalose-phosphatase	WP_085255603.1
G6N56_RS23180	4895651	4897024		0	wax ester/triacylglycerol synthase family O-acyltransferase	WP_085255604.1
G6N56_RS23185	4897180	4900473		0	error-prone DNA polymerase	WP_180150399.1
G6N56_RS23190	4900637	4901281		0	nitroreductase family protein	WP_085255606.1
G6N56_RS23195	4901293	4901757		0	tRNA (cytidine(34)-2'-O)-methyltransferase	WP_085255607.1
G6N56_RS23200	4902013	4904757		0	sensor histidine kinase	WP_180150401.1
G6N56_RS23205	4904757	4905155		0	serine protease inhibitor	WP_085258436.1
G6N56_RS23215	4905518	4906105		0	ATP/GTP-binding protein	WP_085258434.1
G6N56_RS23220	4906102	4906671		0	pentapeptide repeat-containing protein	WP_085258433.1
G6N56_RS23225	4906676	4909285		0	ATP-binding cassette domain-containing protein	WP_085258432.1
G6N56_RS23230	4909322	4912000		0	ATP-binding cassette domain-containing protein	WP_085258431.1
G6N56_RS23235	4912031	4913239		0	NADH:flavin oxidoreductase	WP_085258430.1
G6N56_RS23240	4913334	4914179		0	bifunctional methylenetetrahydrofolate dehydrogenase/methe	WP_085258429.1
G6N56_RS23245	4914176	4914457		0	DUF3017 domain-containing protein	WP_085258428.1
G6N56_RS23250	4914454	4914927		0	DUF732 domain-containing protein	WP_085258427.1
G6N56_RS23255	4915210	4916649		0	FAD-binding oxidoreductase	WP_085258437.1
G6N56_RS23260	4916667	4917998		0	PPE family protein	WP_085258426.1
G6N56_RS23265	4918150	4919952		0	NAD-binding protein	WP_085258425.1
G6N56_RS23275	4922706	4924700		0	APC family permease	WP_085258423.1
G6N56_RS23285	4926254	4926988		0	class I SAM-dependent methyltransferase	WP_085253677.1
G6N56_RS23290	4926985	4928148		0	homoserine O-acetyltransferase	WP_085253672.1
G6N56_RS23295	4928159	4929511		0	bifunctional o-acetylhomoserine/o-acetylserine sulfhydrylase	WP_085253671.1
G6N56_RS23300	4929891	4931102		0	helix-turn-helix domain-containing protein	WP_085253670.1
G6N56_RS23305	4931416	4932429		0	choline/ethanolamine kinase family protein	WP_158090656.1
G6N56_RS23310	4932491	4932883		0	cupin domain-containing protein	WP_158090655.1
G6N56_RS23315	4932967	4934367		0	FAD-dependent oxidoreductase	WP_158090654.1
G6N56_RS23320	4934413	4935906		0	aldehyde dehydrogenase	WP_232069135.1

G6N56_RS23325	4935954	4936826		0	SDR family oxidoreductase	WP_085253665.1
G6N56_RS23330	4936938	4938398		0	cytosine permease	WP_085253664.1
G6N56_RS23335	4938395	4939081		0	histidine phosphatase family protein	WP_197746640.1
G6N56_RS23340	4939112	4940746		0	AMP-binding protein	WP_085253662.1
G6N56_RS23345	4940885	4943122		0	NADP-dependent isocitrate dehydrogenase	WP_085253661.1
G6N56_RS23350	4943150	4944385		0	NADP-dependent isocitrate dehydrogenase	WP_085253660.1
G6N56_RS23355	4944413	4945252		0	alpha/beta hydrolase	WP_085253659.1
G6N56_RS23360	4945462	4946481	trpS		tryptophan--tRNA ligase	WP_085253658.1
G6N56_RS23365	4946491	4947519	yhjD		inner membrane protein YhjD	WP_085253657.1
G6N56_RS23370	4947516	4948616	nagA		N-acetylglucosamine-6-phosphate deacetylase	WP_085253676.1
G6N56_RS23375	4948631	4949992		0	sugar porter family MFS transporter	WP_085253656.1
G6N56_RS23380	4949989	4951236		0	D-alanyl-D-alanine carboxypeptidase family protein	WP_085253655.1
G6N56_RS23385	4951323	4952654		0	aspartate aminotransferase family protein	WP_085253654.1
G6N56_RS23390	4952702	4953643		0	sigma-70 family RNA polymerase sigma factor	WP_085253653.1
G6N56_RS23400	4954287	4955087		0	succinate dehydrogenase iron-sulfur subunit	WP_085253651.1
G6N56_RS23405	4955087	4956841	sdhA		succinate dehydrogenase flavoprotein subunit	WP_085253650.1
G6N56_RS23410	4956858	4957325		0	succinate dehydrogenase hydrophobic membrane anchor subu	WP_085253649.1
G6N56_RS23415	4957322	4957660	sdhC		succinate dehydrogenase, cytochrome b556 subunit	WP_232069386.1
G6N56_RS23420	4957914	4958303		0	cytidine deaminase	WP_085253648.1
G6N56_RS23425	4958300	4959583		0	thymidine phosphorylase	WP_085253647.1
G6N56_RS23430	4959580	4960668		0	adenosine deaminase	WP_085253646.1
G6N56_RS23435	4960726	4961010		0	GlsB/YeaQ/YmgE family stress response membrane protein	WP_085253645.1
G6N56_RS23440	4961111	4961761		0	GntR family transcriptional regulator	WP_085253644.1
G6N56_RS23445	4961908	4962960		0	cupin	WP_085253643.1
G6N56_RS23450	4963028	4964035		0	VWA domain-containing protein	WP_085253674.1
G6N56_RS23455	4964040	4965023		0	VWA domain-containing protein	WP_085253642.1
G6N56_RS23460	4965146	4966468		0	primosomal protein	WP_085253641.1
G6N56_RS23465	4966511	4967347		0	alkaline phosphatase family protein	WP_085253673.1
G6N56_RS23470	4967489	4968112	upp		uracil phosphoribosyltransferase	WP_085253640.1
G6N56_RS23475	4968109	4969695		0	phospho-sugar mutase	WP_085253639.1
G6N56_RS23480	4969701	4970495		0	purine-nucleoside phosphorylase	WP_085253638.1

G6N56_RS23485	4970690	4972603	0	PPE family protein	WP_085253637.1
G6N56_RS23490	4972857	4973519	0	cutinase family protein	WP_085253636.1
G6N56_RS23495	4973566	4974750	0	M20 family metallopeptidase	WP_085253635.1
G6N56_RS23500	4974747	4975916	0	amidohydrolase	WP_085253634.1
G6N56_RS23510	4977126	4978013	0	hypothetical protein	WP_085255960.1
G6N56_RS23515	4978117	4978593	0	gamma-glutamylcyclotransferase	WP_085255915.1
G6N56_RS23520	4978670	4980085	0	NAD(P)H-quinone dehydrogenase	WP_085255916.1
G6N56_RS23525	4980169	4981923	0	glycerol-3-phosphate dehydrogenase/oxidase	WP_085255917.1
G6N56_RS23530	4981920	4982786	0	pseudouridine synthase	WP_085255918.1
G6N56_RS23535	4982930	4983343	0	limonene-1,2-epoxide hydrolase family protein	WP_085255919.1
G6N56_RS23540	4983438	4984079	0	TetR/AcrR family transcriptional regulator	WP_085255920.1
G6N56_RS23545	4984256	4985809	0	GMC family oxidoreductase	WP_085255961.1
G6N56_RS23550	4986365	4986907	0	PaaI family thioesterase	WP_158090718.1
G6N56_RS23555	4987663	4988661	0	hypothetical protein	WP_085255921.1
G6N56_RS23560	4988799	4989824	0	helix-turn-helix domain-containing protein	WP_158090719.1
G6N56_RS23565	4990610	4991749	0	PPE family protein	WP_085255923.1
G6N56_RS23570	4991830	4992975	0	PPE family protein	WP_085255924.1
G6N56_RS23575	4992977	4995316	0	heavy metal translocating P-type ATPase	WP_142280615.1
G6N56_RS23585	4996982	4999486	0	cation-translocating P-type ATPase	WP_085255925.1
G6N56_RS23595	5000082	5004512	0	cation-translocating P-type ATPase	WP_232069387.1
G6N56_RS23600	5004674	5005339	phoU	phosphate signaling complex protein PhoU	WP_085255927.1
G6N56_RS23605	5005354	5006343	0	magnesium transporter CorA family protein	WP_085255928.1
G6N56_RS23610	5006340	5008976	mgtA	magnesium-translocating P-type ATPase	WP_085255929.1
G6N56_RS23615	5008987	5011890	0	arylsulfatase	WP_085255965.1
G6N56_RS23620	5012196	5012525	0	DUF732 domain-containing protein	WP_085255931.1
G6N56_RS23625	5012666	5013016	0	DUF732 domain-containing protein	WP_085255932.1
G6N56_RS23630	5013221	5014138	0	PHB depolymerase family esterase	WP_085255933.1
G6N56_RS23635	5014214	5015317	pstS	phosphate ABC transporter substrate-binding protein PstS	WP_085255934.1
G6N56_RS23640	5015327	5015659	0	DUF732 domain-containing protein	WP_085255935.1
G6N56_RS23645	5015715	5016479	nei2	endonuclease VIII Nei2	WP_085255936.1
G6N56_RS23650	5016489	5021042	0	ATP-dependent helicase	WP_180150598.1

G6N56_RS23655	5021113	5021775		0	TetR/AcrR family transcriptional regulator	WP_085255938.1
G6N56_RS23660	5021939	5022322		0	hypothetical protein	WP_085255939.1
G6N56_RS23665	5022452	5022763	usfY		protein UsfY	WP_085255940.1
G6N56_RS23670	5023139	5023576		0	anti-sigma factor	WP_085255941.1
G6N56_RS23675	5023573	5024361		0	RNA polymerase sigma factor SigF	WP_085255942.1
G6N56_RS23685	5024923	5026725		0	acetyl/propionyl/methylcrotonyl-CoA carboxylase subunit alph	WP_085255944.1
G6N56_RS23690	5026857	5027279		0	SufE family protein	WP_085255945.1
G6N56_RS23695	5027276	5028169		0	sulfurtransferase	WP_085255966.1
G6N56_RS23700	5028401	5029063		0	nucleoside triphosphate pyrophosphatase	WP_085255946.1
G6N56_RS23705	5029060	5029320		0	acyl-CoA carboxylase subunit epsilon	WP_085255947.1
G6N56_RS23710	5029317	5030966		0	acyl-CoA carboxylase subunit beta	WP_085255948.1
G6N56_RS23715	5031018	5031833		0	biotin--[acetyl-CoA-carboxylase] ligase	WP_085255949.1
G6N56_RS23725	5032326	5033009		0	GtrA family protein	WP_085255951.1
G6N56_RS23730	5033127	5034362		0	5-(carboxyamino)imidazole ribonucleotide synthase	WP_264019827.1
G6N56_RS23740	5034949	5036118		0	acyl-CoA dehydrogenase	WP_085255954.1
G6N56_RS23745	5036351	5037496		0	fused response regulator/phosphatase	WP_142280617.1
G6N56_RS23750	5037568	5039082		0	sensor histidine kinase	WP_085255968.1
G6N56_RS23755	5039079	5039519		0	response regulator	WP_085255955.1
G6N56_RS23760	5039632	5040834		0	CoA transferase	WP_085255956.1
G6N56_RS23765	5040849	5041766		0	class I SAM-dependent methyltransferase	WP_085255957.1
G6N56_RS23770	5041903	5043612		0	AMP-binding protein	WP_085255958.1
G6N56_RS29280	5044139	5044315		0	hypothetical protein	WP_232069138.1
G6N56_RS23780	5044328	5045839		0	hypothetical protein	WP_163645166.1
G6N56_RS23785	5045854	5046150		0	PE family protein	WP_085254277.1
G6N56_RS23790	5046203	5047294		0	PPE domain-containing protein	WP_085254276.1
G6N56_RS23795	5047291	5048118		0	ESX secretion-associated protein EspG	WP_085254275.1
G6N56_RS23800	5048185	5048454		0	hypothetical protein	WP_142280452.1
G6N56_RS23805	5048471	5048755		0	hypothetical protein	WP_085254273.1
G6N56_RS28585	5048935	5049315		0	hypothetical protein	WP_180150403.1
G6N56_RS23815	5049333	5051501		0	hypothetical protein	WP_142280451.1
G6N56_RS23820	5051498	5052496		0	hypothetical protein	WP_142280450.1

G6N56_RS23825	5052505	5053266		0	ESX secretion-associated protein EspG	WP_163645167.1
G6N56_RS23830	5053290	5053763		0	YbaB/EbfC family nucleoid-associated protein	WP_085254268.1
G6N56_RS23835	5053766	5055109		0	WXG100 family type VII secretion target	WP_163645168.1
G6N56_RS23840	5055162	5093042		0	NUDIX domain-containing protein	WP_085254266.1
G6N56_RS23850	5094775	5096118	mycP		type VII secretion-associated serine protease mycosin	WP_085254294.1
G6N56_RS23855	5096148	5097563	eccD		type VII secretion integral membrane protein EccD	WP_085254264.1
G6N56_RS23860	5097560	5101591	eccCa		type VII secretion protein EccCa	WP_180150405.1
G6N56_RS23865	5101676	5103166	eccD		type VII secretion integral membrane protein EccD	WP_085254262.1
G6N56_RS23870	5103169	5104674	eccB		type VII secretion protein EccB	WP_085254293.1
G6N56_RS23875	5104792	5106480	eccE		type VII secretion protein EccE	WP_232069139.1
G6N56_RS23880	5106500	5107285		0	DUF5336 domain-containing protein	WP_085254260.1
G6N56_RS23885	5107341	5108291		0	alpha/beta hydrolase	WP_085254259.1
G6N56_RS23890	5108303	5109229		0	MinD/ParA family protein	WP_085254292.1
G6N56_RS23895	5109376	5111187	eccA		type VII secretion AAA-ATPase EccA	WP_085254258.1
G6N56_RS23900	5111355	5111672		0	hypothetical protein	WP_085254257.1
G6N56_RS23905	5111669	5112031		0	YbaB/EbfC family nucleoid-associated protein	WP_085254256.1
G6N56_RS23910	5112214	5112870		0	cation transporter	WP_085254255.1
G6N56_RS23915	5112875	5114449		0	PPE domain-containing protein	WP_085254254.1
G6N56_RS23920	5114883	5118011		0	TIR domain-containing protein	WP_085254253.1
G6N56_RS23925	5118060	5120192	ctpC		manganese-exporting P-type ATPase CtpC	WP_085254252.1
G6N56_RS23930	5120203	5120484		0	DUF1490 family protein	WP_085254251.1
G6N56_RS23940	5121278	5122747		0	LCP family protein	WP_085254250.1
G6N56_RS23950	5123725	5124615		0	glycosyltransferase family 2 protein	WP_085254248.1
G6N56_RS23955	5124617	5125696		0	NDP-sugar synthase	WP_085254247.1
G6N56_RS23960	5125825	5126253		0	hypothetical protein	WP_085254246.1
G6N56_RS23965	5126283	5126822		0	NUDIX hydrolase	WP_085254245.1
G6N56_RS23970	5126819	5126995		0	hypothetical protein	WP_158090678.1
G6N56_RS23975	5126998	5128368		0	coenzyme F420-0:L-glutamate ligase	WP_085254244.1
G6N56_RS23980	5128365	5129366	cofD		2-phospho-L-lactate transferase	WP_085254243.1
G6N56_RS23985	5129790	5130059		0	WhiB family transcriptional regulator	WP_163645229.1
G6N56_RS23990	5130081	5130503		0	metallopeptidase family protein	WP_085254242.1

G6N56_RS23995	5130682	5131143		0	DUF3499 domain-containing protein	WP_085254241.1
G6N56_RS24000	5131220	5132617		0	phosphomannomutase/phosphoglucomutase	WP_085254240.1
G6N56_RS24005	5132614	5133705		0	TobH protein	WP_085254239.1
G6N56_RS24010	5133715	5134941	manA		mannose-6-phosphate isomerase, class I	WP_085254238.1
G6N56_RS24015	5135058	5136476		0	oxidoreductase	WP_232069389.1
G6N56_RS24020	5136567	5138066		0	amino acid permease	WP_085254237.1
G6N56_RS24025	5138214	5139455		0	alkane 1-monooxygenase	WP_085254236.1
G6N56_RS24030	5139452	5139625		0	rubredoxin	WP_085254235.1
G6N56_RS24035	5139622	5139804		0	rubredoxin	WP_085254234.1
G6N56_RS24040	5139801	5140436		0	TetR family transcriptional regulator	WP_142280448.1
G6N56_RS24045	5140542	5142014	ahcY		adenosylhomocysteinase	WP_085254232.1
G6N56_RS24050	5142108	5142752		0	dTMP kinase	WP_085254231.1
G6N56_RS24055	5142822	5143508	mtrA		two-component system response regulator MtrA	WP_067925378.1
G6N56_RS24060	5143522	5145207	mtrB		MtrAB system histidine kinase MtrB	WP_085254230.1
G6N56_RS24065	5145218	5146963	lpqB		MtrAB system accessory lipoprotein LpqB	WP_180150600.1
G6N56_RS24070	5147245	5148153		0	alpha/beta fold hydrolase	WP_085254289.1
G6N56_RS24075	5148340	5148477		0	hypothetical protein	WP_158090677.1
G6N56_RS24080	5149045	5150313	oxlT		oxalate/formate MFS antiporter	WP_197746642.1
G6N56_RS24085	5150456	5151304		0	TIGR03621 family F420-dependent LLM class oxidoreductase	WP_085254288.1
G6N56_RS24090	5151449	5152081		0	ComF family protein	WP_085254227.1
G6N56_RS24095	5152440	5153141	raiA		ribosome-associated translation inhibitor RaiA	WP_085254226.1
G6N56_RS24100	5153249	5156080	secA		preprotein translocase subunit SecA	WP_085254225.1
G6N56_RS24105	5156172	5156915		0	hypothetical protein	WP_085254287.1
G6N56_RS24110	5156927	5158483		0	HD domain-containing phosphohydrolase	WP_085254286.1
G6N56_RS24115	5158553	5159317		0	hypothetical protein	WP_085254224.1
G6N56_RS24120	5159401	5159883		0	cation:proton antiporter regulatory subunit	WP_085254223.1
G6N56_RS24125	5159887	5161044		0	cation:proton antiporter	WP_085254222.1
G6N56_RS29285	5161053	5161409		0	Rv3235 family protein	WP_232069140.1
G6N56_RS24135	5161814	5163232		0	wax ester/triacylglycerol synthase family O-acyltransferase	WP_085254220.1
G6N56_RS24140	5163275	5164141	ppk2		polyphosphate kinase 2	WP_085254285.1
G6N56_RS24145	5164174	5164656		0	hypothetical protein	WP_085254219.1

G6N56_RS24150	5164845	5165993		0	ferredoxin reductase	WP_085254284.1
G6N56_RS24155	5166079	5167374		0	fatty acid desaturase	WP_085254218.1
G6N56_RS24160	5167488	5168744		0	acyl-CoA dehydrogenase family protein	WP_085254217.1
G6N56_RS24165	5168794	5169858		0	AraC family transcriptional regulator	WP_085254283.1
G6N56_RS24170	5169932	5171428		0	NAD(P)/FAD-dependent oxidoreductase	WP_085254216.1
G6N56_RS24175	5171727	5171879		0	hypothetical protein	WP_158090676.1
G6N56_RS24180	5171950	5172486		0	hypothetical protein	WP_085254215.1
G6N56_RS24185	5172526	5172909		0	hypothetical protein	WP_085254214.1
G6N56_RS24190	5173280	5173771		0	DUF302 domain-containing protein	WP_085254213.1
G6N56_RS24195	5173776	5174957		0	acetyl-CoA C-acyltransferase	WP_085254212.1
G6N56_RS24200	5175055	5175528		0	helix-turn-helix domain-containing protein	WP_085254211.1
G6N56_RS24205	5175617	5176603	rsgA		ribosome small subunit-dependent GTPase A	WP_085254210.1
G6N56_RS24210	5176600	5177895	aroA		3-phosphoshikimate 1-carboxyvinyltransferase	WP_232069141.1
G6N56_RS24215	5177951	5178712		0	SOS response-associated peptidase	WP_085254282.1
G6N56_RS24220	5178718	5180073		0	PE domain-containing protein	WP_085254281.1
G6N56_RS24225	5180229	5181104		0	3-hydroxyacyl-CoA dehydrogenase family protein	WP_085254208.1
G6N56_RS24230	5181311	5181796		0	aminoacyl-tRNA deacylase	WP_085254207.1
G6N56_RS24235	5181810	5182658		0	NAD(P)-dependent oxidoreductase	WP_085254206.1
G6N56_RS24240	5182848	5183615		0	sigma-70 family RNA polymerase sigma factor	WP_232069142.1
G6N56_RS24245	5183612	5183914	rsrA		mycothiol system anti-sigma-R factor	WP_085254204.1
G6N56_RS24250	5184205	5184420		0	biotin/lipoyl-binding carrier protein	WP_073876250.1
G6N56_RS24255	5184470	5185984		0	sensor histidine kinase	WP_085254203.1
G6N56_RS24260	5186043	5186297	whiB1		transcriptional regulator WhiB1	WP_085254202.1
G6N56_RS24265	5186567	5187541		0	diacylglycerol kinase family protein	WP_085254201.1
G6N56_RS24275	5188024	5188506		0	GNAT family N-acetyltransferase	WP_085254200.1
G6N56_RS24280	5188503	5189618		0	isochorismate synthase	WP_085254199.1
G6N56_RS24285	5189615	5190226		0	acid phosphatase	WP_085254198.1
G6N56_RS24290	5190303	5191103		0	AAA family ATPase	WP_085254197.1
G6N56_RS24295	5191181	5192422		0	hypothetical protein	WP_085254196.1
G6N56_RS24300	5192436	5193941		0	DEAD/DEAH box helicase	WP_085254195.1
G6N56_RS24305	5194222	5194935		0	ferritin-like fold-containing protein	WP_085254194.1

G6N56_RS24310	5194942	5195691		0	MmpS family transport accessory protein	WP_085254193.1
G6N56_RS24320	5196155	5196838		0	TetR/AcrR family transcriptional regulator	WP_085254192.1
G6N56_RS24325	5197029	5198063		0	DUF3152 domain-containing protein	WP_163645169.1
G6N56_RS24335	5199550	5200743	moeZ		adenylyltransferase/sulfurtransferase MoeZ	WP_232069143.1
G6N56_RS24340	5200890	5201744		0	TIGR02569 family protein	WP_085257939.1
G6N56_RS24345	5201761	5202063		0	MGMT family protein	WP_085257933.1
G6N56_RS24350	5202066	5202878		0	alpha/beta hydrolase	WP_085257932.1
G6N56_RS24360	5203225	5206386		0	ATP-dependent DNA helicase	WP_085257938.1
G6N56_RS24365	5206383	5209667		0	ATP-dependent DNA helicase	WP_085257937.1
G6N56_RS24370	5209761	5210831		0	potassium channel family protein	WP_085257930.1
G6N56_RS24380	5210980	5211903	nudC		NAD(+) diphosphatase	WP_085257928.1
G6N56_RS24385	5211908	5212564		0	sensor domain-containing protein	WP_085257927.1
G6N56_RS24390	5212655	5212906	mrx1		mycoredoxin Mrx1	WP_085257926.1
G6N56_RS24395	5213046	5215145		0	ATP-dependent DNA helicase UvrD2	WP_163645232.1
G6N56_RS28485	5215324	5215473		0	hypothetical protein	WP_169717549.1
G6N56_RS24400	5215608	5215868		0	WhiB family transcriptional regulator	WP_085257925.1
G6N56_RS24405	5215865	5217208		0	AarF/ABC1/UbiB kinase family protein	WP_085257935.1
G6N56_RS28490	5217315	5217488		0	hypothetical protein	WP_169717548.1
G6N56_RS24410	5217506	5218363		0	cyclodehydratase	WP_085257924.1
G6N56_RS24415	5218418	5219779		0	zinc-dependent metalloprotease	WP_142280805.1
G6N56_RS24420	5219858	5220880		0	PDZ domain-containing protein	WP_085257922.1
G6N56_RS24425	5220952	5223933		0	UPF0182 family protein	WP_085257921.1
G6N56_RS24430	5224025	5224999		0	GlxA family transcriptional regulator	WP_180150409.1
G6N56_RS24435	5225140	5225790		0	HD domain-containing protein	WP_085257919.1
G6N56_RS24440	5225817	5226467		0	HD domain-containing protein	WP_085257918.1
G6N56_RS24445	5226501	5226665		0	hypothetical protein	WP_158090766.1
G6N56_RS24450	5226672	5227451		0	IclR family transcriptional regulator	WP_163645170.1
G6N56_RS24455	5227545	5229338	gcl		glyoxylate carboligase	WP_085257916.1
G6N56_RS24460	5229344	5230228		0	2-hydroxy-3-oxopropionate reductase	WP_085257915.1
G6N56_RS24465	5230225	5231073		0	TIM barrel protein	WP_085257914.1
G6N56_RS24470	5231380	5232750		0	FAD-linked oxidase C-terminal domain-containing protein	WP_085257913.1

G6N56_RS24475	5232750	5234489	0	L-lactate permease	WP_085257912.1
G6N56_RS24480	5234489	5235631	0	glycerate kinase	WP_085257911.1
G6N56_RS24485	5235628	5236242	0	DsbA family protein	WP_085257910.1
G6N56_RS24505	5238392	5238625	0	hypothetical protein	WP_085257500.1
G6N56_RS24510	5239106	5239813	0	GAF and ANTAR domain-containing protein	WP_085257495.1
G6N56_RS24515	5239854	5240612	0	GAF and ANTAR domain-containing protein	WP_085257494.1
G6N56_RS24520	5240618	5241421	0	GAF and ANTAR domain-containing protein	WP_085257499.1
G6N56_RS24525	5241485	5243119	0	fatty acyl-AMP ligase	WP_085257498.1
G6N56_RS24530	5243880	5244272	0	hypothetical protein	WP_085257493.1
G6N56_RS24535	5246339	5246806	0	DUF5994 family protein	WP_085257491.1
G6N56_RS24540	5246980	5247783	0	transglutaminase family protein	WP_085257490.1
G6N56_RS24545	5248679	5250199	0	wax ester/triacylglycerol synthase family O-acyltransferase	WP_085257497.1
G6N56_RS24550	5250539	5250991	0	ATP-binding protein	WP_085257489.1
G6N56_RS24555	5251043	5251444	0	hypothetical protein	WP_232069144.1
G6N56_RS24560	5251959	5252393	0	SRPBCC family protein	WP_085257488.1
G6N56_RS24565	5252669	5253577	0	alpha/beta hydrolase	WP_085257487.1
G6N56_RS29555	5254119	5255891	0	PecA family PE domain-processing aspartic protease	WP_163645171.1
G6N56_RS24575	5255888	5256766	0	hypothetical protein	WP_197746643.1
G6N56_RS24580	5256870	5259011	0	PPE domain-containing protein	WP_085257941.1
G6N56_RS24585	5259019	5259792	0	alpha/beta fold hydrolase	WP_085257942.1
G6N56_RS24590	5260160	5261329	0	cytochrome P450	WP_142280806.1
G6N56_RS24595	5261457	5262611	0	cation:proton antiporter	WP_085257944.1
G6N56_RS24600	5262747	5263019	0	hypothetical protein	WP_085257945.1
G6N56_RS24605	5263148	5264146	0	NAD(P)H nitroreductase	WP_085257954.1
G6N56_RS24610	5264187	5264765	0	hypothetical protein	WP_085257946.1
G6N56_RS24615	5264842	5265957	0	4Fe-4S dicluster domain-containing protein	WP_085257947.1
G6N56_RS24625	5266805	5267572	0	oxidoreductase	WP_085257949.1
G6N56_RS24630	5267569	5268861	0	Ni/Fe hydrogenase subunit alpha	WP_085257950.1
G6N56_RS24635	5268858	5269340	0	hydrogenase maturation protease	WP_085257951.1
G6N56_RS24640	5269430	5270836	0	phosphatidylglycerol lysyltransferase domain-containing protei	WP_085257952.1
G6N56_RS24645	5270947	5271366	0	pyridoxamine 5'-phosphate oxidase family protein	WP_085256327.1

G6N56_RS24650	5271495	5272472		0	NAD(P)H nitroreductase	WP_085256328.1
G6N56_RS24655	5272601	5272864		0	phosphopantetheine-binding protein	WP_085256329.1
G6N56_RS24660	5272871	5274073		0	dihydrolipoamide acetyltransferase family protein	WP_085256330.1
G6N56_RS24665	5274070	5275047		0	alpha-ketoacid dehydrogenase subunit beta	WP_085256331.1
G6N56_RS24670	5275044	5276021	pdhA		pyruvate dehydrogenase (acetyl-transferring) E1 component su	WP_085256332.1
G6N56_RS24675	5276018	5277826	acsA		acetate--CoA ligase	WP_085256333.1
G6N56_RS24680	5278056	5279039		0	NAD(P)H nitroreductase	WP_085256334.1
G6N56_RS24685	5279083	5279352		0	DUF1918 domain-containing protein	WP_085256335.1
G6N56_RS24690	5279541	5279774		0	hypothetical protein	WP_085256336.1
G6N56_RS24695	5279878	5281929		0	erythromycin esterase family protein	WP_085256337.1
G6N56_RS24700	5281947	5282951		0	1-phosphofructokinase family hexose kinase	WP_180150411.1
G6N56_RS24705	5283015	5283779		0	universal stress protein	WP_085256338.1
G6N56_RS24710	5283794	5284627		0	universal stress protein	WP_085256339.1
G6N56_RS24715	5284725	5285396		0	response regulator transcription factor	WP_085256340.1
G6N56_RS24720	5285664	5287313		0	GAF domain-containing sensor histidine kinase	WP_180150605.1
G6N56_RS24725	5287399	5287920		0	GNAT family N-acetyltransferase	WP_085256342.1
G6N56_RS24730	5287934	5288818		0	universal stress protein	WP_085256343.1
G6N56_RS24735	5288900	5289244		0	ferredoxin family protein	WP_085256344.1
G6N56_RS24740	5289352	5290173		0	universal stress protein	WP_085256364.1
G6N56_RS24745	5290308	5294003	otsB		trehalose-phosphatase	WP_232069145.1
G6N56_RS24750	5294146	5295027		0	universal stress protein	WP_085256346.1
G6N56_RS24755	5295042	5296556		0	bifunctional aminoglycoside phosphotransferase/ATP-binding p	WP_085256347.1
G6N56_RS24765	5297588	5298688		0	3-oxoacyl-[acyl-carrier-protein] synthase III C-terminal domain-	WP_085256349.1
G6N56_RS24775	5299714	5302110	ppsA		phosphoenolpyruvate synthase	WP_085256351.1
G6N56_RS24780	5302307	5303728		0	wax ester/triacylglycerol synthase family O-acyltransferase	WP_085256352.1
G6N56_RS24790	5304465	5304914		0	HSP20 family small heat-shock protein	WP_085256354.1
G6N56_RS24795	5305058	5305450		0	helix-turn-helix transcriptional regulator	WP_085256355.1
G6N56_RS24800	5305568	5306113		0	DUF1360 domain-containing protein	WP_085256356.1
G6N56_RS24805	5306123	5307445		0	FAD-dependent oxidoreductase	WP_085256357.1
G6N56_RS24815	5308417	5309598		0	zinc-dependent alcohol dehydrogenase	WP_085256359.1
G6N56_RS24820	5309767	5311917	glgX		glycogen debranching protein GlgX	WP_085256360.1

G6N56_RS24825	5312075	5312266	0	hypothetical protein	WP_142280652.1
G6N56_RS24830	5312400	5313158	0	SDR family oxidoreductase	WP_085256365.1
G6N56_RS24835	5313172	5314143	0	aldo/keto reductase	WP_085256361.1
G6N56_RS24840	5314167	5315009	0	sensor domain-containing protein	WP_232069147.1
G6N56_RS24845	5315747	5316307	0	hemerythrin domain-containing protein	WP_085254718.1
G6N56_RS24855	5317005	5318798	0	thiamine pyrophosphate-requiring protein	WP_085254716.1
G6N56_RS29305	5319059	5319418	0	UDP-glucose/GDP-mannose dehydrogenase family protein	WP_264020214.1
G6N56_RS24875	5319962	5321038	0	GDP-mannose 4,6-dehydratase	WP_085254715.1
G6N56_RS24880	5321037	5321330	0	glycosyltransferase	WP_085254714.1
G6N56_RS24885	5321435	5322490	0	DNA topoisomerase IB	WP_085254713.1
G6N56_RS24890	5322625	5323023	0	heme-binding protein	WP_085254712.1
G6N56_RS24900	5324036	5324278	0	CsbD family protein	WP_085254721.1
G6N56_RS24905	5324452	5324739	0	DUF3349 domain-containing protein	WP_085254711.1
G6N56_RS24910	5324752	5325516	0	endonuclease/exonuclease/phosphatase family protein	WP_085254710.1
G6N56_RS24915	5325575	5326201	0	SAM-dependent methyltransferase	WP_085254709.1
G6N56_RS24920	5326198	5326971	0	PIG-L deacetylase family protein	WP_085254708.1
G6N56_RS24925	5326979	5327920	0	acyl-CoA dehydrogenase family protein	WP_085254707.1
G6N56_RS24930	5327917	5328624	0	glycosyltransferase	WP_085254706.1
G6N56_RS24935	5328784	5329353	0	nucleotidyltransferase	WP_085254705.1
G6N56_RS24940	5329360	5329617	0	Rho termination factor	WP_085254704.1
G6N56_RS24945	5329754	5330767	0	chemotaxis protein CheB	WP_085254703.1
G6N56_RS24950	5330764	5331522	0	sulfate transporter	WP_085254702.1
G6N56_RS24955	5331576	5333414	0	CheR family methyltransferase	WP_085254720.1
G6N56_RS24960	5333453	5334355	0	chemotaxis protein CheB	WP_085254719.1
G6N56_RS24965	5334535	5335716	0	glycosyltransferase	WP_085254701.1
G6N56_RS24970	5335758	5336855	0	helix-turn-helix domain-containing protein	WP_085254700.1
G6N56_RS24975	5337282	5337671	0	STAS domain-containing protein	WP_232069148.1
G6N56_RS24980	5337990	5339333	0	FAD-dependent oxidoreductase	WP_085254698.1
G6N56_RS24990	5340446	5341792	0	flavin monoamine oxidase family protein	WP_085254696.1
G6N56_RS24995	5341879	5342877	0	TIGR03617 family F420-dependent LLM class oxidoreductase	WP_085254695.1
G6N56_RS25000	5342979	5344247	0	MFS transporter	WP_232069393.1

G6N56_RS25005	5344252	5345610		0	LLM class flavin-dependent oxidoreductase	WP_142280497.1
G6N56_RS25010	5345718	5346833		0	hypothetical protein	WP_085254692.1
G6N56_RS25015	5346833	5347975		0	phosphotransferase family protein	WP_085254691.1
G6N56_RS25020	5348031	5348681		0	TetR/AcrR family transcriptional regulator	WP_085254690.1
G6N56_RS25025	5348678	5349544		0	SDR family oxidoreductase	WP_085254689.1
G6N56_RS25030	5349562	5350947		0	HNH endonuclease signature motif containing protein	WP_085254688.1
G6N56_RS25040	5352384	5353337		0	DUF4129 domain-containing protein	WP_232069149.1
G6N56_RS25045	5353334	5353816		0	hypothetical protein	WP_085255159.1
G6N56_RS25050	5353813	5354772		0	MoxR family ATPase	WP_085255158.1
G6N56_RS25055	5354780	5356048		0	DUF58 domain-containing protein	WP_085255157.1
G6N56_RS25060	5356045	5356497		0	hypothetical protein	WP_085255156.1
G6N56_RS25065	5356508	5358124	nuoN		NADH-quinone oxidoreductase subunit NuoN	WP_085255155.1
G6N56_RS25070	5358121	5359701		0	NADH-quinone oxidoreductase subunit M	WP_085255154.1
G6N56_RS25075	5359766	5361652	nuoL		NADH-quinone oxidoreductase subunit L	WP_085255153.1
G6N56_RS25080	5361663	5361962	nuoK		NADH-quinone oxidoreductase subunit NuoK	WP_008259456.1
G6N56_RS25085	5361962	5362738		0	NADH-quinone oxidoreductase subunit J	WP_085255152.1
G6N56_RS25095	5363273	5364535	nuoH		NADH-quinone oxidoreductase subunit NuoH	WP_085255150.1
G6N56_RS25100	5364532	5366946		0	NADH-quinone oxidoreductase subunit G	WP_085255149.1
G6N56_RS25105	5367058	5368386	nuoF		NADH-quinone oxidoreductase subunit NuoF	WP_085255148.1
G6N56_RS25110	5368383	5369153	nuoE		NADH-quinone oxidoreductase subunit NuoE	WP_085255147.1
G6N56_RS25115	5369150	5370487	nuoD		NADH dehydrogenase (quinone) subunit D	WP_085255146.1
G6N56_RS25120	5370487	5371215		0	NADH-quinone oxidoreductase subunit C	WP_085255145.1
G6N56_RS25125	5371212	5371766		0	NADH-quinone oxidoreductase subunit B	WP_085255144.1
G6N56_RS25130	5371775	5372152		0	NADH-quinone oxidoreductase subunit A	WP_085255143.1
G6N56_RS25135	5372459	5372860		0	response regulator transcription factor	WP_085255142.1
G6N56_RS25140	5372891	5373427		0	YceI family protein	WP_085255141.1
G6N56_RS25145	5373457	5373846		0	nuclear transport factor 2 family protein	WP_085255140.1
G6N56_RS29315	5373873	5374220		0	DUF6285 domain-containing protein	WP_142280528.1
G6N56_RS25150	5374217	5375212		0	phosphotransferase family protein	WP_142280527.1
G6N56_RS25155	5375212	5376450		0	acyl-CoA dehydrogenase family protein	WP_085255139.1
G6N56_RS25160	5376461	5377081		0	TetR/AcrR family transcriptional regulator	WP_085255138.1

G6N56_RS25165	5377081	5377965		0	hydroxymethylglutaryl-CoA lyase	WP_085255189.1
G6N56_RS25170	5377971	5379176		0	CoA transferase	WP_085255137.1
G6N56_RS25175	5379176	5380366		0	alpha/beta fold hydrolase	WP_085255136.1
G6N56_RS25180	5380363	5381457		0	homogentisate 1,2-dioxygenase	WP_085255135.1
G6N56_RS25185	5381664	5382635		0	NADPH:quinone oxidoreductase family protein	WP_085255134.1
G6N56_RS25190	5382658	5383656		0	acryloyl-CoA reductase	WP_085255133.1
G6N56_RS25200	5383858	5385069		0	acyl-CoA dehydrogenase family protein	WP_085255132.1
G6N56_RS25205	5385097	5386500		0	acyl-CoA dehydrogenase family protein	WP_085255131.1
G6N56_RS25210	5386584	5387363	hisN		histidinol-phosphatase	WP_085255130.1
G6N56_RS25215	5387409	5387738		0	hypothetical protein	WP_085255188.1
G6N56_RS25220	5388315	5390030		0	GAF domain-containing protein	WP_085255129.1
G6N56_RS25225	5390079	5390282		0	hypothetical protein	WP_085255128.1
G6N56_RS25230	5390289	5391656		0	FAD-dependent oxidoreductase	WP_085255127.1
G6N56_RS25235	5391830	5392951	prfB		peptide chain release factor 2	WP_085255126.1
G6N56_RS25245	5393924	5394418		0	hypothetical protein	WP_085255124.1
G6N56_RS25250	5394463	5395152	ftsE		cell division ATP-binding protein FtsE	WP_085255187.1
G6N56_RS25255	5395153	5396046	ftsX		permease-like cell division protein FtsX	WP_085255123.1
G6N56_RS25260	5396049	5396558	smpB		SsrA-binding protein SmpB	WP_085255122.1
G6N56_RS25265	5396651	5397400		0	EamA family transporter	WP_180150607.1
G6N56_RS25270	5397470	5398315		0	maleylpyruvate isomerase family mycothiol-dependent enzyme	WP_085255121.1
G6N56_RS25275	5398540	5399502		0	hypothetical protein	WP_085255119.1
G6N56_RS25285	5400217	5400777		0	ERG2 family protein	WP_158090713.1
G6N56_RS25290	5400822	5401493		0	TetR/AcrR family transcriptional regulator	WP_085255117.1
G6N56_RS25295	5401683	5402612		0	metal-dependent hydrolase	WP_085255116.1
G6N56_RS25315	5405584	5406237		0	Bax inhibitor-1 family protein	WP_085255114.1
G6N56_RS25320	5406354	5406824		0	DUF1003 domain-containing protein	WP_232069150.1
G6N56_RS25325	5406938	5407681		0	YoaK family protein	WP_085255113.1
G6N56_RS25330	5408238	5408552		0	metal-sensitive transcriptional regulator	WP_232069151.1
G6N56_RS25335	5408773	5411325		0	heavy metal translocating P-type ATPase	WP_085255183.1
G6N56_RS25340	5411414	5411632		0	hypothetical protein	WP_142280525.1
G6N56_RS25345	5411647	5411901		0	SHOCT domain-containing protein	WP_085255182.1

G6N56_RS25350	5412055	5412435		0	hypothetical protein	WP_085255111.1
G6N56_RS25360	5413879	5414742		0	universal stress protein	WP_163645172.1
G6N56_RS25365	5415134	5416600		0	amidase	WP_158090712.1
G6N56_RS25370	5417181	5418440		0	acetamidase/formamidase family protein	WP_085255107.1
G6N56_RS25375	5418580	5420112		0	serine hydrolase domain-containing protein	WP_232069152.1
G6N56_RS25380	5420120	5420791		0	M15 family metallopeptidase	WP_085255106.1
G6N56_RS25385	5420827	5422326		0	NAD(P)-binding domain-containing protein	WP_085255105.1
G6N56_RS25390	5422437	5422814		0	DUF732 domain-containing protein	WP_142280532.1
G6N56_RS25395	5422846	5423361		0	DUF732 domain-containing protein	WP_142280524.1
G6N56_RS25400	5423524	5424138		0	DUF732 domain-containing protein	WP_085255103.1
G6N56_RS25405	5424209	5424913		0	TetR/AcrR family transcriptional regulator	WP_085255102.1
G6N56_RS25410	5425009	5425728		0	TetR/AcrR family transcriptional regulator	WP_085255101.1
G6N56_RS25420	5426347	5427792		0	YdiU family protein	WP_180150609.1
G6N56_RS25425	5427870	5428436		0	hemerythrin domain-containing protein	WP_085255098.1
G6N56_RS25430	5428999	5429916		0	CoA ester lyase	WP_085255179.1
G6N56_RS25435	5429934	5430626		0	DsbA family protein	WP_085255097.1
G6N56_RS25440	5430657	5431286		0	GNAT family N-acetyltransferase	WP_085255096.1
G6N56_RS25445	5431297	5431671	crcB		fluoride efflux transporter CrcB	WP_085255095.1
G6N56_RS25450	5431668	5432066	crcB		fluoride efflux transporter CrcB	WP_085255094.1
G6N56_RS25455	5432206	5433834	pgm		phosphoglucomutase (alpha-D-glucose-1,6-bisphosphate-dep	WP_085255093.1
G6N56_RS28595	5434080	5434370		0	hypothetical protein	WP_158090711.1
G6N56_RS25470	5434499	5434966		0	nuclear transport factor 2 family protein	WP_085255092.1
G6N56_RS25475	5435020	5436099		0	alpha/beta hydrolase	WP_197746644.1
G6N56_RS25480	5436096	5436959		0	alpha/beta hydrolase	WP_085255090.1
G6N56_RS25485	5437107	5437763		0	CGNR zinc finger domain-containing protein	WP_085255089.1
G6N56_RS25490	5438047	5438562		0	TetR/AcrR family transcriptional regulator	WP_085255088.1
G6N56_RS25495	5438688	5439338		0	NAD(P)H-dependent oxidoreductase	WP_085255087.1
G6N56_RS25500	5439887	5440336		0	helix-turn-helix domain-containing protein	WP_085255178.1
G6N56_RS25505	5440428	5441261		0	enoyl-CoA hydratase/isomerase family protein	WP_085255177.1
G6N56_RS25510	5441351	5442544		0	acyl-CoA dehydrogenase family protein	WP_085255086.1
G6N56_RS25515	5442802	5443635		0	mycofactocin-coupled SDR family oxidoreductase	WP_085255085.1

G6N56_RS25520	5444438	5445262	0	enoyl-CoA hydratase/isomerase family protein	WP_085255084.1
G6N56_RS25525	5445523	5446770	0	FAD-binding protein	WP_158090710.1
G6N56_RS25530	5446831	5447709	0	LLM class F420-dependent oxidoreductase	WP_085255176.1
G6N56_RS25535	5448111	5448509	0	nuclear transport factor 2 family protein	WP_158090709.1
G6N56_RS25540	5448954	5449724	0	SDR family oxidoreductase	WP_085255081.1
G6N56_RS25545	5449752	5450573	0	mycofactocin-coupled SDR family oxidoreductase	WP_085255080.1
G6N56_RS25555	5451708	5452871	0	sulfotransferase	WP_085255078.1
G6N56_RS25560	5452873	5453748	0	SDR family NAD(P)-dependent oxidoreductase	WP_085255077.1
G6N56_RS25565	5453870	5454823	0	LysR family transcriptional regulator	WP_085255076.1
G6N56_RS25570	5455160	5455936	0	SDR family NAD(P)-dependent oxidoreductase	WP_085255075.1
G6N56_RS25575	5456010	5456855	0	mycofactocin-coupled SDR family oxidoreductase	WP_085255175.1
G6N56_RS25580	5457066	5458220	0	amidohydrolase family protein	WP_085255074.1
G6N56_RS25585	5458312	5459058	0	SDR family NAD(P)-dependent oxidoreductase	WP_085255073.1
G6N56_RS25590	5459086	5459571	0	polyketide cyclase	WP_085255072.1
G6N56_RS25595	5459800	5461269	0	aldehyde dehydrogenase	WP_085255071.1
G6N56_RS25600	5461371	5462474	0	dihydrodipicolinate reductase	WP_085255070.1
G6N56_RS25605	5462900	5463580	0	hypothetical protein	WP_085255068.1
G6N56_RS25610	5463648	5464589	0	hypothetical protein	WP_142280523.1
G6N56_RS25615	5464680	5465177	0	nuclear transport factor 2 family protein	WP_085255066.1
G6N56_RS25620	5465205	5466314	0	NAD(P)-dependent oxidoreductase	WP_085255065.1
G6N56_RS25625	5466352	5466975	0	TetR/AcrR family transcriptional regulator	WP_197746645.1
G6N56_RS25630	5467226	5467873	0	TetR/AcrR family transcriptional regulator	WP_085255064.1
G6N56_RS25635	5467936	5468934	0	Rieske 2Fe-2S domain-containing protein	WP_085255063.1
G6N56_RS25640	5469070	5470260	0	phosphotransferase family protein	WP_085255062.1
G6N56_RS25645	5470282	5471124	0	SDR family oxidoreductase	WP_085255173.1
G6N56_RS25650	5471154	5472011	0	enoyl-CoA hydratase/isomerase family protein	WP_085255061.1
G6N56_RS25655	5472066	5472836	0	glucose 1-dehydrogenase	WP_085255060.1
G6N56_RS25660	5472960	5474216	0	cytochrome P450	WP_085255059.1
G6N56_RS25665	5474495	5475124	0	TetR/AcrR family transcriptional regulator	WP_085255058.1
G6N56_RS25670	5475172	5476530	0	cytochrome P450	WP_085255172.1
G6N56_RS25675	5476533	5476739	0	ferredoxin	WP_085255057.1

G6N56_RS25680	5476755	5477633	0	LLM class F420-dependent oxidoreductase	WP_085255056.1
G6N56_RS25685	5477646	5478470	0	IclR family transcriptional regulator	WP_142280522.1
G6N56_RS25690	5478475	5479359	0	TIGR03619 family F420-dependent LLM class oxidoreductase	WP_085255054.1
G6N56_RS25700	5480501	5481859	0	hypothetical protein	WP_085255052.1
G6N56_RS25705	5481890	5482528	0	nuclear transport factor 2 family protein	WP_180150419.1
G6N56_RS25710	5482525	5483382	0	SDR family oxidoreductase	WP_085255051.1
G6N56_RS25715	5483458	5484195	0	SDR family oxidoreductase	WP_085255050.1
G6N56_RS25720	5484361	5485269	0	LysR family transcriptional regulator	WP_085255049.1
G6N56_RS25725	5485415	5485609	0	long chain fatty acid-CoA synthetase Faa4p	WP_085255048.1
G6N56_RS25730	5485698	5485970	0	hypothetical protein	WP_085255047.1
G6N56_RS25735	5485986	5486726	0	SDR family oxidoreductase	WP_085255171.1
G6N56_RS25740	5486732	5489560	0	RND family transporter	WP_085255046.1
G6N56_RS25745	5489557	5489955	0	MmpS family transport accessory protein	WP_085255045.1
G6N56_RS25750	5490199	5490624	0	hypothetical protein	WP_085255044.1
G6N56_RS25755	5490726	5490998	0	hypothetical protein	WP_085255043.1
G6N56_RS25760	5491090	5493570	0	glycogen/starch/alpha-glucan phosphorylase	WP_085255042.1
G6N56_RS25765	5493747	5494763	0	NAD(P)/FAD-dependent oxidoreductase	WP_085255041.1
G6N56_RS25770	5494760	5495269	0	isoprenylcysteine carboxylmethyltransferase family protein	WP_085255040.1
G6N56_RS25775	5495277	5496383	0	3-oxoacyl-[acyl-carrier-protein] synthase III C-terminal domain-	WP_085255039.1
G6N56_RS25780	5496389	5498650	0	MMPL family transporter	WP_085255170.1
G6N56_RS25785	5498854	5499420	0	TetR/AcrR family transcriptional regulator	WP_085255038.1
G6N56_RS25790	5499417	5499980	0	TetR family transcriptional regulator	WP_085255037.1
G6N56_RS25795	5500049	5500762	0	hypothetical protein	WP_085255036.1
G6N56_RS25800	5500822	5501145	0	multidrug efflux SMR transporter	WP_085255035.1
G6N56_RS28600	5501294	5501647	0	hypothetical protein	WP_158090708.1
G6N56_RS25810	5501725	5502147	0	DoxX family protein	WP_085255034.1
G6N56_RS25815	5502203	5502421	0	hypothetical protein	WP_232069154.1
G6N56_RS25820	5502660	5503304	0	MspA family porin	WP_085255169.1
G6N56_RS25825	5503344	5505161	0	glycoside hydrolase family 15 protein	WP_085255032.1
G6N56_RS25830	5505242	5506198	0	LLM class F420-dependent oxidoreductase	WP_085255031.1
G6N56_RS25835	5506202	5507032	0	oxidoreductase	WP_085255030.1

G6N56_RS25840	5507362	5508174	ppk2		polyphosphate kinase 2	WP_232069155.1
G6N56_RS25845	5508207	5508872		0	nitroreductase family protein	WP_085255029.1
G6N56_RS25850	5508880	5510418		0	Hsp70 family protein	WP_085255028.1
G6N56_RS25855	5510616	5511002		0	STAS/SEC14 domain-containing protein	WP_232069395.1
G6N56_RS25860	5510999	5511508		0	CAP domain-containing protein	WP_085255026.1
G6N56_RS25865	5511577	5511873		0	DUF732 domain-containing protein	WP_232069396.1
G6N56_RS25870	5511980	5512747		0	ABC transporter permease	WP_085255024.1
G6N56_RS25875	5512744	5513601		0	ABC transporter permease	WP_085255023.1
G6N56_RS25880	5513608	5515038		0	MCE family protein	WP_085255022.1
G6N56_RS25885	5515071	5516099		0	MCE family protein	WP_085255021.1
G6N56_RS25890	5516096	5517352		0	MCE family protein	WP_085255168.1
G6N56_RS25895	5517349	5518605		0	MCE family protein	WP_085255020.1
G6N56_RS25900	5518602	5519735		0	virulence factor Mce family protein	WP_085255019.1
G6N56_RS25905	5519737	5521176		0	MlaD family protein	WP_085255018.1
G6N56_RS25915	5521685	5522230		0	hypothetical protein	WP_085255016.1
G6N56_RS25920	5522234	5524486		0	carbon starvation CstA family protein	WP_085255015.1
G6N56_RS25925	5524524	5526053		0	ATP-dependent DNA ligase	WP_085255014.1
G6N56_RS25930	5526165	5526923		0	SDR family NAD(P)-dependent oxidoreductase	WP_085255013.1
G6N56_RS25935	5527030	5529207		0	acyl-CoA dehydrogenase	WP_085255012.1
G6N56_RS25940	5529309	5529653		0	OB-fold domain-containing protein	WP_232069156.1
G6N56_RS25945	5529717	5530907		0	thiolase family protein	WP_085255011.1
G6N56_RS25950	5530929	5532377		0	FCD domain-containing protein	WP_085255166.1
G6N56_RS25955	5532485	5533477		0	sensor histidine kinase	WP_180150421.1
G6N56_RS25960	5533991	5534512		0	homocitrate synthase	WP_085255009.1
G6N56_RS25965	5534523	5536004		0	cytochrome P450	WP_085255008.1
G6N56_RS25970	5536131	5536778		0	TetR/AcrR family transcriptional regulator	WP_085255007.1
G6N56_RS25980	5537673	5538707		0	DNA polymerase IV	WP_085255005.1
G6N56_RS25985	5538707	5539315		0	TetR/AcrR family transcriptional regulator	WP_085255004.1
G6N56_RS25990	5539406	5539978		0	NADPH-dependent FMN reductase	WP_163645174.1
G6N56_RS25995	5540536	5540775	nrdH		glutaredoxin-like protein NrdH	WP_085255002.1
G6N56_RS26005	5541311	5543476	nrdE		class 1b ribonucleoside-diphosphate reductase subunit alpha	WP_085255000.1

G6N56_RS26010	5543615	5544358		0	TetR/AcrR family transcriptional regulator	WP_142280521.1
G6N56_RS26015	5544415	5545986		0	MFS transporter	WP_180150423.1
G6N56_RS26020	5546085	5546750		0	TetR/AcrR family transcriptional regulator	WP_232069157.1
G6N56_RS26025	5546851	5548422		0	NAD(P)/FAD-dependent oxidoreductase	WP_085254999.1
G6N56_RS26035	5549423	5549995		0	TetR/AcrR family transcriptional regulator	WP_085254998.1
G6N56_RS26045	5550504	5550950		0	SRPBCC family protein	WP_085254996.1
G6N56_RS26050	5551193	5552569		0	HNH endonuclease signature motif containing protein	WP_085254995.1
G6N56_RS26055	5552721	5553695	nrdF		class 1b ribonucleoside-diphosphate reductase subunit beta	WP_180150425.1
G6N56_RS26060	5553798	5554286		0	hypothetical protein	WP_085254993.1
G6N56_RS26065	5554424	5554864		0	DUF3349 domain-containing protein	WP_085254992.1
G6N56_RS26070	5554867	5555907		0	NAD(P)-dependent alcohol dehydrogenase	WP_085254991.1
G6N56_RS26075	5556046	5557125		0	iron-siderophore ABC transporter substrate-binding protein	WP_180150616.1
G6N56_RS26085	5558630	5560390	ctaD		cytochrome c oxidase subunit I	WP_085253856.1
G6N56_RS26090	5560414	5561652	serB		phosphoserine phosphatase SerB	WP_085253851.1
G6N56_RS26095	5561773	5562567		0	ABC transporter ATP-binding protein	WP_232069398.1
G6N56_RS26100	5562564	5563415		0	NUDIX hydrolase	WP_085253849.1
G6N56_RS26115	5565074	5566306		0	SAM-dependent methyltransferase	WP_085253855.1
G6N56_RS26120	5566379	5567071		0	RsiV family protein	WP_085253846.1
G6N56_RS26125	5567111	5567509		0	DUF6264 family protein	WP_232069399.1
G6N56_RS26130	5567506	5568783		0	PQQ-binding-like beta-propeller repeat protein	WP_180150620.1
G6N56_RS26135	5568985	5569722		0	acyltransferase	WP_085253843.1
G6N56_RS26145	5570103	5571431		0	deoxyribodipyrimidine photo-lyase	WP_085253842.1
G6N56_RS26150	5571445	5571924		0	TspO/MBR family protein	WP_085253841.1
G6N56_RS26155	5571921	5574023		0	MMPL family transporter	WP_232069158.1
G6N56_RS26160	5574157	5575203	fni		type 2 isopentenyl-diphosphate Delta-isomerase	WP_085253840.1
G6N56_RS26165	5575214	5576464		0	glycosyltransferase	WP_085253839.1
G6N56_RS26170	5576461	5577024		0	MarR family transcriptional regulator	WP_085253838.1
G6N56_RS26175	5577189	5577422		0	DUF3140 domain-containing protein	WP_232069400.1
G6N56_RS26180	5577875	5578273		0	hypothetical protein	WP_085253837.1
G6N56_RS26185	5578440	5578769		0	lipopolysaccharide assembly protein LapA domain-containing p	WP_085253836.1
G6N56_RS26190	5578855	5579094		0	hypothetical protein	WP_085253835.1

G6N56_RS26195	5579091	5579456		0	DUF2784 domain-containing protein	WP_085253834.1	
G6N56_RS26200	5579453	5580451		0	DUF5914 domain-containing protein	WP_085253833.1	
G6N56_RS26205	5580448	5581977		0	FAD-dependent oxidoreductase	WP_085253832.1	
G6N56_RS26210	5581974	5582699		0	class I SAM-dependent methyltransferase	WP_085253831.1	
G6N56_RS26220	5582994	5583317		0	lycopene cyclase domain-containing protein	WP_085253829.1	
G6N56_RS26225	5583314	5584270		0	phytoene/squalene synthase family protein	WP_085253828.1	
G6N56_RS26230	5584267	5585799	crtI		phytoene desaturase family protein	WP_085253827.1	
G6N56_RS26235	5585828	5586949		0	polyprenyl synthetase family protein	WP_085253853.1	
G6N56_RS26240	5587219	5587713		0	MarR family transcriptional regulator	WP_085253826.1	
G6N56_RS26245	5588446	5589480		0	sulfate ABC transporter substrate-binding protein	WP_085253852.1	
G6N56_RS26250	5589477	5590370	cysT		sulfate ABC transporter permease subunit CysT	WP_085253825.1	
G6N56_RS26255	5590367	5591185	cysW		sulfate ABC transporter permease subunit CysW	WP_085253824.1	
G6N56_RS29335	5592365	5592463		0	phosphodiesterase		0
G6N56_RS26270	5592489	5593838		0	TIGR01777 family oxidoreductase	WP_085256951.1	
G6N56_RS26275	5593849	5595102		0	glycosyltransferase family 4 protein	WP_085256952.1	
G6N56_RS26280	5595195	5596766		0	glycoside hydrolase family 57 protein	WP_085256953.1	
G6N56_RS26285	5596763	5597587		0	class I SAM-dependent methyltransferase	WP_085256954.1	
G6N56_RS26290	5597869	5598660		0	electron transfer flavoprotein subunit beta/FixA family protein	WP_085256955.1	
G6N56_RS26295	5598696	5599652		0	electron transfer flavoprotein subunit alpha/FixB family protein	WP_085256956.1	
G6N56_RS29560	5599712	5599843		0	hypothetical protein	WP_264020691.1	
G6N56_RS26300	5600055	5600903		0	GNAT family N-acyltransferase	WP_085256957.1	
G6N56_RS26305	5600903	5601721		0	lysophospholipid acyltransferase family protein	WP_085256958.1	
G6N56_RS26310	5601755	5602936		0	cysteine desulfurase family protein	WP_085256959.1	
G6N56_RS26315	5602933	5604009	mnmA		tRNA 2-thiouridine(34) synthase MnmA	WP_142280715.1	
G6N56_RS26320	5604006	5604641		0	sensor domain-containing protein	WP_085256960.1	
G6N56_RS26325	5604728	5605741		0	methionine synthase	WP_085256961.1	
G6N56_RS26330	5605805	5607889	ligA		NAD-dependent DNA ligase LigA	WP_085256962.1	
G6N56_RS26335	5608017	5609261		0	glycosyltransferase	WP_085256963.1	
G6N56_RS26340	5609266	5609922		0	amino acid-binding protein	WP_142280716.1	
G6N56_RS26345	5610030	5610329	gatC		Asp-tRNA(Asn)/Glu-tRNA(Gln) amidotransferase subunit GatC	WP_085256969.1	
G6N56_RS26350	5610326	5611807	gatA		Asp-tRNA(Asn)/Glu-tRNA(Gln) amidotransferase subunit GatA	WP_085256965.1	

G6N56_RS26355	5611930	5612961		0	ATP-dependent 6-phosphofructokinase	WP_085256966.1
G6N56_RS26360	5613365	5614876	gatB		Asp-tRNA(Asn)/Glu-tRNA(Gln) amidotransferase subunit GatB	WP_085256967.1
G6N56_RS26365	5614943	5616064		0	sorbosone dehydrogenase family protein	WP_163645175.1
G6N56_RS26370	5616294	5617151		0	DoxX family protein	WP_085256175.1
G6N56_RS26380	5617834	5619693		0	acetolactate synthase large subunit	WP_085256177.1
G6N56_RS26385	5619693	5620199	ilvN		acetolactate synthase small subunit	WP_085256178.1
G6N56_RS26390	5620258	5621259	ilvC		ketol-acid reductoisomerase	WP_085256309.1
G6N56_RS26395	5621331	5621933	wrbA		NAD(P)H:quinone oxidoreductase	WP_085256179.1
G6N56_RS26400	5622162	5623748	serA		phosphoglycerate dehydrogenase	WP_085256180.1
G6N56_RS26410	5624655	5625959		0	MFS transporter	WP_085256182.1
G6N56_RS26415	5626063	5626860		0	fumarylacetoacetate hydrolase family protein	WP_085256183.1
G6N56_RS26420	5626857	5628323	gltX		glutamate--tRNA ligase	WP_085256184.1
G6N56_RS26435	5628697	5629209		0	PPOX class F420-dependent oxidoreductase	WP_085256185.1
G6N56_RS26440	5629539	5630240		0	IclR family transcriptional regulator	WP_085256186.1
G6N56_RS26445	5630308	5631756	leuC		3-isopropylmalate dehydratase large subunit	WP_085256187.1
G6N56_RS26450	5631775	5632371	leuD		3-isopropylmalate dehydratase small subunit	WP_085256188.1
G6N56_RS26455	5632586	5633221		0	HU family DNA-binding protein	WP_085256189.1
G6N56_RS26460	5633286	5634227		0	NUDIX hydrolase	WP_085256190.1
G6N56_RS26465	5634313	5636502		0	RNA degradosome polyphosphate kinase	WP_085256191.1
G6N56_RS26470	5636751	5637401	cofC		2-phospho-L-lactate guanylyltransferase	WP_085256310.1
G6N56_RS26475	5637625	5638644		0	NAD(P)H-dependent glycerol-3-phosphate dehydrogenase	WP_085256192.1
G6N56_RS26480	5638712	5639830		0	D-alanine--D-alanine ligase family protein	WP_085256193.1
G6N56_RS26485	5639827	5640399		0	DUF3515 domain-containing protein	WP_085256194.1
G6N56_RS26490	5640519	5641487		0	thiamine-phosphate kinase	WP_085256195.1
G6N56_RS26495	5641529	5642212		0	uracil-DNA glycosylase	WP_085256196.1
G6N56_RS26500	5642218	5642748		0	zinc-binding dehydrogenase	WP_085256197.1
G6N56_RS26505	5642781	5643431		0	nitroreductase	WP_085256198.1
G6N56_RS26510	5643442	5643636	rpmB		50S ribosomal protein L28	WP_085256199.1
G6N56_RS26515	5643904	5645586		0	DAK2 domain-containing protein	WP_085256200.1
G6N56_RS26520	5645589	5647814	recG		ATP-dependent DNA helicase RecG	WP_085256201.1
G6N56_RS26530	5648505	5649386		0	aldo/keto reductase	WP_142280642.1

G6N56_RS26535	5649393	5650238		0	aldo/keto reductase	WP_085256204.1
G6N56_RS26540	5650237	5651355		0	alpha/beta hydrolase	WP_085256205.1
G6N56_RS26545	5651447	5652214		0	DsbA family protein	WP_085256206.1
G6N56_RS26550	5652223	5652858		0	vitamin K epoxide reductase family protein	WP_085256311.1
G6N56_RS26555	5652888	5653442	rsmD		16S rRNA (guanine(966)-N(2))-methyltransferase RsmD	WP_085256207.1
G6N56_RS26560	5653467	5653943	coaD		pantetheine-phosphate adenylyltransferase	WP_085256208.1
G6N56_RS26565	5654595	5655548		0	amidohydrolase family protein	WP_085256210.1
G6N56_RS26570	5655573	5657270		0	thiamine pyrophosphate-requiring protein	WP_085256211.1
G6N56_RS26575	5657277	5658458		0	permease	WP_085256312.1
G6N56_RS26580	5658792	5659985		0	hypothetical protein	WP_085256212.1
G6N56_RS26585	5660000	5660269		0	hypothetical protein	WP_142280643.1
G6N56_RS26590	5660503	5661240		0	DivIVA domain-containing protein	WP_085256214.1
G6N56_RS26595	5661300	5661899		0	DUF177 domain-containing protein	WP_180150428.1
G6N56_RS26600	5661896	5662609	rnc		ribonuclease III	WP_085256216.1
G6N56_RS26605	5662758	5663618	mutM		DNA-formamidopyrimidine glycosylase	WP_085256217.1
G6N56_RS26610	5663742	5664155		0	OsmC family protein	WP_085256218.1
G6N56_RS26620	5664646	5668257	smc		chromosome segregation protein SMC	WP_085256220.1
G6N56_RS26625	5668320	5669618	ftsY		signal recognition particle-docking protein FtsY	WP_085256221.1
G6N56_RS26630	5669806	5671194		0	ammonium transporter	WP_085256222.1
G6N56_RS26635	5671191	5671529	glnB		nitrogen regulatory protein P-II	WP_085256223.1
G6N56_RS26640	5671575	5674040		0	[protein-PII] uridylyltransferase	WP_085256224.1
G6N56_RS26645	5674068	5675786		0	DEAD/DEAH box helicase	WP_180150430.1
G6N56_RS26650	5675889	5676683		0	alpha/beta fold hydrolase	WP_085256226.1
G6N56_RS26655	5676975	5678540	ffh		signal recognition particle protein	WP_085256227.1
G6N56_RS26660	5678548	5679633		0	amidohydrolase family protein	WP_085256228.1
G6N56_RS26665	5679687	5681381		0	serine/threonine-protein kinase	WP_085256229.1
G6N56_RS26670	5681482	5683296		0	amidohydrolase family protein	WP_085256230.1
G6N56_RS26675	5683298	5683894		0	TetR/AcrR family transcriptional regulator	WP_085256231.1
G6N56_RS26680	5683913	5684800		0	D-alanyl-D-alanine carboxypeptidase family protein	WP_085256232.1
G6N56_RS26685	5684846	5685103		0	hypothetical protein	WP_142280645.1
G6N56_RS26690	5685296	5685715		0	nuclear transport factor 2 family protein	WP_085256234.1

G6N56_RS26695	5685983	5686459	rpsP		30S ribosomal protein S16	WP_085256235.1
G6N56_RS26700	5686465	5686707		0	RNA-binding protein	WP_036354608.1
G6N56_RS26705	5686734	5687261	rimM		ribosome maturation factor RimM	WP_085256236.1
G6N56_RS26710	5687264	5687953	trmD		tRNA (guanosine(37)-N1)-methyltransferase TrmD	WP_085256237.1
G6N56_RS26715	5687965	5688906		0	hypothetical protein	WP_085256238.1
G6N56_RS26720	5689230	5689571	rplS		50S ribosomal protein L19	WP_085256239.1
G6N56_RS26725	5689640	5690503	lepB		signal peptidase I	WP_085256240.1
G6N56_RS26730	5690517	5691236		0	ribonuclease HII	WP_085256241.1
G6N56_RS26735	5691337	5691642		0	DUF2469 domain-containing protein	WP_085256242.1
G6N56_RS26740	5691651	5692415		0	GAF and ANTAR domain-containing protein	WP_085256243.1
G6N56_RS26745	5692608	5693588		0	SDR family oxidoreductase	WP_085256244.1
G6N56_RS26750	5693680	5694501	fdhD		formate dehydrogenase accessory sulfurtransferase FdhD	WP_085256245.1
G6N56_RS26755	5694605	5694991		0	YraN family protein	WP_085256246.1
G6N56_RS26760	5694991	5696502		0	YifB family Mg chelatase-like AAA ATPase	WP_085256247.1
G6N56_RS26765	5696499	5697644	dprA		DNA-processing protein DprA	WP_085256248.1
G6N56_RS26770	5697679	5698536		0	siderophore-interacting protein	WP_085256249.1
G6N56_RS26775	5698561	5699721		0	lactate 2-monooxygenase	WP_085256250.1
G6N56_RS26780	5699755	5700651		0	tyrosine recombinase XerC	WP_085256251.1
G6N56_RS26790	5701539	5702396	rpsB		30S ribosomal protein S2	WP_085256252.1
G6N56_RS26795	5702400	5703215	tsf		translation elongation factor Ts	WP_085256253.1
G6N56_RS26800	5703239	5704645		0	amidase	WP_085256254.1
G6N56_RS26805	5704647	5705069		0	MarR family transcriptional regulator	WP_085256314.1
G6N56_RS26810	5705183	5705596		0	ChaB family protein	WP_085256255.1
G6N56_RS26815	5705640	5705969		0	DUF2795 domain-containing protein	WP_085256256.1
G6N56_RS26820	5706009	5706608		0	SDR family oxidoreductase	WP_085256257.1
G6N56_RS26825	5706932	5707414		0	MPT63 family protein	WP_085256315.1
G6N56_RS26830	5707539	5708474		0	lysophospholipid acyltransferase family protein	WP_085256316.1
G6N56_RS26835	5708507	5709253		0	response regulator transcription factor	WP_085256317.1
G6N56_RS26840	5709485	5709724		0	CsbD family protein	WP_085256258.1
G6N56_RS26845	5710069	5710857	pyrH		UMP kinase	WP_163645176.1
G6N56_RS26850	5710932	5711489	frr		ribosome recycling factor	WP_085256259.1

G6N56_RS26855	5711521	5712450		0	phosphatidate cytidylyltransferase	WP_085256260.1
G6N56_RS26860	5712472	5713572	rlmN		23S rRNA (adenine(2503)-C(2))-methyltransferase RlmN	WP_085256261.1
G6N56_RS26865	5713815	5714324		0	protein disulfide oxidoreductase	WP_085256262.1
G6N56_RS26870	5714329	5715207		0	cytochrome c biogenesis CcdA family protein	WP_085256263.1
G6N56_RS26875	5715208	5715471		0	DUF2631 domain-containing protein	WP_085256264.1
G6N56_RS26880	5715588	5716811	dxr		1-deoxy-D-xylulose-5-phosphate reductoisomerase	WP_163645177.1
G6N56_RS26885	5716819	5718033		0	M50 family metallopeptidase	WP_085256266.1
G6N56_RS26890	5718056	5719216	ispG		flavodoxin-dependent (E)-4-hydroxy-3-methylbut-2-enyl-diphos	WP_085256267.1
G6N56_RS26895	5719273	5720127		0	GNAT family N-acetyltransferase	WP_085256268.1
G6N56_RS26900	5720162	5722912		0	AAA family ATPase	WP_085256319.1
G6N56_RS26905	5723119	5724774		0	thiamine pyrophosphate-binding protein	WP_085256269.1
G6N56_RS26910	5724927	5726018		0	cupin domain-containing protein	WP_085256270.1
G6N56_RS26915	5726155	5727984		0	penicillin-binding transpeptidase domain-containing protein	WP_085256271.1
G6N56_RS26920	5728029	5728613		0	DUF1707 domain-containing protein	WP_085256272.1
G6N56_RS26925	5728661	5729518	map		type I methionyl aminopeptidase	WP_085256273.1
G6N56_RS26930	5729598	5731100		0	cobyric acid synthase	WP_085256274.1
G6N56_RS26935	5731217	5731768		0	alpha/beta hydrolase	WP_085256320.1
G6N56_RS26940	5731789	5733165		0	glutamine synthetase family protein	WP_085256275.1
G6N56_RS26950	5733895	5735259		0	aldehyde dehydrogenase family protein	WP_085256277.1
G6N56_RS26955	5735256	5736032		0	3-oxoacyl-ACP reductase	WP_163645178.1
G6N56_RS26960	5736040	5736924		0	PPE family protein	WP_085256279.1
G6N56_RS26970	5738072	5739292		0	PPE family protein	WP_085256280.1
G6N56_RS26975	5739822	5740940		0	alkaline phosphatase family protein	WP_085256321.1
G6N56_RS26980	5740960	5741703		0	DUF6390 family protein	WP_264020456.1
G6N56_RS26985	5741700	5742839	hypD		hydrogenase formation protein HypD	WP_085256281.1
G6N56_RS26990	5742856	5743128		0	HypC/HybG/HupF family hydrogenase formation chaperone	WP_085256282.1
G6N56_RS26995	5743333	5744445	hypE		hydrogenase expression/formation protein HypE	WP_085256283.1
G6N56_RS27000	5744442	5745110		0	SIS domain-containing protein	WP_085256284.1
G6N56_RS27005	5745110	5745835		0	hydrogenase assembly protein HupF	WP_085256285.1
G6N56_RS27010	5745832	5748165	hypF		carbamoyltransferase HypF	WP_085256286.1
G6N56_RS27020	5748406	5748942		0	hydrogenase maturation protease	WP_085256288.1

G6N56_RS27025	5749092	5750423		0	hypothetical protein	WP_085256289.1
G6N56_RS27030	5750420	5751061		0	DUF6084 family protein	WP_085256290.1
G6N56_RS27035	5751058	5751699		0	DUF5947 family protein	WP_085256291.1
G6N56_RS27040	5751696	5752562		0	NifU family protein	WP_085256292.1
G6N56_RS27045	5752838	5754637		0	nickel-dependent hydrogenase large subunit	WP_085256293.1
G6N56_RS27050	5754703	5755758		0	hydrogenase expression protein HypE	WP_085256294.1
G6N56_RS27055	5755998	5756786	hypB		hydrogenase nickel incorporation protein HypB	WP_085256295.1
G6N56_RS27060	5756816	5757151		0	hydrogenase maturation nickel metallochaperone HypA	WP_085256296.1
G6N56_RS27065	5757251	5758366		0	HoxN/HupN/NixA family nickel/cobalt transporter	WP_085256297.1
G6N56_RS27070	5758504	5758965		0	Fur family transcriptional regulator	WP_085256298.1
G6N56_RS27075	5758975	5760357	mtr		mycothione reductase	WP_085256323.1
G6N56_RS27080	5760374	5761384		0	alpha/beta hydrolase	WP_085256325.1
G6N56_RS27085	5761478	5762974	mqo		malate dehydrogenase (quinone)	WP_264020455.1
G6N56_RS27090	5762971	5763441		0	GNAT family N-acetyltransferase	WP_085256299.1
G6N56_RS27095	5763438	5765261		0	magnesium chelatase subunit D family protein	WP_085256300.1
G6N56_RS27100	5765277	5765891	cobO		cob(I)yrinic acid a,c-diamide adenosyltransferase	WP_085256301.1
G6N56_RS27105	5765888	5767273		0	cobyrinate a,c-diamide synthase	WP_232069159.1
G6N56_RS27110	5767355	5768590	cobA		uroporphyrinogen-III C-methyltransferase	WP_085256303.1
G6N56_RS27115	5768613	5770205		0	MFS transporter	WP_085256304.1
G6N56_RS27120	5770217	5770996		0	hypothetical protein	WP_085256305.1
G6N56_RS27125	5770993	5771421		0	hypothetical protein	WP_085256306.1
G6N56_RS27130	5771494	5772063		0	hypothetical protein	WP_085256307.1
G6N56_RS27135	5772066	5772374		0	PE domain-containing protein	WP_085256308.1
G6N56_RS27140	5772377	5774011		0	PPE family protein	WP_180150432.1
G6N56_RS27145	5774090	5775850		0	proline--tRNA ligase	WP_085257955.1
G6N56_RS27150	5775858	5776304		0	ferritin-like domain-containing protein	WP_085257956.1
G6N56_RS27155	5776301	5776846		0	hypothetical protein	WP_163645179.1
G6N56_RS27160	5777051	5777593	rimP		ribosome maturation factor RimP	WP_085256419.1
G6N56_RS27165	5777727	5778767	nusA		transcription termination factor NusA	WP_085256418.1
G6N56_RS27170	5778828	5779199		0	YlxR family protein	WP_085256417.1
G6N56_RS27175	5779287	5782082	infB		translation initiation factor IF-2	WP_085256416.1

G6N56_RS27185	5782655	5783665		0	bifunctional oligoribonuclease/PAP phosphatase NrnA	WP_085256414.1
G6N56_RS27190	5783673	5784245		0	hypothetical protein	WP_085256413.1
G6N56_RS27195	5784330	5784722		0	hypothetical protein	WP_085256412.1
G6N56_RS27200	5784738	5785487		0	enoyl-CoA hydratase	WP_085256411.1
G6N56_RS27205	5785557	5786006		0	PAS domain S-box protein	WP_232069160.1
G6N56_RS27210	5786156	5787211		0	LysR family transcriptional regulator	WP_232069161.1
G6N56_RS27215	5787383	5789107	oxc		oxalyl-CoA decarboxylase	WP_085256408.1
G6N56_RS27220	5789182	5790438	frc		formyl-CoA transferase	WP_085256407.1
G6N56_RS27225	5790662	5790844		0	hypothetical protein	WP_085256406.1
G6N56_RS27230	5791289	5792518	frc		formyl-CoA transferase	WP_085256426.1
G6N56_RS27235	5792954	5794213		0	OFA family MFS transporter	WP_232069162.1
G6N56_RS27245	5795147	5796322		0	NAD-dependent formate dehydrogenase	WP_085256403.1
G6N56_RS27255	5797428	5798453		0	2-dehydropantoate 2-reductase	WP_085256402.1
G6N56_RS27260	5798485	5799726	pyk		pyruvate kinase	WP_085256401.1
G6N56_RS27265	5799808	5800737		0	histone deacetylase family protein	WP_085256400.1
G6N56_RS27270	5800753	5801130		0	hypothetical protein	WP_085256399.1
G6N56_RS27280	5801869	5802141		0	DUF2277 family protein	WP_085256397.1
G6N56_RS27290	5802657	5804360		0	CocE/NonD family hydrolase	WP_085256395.1
G6N56_RS27295	5804388	5804972		0	DUF3558 domain-containing protein	WP_085256425.1
G6N56_RS27300	5805145	5806110		0	metallophosphoesterase	WP_085256424.1
G6N56_RS27305	5806107	5806805		0	4'-phosphopantetheinyl transferase	WP_085256394.1
G6N56_RS27310	5806802	5807722	truB		tRNA pseudouridine(55) synthase TruB	WP_085256393.1
G6N56_RS27315	5807883	5808683		0	SHOCT domain-containing protein	WP_085256392.1
G6N56_RS27320	5808849	5810039		0	lipid-transfer protein	WP_085256391.1
G6N56_RS27325	5810047	5811279		0	acyl-CoA dehydrogenase family protein	WP_085256390.1
G6N56_RS27330	5811466	5812170	mntR		manganese-binding transcriptional regulator MntR	WP_085256389.1
G6N56_RS27335	5812322	5813296		0	bifunctional riboflavin kinase/FAD synthetase	WP_085256388.1
G6N56_RS27340	5813451	5813720	rpsO		30S ribosomal protein S15	WP_085256423.1
G6N56_RS27345	5814084	5816357		0	polyribonucleotide nucleotidyltransferase	WP_163645180.1
G6N56_RS27355	5817722	5818744		0	nitronate monooxygenase	WP_085256385.1
G6N56_RS27360	5818758	5819873	ald		alanine dehydrogenase	WP_085256384.1

G6N56_RS27365	5819986	5820447		0	SRPBCC family protein	WP_085256383.1
G6N56_RS27370	5820492	5822282		0	SDR family oxidoreductase	WP_085256382.1
G6N56_RS27380	5822818	5823411		0	TetR family transcriptional regulator	WP_085256421.1
G6N56_RS27385	5823560	5824558		0	Rieske 2Fe-2S domain-containing protein	WP_085256381.1
G6N56_RS27390	5824565	5825653		0	dihydrodipicolinate reductase	WP_085256380.1
G6N56_RS27395	5825661	5827022		0	HNH endonuclease signature motif containing protein	WP_085256379.1
G6N56_RS27400	5827152	5827460		0	EAL domain-containing protein	WP_158090725.1
G6N56_RS27405	5827674	5828411	dapB		4-hydroxy-tetrahydrodipicolinate reductase	WP_085256420.1
G6N56_RS27410	5828408	5828863		0	hypothetical protein	WP_085256377.1
G6N56_RS27415	5828860	5829315		0	flavodoxin family protein	WP_085256376.1
G6N56_RS27420	5829334	5830893		0	DUF4118 domain-containing protein	WP_085256375.1
G6N56_RS27425	5830897	5831541		0	response regulator transcription factor	WP_085256374.1
G6N56_RS27430	5831724	5831924		0	hypothetical protein	WP_142280655.1
G6N56_RS27435	5832038	5833561		0	class I adenylate-forming enzyme family protein	WP_085256373.1
G6N56_RS27440	5833558	5836797		0	carboxyl transferase domain-containing protein	WP_085256372.1
G6N56_RS27445	5836798	5837124		0	PE-PGRS family protein	WP_085256371.1
G6N56_RS27450	5837224	5838006		0	SDR family oxidoreductase	WP_085256370.1
G6N56_RS27455	5838003	5839184		0	acyl-CoA dehydrogenase family protein	WP_085256369.1
G6N56_RS27460	5839181	5840275		0	acyl-CoA dehydrogenase family protein	WP_085256368.1
G6N56_RS27470	5840477	5841217		0	dienelactone hydrolase family protein	WP_085256366.1
G6N56_RS27475	5841507	5842307		0	thymidylate synthase	WP_085257693.1
G6N56_RS27480	5842304	5842798		0	dihydrofolate reductase	WP_085257692.1
G6N56_RS27485	5842898	5843644		0	DivIVA domain-containing protein	WP_085257719.1
G6N56_RS27490	5843682	5844908		0	crosslink repair DNA glycosylase YcaQ family protein	WP_085257691.1
G6N56_RS27495	5844908	5846311		0	type I restriction-modification system subunit M	WP_085257690.1
G6N56_RS27500	5846308	5847384		0	restriction endonuclease subunit S	WP_085257689.1
G6N56_RS27505	5847395	5848147	thyX		FAD-dependent thymidylate synthase	WP_085257718.1
G6N56_RS27510	5848209	5849111	dapA		4-hydroxy-tetrahydrodipicolinate synthase	WP_085257688.1
G6N56_RS27515	5849131	5850807		0	ribonuclease J	WP_085257687.1
G6N56_RS27520	5850897	5851769		0	SAM-dependent methyltransferase	WP_085257686.1
G6N56_RS27525	5851773	5852606		0	mycofactocin-coupled SDR family oxidoreductase	WP_085257685.1

G6N56_RS27530	5852606	5852920		0	putative quinol monooxygenase	WP_085257684.1
G6N56_RS27535	5853090	5855603		0	DNA translocase FtsK	WP_180150438.1
G6N56_RS27540	5855670	5856185		0	amino-acid N-acetyltransferase	WP_180150440.1
G6N56_RS27545	5856242	5856835	pgsA		CDP-diacylglycerol--glycerol-3-phosphate 3-phosphatidyltrans	WP_085257681.1
G6N56_RS27550	5856965	5857303		0	transcriptional regulator ClgR	WP_085257680.1
G6N56_RS27555	5857426	5858244	pspA		phage shock protein PspA	WP_085257679.1
G6N56_RS27560	5858263	5859075		0	hypothetical protein	WP_085257678.1
G6N56_RS27565	5859089	5859520		0	limonene-1,2-epoxide hydrolase family protein	WP_085257677.1
G6N56_RS27570	5859573	5860739		0	glycosyltransferase	WP_085257676.1
G6N56_RS27575	5860750	5860956		0	DUF3046 domain-containing protein	WP_142280781.1
G6N56_RS27580	5861063	5861710		0	(2Fe-2S)-binding protein	WP_085257717.1
G6N56_RS27585	5861873	5862925	recA		recombinase RecA	WP_085257674.1
G6N56_RS27595	5863473	5864975	miaB		tRNA (N6-isopentenyl adenosine(37)-C2)-methylthiotransferas	WP_163645235.1
G6N56_RS27605	5865562	5866926		0	DUF349 domain-containing protein	WP_085257670.1
G6N56_RS27610	5867345	5868334		0	DMT family transporter	WP_169717545.1
G6N56_RS27615	5868365	5869048		0	hypothetical protein	WP_085257669.1
G6N56_RS27620	5869045	5869962	miaA		tRNA (adenosine(37)-N6)-dimethylallyltransferase MiaA	WP_085257668.1
G6N56_RS27625	5870020	5870904	dapF		diaminopimelate epimerase	WP_085257667.1
G6N56_RS27630	5870951	5872372	hflX		GTPase HflX	WP_085257666.1
G6N56_RS27635	5872553	5873713		0	acyl-CoA dehydrogenase family protein	WP_085257665.1
G6N56_RS27640	5874205	5876412		0	LGFP repeat-containing protein	WP_085257664.1
G6N56_RS27645	5876443	5877141	lexA		transcriptional repressor LexA	WP_085257663.1
G6N56_RS27650	5877433	5877927		0	LysM peptidoglycan-binding domain-containing protein	WP_085257662.1
G6N56_RS27655	5878089	5878553	nrdR		transcriptional regulator NrdR	WP_085257661.1
G6N56_RS27660	5878589	5879074		0	FABP family protein	WP_085257660.1
G6N56_RS27665	5879127	5880158		0	alpha/beta hydrolase	WP_085257659.1
G6N56_RS27670	5880261	5881235		0	PAC2 family protein	WP_085257658.1
G6N56_RS27675	5881434	5882258		0	trypsin-like peptidase domain-containing protein	WP_085257657.1
G6N56_RS27680	5882255	5883670	sthA		Si-specific NAD(P)(+) transhydrogenase	WP_085257656.1
G6N56_RS27685	5883784	5884842		0	DUF4192 domain-containing protein	WP_085257655.1
G6N56_RS27690	5884864	5885559	ideR		iron-dependent transcriptional regulator IdeR	WP_085257654.1

G6N56_RS27695	5885692	5886651	sigB	sigma-70 family RNA polymerase sigma factor SigB	WP_085257653.1
G6N56_RS27700	5886801	5887214	0	DUF3099 domain-containing protein	WP_085257652.1
G6N56_RS27705	5887278	5887517	0	DUF3039 domain-containing protein	WP_085257716.1
G6N56_RS27710	5887518	5888507	0	YihY/virulence factor BrkB family protein	WP_085257651.1
G6N56_RS27715	5888574	5888759	0	hypothetical protein	WP_085257650.1
G6N56_RS27720	5888895	5889257	0	DUF952 domain-containing protein	WP_232069165.1
G6N56_RS27725	5889336	5890871	0	RNA polymerase sigma factor	WP_085257648.1
G6N56_RS27730	5891058	5891855	0	ROK family protein	WP_085257647.1
G6N56_RS27735	5891968	5892840	0	inositol monophosphatase family protein	WP_085257646.1
G6N56_RS27740	5892844	5893494	cei	envelope integrity protein Cei	WP_085257645.1
G6N56_RS27745	5893686	5893988	0	DUF4193 domain-containing protein	WP_067115263.1
G6N56_RS27750	5894001	5894477	0	DUF3093 domain-containing protein	WP_085257644.1
G6N56_RS27755	5894503	5894967	dut	dUTP diphosphatase	WP_085257643.1
G6N56_RS27760	5894967	5895725	0	DUF3710 domain-containing protein	WP_085257642.1
G6N56_RS27765	5895722	5896420	0	hypothetical protein	WP_085257641.1
G6N56_RS27770	5896569	5896937	0	OB-fold nucleic acid binding domain-containing protein	WP_085257640.1
G6N56_RS27775	5896994	5897629	0	DUF3159 domain-containing protein	WP_232069404.1
G6N56_RS27780	5898526	5899353	0	mycofactocin-coupled SDR family oxidoreductase	WP_085257638.1
G6N56_RS27785	5899370	5900854	0	aldehyde dehydrogenase family protein	WP_085257637.1
G6N56_RS27790	5900947	5901705	0	ABC transporter permease	WP_085257715.1
G6N56_RS27795	5901738	5902556	0	ABC transporter permease	WP_085257714.1
G6N56_RS27800	5902562	5903962	0	MCE family protein	WP_085257636.1
G6N56_RS27805	5903975	5905006	0	MCE family protein	WP_085257635.1
G6N56_RS27810	5905003	5906337	0	MCE family protein	WP_085257634.1
G6N56_RS27840	5911073	5911516	0	hypothetical protein	0
G6N56_RS27845	5911636	5912178	0	hypothetical protein	WP_232069405.1
G6N56_RS27850	5912385	5913017	0	CAP domain-containing protein	WP_085257629.1
G6N56_RS29350	5913301	5913606	0	DUF732 domain-containing protein	WP_232069166.1
G6N56_RS27860	5913699	5914835	0	cytochrome P450	WP_197746648.1
G6N56_RS27870	5915855	5917048	0	thiolase family protein	WP_142280777.1
G6N56_RS27875	5917215	5917535	0	acyl-CoA synthase	WP_142280776.1

G6N56_RS27880	5917662	5918813		0	amidohydrolase family protein	WP_142280775.1
G6N56_RS27890	5919408	5920544		0	acyl-CoA dehydrogenase family protein	WP_085257625.1
G6N56_RS27895	5920549	5921451		0	acyl-CoA dehydrogenase family protein	WP_085257624.1
G6N56_RS27900	5921448	5921933		0	MaoC family dehydratase N-terminal domain-containing protei	WP_085257623.1
G6N56_RS27905	5921930	5922361		0	MaoC family dehydratase N-terminal domain-containing protei	WP_142280774.1
G6N56_RS27910	5922389	5923537		0	acyl-CoA dehydrogenase family protein	WP_085257621.1
G6N56_RS27915	5923571	5924662		0	acyl-CoA dehydrogenase family protein	WP_158090758.1
G6N56_RS27920	5924704	5925897		0	acyl-CoA dehydrogenase family protein	WP_085257619.1
G6N56_RS27925	5925897	5926751		0	TIGR03619 family F420-dependent LLM class oxidoreductase	WP_232069407.1
G6N56_RS27930	5926781	5926969		0	ferredoxin	WP_085257617.1
G6N56_RS27935	5927080	5927847		0	FCD domain-containing protein	WP_085257616.1
G6N56_RS27940	5928188	5929768		0	AMP-binding protein	WP_197746649.1
G6N56_RS27945	5930017	5931177		0	cytochrome P450	WP_158090757.1
G6N56_RS27950	5931453	5932205		0	3-hydroxyacyl-CoA dehydrogenase	WP_085257613.1
G6N56_RS27955	5932374	5933615		0	cytochrome P450	WP_085257612.1
G6N56_RS27960	5934459	5935346		0	TIGR03619 family F420-dependent LLM class oxidoreductase	WP_085257611.1
G6N56_RS27965	5935394	5936251		0	NAD(P)-dependent oxidoreductase	WP_158090756.1
G6N56_RS27970	5936543	5937457		0	MBL fold metallo-hydrolase	WP_085257609.1
G6N56_RS27975	5937741	5938418		0	TrkA family potassium uptake protein	WP_085257608.1
G6N56_RS27980	5938415	5939098		0	TrkA family potassium uptake protein	WP_142280772.1
G6N56_RS27985	5939231	5941225		0	APC family permease	WP_085257606.1
G6N56_RS27990	5941222	5942418		0	TRAM domain-containing protein	WP_232069168.1
G6N56_RS27995	5942540	5944039		0	cation:proton antiporter	WP_085257604.1
G6N56_RS28000	5944098	5946011	dxs		1-deoxy-D-xylulose-5-phosphate synthase	WP_085257603.1
G6N56_RS28005	5946048	5947337		0	ribonuclease D	WP_085257602.1
G6N56_RS28010	5947339	5947920		0	DUF3000 domain-containing protein	WP_232069169.1
G6N56_RS28015	5948040	5948900		0	enoyl-CoA hydratase/isomerase family protein	WP_142280771.1
G6N56_RS28020	5948909	5949970	hemE		uroporphyrinogen decarboxylase	WP_085257599.1
G6N56_RS28025	5949967	5951319		0	protoporphyrinogen oxidase	WP_085257598.1
G6N56_RS28030	5951325	5952020		0	chlorite dismutase family protein	WP_085257597.1
G6N56_RS28035	5952017	5952769		0	class I SAM-dependent methyltransferase	WP_085257596.1

G6N56_RS28040	5952919	5953776		0	hypothetical protein	WP_085257595.1
G6N56_RS28045	5953846	5954253	msrB		peptide-methionine (R)-S-oxide reductase MsrB	WP_085257594.1
G6N56_RS28050	5954250	5955560	aftC		arabinofuranan 3-O-arabinosyltransferase	WP_085257593.1
G6N56_RS28055	5955580	5957139		0	alpha/beta hydrolase	WP_085257592.1
G6N56_RS28060	5957154	5957930		0	pyrimidine reductase family protein	WP_085257591.1
G6N56_RS28065	5957929	5959026	zapE		cell division protein ZapE	WP_142280770.1
G6N56_RS28075	5959519	5960040		0	hypothetical protein	WP_085257588.1
G6N56_RS28080	5960085	5960822		0	Clp protease N-terminal domain-containing protein	WP_085257587.1
G6N56_RS28085	5960931	5961260		0	DUF732 domain-containing protein	WP_085257586.1
G6N56_RS28090	5961382	5962899		0	sodium:alanine symporter family protein	WP_085257585.1
G6N56_RS28095	5962918	5963937		0	acetylserotonin O-methyltransferase	WP_232069170.1
G6N56_RS28100	5964000	5964962		0	alpha/beta hydrolase	WP_085257584.1
G6N56_RS28105	5965099	5965752		0	hypothetical protein	WP_142280769.1
G6N56_RS28110	5965749	5966672		0	cation diffusion facilitator family transporter	WP_085257582.1
G6N56_RS28115	5966733	5967902		0	glycosyltransferase	WP_085257581.1
G6N56_RS28120	5967906	5968535		0	hypothetical protein	WP_085257580.1
G6N56_RS28125	5968744	5968944		0	hypothetical protein	WP_163645184.1
G6N56_RS28130	5969010	5969459		0	hypothetical protein	WP_085257579.1
G6N56_RS28135	5969524	5969724		0	hypothetical protein	WP_085257578.1
G6N56_RS28140	5969816	5970046		0	hypothetical protein	WP_085257577.1
G6N56_RS28165	5971117	5971638		0	DUF5994 family protein	WP_085257709.1
G6N56_RS28170	5972157	5972969		0	transglutaminase family protein	WP_085257708.1
G6N56_RS28175	5973067	5973777		0	N-formylglutamate amidohydrolase	WP_085257576.1
G6N56_RS28180	5973793	5974500		0	peptidase	WP_085257575.1
G6N56_RS28195	5976431	5977369		0	zinc-binding dehydrogenase	WP_232069171.1
G6N56_RS28205	5978216	5980789		0	SpoIIE family protein phosphatase	WP_085257572.1
G6N56_RS28210	5980789	5981115		0	STAS domain-containing protein	WP_085257571.1
G6N56_RS28215	5981263	5981553		0	hypothetical protein	WP_085257707.1
G6N56_RS28220	5981621	5982448		0	enoyl-CoA hydratase/isomerase family protein	WP_085257570.1
G6N56_RS28225	5982569	5983618		0	AraC family transcriptional regulator	WP_142280768.1
G6N56_RS28230	5983726	5985090		0	NAD(P)-binding domain-containing protein	WP_158090755.1

G6N56_RS28240	5986588	5987775	0	lipid-transfer protein	WP_142280767.1
G6N56_RS28245	5987778	5988290	0	MaoC family dehydratase N-terminal domain-containing protei	WP_085257565.1
G6N56_RS28250	5988287	5988697	0	MaoC family dehydratase	WP_085257564.1
G6N56_RS28255	5988717	5989544	0	SDR family oxidoreductase	WP_085257563.1
G6N56_RS28260	5989553	5990701	0	acyl-CoA dehydrogenase family protein	WP_085257562.1
G6N56_RS28345	6006700	6007326	0	NmrA family NAD(P)-binding protein	WP_269473805.1
G6N56_RS28355	6007597	6008760	0	FAD-dependent oxidoreductase	WP_163645187.1
